# Supplementary material for: Six-month follow-up of a booster dose of CoronaVac in two single-centre phase 2 clinical trials
Source: Nat Commun. 2022 Jun 3;13:3100. doi: 10.1038/s41467-022-30864-w (PMC9166693; doi:10.1038/s41467-022-30864-w)
Supplement: Supplementary file 1 — Supplementary Information [file 41467_2022_30864_MOESM1_ESM.pdf]

Content

Supplementary Neutralization Assay. Detection of Neutralization Potency against Live SARS-CoV-2 2

Supplementary Visit Plan. Essential steps and timing for each visit 6

Supplementary Study endpoints 7

Supplementary Figure 1. Trial profile 9

Supplementary Figure 2. Level of neutralising antibodies to infectious SARS-CoV-2 in adults aged 18-59 years 12

Supplementary Figure 3. Decline in neutralizing antibodies to ancestral SARS-CoV-2 in adults aged 18-59 years 13

Supplementary Figure 4. Decline in neutralizing antibodies to ancestral SARS-CoV-2 in adults aged 60 years and older 14

Supplementary Table 1. Baseline demographic characteristics in the safety population received the third dose 15

Supplementary Table 2. Results of immunological endpoints in adults aged 18-59 years old 16

Supplementary Table 3. Results of immunological endpoints in adults aged 60 years and older 18

Supplementary Table 4. Sensitivity analysis of lower dilution in adults aged 18-59 years old in cohort 2b-28d-8m 19

Supplementary Table 5. Sensitivity analysis of higher dilution in adults aged 18-59 years old in cohort 2b-28d-8m 20

Supplementary Table 6. Sensitivity analysis of lower dilution in adults aged 60 years and older in cohort 3-28d-8m 21

Supplementary Table 7. Sensitivity analysis of higher dilution in adults aged 60 years and older in cohort 3-28d-8m 22

Supplementary Table 8. Serious adverse events by system organ class and preferred term reported during 6-month follow-up after administration of booster dose among adults aged 18-59 years old (cohort 2b-28d-8m) 23

Supplementary Table 9. Serious adverse events by system organ class and preferred term reported during 6-month follow-up after administration of third dose among adults aged 60 years and older (cohort 3-28d-8m) 24

Supplementary Neutralization Assay. Detection of Neutralization Potency against Live SARS-CoV-2

Cells: Vero cells obtained from kidney tissue of adult African green monkey (W.H.O.VERO SEED LOT 10-87) were used in the neutralization assay, which was the 10th generation passaged from original ATCC Vero CCL-81. Cell line was authenticated by cell morphology examination and species identification (PCR) and tested negative for mycoplasma by culture method and indicator cell culture medium (DNA staining).

Virus: SARS-CoV-2/human/CHN/CN1/2020 (GenBank number MT407649.1, https://www.ncbi.nlm.nih.gov/nuccore/MT407649.1) were used in the neutralization assay. Second generation sequencing for the Spike protein was conducted and the results showed complete agreement between the virus used in neutralization assays and the original vaccine virus, excluding the possibility that any mutations existed that could affect neutralizing potency.

Viral growth in cell culture: Cells were seeded in T175 flasks and the cell monolayer was washed with sterile phosphate buffered saline (PBS). After removal of the PBS, the cells were infected with 0.04mL/cm2 of medium containing the virus. The cell-virus mixture was incubated at 37°C in a humidified atmosphere with 5% CO2 for 5-10 days. The flasks were observed every two days and the virus was harvested when 70%-90% of the cells manifested CPE. The flask was placed horizontally at -20±2℃ for more than two hours to harvest the virus. After unfreezing propagated virus, the culture medium was centrifuged to remove the cell debris, then they aliquoted and stored at -70°C.

Serum treatment: all serum samples were inactivated at 56°C in a water bath for 30 minutes.

Medium addition: cell maintenance medium was added to the cell control group at 100 μL/well, and 50 μL/well of maintenance medium was supplemented to the to-be-tested serum group, virus back titration group and positive control group from the second dilution.

Dilution of the serum sample: The serum was serially diluted from 1:4 to 1:8192 with a four-fold dilution (60 μL sample + 180 μL maintenance medium) using cell

maintenance medium (2% newborn calf serum-199 (2% sodium hydrogen carbonate) cell maintenance medium). The diluted serum was added to the cell plate at 100 µL/well, and each sample was diluted to 2 wells in parallel. 50 µL of the mixture in the first dilution was pipetted into the next dilution, and the mixture was pipetted up and down for 8-10 times. The mixture was diluted to the appropriate dilution range by this method, and 50 µL of the last dilution was discarded, and 50 µL of the diluted sample was retained in each well. The initial dilution of serum sample, rather than serum-virus mixture, was used to serve as the lower limit of detection (1:4)

Dilution of the virus for neutralization: the SARS-CoV-2 used for neutralization was diluted to 100$CCID_{50}$/0.05ml by titer.

Neutralization: Serum of different dilutions was mixed with 100 CCID50/0.05ml virus liquid in equal volume (50 µL+50 µL), respectively, and then incubated in an incubator at 36.5°C, 5%CO_2 for 2h.

Virus Back Titration: The virus suspension diluted to 100 CCID50/0.05 mL was diluted via ten-fold serial dilution, i.e. diluted to 10 CCID50/0.05 mL, 1 CCID50/0.05 mL and 0.1 $CCID_{50}$/0.05 mL, and added to the 96-well cell plate respectively, 8 wells per dilution and 50 µL per well, then 50 µL of cell maintenance medium was added to each well, and the plate was incubated in an incubator at 36.5°C, 5% CO2 for 5 days. The end-point titres were calculated according to the Behrens-Karber method based on eight replicates for titration.

Interpretation of the Results: Wells were observed for cytopathic effect after being cultured for 3-5 days, and the neutralizing antibody titers of the to-be-tested serum samples were determined according to the observed results of the cytopathic effect (CPE). The reciprocal of the highest serum dilution without cytopathic effect is the end titer. When 1 of the 2 wells of the highest dilution serum shows CPE and the other does not, the reciprocal of the dilution should be the neutralizing antibody titre of the serum specimen; the reciprocal of the mean dilution of the two wells should be the neutralizing antibody titre of the serum specimen when the 2 wells with the highest dilution are completely pathological while the adjacent 2 wells with low dilution are not pathological completely; when 1 of two adjacent wells is pathological while the other not, the reciprocal of the average dilutions of 2 wells should be the neutralizing antibody titer of the serum specimen. For example, 2 wells with high

dilution of 1:16 have a complete CPE, while the adjacent 2 wells with low dilution of 1:8 have no CPE; or in 2 adjacent wells with dilutions of 1:8 and 1:16, one has a CPE, while the other does not. In this case, the reciprocal 12 of the average dilutions of 2 wells is the neutralizing antibody titer of the serum. Sensitivity analyses only adopting higher dilutions or lower dilutions were conducted to ensure that use of the average would not deflate or inflate the values of nAb titers (See Supplementary tables 5-8).

Experimental control: Negative serum control, positive serum control, serum sample and cell control were set for each batch of assay simultaneously.

The following validations were implemented to ensure the specificity, accuracy, and applicability of neutralization assay:

Table 1. Validation of specificity of neutralization assay

Samples	Result	Positive/ Negative	Whether acceptable
Hepatitis A mab ascites	<1: 4	N	Yes
EV71 positive serum control	<1: 4	N	Yes
Poliovirus type I bovine serum	<1: 4	N	Yes
Influenza A(H1N1) positive serum control	<1: 4	N	Yes
Influenza A(H3N2) positive serum control	<1: 4	N	Yes
Influenza B positive serum control	<1: 4	N	Yes
Influenza A(H5N1) positive serum control	<1: 4	N	Yes
SARS-CoV-2 goat antiserum	1: 1024	P	Yes
COVID-19 convalescent patient	1: 96	P	Yes

Table 2 Validation of accuracy of neutralization assay

Staff	Sample	Neutralization titer （1: ）			Geometric mean value （1: ）	Reference range （1: ）
		Day 1	Day 2	Day 3		
A	SARS-CoV-2	768/768	1024/512	1536/512	768	384~1536

B	goat antiserum	768/512	768/384	1536/768	768	384~1536
**Geometric mean value（1: ）**		768	512	768	/	
**Reference range（1: ）**		384~1536	256~1024	384~1536		

Table 3 Validation of applicability of neutralization assay

Sample	Result	Acceptable standard	Whether acceptable
Negative serum control	<1: 4	Negative	Yes
COVID-19 convalescent patient 1	96	Positive	Yes
COVID-19 convalescent patient 2	24	Positive	Yes
COVID-19 convalescent patient 3	24	Positive	Yes
Rabbit antiserum	256	Positive	Yes
Mouse antiserum	192	Positive	Yes
Mouse antiserum	48	Positive	Yes
Goat antiserum	384	Positive	Yes
Recombinant protein immunized rabbit antiserum	6144	Positive	Yes

Supplementary Visit Plan. Essential steps and timing for each visit

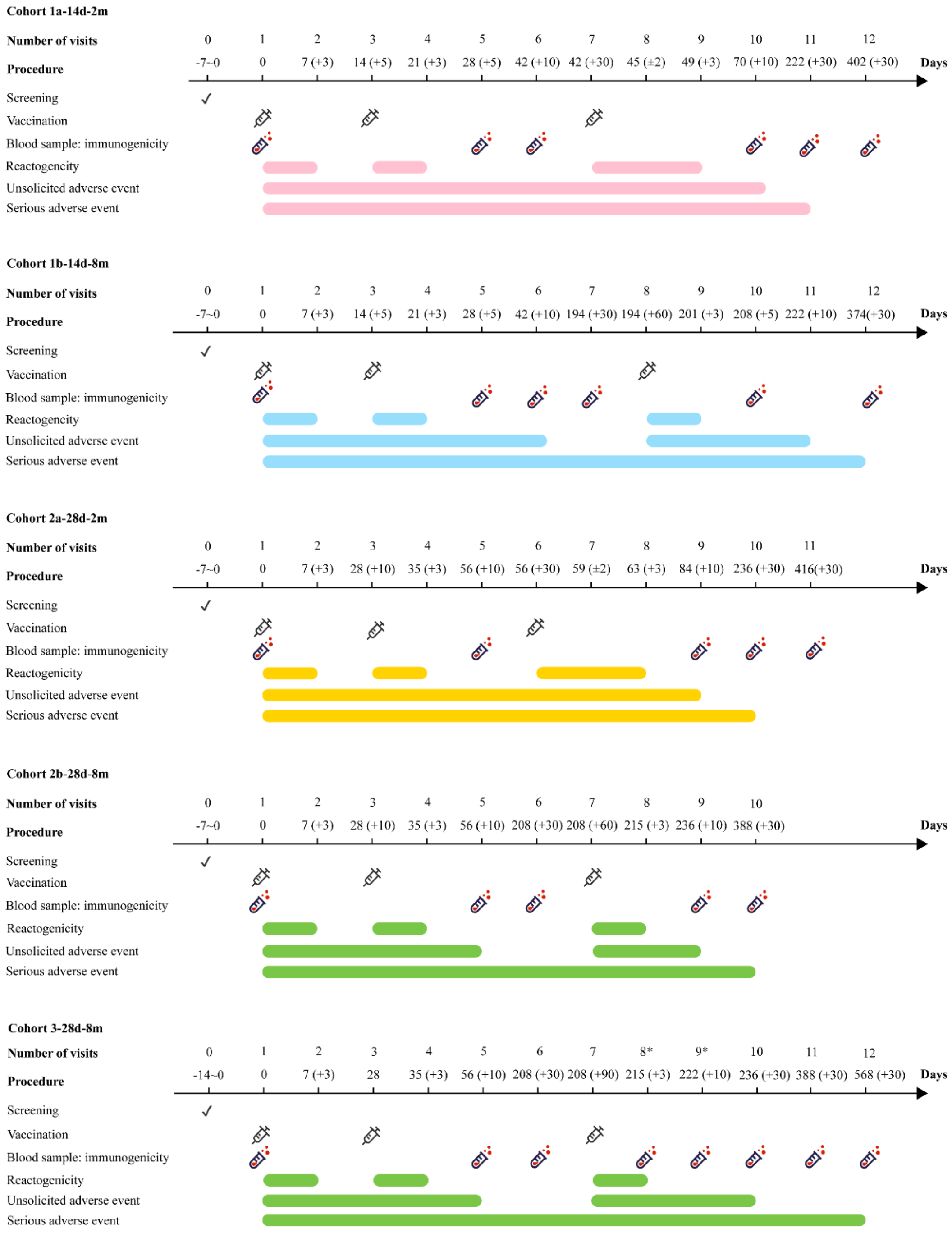

Supplementary Study endpoints

**Phase 2 trial among adults aged 18-59 years old**	
**Primary endpoints**	- Positive conversion rate of serum neutralizing antibody on Day 14 (***Cohort 1***)/Day 28 (***Cohort 2***) after two doses of test vaccine;<br>- Incidence of adverse reaction on Day 0-28 (Day 0-14 for the first dose for ***Cohort 1***) after each dose;
**Secondary endpoints**	- Positive rate, GMT and GMI of serum neutralizing antibody on Day 28 after two doses of test vaccine;<br>- Positive conversion rate, positive rate, GMT and GMI of serum neutralizing antibody on Day 28 after three doses of test vaccine (only for ***Cohort 1a-14d-2m*** and ***Cohort2a-28d-2m***);<br>- Incidence of adverse reactions 0-7 days after each dose of vaccination;<br>- Incidence of serious adverse event from the inoculation to 6 months after full course vaccination;
**Exploratory Endpoints**	- The seropositive rate and GMT of neutralizing antibody at 6 months after the second dose (only for ***Cohort 1b-14d-8m*** and ***Cohort 2b-28d-8m***);<br>- The seropositive rate and GMT of neutralizing antibody at 12 months after the third dose (only for ***Cohort 1a-14d-2m*** and ***Cohort 2a-28d-2m***);<br>- The seropositive rate and GMT of neutralizing antibody at 14 days (only for<br>***Cohort 1b-14d-8m***) or 28 days (only for ***Cohort 2b-28d-8m***) after the booster dose;<br>- The seropositive rate and GMT of neutralizing antibody at 6 months after the booster (only for ***Cohort 1b-14d-8m*** and ***Cohort 2b-28d-8m***).
**Phase 2 trial among adults aged 60 years and older**	
**Primary**	- Incidence of adverse reactions within 28 days after each dose of

**Endpoints**	vaccination;<br>- The seroconversion rate of neutralizing antibodies 28 days after the second dose vaccination.
**Secondary Endpoints**	- Incidence of adverse reactions within 7 days after each dose vaccination;<br>- Incidence of SAEs from the beginning of the vaccination to 12 months after booster immunization;<br>- The seropositive rate, GMT, and GMI of neutralizing antibodies 28 days after the second dose vaccination;
**Exploratory Endpoints**	- The seropositive rate and GMT 6 months after the second dose vaccination;<br>- The seropositive rate, GMT, and GMI 28 days after the booster vaccination;<br>- The seropositive rate and GMT 6 months after the booster vaccination;<br>- The seropositive rate and GMT 12 months after the booster vaccination.

Supplementary Figure 1. Trial profile

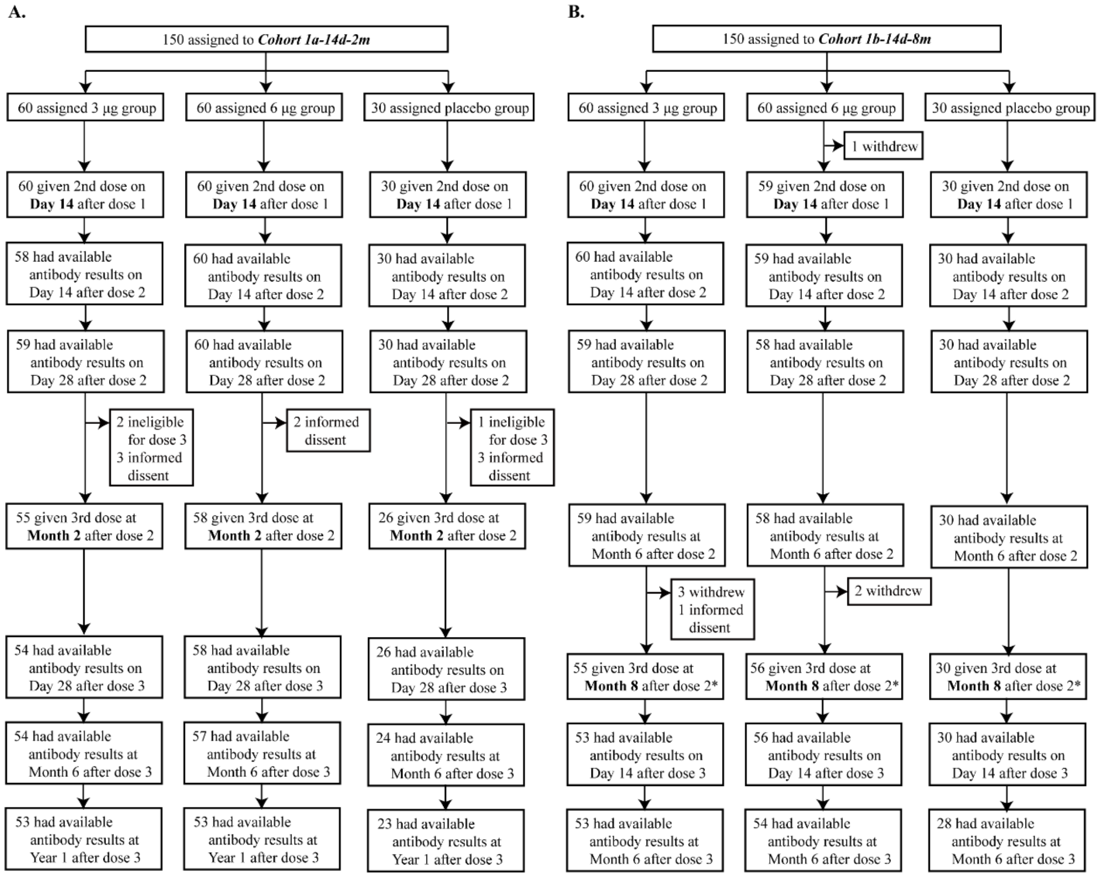

*: 141 participants were given third doses 9-11 days outside of the prespecified time window. The antibody results of these participants were included in the immunogenicity analysis.

C.

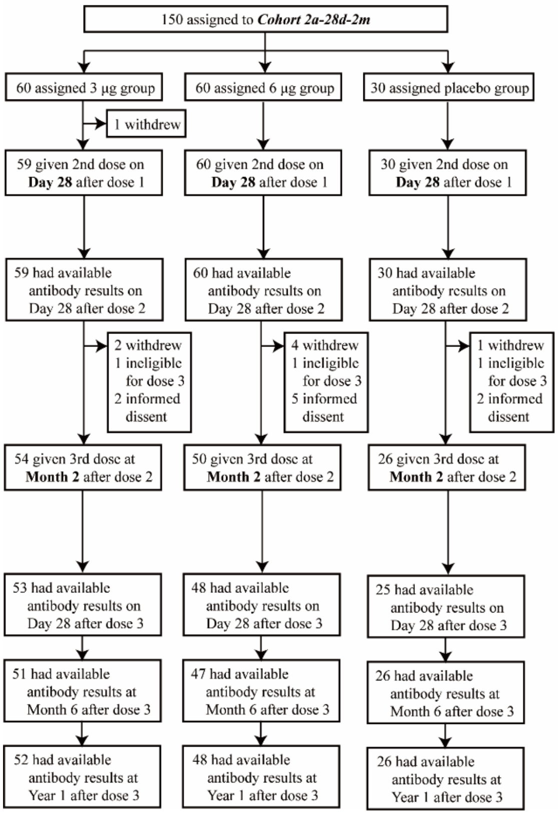

150 assigned to Cohort 2a-28d-2m
60 assigned 3 μg group
60 assigned 6 μg group
30 assigned placebo group
1 withdrew
59 given 2nd dose on Day 28 after dose 1
60 given 2nd dose on Day 28 after dose 1
30 given 2nd dose on Day 28 after dose 1
59 had available antibody results on Day 28 after dose 2
60 had available antibody results on Day 28 after dose 2
30 had available antibody results on Day 28 after dose 2
2 withdrew 1 ineligible for dose 3 2 informed dissent
4 withdrew 1 ineligible for dose 3 5 informed dissent
1 withdrew 1 ineligible for dose 3 2 informed dissent
54 given 3rd dose at Month 2 after dose 2
50 given 3rd dose at Month 2 after dose 2
26 given 3rd dose at Month 2 after dose 2
53 had available antibody results on Day 28 after dose 3
48 had available antibody results on Day 28 after dose 3
25 had available antibody results on Day 28 after dose 3
51 had available antibody results at Month 6 after dose 3
47 had available antibody results at Month 6 after dose 3
26 had available antibody results at Month 6 after dose 3
52 had available antibody results at Year 1 after dose 3
48 had available antibody results at Year 1 after dose 3
26 had available antibody results at Year 1 after dose 3

D.

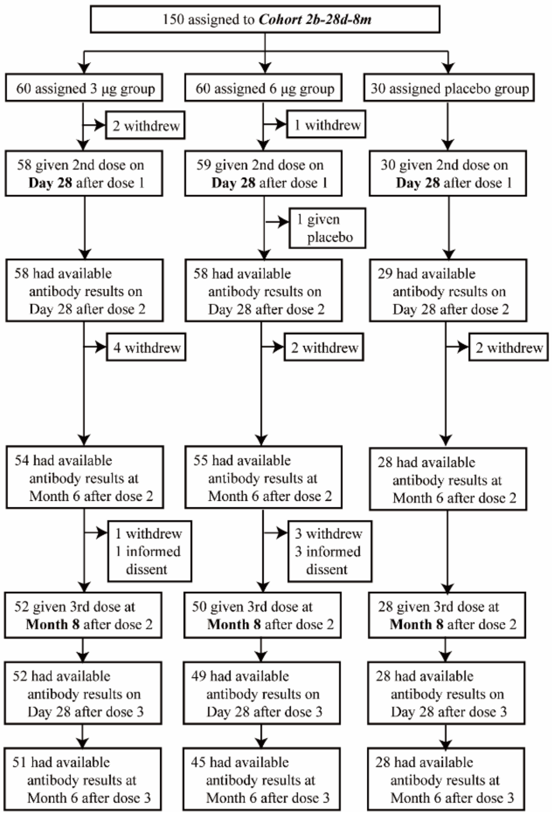

150 assigned to Cohort 2b-28d-8m
60 assigned 3 μg group
60 assigned 6 μg group
30 assigned placebo group
2 withdrew
1 withdrew
58 given 2nd dose on Day 28 after dose 1
59 given 2nd dose on Day 28 after dose 1
30 given 2nd dose on Day 28 after dose 1
1 given placebo
58 had available antibody results on Day 28 after dose 2
58 had available antibody results on Day 28 after dose 2
29 had available antibody results on Day 28 after dose 2
4 withdrew
2 withdrew
2 withdrew
54 had available antibody results at Month 6 after dose 2
55 had available antibody results at Month 6 after dose 2
28 had available antibody results at Month 6 after dose 2
1 withdrew 1 informed dissent
3 withdrew 3 informed dissent
52 given 3rd dose at Month 8 after dose 2
50 given 3rd dose at Month 8 after dose 2
28 given 3rd dose at Month 8 after dose 2
52 had available antibody results on Day 28 after dose 3
49 had available antibody results on Day 28 after dose 3
28 had available antibody results on Day 28 after dose 3
51 had available antibody results at Month 6 after dose 3
45 had available antibody results at Month 6 after dose 3
28 had available antibody results at Month 6 after dose 3

E

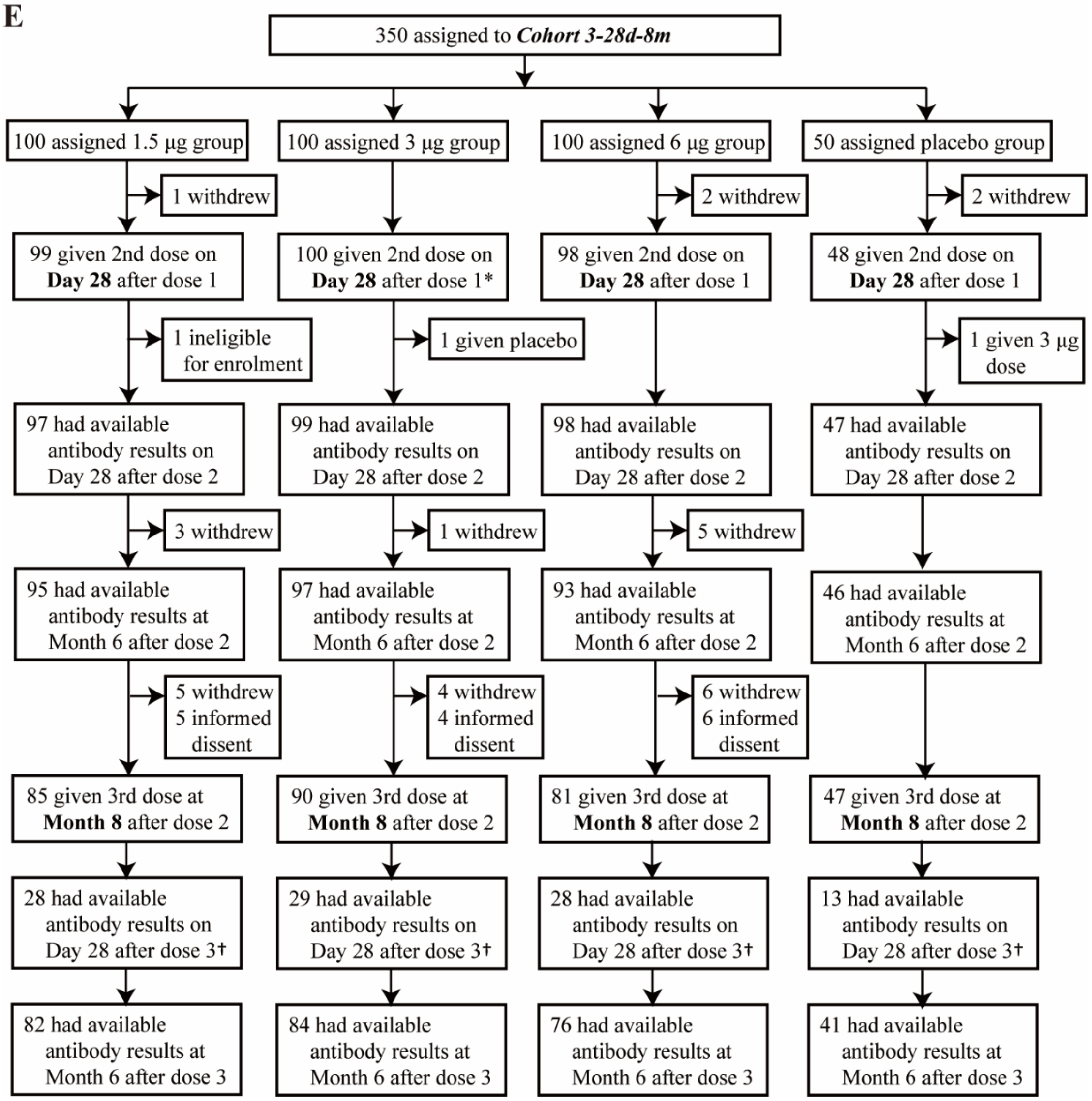

*: One participant received the second dose 5 days outside of the specified time window. The antibody results of this participant were included in the immunogenicity analysis.

†: Retest results were included in the analysis. Details were reported in Zeng G et al. *The Lancet Infect Dis* 2021. https://doi.org/10.1016/ S1473-3099(21)00681-2.

Supplementary Figure 2. Level of neutralising antibodies to infectious SARS-CoV-2 in adults aged 18-59 years

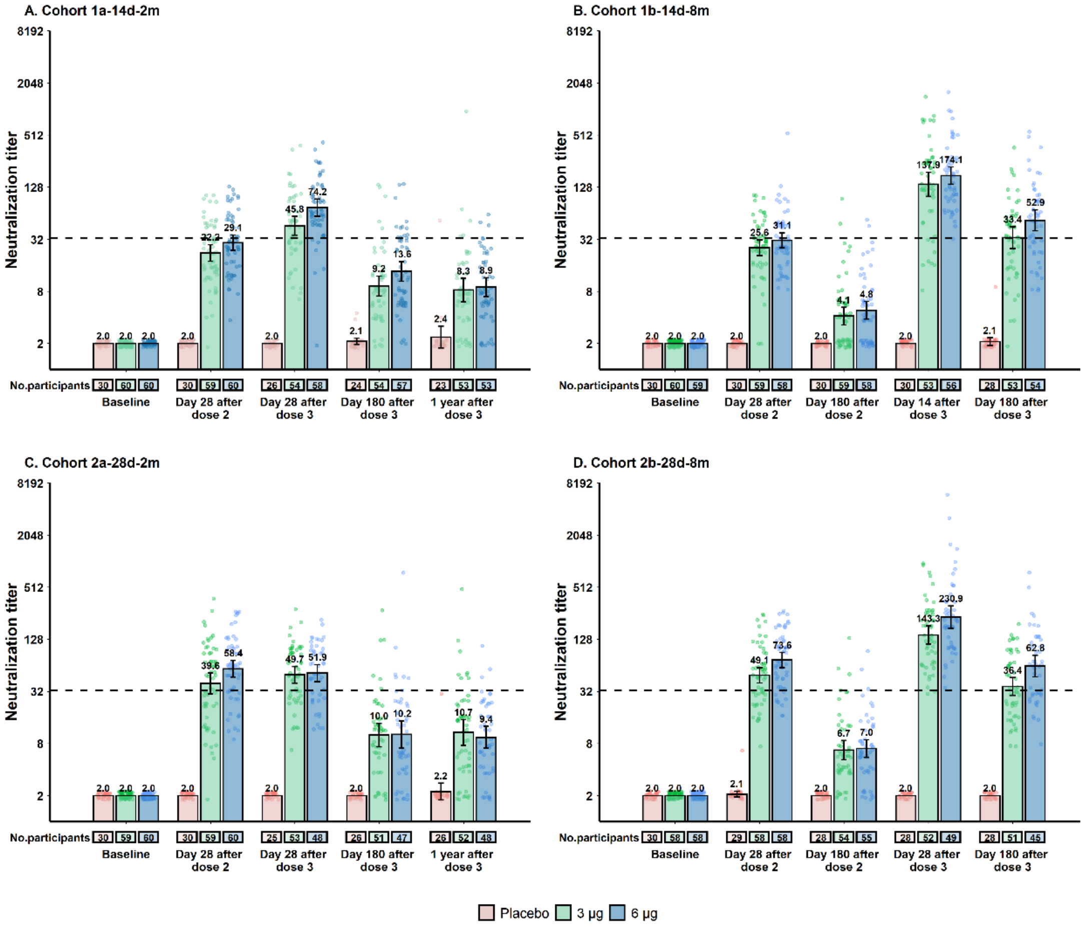

The number of participants for each group (placebo group, pink; 3 µg group, green; 6 µg group, blue) at each visit included in the analysis is provided below the bars. Dots are reciprocal neutralising antibody titres for individuals in the per-protocol population. Numbers above the bars are geometric mean titres (GMTs), and error bars indicate 95% CIs. GMTs and corresponding 95% CIs were calculated on the basis of standard normal distributions of log-transformed antibody titres. Titres lower than the limit of detection (1:4) are presented as half the limit of detection. The dotted horizontal line represents the protective threshold (1:33). Individual participant data of the graph are provided as a Source Data File.

Supplementary Figure 3. Decline in neutralizing antibodies to ancestral SARS-CoV-2 in adults aged 18-59 years

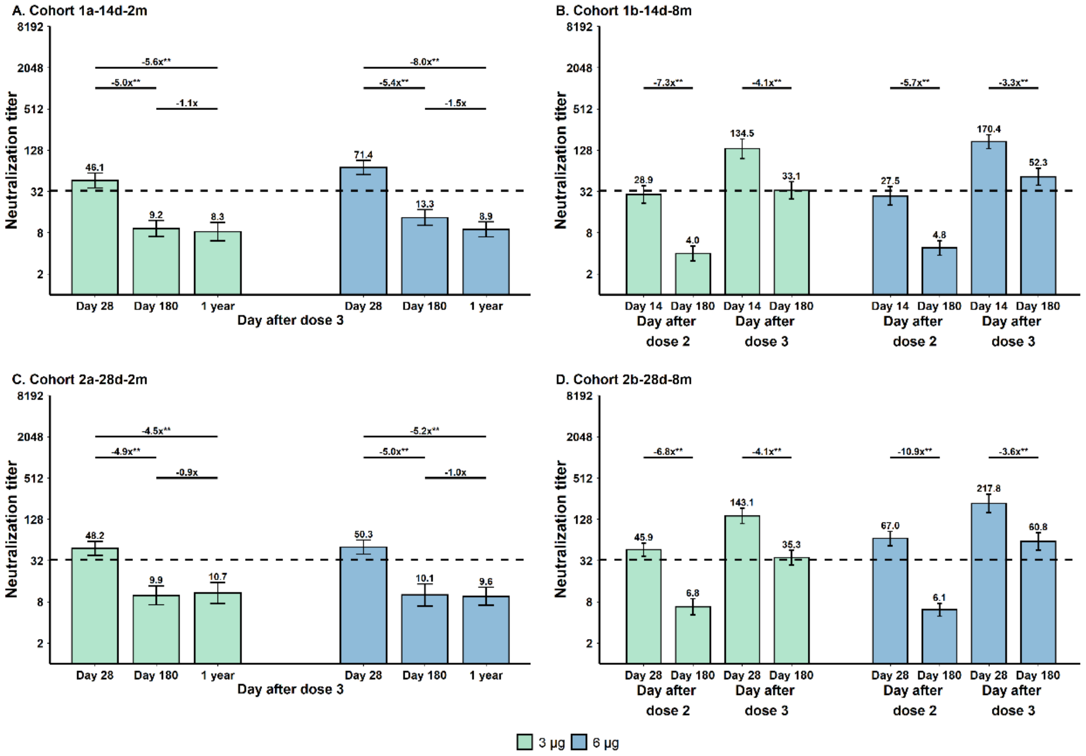

The number of participants for 3 μg group (green) and 6 μg group (blue) was A) 53 and 53, B) 52 and 53, C) 50 and 46, D) 49 and 43, respectively. Numbers above the bars are geometric mean titres (GMTs), and error bars indicate the 95% CIs. The dotted horizontal line represents the protective threshold (1:33). Numbers above the short horizontal lines are pairwise fold-change values. Individual participant data of the trial in adults aged between 18-59 years old are provided as a Source Data File. GMTs and corresponding 95% CIs were calculated on the basis of standard normal distributions of log-transformed antibody titres. GMT fold-decreases in neutralization titre were calculated as ratios of paired sera at two visits. Comparisons between groups were conducted by group t-tests with log-transformation (two-sided). * Represents p values for pairwise comparisons <0.05. ** Represents p values of pairwise comparisons <0.01.

Supplementary Figure 4. Decline in neutralizing antibodies to ancestral SARS-CoV-2 in adults aged 60 years and older

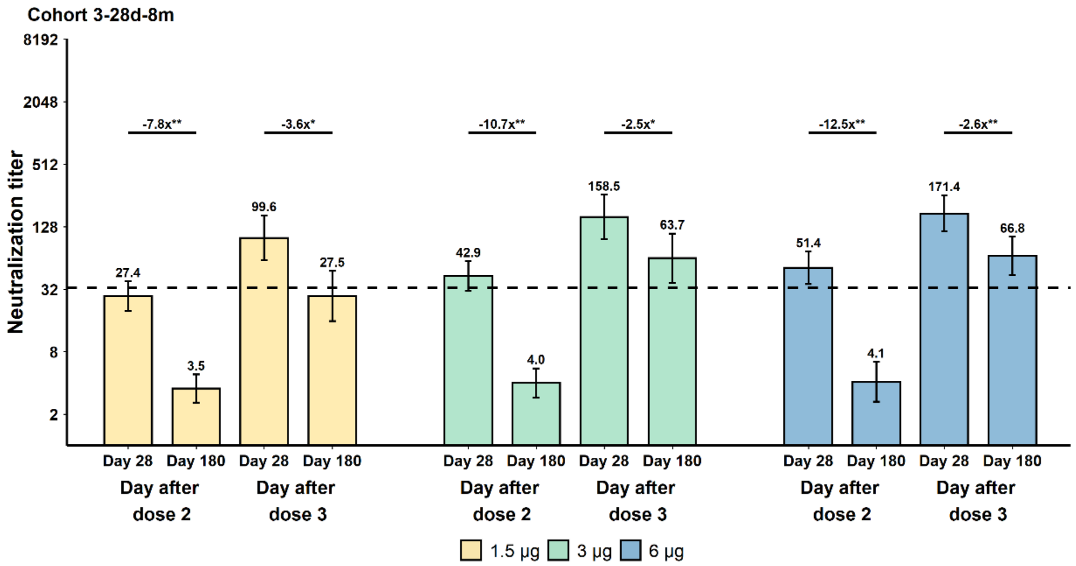

The number of participants for 1.5 μg (yellow), 3 μg (green) and 6 μg (blue) group was 28, 29 and 26, respectively. Numbers above the bars are geometric mean titres (GMTs), and error bars indicate the 95% CIs. The dotted horizontal line represents the protective threshold (1:33). Numbers above the short horizontal lines are pairwise fold-change values. Individual participant data of the trial in adults aged between 18-59 years old are provided as a Source Data File. GMTs and corresponding 95% CIs were calculated on the basis of standard normal distributions of log-transformed antibody titres. GMT fold-decreases in neutralization titre were calculated as ratios of paired sera at two visits. Comparisons between groups were conducted by group t-tests with log-transformation (two-sided). * Represents p values for pairwise comparisons <0.05. ** Represents p values of pairwise comparisons <0.01.

Supplementary Table 1. Baseline demographic characteristics in the safety population received the third dose

	1.5 μg group	3 μg group	6 μg group	Placebo group
**Cohort 1a-14d-2m**				
No. of Participants	—	55	58	26
Age, years (mean±SD)	—	45.2±9.1	44.7±8.6	44.3±8.6
Sex (male, %)	—	29 (53)	20 (35)	10 (39)
**Cohort 1b-14d-8m**				
No. of Participants	—	55	56	30
Age, years (mean±SD)	—	40.4±10.3	42.4±8.8	44.8±6.9
Sex (male, %)	—	24 (44)	27 (48)	12 (40)
**Cohort 2a-28d-2m**				
No. of Participants	—	54	50	26
Age, years (mean±SD)	—	42.5±8.6	40.7±9.4	44.0±7.7
Sex (male, %)	—	34 (63)	26 (52)	14 (54)
**Cohort 2b-28d-8m**				
No. of Participants	—	52	50	28
Age, years (mean±SD)	—	44.3±9.5	43.1±9.9	45.7±9.7
Sex (male, %)	—	23 (44)	26 (52)	11 (39)
**Cohort 3-28d-8m**				
No. of Participants	85	90	81	47
Age, years (mean±SD)	66.3±4.4	66.4±4.4	66.3±4.4	67.1±4.7
Sex (male, %)	41 (48)	44 (49)	37 (46)	27 (57)

Supplementary Table 2. Results of immunological endpoints in adults aged 18-59 years old

Indicator	Placebo group	3 μg group	6 μg group	P value (All groups)	P value (Placebo vs 3 μg)	P value (Placebo vs 6 μg)	P value (3 μg vs 6 μg)
**Cohort 1a-14d-2m**							
**Baseline (Pre-immunization)**							
GMT (95%CI)	2.0 (2.0-2.0)	2.0 (2.0-2.0)	2.0 (2.0-2.0)	—	—	—	—
**Day 14 after dose 2**							
GMT (95%CI)	2.2 (2.0-2.5)	27.0 (20.6-35.4)	40.8 (31.7-52.4)	<0.0001	<0.0001	<0.0001	0.0274
**Day 28 after dose 2**							
GMT (95%CI)	2.0 (2.0-2.0)	22.2 (17.8-27.8)	29.1 (23.7-35.8)	<0.0001	<0.0001	<0.0001	0.0764
**Day 28 after dose 3**							
GMT (95%CI)	2.0 (2.0-2.0)	45.8 (35.7-58.9)	74.2 (59.0-93.3)	<0.0001	<0.0001	<0.0001	0.0053
**Day 180 after dose 3**							
GMT (95%CI)	2.1 (2.0-2.3)	9.2 (7.1-12.0)	13.6 (10.5-17.7)	<0.0001	<0.0001	<0.0001	0.0393
**1 year after dose 3**							
GMT (95%CI)	2.4 (1.8-3.2)	8.3 (6.1-11.3)	8.9 (7.0-11.5)	<0.0001	<0.0001	<0.0001	0.7047
**Cohort 1b-14d-8m**							
**Baseline (Pre-immunization)**							
GMT (95%CI)	2.0 (2.0-2.0)	2.0 (2.0-2.0)	2.0 (2.0-2.0)	—	—	—	—
**Day 14 after dose 2**							
GMT (95%CI)	2.3 (1.9-2.8)	28.2 (21.1-37.6)	29.2 (21.8-39.0)	<0.0001	<0.0001	<0.0001	0.8647
**Day 28 after dose 2**							
GMT (95%CI)	2.0 (2.0-2.0)	25.6 (20.9-31.4)	31.1 (25.4-38.0)	<0.0001	<0.0001	<0.0001	0.1770
**Day 180 after dose 2**							
GMT (95%CI)	2.0 (2.0-2.0)	4.1 (3.3-5.2)	4.8 (3.8-6.1)	<0.0001	<0.0001	<0.0001	0.3574
**Day 14 after dose 3**							
GMT (95%CI)	2.0 (2.0-2.0)	137.9 (99.9-190.4)	174.1 (138.5-218.9)	<0.0001	<0.0001	<0.0001	0.2395
**Day 180 after dose 3**							

GMT (95%CI)	2.1 (1.9-2.3)	33.4 (25.0-44.6)	52.9 (40.1-70.0)	<0.0001	<0.0001	<0.0001	0.0232
**Cohort 2a-28d-2m**							
**Baseline (Pre-immunization)**							
GMT (95%CI)	2.0 (2.0-2.0)	2.0 (2.0-2.0)	2.0 (2.0-2.0)	—	—	—	—
**Day 28 after dose 2**							
GMT (95%CI)	2.0 (2.0-2.0)	39.6 (30.1-52.2)	58.4 (46.9-72.7)	<0.0001	<0.0001	<0.0001	0.0296
**Day 28 after dose 3**							
GMT (95%CI)	2.0 (2.0-2.0)	49.7 (39.9-61.9)	51.9 (41.3-65.3)	<0.0001	<0.0001	<0.0001	0.7793
**Day 180 after dose 3**							
GMT (95%CI)	2.0 (2.0-2.0)	10.0 (7.3-13.7)	10.2 (7.1-14.6)	<0.0001	<0.0001	<0.0001	0.9486
**1 year after dose**							
GMT (95%CI)	2.2 (1.8-2.8)	10.7 (7.6-15.1)	9.4 (7.0-12.6)	<0.0001	<0.0001	<0.0001	0.5750
**Cohort 2b-28d-8m**							
**Baseline (Pre-immunization)**							
GMT (95%CI)	2.0 (2.0-2.0)	2.0 (2.0-2.0)	2.0 (2.0-2.0)	—	—	—	—
**Day 28 after dose 2**							
GMT (95%CI)	2.1 (1.9-2.2)	49.1 (40.1-60.2)	73.6 (60.2-90.0)	<0.0001	<0.0001	<0.0001	0.0054
**Day 180 after dose 2**							
GMT (95%CI)	2.0 (2.0-2.0)	6.7 (5.2-8.6)	7.0 (5.5-8.8)	<0.0001	<0.0001	<0.0001	0.8027
**Day 28 after dose 3**							
GMT (95%CI)	2.0 (2.0-2.0)	143.3 (112.3-182.8)	230.9 (171.2-311.5)	<0.0001	<0.0001	<0.0001	0.0147
**Day 180 after dose 3**							
GMT (95%CI)	2.0 (2.0-2.0)	36.4 (28.7-46.1)	62.8 (47.1-83.8)	<0.0001	<0.0001	<0.0001	0.0041

GMT (geometric mean titer) was calculated based on log-transformation data. ANOVA model with log-transformation (GMT) was used to detect the difference among groups (two-sided). Comparison between groups was conducted by group t-test with log-transformation (two-sided).

Supplementary Table 3. Results of immunological endpoints in adults aged 60 years and older

Indicator	placebo group	1.5 µg group	3 µg group	6 µg group	P value (All groups)	P value (1.5 µg vs 3 µg)	P value (1.5 µg vs 6 µg)	P value (3 µg vs 6 µg)
**Baseline (Pre-immunization)**								
GMT (95%CI)	2.0 (2.0-2.0)	2.0 (2.0-2.0)	2.0 (2.0-2.1)	2.1 (1.9-2.2)	—	—	—	—
**Day 28 after dose 2**								
GMT (95%CI)	2.1 (2.0-2.1)	23.4 (19.4-28.3)	41.2 (34.2-49.6)	49.9 (42.2-58.9)	<0.0001	<0.0001	<0.0001	0.1310
**Day 180 after dose 2**								
GMT (95%CI)	2.1 (1.9-2.2)	3.1 (2.7-3.6)	3.4 (2.9-4.1)	4.1 (3.3-5.0)	0.0002	0.3858	0.0426	0.2427
**Day 28 after dose 3**								
GMT (95%CI)	2.0 (2.0-2.0)	99.6 (60.6-163.5)	158.5 (96.9-259.2)	178.9 (123.2-259.9)	<0.0001	0.1779	0.0584	0.6893
**Day 180 after dose 3**								
GMT (95%CI)	2.0 (2.0-2.0)	20.6 (15.5-27.3)	53.2 (39.7-71.1)	91.2 (71.5-116.3)	<0.0001	<0.0001	<0.0001	0.0052

GMT (geometric mean titer) was calculated based on log-transformation data. ANOVA model with log-transformation was used to detect the difference among groups (two-sided). Comparison between groups was conducted by group t-test with log-transformation (two-sided).

Supplementary Table 4. Sensitivity analysis of l**ower dilution in adults aged 18-59 years old in cohort 2b-28d-8m**

**Visit**	**3 µg group**			**6 µg group**		
	**Main analysis**	**Sensitivity analysis**	**p-value**	**Main analysis**	**Sensitivity analysis**	**p-value**
Baseline	2.0 (2.0-2.0)	2.0 (2.0-2.0)	-	2.0 (2.0-2.0)	2.0 (2.0-2.0)	-
Day 28 after dose 2	49.1 (40.1-60.2)	42.1 (34.3-51.7)	0.29	73.6 (60.2-90.0)	58.9 (48.1-72.1)	0.12
Day 180 after dose 2	6.7 (5.2-8.6)	5.8 (4.5-7.4)	0.42	7.0 (5.5-8.8)	6.0 (4.8-7.5)	0.34
Day 28 after dose 3	143.3 (112.3-182.8)	119.7 (93.7-153.1)	0.30	230.9 (171.2-311.5)	177.2 (131.4-239.0)	0.21
Day 180 after dose 3	36.4 (28.7-46.1)	30.3 (23.8-38.6)	0.28	62.8 (47.1-83.8)	54.9 (41.2-73.1)	0.50

Supplementary Table 5. Sensitivity analysis of higher dilution in adults aged 18-59 years old in cohort 2b-28d-8m

Visit	3 μg group			6 μg group		
	Main analysis	Sensitivity analysis	p-value	Main analysis	Sensitivity analysis	p-value
Baseline	2.0 (2.0-2.0)	2.0 (2.0-2.0)	-	2.0 (2.0-2.0)	2.0 (2.0-2.0)	-
Day 28 after dose 2	49.1 (40.1-60.2)	54.8 (44.4-67.6)	0.46	73.6 (60.2-90.0)	86.3 (70.1-106.2)	0.27
Day 180 after dose 2	6.7 (5.2-8.6)	7.4 (5.7-9.7)	0.58	7.0 (5.5-8.8)	7.8 (6.1-10.0)	0.52
Day 28 after dose 3	143.3 (112.3-182.8)	162.7 (126.7-209.0)	0.47	230.9 (171.2-311.5)	278.7 (205.2-378.4)	0.38
Day 180 after dose 3	36.4 (28.7-46.1)	41.4 (32.6-52.7)	0.44	62.8 (47.1-83.8)	69.1 (51.5-92.9)	0.64

Supplementary Table 6. Sensitivity analysis of lower dilution in adults aged 60 years and older in cohort 3-28d-8m

Visit	1.5 μg group			3 μg group			6 μg group		
	Main analysis	Sensitivity analysis	p-value	Main analysis	Sensitivity analysis	p-value	Main analysis	Sensitivity analysis	p-value
Baseline	2.0 (2.0-2.0)	2.0 (2.0-2.0)	-	2.0 (2.0-2.1)	2.0 (2.0-2.0)	-	2.1 (1.9-2.2)	2.1 (1.9-2.2)	
Day 28 after dose 2	23.4 (19.4-28.3)	19.0 (15.7-23.0)	0.12	41.2 (34.2-49.6)	32.2 (26.8-38.8)	0.06	49.9 (42.2-58.9)	38.7 (32.7-45.9)	0.04
Day 180 after dose 2	3.1 (2.7-3.6)	2.9 (2.6-3.3)	0.55	3.4 (2.9-4.1)	3.3 (2.8-3.9)	0.74	4.1 (3.3-5.0)	3.7 (3.0-4.5)	0.51
Day 28 after dose 3	99.6 (60.6-163.5)	86.1 (52.0-142.6)	0.68	158.5 (96.9-259.2)	125.0 (76.1-205.2)	0.49	178.9 (123.2-259.9)	137.9 (96.1-197.8)	0.31
Day 180 after dose 3	20.6 (15.5-27.3)	17.4 (13.2-23.0)	0.40	53.2 (39.7-71.1)	42.4 (31.8-56.4)	0.27	91.2 (71.5-116.3)	74.1 (57.9-94.7)	0.23

Supplementary Table 7. Sensitivity analysis of higher dilution in adults aged 60 years and older in cohort 3-28d-8m

Visit	1.5 μg group			3 μg group			6 μg group		
	Main analysis	Sensitivity analysis	p-value	Main analysis	Sensitivity analysis	p-value	Main analysis	Sensitivity analysis	p-value
Baseline	2.0 (2.0-2.0)	2.0 (2.0-2.0)	-	2.0 (2.0-2.1)	2.0 (2.0-2.1)	-	2.1 (1.9-2.2)	2.1 (1.9-2.2)	-
Day 28 after dose 2	23.4 (19.4-28.3)	27.2 (22.4-32.9)	0.28	41.2 (34.2-49.6)	49.0 (40.5-59.4)	0.20	49.9 (42.2-58.9)	59.6 (50.4-70.6)	0.13
Day 180 after dose 2	3.1 (2.7-3.6)	3.2 (2.7-3.8)	0.70	3.4 (2.9-4.1)	3.5 (2.9-4.3)	0.82	4.1 (3.3-5.0)	4.3 (3.5-5.5)	0.67
Day 28 after dose 3	99.6 (60.6-163.5)	110.3 (67.1-181.6)	0.77	158.5 (96.9-259.2)	187.6 (114.2-308.2)	0.62	178.9 (123.2-259.9)	215.3 (145.5-318.4)	0.49
Day 180 after dose 3	20.6 (15.5-27.3)	23.2 (17.4-31.0)	0.56	53.2 (39.7-71.1)	62.4 (46.4-84.1)	0.44	91.2 (71.5-116.3)	105.7 (82.6-135.2)	0.40

Supplementary Table 8. Serious adverse events by system organ class and preferred term reported during 6-month follow-up after administration of booster dose among adults aged 18-59 years old (cohort 2b-28d-8m)

Adverse events (MedDRA 23.0)	3 µg group (N=52)	6 µg group (N=50)	Placebo (N=28)	Total (N=130)
**No of participants**	1 (2%)	0	0	1 (1%)
**No of events**	1	0	0	1
**Neoplasms benign, malignant and unspecified (incl cysts and polyps)**				
Breast cancer	1 (2%)	0	0	1 (1%)

Supplementary Table 9. Serious adverse events by system organ class and preferred term reported during 6-month follow-up after administration of third dose among adults aged 60 years and older (cohort 3-28d-8m)

Adverse Events (MedDRA 23.0)	1.5 µg group (N=85)	3 µg group (N=90)	6 µg group (N=81)	Placebo group (N=47)	Total (N=303)
**No of participants**	4 (5%)	5 (6%)	3 (4%)	2 (4%)	14 (5%)
**No of events**	5	5	4	3	17
**Nervous system disorders**					
Cerebral infarction	0	2 (2%)	1 (1%)	1 (2%)	4 (1%)
Transient ischemic attack	1 (1%)	1 (1%)	0	0	2 (1%)
**Neoplasms benign, malignant and unspecified (incl cysts and polyps)**					
Lung neoplasm	1 (1%)	0	0	0	1 (0%)
Breast cancer	1 (1%)	0	1 (1%)	0	2 (1%)
**Cardiac disorders**					
Unstable angina pectoris	1 (1%)	0	0	1 (2%)	2 (1%)
Coronary artery disease	1 (1%)	0	0	1 (2%)	2 (1%)
Atrial flutter	0	0	1 (1%)	0	1 (0%)
**Musculoskeletal and connective tissue disorders**					
Osteoarthritis	0	1 (1%)	0	0	1 (0%)
**Vascular and lymphatic diseases**					
Varicosity	0	1 (1%)	0	0	1 (0%)
**Injury, poisoning and operative complications**					
Craniocerebral injury	0	0	1 (1%)	0	1 (0%)

Project Title: Phase I/II Clinical Trial for COVID-19 Vaccine (Vero Cell), Inactivated

Protocol Title: A Randomized, Double-Blinded, Placebo-Controlled, Phase I/II Clinical Trial, to Evaluate the Safety and Immunogenicity of the COVID-19 Vaccine (Vero Cell), Inactivated in Healthy Adults Aged 18~59 Years

Product Name: COVID-19 Vaccine (Vero Cell), Inactivated

Sponsor: Sinovac Research & Development Co., Ltd.

Research Organization: Jiangsu Provincial Center for Disease Control and Prevention (Public Health Research Institute of Jiangsu Province)

Statistical Organization: Beijing KEY TECH Statistical Technology Co., Ltd.

Protocol No.: PRO-nCOV-1001

Protocol version/date: December 25, 2020

Version No.: 1.5

Protocol approver: Gao Qiang

Signature of approver:

Approval date: MM/DD/YY

北京科兴中维生物技术有限公司
SINOVAC RESEARCH & DEVELOPMENT CO., LTD.

Signature of Principal Investigator

I hereby agree to:

- Assume the responsibility for properly instructing the Clinical Research in this region.
- Ensure that the Research is carried out in accordance with the Trial Protocol and standard operating procedure for clinical research.
- Ensure that the personnel involved in the Project have a full knowledge of the research product information, as well as other responsibilities and obligations in connection with the Research as specified in the Trial Protocol.
- Ensure that no change will be made to the Trial Protocol without review and written approval of the Sponsor and the Independent Ethics Committee (IEC), unless it is necessary to eliminate the immediate hazard to the subjects or as required by the registration authority (for example: in terms of administration of the Project).
- I am thoroughly familiar with the methods for properly using the Vaccine as described in the Trial Protocol and have a full knowledge of other information provided by the Sponsor, including but not limited to the following: the current Investigator's Brochure (IB) or equivalent document and Supplementary Documents to the Investigator's Brochure (if any).

- I am familiar with and will comply with the *Good Clinical Practice* (GCP), *Guidelines for Quality Management of Vaccine Clinical Trial (Trial)* and all prevailing regulatory requirements.

Name of Principal Investigator: Zhu Fengcai

Signature:

Date: MM/DD/YY

Research Team

Sponsor

Organization name: Sinovac Research & Development Co., Ltd.

Add: Peking University Biopolis, No. 39, Shangdi West Road, Haidian District, Beijing

Contact name: Gao Qiang

Mobile: 13693092396 Fax: 010-62979669 Postcode: 100085

E-mail: gaoq@sinovac.com

Organization Responsible for the Clinical Trial

Organization name: Jiangsu Provincial Center for Disease Control and Prevention (Public Health Research Institute of Jiangsu Province)

Add: No. 172, Jiangsu Road, Nanjing, Jiangsu

Specialized department: Institute for Clinical Evaluation of Vaccines

Name of person in charge: Pan Hongxing

Mobile: 18118996996 Fax: 02583759529 Postcode: 210009

Email: panhongxing@126.com

Principal Investigator (PI)

Name: Zhu Fengcai

Organization: Jiangsu Provincial Center for Disease Control and Prevention (Public Health Research Institute of Jiangsu Province)

Mobile: 13951994867 Fax: 02583759529 Postcode: 210009

Email: jszfc@vip.sina.com

Principal Investigator (CO-PI)

Name: Pan Hongxing

Organization: Jiangsu Provincial Center for Disease Control and Prevention (Public Health Research Institute of Jiangsu Province)

Mobile: 18118996996 Fax: 02583759529 Postcode: 210009

E-mail: panhongxing@126.com

Site Organization of the Clinical Trial

Organization name: Center for Disease Control and Prevention of Suining County

Add: North Side of Yongchang Road and West Side of Suihe North Road, Suining County

Contact name: Jiang Congbing

Mobile: 18751728180 Fax: 0516-80377828 Postcode: 221200

E-mail: snjkws@126.com

Organization Responsible for Monitoring

Name: Hu Yuansheng

Organization: Sinovac Biotech Co., Ltd.

Add: Peking University Biopolis, No. 39, Shangdi West Road, Haidian District, Beijing

Mobile: 13436950182,010-82799318 Fax: 010-82890408 Postcode: 100085

E-mail: huys@sinovac.com

Organization for IgG and IgM Screening and Nucleic Acid Test

Organization name: Center for Disease Control and Prevention of Suining County

Add: North Side of Yongchang Road and West Side of Suihe North Road, Suining County

Contact name: Jiang Congbing

Mobile: 18751728180 Fax: 0516-80377828 Postcode: 221200

E-mail: 413659915@qq.com

Organization for Blood Routine Examination/Blood Biochemistry/Routine Urine Test

Organization name: Traditional Chinese Medicine Hospital of Suining County

Add: No. 75, Bayi East Road, Suicheng Town, Suining County, Xuzhou, Jiangsu

Contact name: Fan Ke

Mobile: 13375115098 Postcode: 221200 E-mail: snxzyy@163.com

Organization for Serum Antibody Detection

Organization name: National Institutes for Food and Drug Control

Add: No. 31, Huatuo Road, Daxing District, Beijing

Mobile: 010-53851770 Postcode: 102629

Organization for T Cell Response/Serum Inflammatory Factor Detection

Organization name: Jiangsu Huatai Vaccine Engineering Technology Research Co., Ltd.

Add: Vaccine Engineering Center of China Medical City, No. 1, Yaocheng Avenue, Taizhou, Jiangsu

Contact name: Wang Yongzhi

Mobile: 15952610030 Postcode: 225300 E-mail: sxwyz@126.com

Organization for Data Management

Organization name: Meida Kelin (Nanjing) Medicine Technology Co., Ltd.

Add: Room A, 20F, Oriental International Technology and Science Building, No.58 Xiangcheng Road, Pudong District, Shanghai

Contact name: Sun Hualong

Mobile: 13816706496 Postcode: 200031 E-mail: hualong.sun@meta-clinical .com

Organization for Statistical Analysis

Organization name: Beijing KEY TECH Statistical Technology Co., Ltd.

Add: 1018-1119w, Sihui Building, Huihe South Street, Chaoyang District, Beijing

Contact name: Jiang Zhiwei

Mobile: 18618483152 Postcode: 100025 E-mail: zhi.wei.jiang@ktstat.com

Revision History of the Protocol

S/N	Original version No./version date/revision part	Current version No./version date/revision description
1	Version 1.4 /June 30, 2020/4. Preclinical Research and Laboratory Evaluation of Vaccine	In Version 1.5/December 25, 2020, reproductive development toxicity test on rats was updated.
2	Version 1.4 / June 30, 2020 /5. Preliminary Clinical Trial	In Version 1.5/December 25, 2020, immunization persistence results after two doses of immunization and immunogenicity results after three doses of immunization were added.
3	Version 1.4 / June 30, 2020 / 6.2 Vaccine stability	In Version 1.5/December 25, 2020, stability research findings were supplemented.
4	Version 1.4/June 30, 2020/8.2.1 Phase I test endpoint	In Version 1.5/December 25, 2020, "Positive rate of IgG and IgM antibodies 6 months after the full-course vaccination of test vaccine", "positive rate of antinuclear antibody on Day 194 after the first dose of test vaccine" and "positive rate of antinuclear antibody on Day 208 after the first dose of test vaccine" were added in exploratory endpoint.
5	Version 1.4 / June 30, 2020 / 8.2.2 Phase II test endpoint	In Version 1.5/December 25, 2020, relevant test endpoint for booster immunization 6 months after Day 0, Day 14 and Day 0, Day 28 schedule was added.
6	Version 1.4 / June 30, 2020 / 8.3.2 Phase II research plan & 10.1 Visit plan & 10.6 Sample collection plan	6-month booster immunization after the second dose of vaccination was added into day 0, 14 and day 0, 28 primary immunization schedule in Version 1.5 / December 25, 2020/research plan. Besides, the corresponding visit and sample collection were added in the visit plan.
7	Version 1.4 / June 30, 2020 / 10.7.7 Report on serious adverse events (SAE)	In Version 1.5/December 25, 2020, the reporting requirements for serious adverse events were revised according to 2020 New GCP.
8	Version 1.4/June 30, 2020 /10.11.1 Analysis set	In Version 1.5/December 25, 2020, the division of data analysis set was revised according to the revision of this

S/N	Original version No./version date/revision part	Current version No./version date/revision description
		immunization schedule.
9	After version 1.5 is approved, version 1.4 is null and void	
10	Version 1.3/June 25, 2020	In Version 1.4/June 30, 2020, the enrollment method for the third dose was changed to enrollment of 30 subjects under Day 0, Day 14, Day 42 schedule, and another 30 subjects were enrolled under Day 0, Day 28, Day 56 schedule; the third dose was given after it was confirmed that the above subjects were safe.
11	Version 1.3/June 25, 2020	In Version 1.4 / June 30, 2020, "blood collection on D3 before and after the third dose of immunization" was added for 60 subjects numbered C001 ~ C030 and D001 ~ D030.
12	After version 1.4 is approved, version 1.3 is null and void	
13	Version 1.2/April 14, 2020	In Version 1.3/June 25, 2020, "5. Preliminary Clinical Trial" was added.
14	Version 1.2/April 14, 2020	In Version 1.3/June 25, 2020, immunization schedule for some subjects vaccinated with the third dose was added in phase II clinical trial.
15	Version 1.2/April 14, 2020	In Version 1.3/June 25, 2020, the observation time of 3-dose immunization schedule was extended from 6 months to 12 months during phase II clinical trial.
16	After Version 1.3 is approved, Version 1.2 is null and void	
17	Version 1.1/ April 14, 2020/8.6 Test suspension and termination criteria	In Version 1.2/April 14, 2020, "After each dose of vaccination, statistics were given on adverse reaction of the subjects, and the test was suspended or terminated according to the following criteria" was added in "8.6 Test suspension and termination criteria" This criterion is determined as the suspension and termination criteria for vaccination groups.
18	Version 1.1/April 14, 2020/9.2 Subject exclusion criteria	In Version 1.2/April 14, 2020, "(Only for Phase I)" was deleted from "(7) IgG or IgM screening result was positive (only for Phase I);" "(only for Phase I)" was deleted from "(7) RT-PCR result of throat

S/N	Original version No./version date/revision part	Current version No./version date/revision description
		or anal swabs was positive (only for Phase I)”
19	Version 1.1/ April 14, 2020/Table 9, Tables 13-16, Tables 19-22	In Version 1.2/April 14, 2020, “Anti-nuclear antibody test” was added in Table 9; “anti-nuclear antibody test, IgG and IgM screening, RT-PCR test of throat and anal swabs” was added in “Tables 13-16”; the collection time of informed consent and demographic information was changed into visit 0; “anti-nuclear antibody” was added in Tables 19-22
20	After Version 1.2 is approved, Version 1.1 is null and void	
21	Version 1.0/April 11, 2020<br>2.6 Organization for the Trial Sample Test	In Version 1.1/April 14, 2020, antinuclear antibody (ANA) test was added.
22	Version 1.0/April 11, 2020<br>8.2.1 Endpoints of phase I trial	In Version 1.1/April 14, 2020/exploratory endpoint, “Positive rate of antinuclear antibody on Day 7/14/21/28/42 after the first dose of test vaccine (emergency immunization schedule); positive rate of antinuclear antibody on Day 28/35/42/56 after the first dose of test vaccine (routine immunization schedule)” was added.
23	Version 1.0/April 11, 2020<br>9.2 Exclusion Criteria for Subjects	In Version 1.1/April 14, 2020, “with SARS record in self-report” was added in exclusion criteria.
24	Version 1.0/April 11, 2020<br>9.2 Exclusion Criteria for Subjects	In Version 1.1/April 14, 2020, “Platelet count (PLT)” was added in routine blood indexes for laboratory screening.
25	Version 1.0/April 11, 2020<br>10.1 Visit plan	In Version/April 14, 2020, the time of visit 0 in Tables 11 and 12 was changed from “D-14~0” into “D-7~0”.
26	Version 1.0/April 11, 2020<br>10.5 Safety Follow-up and Observation	In Version 1.1/April 14, 2020, “The investigator paid a visit and collected safety data on Day 0-7” was changed into “the investigator paid a visit (not less than 2 face-to-face visits in Phase I)”.
27	Version 1.0/April 11, 2020<br>10.6 Sample collection	In Version 1.1/April 14, 2020, “Serum shall be separated from venous blood samples for serum antibody (neutralizing antibody/IgG/IgM) test and serum inflammatory factor + antinuclear

S/N	Original version No./version date/revision part	Current version No./version date/revision description
		antibody test in time, placed into 2 tubes (no less than 1ml for single serum tube A in phase I and no less than 0.5ml in phase II, and tube B for backup serum), and recorded" was changed into "Serum shall be separated from blood samples for serum antibody (neutralizing antibody/IgG/IgM) test, placed into 2 tubes (no less than 1ml for serum tube A in phase I and no less than 0.5ml in phase II, and tube B for backup serum), and recorded".
28	Version 1.0/April 11, 2020<br>10.7.1 Safety Observation Indexes	In Version 1.1/April 14, 2020, "Platelet count (PLT)" was added in routine blood indexes.
29	Version 1.0/April 11, 2020<br>10.7.6 Handling of Adverse Events	In Version 1.1/April 14, 2020/test observation, subjects who developed fever, with cough and other respiratory symptoms should be seen immediately at the designated hospitals, throat swabs/sputum and anal swabs should be collected if necessary. Besides, imaging examinations such as CT should be performed to analyze and determine if the disease was caused by COVID-19 infection. In case of novel coronavirus infection, it shall be treated according to SAE, and the presence of ADE phenomenon was especially analyzed.

List of Abbreviations of the Protocol

**PROTOCOL TITLE**	A Randomized, Double-Blinded, Placebo-Controlled, Phase I/II Clinical Trial, to Evaluate the Safety and Immunogenicity of the SARS-CoV-2 Inactivated Vaccine in Healthy Adults Aged 18~59 Years
**SPONSOR**	Sinovac Research & Development Co., Ltd.
**PROJECT PHASE**	Phase I/II
**OBJECTIVE(S)**	To evaluate the safety and immunogenicity of SARS-CoV-2 vaccine
**EXPERIMENTAL DESIGN OF THE TRIAL**	A randomized, Double-blinded, Placebo-Controlled, Phase I/II Clinical Trial
**PLANNED SAMPLE SIZE**	Total of 744 subjects， with 144 in the phase I and 600 in the phase II clinical trial
**SUBJECT SELECTION CRITERIA**	Healthy adults aged 18-59 years, with equal percentage of each gender
**NAME AND FORMULATION OF DRUG**	SARS-CoV-2 Inactivated Vaccine<br>-Inactivated SARS-CoV-2<br>-Aluminum hydroxide, disodium hydrogen phosphate, sodium dihydrogen phosphate, sodium chloride, etc.
**DOSAGE AND SCHEDULE**	Dosage: 0.5ml per dose<br>Phase **I**<br>Emergency Immunization Schedule:<br>Day 0,14<br>Routine Immunization Schedule:<br>Day 0,28<br>Phase **II**<br>Emergency Immunization Schedule:<br>Day 0,14,42 or Day 0,14<br>Routine Immunization Schedule:<br>Day 0,28,56 or Day 0,28<br>A booster dose is given 6 months after the days 0, 14 or days 0, 28 primary immunization schedule in the phase II trial.
**ROUTE OF ADMINISTRATION**	Intramuscularly, deltoid region
**CHALLENGE SCHEDULE, if applicable**	None
**BLOOD SAMPLE COLLECTION**	The blood-collection time points for different vaccination schedules were listed below:<br>**The schedule of day 0,14 (Phase I)**

	Blood collection on day 0(-7),3,7,14,17,21,28,42,194<br>**The schedule of day 0,28 (Phase I)**<br>Blood collection on day 0(-7),3,7,28,31,35,42,56, 208<br>**The schedule of day 0,14,42 (Phase II)**<br>Blood collection on day 0,28,42,45,70, 222,402<br>**The schedule of day 0,14 (plus the 6-month booster dose, Phase II)**<br>Blood collection on day 0,28,42,194;<br>1 month and 6 months after the booster dose.<br>**The schedule of day 0,28,56 (Phase II)**<br>Blood collection on day 0,56,59,84, 236,416<br>**The schedule of day 0,28 (plus the 6-month booster dose, Phase II)**<br>Blood collection on day 0,56, 208;<br>1 month and 6 months after the booster dose
**PARAMETERS OF SAFETY**	**Primary Endpoint**<br>− Incidence of adverse reactions occurred from Day 0 to Day 28 after each dose.<br>**Secondary Endpoints**<br>− Incidence of adverse reactions 7 days after each dose of vaccination;<br>− Phase I : Incidence of abnormal laboratory index (blood routine test, blood chemistry test, and urine routine test) on the 3th day after each dose of vaccination in phase I;<br>− Incidence of SAEs from the beginning of the vaccination to 6 months post the whole-schedule vaccination.<br>**Exploratory Endpoints**<br>− Phase I : The change of IL-6, IL-2, and TNF-α in serum 7 days after each dose of vaccination in phase I;<br>− Phase I: The positive rate of serum antinuclear antibody on the 7/14/21/28/42/194th day after the first dose vaccination (emergency schedule);<br>− Phase I : The positive rate of serum antinuclear antibody on the 28/35/42/56/208th day after the first dose vaccination (routine schedule);<br>− Phase II: The positive rate of serum antinuclear antibody on the 28/42/70 th day after the first dose vaccination (days 0,14,42 emergency schedule);

	− Phase II: The positive rate of serum antinuclear antibody on the 28/42/194th day after the first dose vaccination (days 0,14,194 emergency schedule);<br>− Phase II: The positive rate of serum antinuclear antibody on the 56/84th day after the first dose vaccination (days 0, 28,56 routine schedule);<br>− Phase II: The positive rate of serum antinuclear antibody on the 56/208th day after the first dose vaccination (days 0,28, 208 routine schedule).
**PARAMETERS OF IMMUNOGENICITY**	**Primary Endpoint**<br>− The seroconversion rate of neutralizing antibodies 14 days (emergency schedule)/28 days (routine schedule) after the two-dose vaccination.<br>**Secondary Endpoints**<br>− The seropositive rate, GMT, and GMI of neutralizing antibodies 14 days (emergency schedule)/28 days (routine schedule) after two-dose vaccination;<br>− The seroconversion rate, seropositive rate, GMT and GMI of neutralizing antibodies 28 days after the two doses (emergency schedule);<br>− Phase II: The seroconversion rate, seropositive rate, GMT, and GMI 28 days after the third dose (only for days 0,14,42 and days 0,28,56 schedule);<br>− Phase II: The seropositivity rate, GMT, and GMI 14 day (emergency schedule)/28 days (routine schedule) after the booster dose (only for days 0,14 and days 0,28 schedule);<br>− Phase I: The seroconversion rate, seropositive rate, GMT, and GMI 7/14/21 days after the first dose vaccination (emergency schedule);<br>− Phase I: The seroconversion rate, seropositive rate, GMT, and GMI 28/35/42 days after the first dose vaccination (routine schedules);<br>− Phase I: The seropositive rate of IgG, IgM antibodies 7/14/21/28/42 days after the first dose vaccination (emergency schedule);<br>− Phase I: The seropositive rate of IgG, IgM antibodies 28/35/42/56 days after the first dose vaccination (routine schedule).<br>**Exploratory Endpoints**<br>− Phase I: Positive rate of specific T cell response 14 days after vaccination (IFN-γ detection using Elispot);

	− The seropositive rate and GMT of neutralizing antibody at 6 months after the second dose (only for the days 0,14 and days 0,28 schedule);<br>− Phase II: The seropositive rate, GMT, and GMI of neutralizing antibody at 12 months after the third dose (only for the days 0,14,42 and days 0,28,56 schedule);<br>− Phase II: The seropositive rate and GMT of neutralizing antibody at 14 days (emergency schedule) or 28 days (routine schedule) after the booster dose (only for the days 0,14 and 0,28 schedule);<br>− Phase II: The seropositive rate and GMT of neutralizing antibody at 6 months after the booster dose (only for the days 0,14 and 0,28 schedule)

List of Vocabulary Abbreviations

ADE	Antibody Dependent Enhancement
AE	Adverse Event
ALB	Albumin
ALT	Alanine Aminotransferase
AST	Aspartate Aminotransferase
CDC	Center for Disease Control and Prevention
CDE	Center for Drug Evaluation
CFDA	China Food and Drug Administration
CK	Creatine Kinase
COVID-19	Corona Virus Disease 2019
CPK	Creatine Phosphokinase
NMPA	National Medical Products Administration
CFDI	Center for Food and Drug Inspection
CRF	Case Report Form
eCRF	Electronic Case Report Form
ELISA	Enzyme-Linked Immune-sorbent Assay
ELISPOT	Enzyme-Linked Immuno-spot Assay
EDC	Electronic Data Capture
FAS	Full Analysis Set
GCP	Good Clinical Practice
IEC	Independent Ethics Committee
ITT	Intention-to-Treat
LDH	Lactate Dehydrogenase
MedDRA	Medical Dictionary for Regulatory Activities
PI	Principal Investigator
PPS	Per Protocol Set
PT	Preferred Term
SAE	Serious Adverse Event
SARS	Severe Acute Respiratory Syndrome
SARS-CoV-2	Severe Acute Respiratory Syndrome Coronavirus 2
SOC	System Organ Class
SOP	Standard Operation Procedure
SS	Safety Set
SUSAR	Suspected Unexpected Serious Adverse Reaction

Summary of the Clinical Protocol

The COVID-19 Vaccine, Inactivated developed by Sinovac Research & Development Co., Ltd. (hereinafter referred to as "SINOVAC") is capable to induce the body to produce active immunity and prevent the disease caused by SARS-CoV-2. The results of the preliminary immunogenicity research show that the COVID-19 Vaccine, Inactivated can produce a good neutralizing antibody response in animals, which preliminarily proves the effectiveness of the vaccine. At the same time, a comprehensive safety evaluation has been conducted in animals, the preliminary results of which show that the new vaccine is safe for animals. The Clinical Trial Protocol is hereby formulated in accordance with relevant requirements of the *Provisions of Drug Registration*[1], *Good Clinical Practice* (GCP)[2], *Technical Guidelines for Clinical Trial of Vaccines*[3], *Guidelines for Quality Management of Vaccine Clinical Trial (Trial)*[4] and *Technical Guidelines for Research and Development of Vaccine for Prevention of SARS-CoV-2 (Trial)*[5].

The main objective of the Trial is to evaluate the safety and immunogenicity of the test vaccine. The randomized, double-blinded and placebo-controlled experiment design is applied. 144 healthy adults aged 18~59 year are selected as the subjects of phase I clinical trial. Upon informed consent, physical examination and screening based on the inclusion/exclusion criteria, the subjects are enrolled to be vaccinated by 2 doses respectively according to the emergency immunization schedule of day 0,14 and the routine immunization schedule of day 0,28. 72 subjects are enrolled for each immunization schedule in stages of medium and high doses respectively, each with 36 subjects, who are vaccinated by the test vaccine or placebo respectively at the ratio of 2:1. According to the occurrence of the solicited and non-solicited adverse events, and of the abnormal indexes of blood routine examination, blood biochemistry and routine urine test, the vaccination in the stage of high dose may be conducted only 0~7 days after the first dose in the stage of medium dose is vaccinated when the safety observation is completed and the safety is confirmed by DMC.

The immediate reaction within 30 minutes after each dose of vaccination is observed, the local and systemic solicited adverse events within 0~7 days and the non-solicited adverse events from the beginning of the vaccination to 28 days after the whole-schedule vaccination are collected, and the SAE monitoring from the beginning of the vaccination to 6 months after the whole-schedule vaccination is completed.

The blood of volunteers was sampled at different times before and after immunization to test the blood routine, blood biochemistry, urine routine, serum inflammatory factors and anti-nuclear antibodies and evaluate the safety of the vaccine; test the serum neutralizing antibody, IgG and IgM antibodies and IFN-γ secretory reaction of specific T cells and evaluate the immunogenicity and immunization persistence of the vaccine.

According to the occurrence of the solicited and non-solicited adverse events, and of the abnormal indexes of blood routine examination, blood biochemistry and routine urine test, phase II clinical trial may be started only 0~7 days after the first high dose of phase I clinical trial is vaccinated when the safety observation is completed and the safety is confirmed by DMC. 600 healthy adults aged 18~59 years were selected as the subjects of the clinical trial for Phase II clinical trial. Upon informed consent, physical examination and screening based on the inclusion/exclusion criteria, the subjects are enrolled to be vaccinated according to Day 0, Day 14, Day 42 or Day 0, Day 14 emergency immunization schedule or Day 0, Day 28, Day 56 or Day 0, Day 28 routine immunization schedule. 300 subjects were enrolled for each immunization schedule, randomized into 3 groups by a ratio of 2:2:1 and were vaccinated by the medium dose, high dose or placebo respectively. The immunization schedule for the subjects numbered C001~C150 was Day 0, Day 14 and Day 42, while that for the subjects numbered C151~C300 was Day 0, Day 14. The immunization schedule for the subjects numbered D001~D150 was Day 0, Day 28 and Day 56, while that for the subjects numbered D151~D300 was Day 0 and Day 28. The subjects given the third dose of Day 0, Day 14, Day 42 and Day 0, Day 28, Day 56 immunization schedule were enrolled in phases, and a total of 30 subjects numbered C001 ~ C030 were given the

third dose first, and 30 subjects numbered D001 ~ D030 were given the third dose based on the abnormalities in blood routine and blood biochemical indexes and the occurrence of adverse events on Day 0 ~3 after vaccination. After it was preliminarily confirmed safe, a total of 30 subjects numbered D001~D030 were given the third dose. It is confirmed safe through assessment, the subjects numbered C031 ~ C150 and D031 ~ D150 may be given the third dose based on the abnormalities in blood routine and blood biochemical indexes and the occurrence of adverse events of 30 subjects in each of the above groups on Day 0~7 after vaccination.

The adverse immediate reaction within 30 minutes after each dose of vaccination was observed; the local and systemic solicited adverse events on Day 0~7 and the non-solicited adverse events on Day 0~28 (Day 0~14 for the first dose under the emergency immunization schedule) after each vaccination were collected, and the SAE monitoring from the beginning of the vaccination to 6 months after full-course vaccination. The blood of volunteers is sampled at different times before and after immunization to test the serum neutralizing antibody for evaluating the immunogenicity and immunization persistence. Volunteers numbered C001~C030 and D001~D030 were required to have blood collected on D3 before and after the third dose for laboratory testing to evaluate the safety of the vaccine. If the blood routine and blood biochemical indexes before the immunization for the third dose were abnormal and had a clinical significance (Grade 2 or higher), the subjects would not be given the third dose.

Based on the immunogenicity results 6 months after 2 doses of vaccination in this study, the subjects under Day 0, Day 14 (C151~C300) and Day 0, Day 28 (D151~D300) 2-dose primary immunization schedules were given 1 dose of booster immunization 6 months after primary immunization. The adverse immediate reactions within 30 min after booster immunization were observed; local and systemic solicited adverse events on Day 0~7 and the non-solicited adverse events on Day 0~28 after inoculation were collected; and the SAE monitoring 6 months after inoculation was completed.

The Clinical Protocol will be under sole responsibility of the Investigator upon approval by the Independent Ethics Committee. The Monitor designated by the Sponsor

will conduct monitoring of the whole research process and the Data Monitoring Committee (DMC) will be established for risk assessment of the clinical trial to ensure that the Trial is carried out in a safe and standard way.

Contents

1 Introduction 18
2 Participating Organizations and their Responsibilities 18
2.1 Organization Responsible for the Clinical Trial 18
2.2 Site Organization of the Clinical Trial 20
2.3 Sponsor of the Clinical Trial 22
2.4 Organization 1 for the Trial Sample Test 24
2.5 Organization 2 for the Trial Sample Test 25
2.6 Organization 3 for the Trial Sample Test 25
2.7 Organization 4 for the Trial Sample Test 26
2.8 Monitor 27
2.9 Organization for Data Management 28
2.10 Organization Responsible for Statistical Analysis 29
2.11 Data Monitoring Committee 31
3 Background and Principle 31
3.1 Summary 31
3.2 Virology 31
3.3 Clinical Manifestation 32
3.4 Epidemiological Characteristics 33
3.5 Vaccine Research and Development 35
4 Preclinical Research and Laboratory Evaluation of Vaccine 35
4.1 Safety Research 35
4.2 Immunogenicity Research 42
4.3 Challenge Protection Research 45
4.4 Cross Neutralizing Research 48
5. Preliminary Clinical Trial 49
5.1 Safety Evaluation 49
5.2 Immunogenicity Evaluation 52
5.3 Conclusion 57
6 Introduction of Product Features 58
6.1 Preparation Technology and Formulation of the Vaccine 58
6.2 Vaccine Stability 59
6.3 Control Vaccine 59
6.4 Transportation and Storage of Vaccine 60
6.5 Inoculation Route and Procedure 60
6.6 Information of Test Product 60
6.7 Vaccine Packaging 60
7 Purpose 61
7.1 Phase I Clinical Trial 61
7.2 Phase II Clinical Trial 61
8 Test Design 61
8.1 Design 61
8.2 Endpoint 62

8.3 Study Plan 64
8.4 Randomization and Double-blind 68
8.5 Research Duration 72
8.6 Test Suspension and Early Termination 73
8.7 Protocol Violation and Deviation 73
9 Subjects 74
9.1 Inclusion Criteria for Subjects 74
9.2 Exclusion Criteria for Subjects 74
9.3 Exclusion Criteria for Second and Third Doses of Vaccination 76
9.4 Withdrawal and Termination Criteria for Subjects 77
10 Method and Schedule 77
10.1 Visit Plan 77
10.2 Recruitment and Informed Consent 90
10.3 Screening and Random Enrollment 90
10.4 Vaccination 91
10.5 Safety Follow-up and Observation 91
10.6 Sampling 92
10.7 Safety Evaluation 96
10.8 Concomitant Medication and Vaccination 105
10.9 Immunogenicity evaluation 106
10.10 Data Management 107
10.11 Statistical Analysis 108
11 Clinical Trial Monitoring 119
11.1 Sponsor's Responsibility 119
11.2 Investigator's Responsibility 119
11.3 Personnel Training 119
11.4 Compliance Guaranteeing of Subjects 120
11.5 Reported on Protocol Deviations/Violations 120
11.6 Management of Test Vaccine 120
11.7 Sample Management in Clinical Trials 121
11.8 Storage of Data on Clinical Test 121
11.9 Finished Criteria for Clinical Trial 122
12 Ethical Approval 122
12.1 Review and Approval 122
12.2 Field Supervision 122
12.3 Confidentiality 124
13 Modification of Clinical Trial Protocol 124
14 Disclosure and Publication of Data 124
15 References 125

1 Introduction

The COVID-19 Vaccine (Vero Cell), Inactivated (hereinafter referred to as "COVID-19 Vaccine") developed by Sinovac Research & Development Co., Ltd. (hereinafter referred to as "SINOVAC") is capable to induce the body to produce active immunity and prevent the disease caused by SARS-CoV-2. The results of the preliminary immunogenicity research show that the COVID-19 Vaccine, Inactivated can produce a good neutralizing antibody response in animals, which preliminarily proves the effectiveness of the vaccine. At the same time, a comprehensive safety evaluation has been conducted in animals, the preliminary results of which show that the COVID-19 Vaccine is safe for animals. The Clinical Protocol is hereby formulated to evaluate the safety and immunogenicity of the COVID-19 Vaccine.

2 Participating Organizations and their Responsibilities

2.1 Organization Responsible for the Clinical Trial

2.1.1 Responsibilities

The organization responsible for the Clinical Trial is Jiangsu Provincial Center for Disease Control and Prevention (Public Health Research Institute of Jiangsu Province), the main responsibilities of which are as follows:

- Participating in the formulation of tables and cards required by the Vaccine Clinical Trial Protocol and the Trial;
- Participating in the drafting of informed consent for vaccination, the preparation of SOP for site operation of the Clinical Trial, and the application for approval by the Independent Ethics Committee; organizing the selection and assessment of the site for the Clinical Trial that meets the requirements of GCP, and filing with the "Record Management Information Platform for Drug Clinical Trial Institutions" of the National Medical Products Administration;
- Organizing the implementation of the Clinical Trial, and performing quality control over its implementation process;
- Instructing the site, reporting Serious Adverse Events occurring during the Clinical Trial to the provincial medical products administration, Sponsor and

Independent Ethics Committee in a timely manner, and carrying out investigation and handling;

- Participating in database locking and keeping a backup of the locked database for verification;
- Reporting the implementation progress of the Clinical Trial to relevant administrative departments and writing the summary report of the Clinical Trial.

2.1.2 Organization Introduction

Jiangsu Provincial Center for Disease Control and Prevention (Public Health Research Institute of Jiangsu Province), as a non-profit public institution engaged in disease control and prevention under direct leadership of Jiangsu Commission of Health, is established by merger of former Jiangsu Provincial Sanitation and Anti-epidemic Station (Jiangsu Institute for Tuberculosis Control), Jiangsu Provincial Institute for Occupational Disease Prevention and Treatment (Jiangsu Provincial Hospital for Occupational Disease Prevention and Treatment), Health Propaganda & Education Institute of Jiangsu and Jiangsu Provincial Institute for Dermatology Prevention and Treatment (Jiangsu Provincial Monitoring Center for Venereal Disease), later adding Jiangsu Provincial Sanitation Test Center and Public Health Research Institute of Jiangsu Province, and is a first-class provincial disease control and prevention institution in China. The Center is currently governing 31 institutes (departments and offices), including the specialty disciplines such as prevention and control of acute infectious diseases, prevention and control of venereal diseases and AIDS, prevention and control of chronic non-communicable diseases, prevention and control of occupational diseases, food safety and evaluation, toxicology and function evaluation, physical and chemical testing, pathogenic microorganism research, health education and health promotion, and public health information. It has strong capabilities in scientific research, monitoring, testing, prevention and control. In recent years, Jiangsu Provincial Center for Disease Control and Prevention has made fruitful achievements in responding to various public health emergencies and emerging infectious diseases,

as well as in the follow-up scientific research. The Center has presided over and participated in the clinical research of more than 100 vaccines, such as common vaccines or other biological products for influenza, epidemic hemorrhagic fever, rabies, chickenpox, leprosy gills, meningitis, hepatitis B, tuberculosis, typhoid fever, diphtheria pertussis tetanus, haemophilus influenzae and interferon, as well as new vaccines such as the world-leading Ebola vaccine, enterovirus 71 vaccine, hepatitis E vaccine, HPV vaccine, pandemic influenza vaccine, staphylococcus aureus vaccine and influenza A H1N1 vaccine. The Institute for Vaccine Clinical Evaluation has 8 senior professional titles, 3 intermediate professional titles, 2 primary professional titles, 11 physicians and 2 laboratory technicians.

2.2 Site Organization of the Clinical Trial

2.2.1 Responsibilities

The site of the Clinical Research is located in Suining County and the research site organization is the Center for Disease Control and Prevention of Suining County, the main responsibilities of which are as follows:

- Cooperating in the assessment and filing of the test site;
- Organizing personnel with corresponding expertise and rich clinical research experience to participate in the work on the research site, and all participants to read and understand the content of the research protocol in detail and strictly observe the protocol to ensure that there is sufficient time to complete the clinical research within the time limit as specified;
- Organizing the on-site implementation, including the organization and selection of subjects, obtaining the *Informed Consent* signed by subjects, screening and enrollment, vaccination, safety visit, sample collection, serum separation, sample cryopreservation and submission;
- Data input and ensuring that all collected data are true, accurate, complete and legal;

- Accepting the monitoring and inspection by the Monitor or Inspector dispatched by the Sponsor, and the inspection and visit by the medical products administration to ensure the quality of the Clinical Research;
- Ensuring that subjects are properly handled when they suffer adverse reactions/events during the research, and in case of serious adverse reactions/events, taking appropriate treatment measures for the subjects immediately, and reporting to the Sponsor, Independent Ethics Committee and provincial medical products administration;
- Keeping relevant clinical trial data during the Clinical Trial.

2.2.2 Organization Introduction

The Center for Disease Control and Prevention of Suining County is located 200m to the north of the intersection of Suihe North Road and Yongchang Road in Suining County, with 54 persons as the authorized personnel force and 48 as the actually permanent staff. Among them, there are 42 health professionals, 28 college graduates or above, 9 junior college graduates, 5 technical secondary school graduates, 10 senior technical professionals and 7 intermediate technical professionals. The Center consists of 10 departments, namely General Department, Health Education Department, Disease Control Department, Quality Control Department, Emergency Response Office, Endemic and Parasitic Disease Prevention and Control Department, Health Department, Health Laboratory Department, Chronic Non-communicable Disease Control and Prevention Department, and Outpatient Department of CDC. It has been awarded the Advanced Collective of Disease Control and Prevention in Xuzhou and the Progress Award of Disease Control and Prevention in Xuzhou for many times. The Center's laboratory has passed the "Laboratory Qualification Accreditation" and "Food Inspection Qualification Accreditation" organized by the Quality and Technology Supervision Bureau of Jiangsu.

The Center for Disease Control and Prevention of Suining County has always been focused on laboratory construction, and now has advanced detection and testing instruments and equipment such as gas chromatography - mass spectrometer, atomic

absorption spectrometer, ion chromatography, atomic fluorescence spectrometer and PCR detector. In 2017, the government invested more than RMB 2 million for the Center to add flow injection analyzer, automatic biochemical analyzer, five-category globulimeter and other equipment. In 2017, the Center's laboratory successfully passed the provincial laboratory qualification accreditation and participated in the external proficiency testing and laboratory comparison for 9 times, all with excellent performance. At present, the Center has 143 test items passing the qualification accreditation or recognition, and the coverage of laboratory accreditation is 80%.

The Center for Disease Control and Prevention of Suining County has undertaken many projects for consecutive years, including the Measles, Rubella and Parotitis Immunity Level Monitoring and Etiology Surveillance Project, the Fifth Round Global Fund Malaria Project, the Global Fund AIDS Project, the National Normal Iodine Program, the Comprehensive Mosquito-borne Disease Monitoring Project in Jiangsu, the Adult Tobacco Epidemic Survey in China and the National Residents' Health Literacy Monitoring Project.

It has successively undertaken phase I clinical trial of the typhoid A paratyphoid conjugate vaccine of Royal (Wuxi) Bio-pharmaceutical Co., Ltd., phase III clinical trial of Influenza Vaccine (Split Virion), Inactivated, Quadrivalent of Shanghai Institute of Biological Products Co., Ltd. and phase Ib clinical trial of recombinant staphylococcus aureus vaccine (escherichia coli) of Olymvax Biopharmaceuticals Inc. On June 27-28, 2018, it accepted the GCP site inspection by the former Center for Food and Drug Inspection of China Food and Drug Administration.

2.3 Sponsor of the Clinical Trial

2.3.1 Responsibilities

The Sponsor of the Clinical Trial is Sinovac Research & Development Co., Ltd., the main responsibilities of which are as follows:

- Providing the preliminary clinical trial protocol and approving the final protocol with signature and seal.

- Providing approval documents for the Clinical Research, the Investigator's Brochure for the Clinical Trial (preclinical safety information of the product), executive standards for products and other site application documents.
- Providing vaccines for the Clinical Research and issuing the acceptable verification report.
- Evaluating and selecting the responsible organization and site of the Clinical Trial, assigning the Monitor to perform the assessment and accreditation of the site of the Clinical Trial and the monitoring duties according to the GCP requirements, and being ultimately responsible for the quality of the Clinical Trial.
- Participating in the investigation and handling of the cases with abnormal reaction to vaccine, and providing medical treatment and relevant compensation for the cases with clinically proven abnormal reaction related to the vaccination according to relevant regulations. For other cases, please refer to the Working Agreement.
- Providing funds for the Clinical Research.

2.3.2 Organization Introduction

Sinovac Research & Development Co., Ltd., as the former R&D Center of Sinovac Biotech Co., Ltd. and a biological high-tech enterprise solely-invested and established by Sinovac Biotech (Hong Kong) Limited, was incorporated in 2009, with the registered capital of USD 9.60 million. The Company is a Zhongguancun high-tech enterprise and Zhongguancun gold seed enterprise.

SINOVAC is specialized in the research, development and technical services of vaccines for human use and related products to provide technical support for the prevention and control of serious infectious diseases. Relying on the Group's advantages in vaccine research and development and industrialization over the years, the Company has gradually formed a research and development mode with enterprises as the main body of research and development and the combination of the efforts of enterprises, universities and research institutions, and built the virus isolation

identification technology platform, cell factory platform, microcarrier fermentation technology platform, virus purification technology platform, bacterial fermentation and purification platform, polysaccharide-protein combination technology platform, freeze-drying technology platform, animal evaluation platform, quality control platform and diagnostic reagent raw materials development platform, the expertise of which complements each other's advantages with cross penetration to promote the stable and efficient progress of Company's research and development.

SINOVAC has undertaken 2 special projects of national major new drug development and one science and technology program in Beijing, and obtained 12 authorized patents for invention in China. The clinical research of the 23-valent pneumopolysaccharide vaccine developed by the Company has been successfully completed and its industrialization has been realized in Sinovac. The Company is developing DPT polio Hib series combined vaccine, 13-valent pneumococcus conjugate vaccine, recombinant hepatitis B vaccine and other varieties.

2.4 Organization 1 for the Trial Sample Test

2.4.1 Responsibilities

Traditional Chinese Medicine Hospital of Suining County, the main responsibilities of which are as follows:

- Performing blood routine examination, blood biochemistry and routine urine test.

2.4.2 Organization Introduction

The Traditional Chinese Medicine Hospital of Suining County is a tertiary general traditional Chinese medicine hospital integrating medical treatment, first aid, teaching, scientific research, prevention, health care and rehabilitation with advanced equipment, powerful technology, complete specialties and orderly management. It is a "Baby Friendly Hospital" named by the Ministry of Health, a teaching hospital of Nanjing University of Chinese Medicine, a "Safe Hospital" in Jiangsu, a demonstration unit of "Safe Consumption" in Jiangsu, a member of the "Cooperative Development Consortium of Traditional Chinese Medicine Hospitals in Nanjing Metropolitan Area" in Jiangsu, and a "Technical Collaboration Hospital of Traditional Chinese Medicine

Hospitals in Jiangsu”. In recent years, it has been awarded the “People's Satisfaction Hospital” and “Civilized Unit” in Xuzhou.

The Hospital is divided into south and north areas, where the south area has a floor area of 36.4mu and a building area of 48,000m^2. There are 28 clinical departments, 10 medical laboratories, 14 wards, 400 approved beds and 715 actually open beds. There are currently 1,177 employees, including 283 with intermediate and senior titles, 2 doctoral students and 11 postgraduates. The Hospital is well equipped with all kinds of modern diagnostic and treatment equipment, currently with 510 pieces of modern diagnostic and treatment equipment at a value over RMB 10,000. The north area of the Hospital is an important livelihood project in Suining County and is expected to be put into use in 2020 according to the requirements and construction standards of a tertiary hospital. The completion of the branch will make the Hospital the largest and most complete modern hospital with green gardens, ecological environment protection, low carbon and energy saving in Suining County, and provide reliable guarantee for the construction of a tertiary traditional Chinese medicine hospital leading in the North Jiangsu Region.

2.5 Organization 2 for the Trial Sample Test

2.5.1 Responsibilities

The Center for Disease Control and Prevention of Suining County, the main responsibilities of which are as follows:

- IgG and IgM screening of volunteers before enrollment;
- PT-PCR nucleic acid tests by throat swab and anal swab of volunteers.

2.5.2 Organization Introduction

See Section 2.2.2.

2.6 Organization 3 for the Trial Sample Test

2.6.1 Responsibilities

National Institutes for Food and Drug Control, the main responsibilities of which are as follows:

- Test of serum neutralizing antibody, IgG, IgM and anti-nuclear antibodies in

enrolled subjects.

2.6.2 Organization Introduction

National Institutes for Food and Drug Control is a public institution directly under the National Medical Products Administration, the national statutory body and the supreme technical arbitration body for testing the quality of medicines and biological products, and the "WHO Collaborating Center for Drug Quality Assurance" designated by the World Health Organization. In accordance with the laws, it implements the approval and registration inspection, import inspection, supervision inspection and safety assessment of medicines, biological products, medical devices, food, health food, cosmetics, experimental animals, packing materials and other products in various fields, as well as lot release of biological products, is responsible for the research, distribution and management of culture and virus seed used for the reference material and production verification of national drugs and medical devices, and carries out related technical research.

2.7 Organization 4 for the Trial Sample Test

2.7.1 Responsibilities

Jiangsu Huatai Vaccine Engineering Technology Research Co., Ltd., the main responsibilities of which are as follows:

- Detecting the IFN-γ secretion by T cell response;
- Detecting the serum inflammatory factor.

2.7.2 Organization Introduction

Jiangsu Huatai Vaccine Engineering Technology Research Co., Ltd., as a wholly state-owned holding company in Taizhou Pharmaceutical High-tech Industrial Development Zone with the registered capital of RMB 50 million, is mainly engaged in research and development of vaccine engineering technology, research and development of biological products, clinical evaluation, technology transfer and consultation. The Company has 22 professional service staff, including 2 doctors and 10 masters. Relying on the Administrative Committee of Taizhou Pharmaceutical

High-tech Industrial Development Zone, the Company has established partnership with Chinese Academy of Medical Sciences, National Institutes for Food and Drug Control, Nanjing University, Jiangsu Provincial Center for Disease Control and Prevention and Vaccine Research and Development Center of Taiwan Health Research Institute, and has a number of expert consultants. Its existing laboratories cover an area of 13,000m^2, including the testing laboratory, large-scale instrument sharing laboratory, R&D incubation laboratory, phase I clinical evaluation base, vaccine production pilot line and bulk filling workshop. In addition, there is an office service space of 6,000m^2. The Vaccine Engineering Center has established a vaccine research and development technology center, a pilot production base for biological products, a vaccine clinical evaluation center and a biological product testing center, and has the qualifications and abilities to independently provide external technical services.

The Company is in technical cooperation with 20 enterprises for biological products under research, mainly including Qyuns Therapeutics Co., Ltd., Ab&b Bio-Tech Co., Ltd. JS, Jiangsu Rec-Biotechnology Co., Ltd. and Jiangsu Saihua Biological Technology Co., Ltd., with 420 R&D personnel. There are more than 350 sets of main instruments and equipment, including ultra-performance liquid chromatograph, separation flow cytometer, molecular interaction instrument, purifier, ultra-speed centrifuge, fluorescence quantitative PCR and biochemical analyzer, with a value of more than RMB 40 million.

Ongoing research projects of the cooperators of the Company mainly include VERO rabies vaccine, VLP cervical cancer vaccine, quadrivalent influenza vaccine, monoclonal antibody drugs and Biosimilar products, including 4 Class I innovative drugs. It has obtained 8 approval documents for new drug clinical trials in recent years, and completed phase I clinical evaluation experiment of Ebola, anthrax, cholera vaccine projects.

2.8 Monitor

2.8.1 Responsibilities

The Clinical Research Department of Sinovac Biotech Co., Ltd. is responsible for

the monitoring of the Clinical Trial.

- Conducting monitoring of the Clinical Trial according to GCP, protocol and SOP;
- Assisting the Sponsor in undertaking the screening and training of the institutions for the Clinical Trial, holding kick-off meeting and other work;
- Verifying the test process and progress;
- Verifying the signing of informed consent;
- Verifying the qualifications of the investigators and the effectiveness of the implementation equipment;
- Verifying the transportation, storage, distribution, use, return and disposal of the vaccines for the Clinical Trial;
- Verifying the collection, storage and transportation of biological samples;
- Verifying the handling of adverse events;
- Verifying the logicality of the original records and the report documents in the Trial;
- Completing the monitoring after the Trial, etc.

2.8.2 Organization Introduction

Since its establishment in 2002, Sinovac Clinical Research Department has independently undertaken the organization, implementation, monitoring, data and statistical analysis management of many clinical trials, including inactivated hepatitis A vaccine, hepatitis A and B combined vaccine, SARS vaccine, H1N1 vaccine, H5N1 vaccine, EV71 vaccine, 23-valent pneumococcus vaccine, varicella vaccine, inactivated poliomyelitis vaccine, and quadrivalent influenza vaccine, making it experienced in clinical trial organization, implementation and management.

2.9 Organization for Data Management

2.9.1 Responsibilities

Meida Kelin (Nanjing) Medicine Technology Co., Ltd. is responsible for clinical trial data management.

- Formulating the Data Management Plan and Data Validation Plan according to protocol requirements;
- Providing EDC and other related online services;
- Carrying out data management in accordance with the *Technical Guidelines for Clinical Trial Data Management* during the Trial, and confirming that all data reports and records are correct and complete;
- Conducting data cleaning, raising questions about research data, and assisting investigators in verification and clarification;
- Preparing the data management report.

2.9.2 Organization Introduction

Meida Kelin (Nanjing) Medicine Technology Co., Ltd. established in September 2014, is a Contract Research Organization (CRO) mainly engaged in the outsourcing of data related services in clinical trials for domestic and foreign pharmaceutical companies. It has now offices in Shanghai, Beijing, Xi'an and Shenyang, and is in a strategic partnership with the CRO Clinical Service Center providing all-round services. It has provided data management, statistical analysis and drug safety alert services for phases I~IV and bioequivalence clinical trials of the innovative drugs and generic drugs of dozens of domestic and foreign pharmaceutical companies. It has:

- Standard Operating Procedure (SOP) and strict quality management system that meet the requirements of ICH-GCP/FDA 21 CFR part 11/international or domestic clinical trials;
- Personnel familiar with the clinical trial design, implementation, data management and statistical analysis experience in China, US, EU, Japan, South Korea and other countries, and with relevant drug management regulations and implementation rules;

  Complete education and training system.

2.10 Organization Responsible for Statistical Analysis

2.10.1 Responsibilities

Beijing KEY TECH Statistical Technology Co., Ltd. is responsible for the

statistical analysis of the Clinical Trial.

- Writing the randomization, sample size and statistical analysis parts of the Clinical Trial Protocol;
- Writing the statistical analysis plan according to Clinical Trial Protocol;
- Conducting the randomization and blinding of the Clinical Trial;
- Carrying out the statistical analysis according to the proposed statistical analysis plan and writing the statistical analysis report.

2.10.2 Organization Introduction

Beijing KEY TECH Statistical Technology Co., Ltd. (referred to as "KEY TECH"), incorporated in August 2017 in Beijing, is a wholly domestic-funded company engaged in data management and statistical analysis services of clinical trial. It focuses on the biostatistics service of clinical research, and mainly provides, with respect to registered clinical trials, the statistical strategy consultation for drug research plan throughout the whole process, the statistical design and statistical analysis, etc. KEY TECH has currently set up offices in Beijing, Xi'an and Nanning, now with 43 employees, who have mainly graduated from the Fourth Military Medical University, Peking University, Sichuan University and other first-class universities in China. At present, there are 21 statisticians/statistical programmers, 18 data managers, 1 quality control person and 3 non-business personnel among the employees. In terms of education background, there are 3 doctors, 6 masters and 34 bachelors.

Since its establishment, KEY TECH have assisted the Sponsor in obtaining 8 approval documents for clinical trial, completed 18 new drug applications, including 5 Class I new drugs of biological products, and obtained the approval for marketing of 5 products in the applied projects, including the first 13-valent pneumonia vaccine in China, nasal spray influenza vaccine, the second Adalimumab monoclonal antibody product in China, the third quadrivalent influenza vaccine in China and varicella vaccine. KEY TECH signed an agreement with Abbott in 2019 for statistical consulting services in the Asia Pacific Region, establishing long-term partnership with leading innovative pharmaceutical companies at home and abroad.

2.11 Data Monitoring Committee

The Data Monitoring Committee consists of specialists in clinical medicine, epidemiology and statistics.

Its main responsibilities are as follows:

– Performing safety data review and clinical trial risk assessment to ensure that the Trial is carried out in a safe and standard way.

3 Background and Principle

3.1 Summary

Since December 8, 2019, several cases of pneumonia for unknown cause were reported in Hubei, with most of the patients working or living in South China Seafood Wholesale Market where live animals are sold. At early stage, this pneumonia presented severe symptoms of acute respiratory infection, with some patients rapidly developing to acute respiratory distress syndrome (ARDS). This pneumonia, which was later proved to be human-to-human transmission, escalated rapidly in early January 2020, and there were cases found in provinces of China and more than 20 other countries, including Japan, Singapore and US. Chinese Center for Disease Control and Prevention (CDC) identified a novel coronavirus from a patient’s throat swab sample on January 7, 2020. The World Health Organization (WHO) declared the pneumonia outbreak caused by the novel coronavirus to be a public health emergency of international concern (PHEIC) on January 31, 2020. WHO declared the outbreak to enter the international pandemic phase on March 12, 2020.

As shown by research, the novel coronavirus gene sequences were most closely associated with two SARS-like coronaviruses from bat (bat-SL-CoVZC45 and bat-SL-CoVZXC21)[6]. International Committee on Taxonomy of Viruses (ICTV) declared the official class name of this novel coronavirus as Severe Acute Respiratory Syndrome Coronavirus 2 (SARS-CoV-2) on February 12, 2020, while WHO declared the official name of the disease caused by the virus as COVID-19 on the same day.

3.2 Virology

Coronavirus (COV) is an important pathogen of human and vertebrate that can infect the respiratory tract, gastrointestinal tract, liver and central nervous system of humans, livestock, birds, bats, mice and many other wild animals. Since the outbreak of Severe Acute Respiratory Syndrome (SARS) in 2003 and Middle East Respiratory Syndrome (MERS) in 2012, the possibility for COVs to transmit from animals to humans has been proved. COVs belongs to the Coronavirinae subfamily of the Nidovirales coronavirus family, which includes four genera: α-coronaviruses, β-coronaviruses, γ-coronaviruses, and δ-coronaviruses[7].

SARS-CoV-2 is from genus β that is enveloped with round or oval and often pleomorphic particles in the diameter of 60-140nm, and is a plus-stranded RNA virus. Its gene characteristics are clearly different from those of SARSr-COV and MERSr-COV. The present research shows that it has more than 85% homology with bat SARS-like coronavirus (bat-SL-COVZC45). SARS-CoV-2 could be found in respiratory epithelial cells in about 96h when isolated and cultured in vitro, while in about 6 days when isolated and cultured in Vero E6 and Hun-7 cell lines.

So far, the full-length genome sequences among virus samples are almost identical, suggesting that no significant virus variation has taken place. Close monitoring of SARS-CoV-2 also shows that no significant variation has been found in viruses isolated from the environment, previously isolated from humans and recently isolated[8]. However, there is still the possibility of future mutation or recombination in which the virulence may increase or decrease.

The understanding of physicochemical properties of coronavirus mainly comes from the researches of SARS-COV and MERS-COV. The virus is sensitive to ultraviolet and heat. At 56°C for 30 minutes, diethyl ether, 75% ethanol, chlorine-containing disinfectant, peracetic acid, chloroform and other lipid solvents can effectively inactivate the virus, but chlorhexidine cannot[9].

3.3 Clinical Manifestation

According to the current epidemiological survey, the incubation period is 1-14 days, mostly 3-7 days, with fever, fatigue and dry cough as the main manifestations. A

few patients have symptoms such as nasal congestion, runny nose, sore throat, myalgia and diarrhea. Critical patients often develop dyspnea and/or hypoxemia one week after attack, with rapid development to acute respiratory distress syndrome, septic shock, incorrigible metabolic acidosis, haemorrhagic and coagulation dysfunction in severe cases. It is worth noting that the course of the disease in the severe and critical patients may present moderate to low fever, and even no obvious fever.

Some cases of children and newborns show atypical symptoms, such as vomiting, diarrhea and other digestive tract symptoms or only mental weakness and polypnea. Mild patients only show low fever, fatigue, etc., without pneumonia.

The prognosis of most patients is favorable and a few patients are in critical condition. The elderly and those with chronic underlying diseases have a poor prognosis. The clinical course of pregnant and birth-giving women with COVID-19 is similar to that of patients of the same age, and children have relatively mild symptoms[9].

3.4 Epidemiological Characteristics

Transmission routes and susceptible groups

The main source of infection of COVID-19 is the patients infected by SARS-CoV-2, and asymptomatic carriers may also become the source of infection. The main routes of transmission are respiratory droplets and contact transmission, the virus is transmitted through the droplets produced during patients' cough, sneeze and talk, susceptible people inhaling them will be infected, and the population is generally susceptible. Exposure to high concentration of aerosol in a relatively closed environment for a long time presents the possibility of aerosol transmission.

The fecal-oral transmission route remains to be determined. Recently, SARS-CoV-2 was detected in the feces of the confirmed patients in Wuhan, Shenzhen and even the US in the first case, indicating that the virus can replicate and exist in the digestive tract and suggesting that fecal-oral transmission is possible[10], but it has not been established that eating food contaminated by the virus will cause infection and transmission. There is also a view that the virus in feces may be retransmitted by aerosol formed by droplets containing the virus, and further investigation is needed.

It has been currently reported that there is the case where the mother is a confirmed

COVID-19 patient and the newborn is with positive viral nucleic acid by throat swab 30h after birth, suggesting that SARS-CoV-2 may cause neonatal infection through mother-to-child transmission[11].

Epidemic Status of COVID-19 in China

As of 24:00 on June 11, 2020 (Beijing time), there have been 83,064 confirmed cases of COVID-19 in China, with 4,624 deaths[12]. As pointed out in the *Joint Investigation Report of China-WHO on Novel Coronavirus Pneumonia (COVID-19)*[13], among the 55,924 laboratory confirmed cases reported, the median of age is 51 years old, the age range is 2 days~100 years old, and the interquartile range is 39~63 years old. Most of the cases (77.8%) are in the range of 30~69 years old. Among them, males account for 51.1%, cases from Hubei for 77% and peasants or manual workers for 21.6%.

In China, human-to-human transmission of COVID-19 mainly occurs within families. Detailed information on continuous transmission among family members in some provinces can be obtained from cluster case surveys and some family transmission case studies. There are 1,836 cases in total in Guangdong and Sichuan, and the reported 344 cluster cases involve 1,308 cases, of which 78%~85% occur in family members. Research on the transmission within family members is ongoing, but preliminary findings in Guangdong estimate that the second-generation secondary attack rate in family members is about 3%~10%. As the pandemic continues, community cluster infections are also increasing with hospital cluster attack, although family cluster infections are dominant[13].

Epidemic Status of COVID-19 in the World

As of 2:25 p.m. on June 13, 2020 (CEST), a total of 7,553,182 COVID-19 confirmed cases and 423,349 deaths had been reported across the world. The countries with a high incidence of COVID-19 infection are the US (2,010, 391 cumulative confirmed cases), Brazil (802,828 cumulative confirmed cases), Russia (520, 129 cumulative confirmed cases), India (308,993 cumulative confirmed cases), the United Kingdom (292,954 cumulative confirmed cases), Spain (243,209 cumulative confirmed cases), Italy (236,305 cumulative confirmed cases), Peru (214,788 cumulative confirmed cases), German (186,022 cumulative confirmed cases), Iran (182,545 cumulative confirmed cases) and Turkey (175,218 cumulative confirmed cases). The COVID-19 has spread to global 216 countries around the world, leading to a global

COVID-19 pandemic.[12]

3.5 Vaccine Research and Development

There are no approved specific therapies or vaccines against COVID-19. According to the data published by WHO, there were 119 candidate vaccines under development, 110 candidate vaccines in preclinical studies, and 9 candidate vaccines in clinical trials, including 1 candidate vaccine in phase I clinical trials, 2 candidate vaccines in phase II clinical trials, and 6 candidate vaccines in phase I/II clinical trials as of May 15, 2020. According to the technical route, 48 of the candidate vaccines are recombinant protein vaccines (mainly subunit vaccines and virus-like particles); 30 are nucleic acid vaccines (including 20 mRNA vaccines and 10 DNA vaccines); 27 are vector vaccines (using 9 vectors, including adenovirus, influenza virus, poxvirus, measles virus vector, vesicular stomatitis virus, yellow fever virus, rabies virus, Newcastle disease virus and avian paramyxovirus); 8 are inactivated vaccines (under development in China, Japan, USA and Kazakhstan); 2 are attenuated live vaccines (under development in India); 4 are other vaccines (therapeutic vaccines).

4 Preclinical Research and Laboratory Evaluation of Vaccine

4.1 Safety Research

The toxicity test of single administration in rats, active systemic anaphylaxis test in guinea pigs, toxicity test of repeated administration in rats, toxicity test of repeated administration in macaca fascicularis and reproductive development toxicity test in rats have been carried out for the test vaccine, the results of which are as follows:

4.1.1 Toxicity test of single administration in rats

Test objective: to evaluate the possible acute toxicity in Sprague-Dawley (SD) rats within 14 days after single intramuscular injection of COVID-19 Vaccine, so as to provide animal experimental data for subsequent research.

Experimental design: according to the body weight of animals measured before administration, 20 animals with similar body weight and quarantine inspection passed were selected for the test, with half males and half females, which were randomly

divided into 2 groups by sex section, namely the test group and the negative control group. The rats in the test group were intramuscularly injected with the proposed clinical high-dose vaccine by 0.5ml/1200SU/rat and the rats in the negative control group were intramuscularly injected with normal saline by 0.5ml/rat, which were observed for 14 days after the administration and further observed by gross anatomy.

Test results: No death or near-death was observed in the animals of both the test group and the negative control group, and no abnormalities were observed in clinical observation. The body weights of the animals in all groups normally increased, and no statistical differences were observed in the body weights of animals in the test group when compared with the animals in the negative control group of same sex during the same period, and no significant effects of drug administration on animal intake were observed. Visual observations of pathological gross autopsy showed that no abnormities were seen in major organs and tissues of the animals in all the groups.

Conclusion: No abnormalities associated with administration were seen in SD rats inoculated with high-dose vaccines in clinic, and the maximum tolerated dose (MTD) in SD rats was greater than or equal to 1,200 SU/1 dose/rat.

4.1.2 Active systemic anaphylaxis test in guinea pigs

Test objective: to observe the immediate systemic anaphylaxis in guinea pigs sensitized by intramuscular injection of COVID-19 Vaccine (once every other day, for 3 times in total) and stimulated by intravenous injection on D19 and/or D26, so as to provide animal experimental data for clinical research of the test article.

Experimental design: according to the body weight of animals measured before administration, animals with similar body weight were selected, and 36 Hartley guinea pigs were randomly divided into 4 groups, i.e., low-dose test group, high-dose test group, negative control group and positive control group, respectively sensitized by intramuscular administration on D1, D3 and D5 and stimulated by intravenous administration on D19 (14 days after the last sensitization) and D26 with 0.5ml/1200SU/dose test article, normal saline and human hemoglobin. The first 3 animals in each group were stimulated by intravenous injection in the feet, and the

stimulation dose in each group was twice the sensitization dose. Clinical observation was performed after administration. The experimental design is shown in the table below:

Table 1 Experimental Design of Active Systemic Anaphylaxis in Guinea Pigs

Group	Test article/ control	Qty. of animals	Sensitization (i.m) D1,D3,D5		Stimulation (i.v) D19 and D26	
			Dosage of administration	Capacity of administration (mL/Nr.)	Dosage of administration	Capacity of administration (mL/Nr.)
1	Negative control	4	0	0.5	0	1
2	Positive control	4	20 mg/Nr.	0.5	40 mg/Nr.	1
3	Low-dose test article	4	0.1 dose/Nr.	0.05	0.2 dose/Nr.	0.1
4	High-dose test article	4	1 dose/Nr.	0.5	2 doses/Nr.	1

Test results: no abnormal reaction was observed in general clinical observation. The body weight of animals in each group was in normal growth according to the weighing before grouping, before the last sensitization and before administration on the day of stimulation respectively. The low-dose group, high-dose group and negative control group all showed negative anaphylaxis. The positive control group was stimulated on D19 and D26 and showed positive anaphylaxis.

Conclusion: no allergic reaction was found in SD rats inoculated with high-dose vaccines in clinic.

4.1.3 Toxicity test of repeated administration in rats

Test objective: to evaluate the toxicity and determine target organs for toxicity and recovery of toxic reaction in SD rats after repeated intramuscular injection of COVID-19 vaccine, determine the safe dose for repeated administration, and provide basic data for clinical trial and application of the test articles.

Experimental design after administration of three doses: according to the body weight of animals measured before grouping, 150 animals with similar body weight and satisfactory quarantine inspection results were selected, which were randomized into 7 groups by sex for the main test groups (1~4 groups, i.e. low-dose test group, high-dose test group, negative control group and adjuvant control group) and satellite groups

(5~7 groups, i.e. low-dose test group, high-dose test group and negative control group). There were 15 animals per sex in each main test group and 5 animals per sex in each satellite group. The rats in the low-dose test group, high-dose test group, negative control group and adjuvant control group were administrated by intramuscular injection of 0.5ml/300SU/dose of test article, 0.5ml/1200SU/dose of test article, 0.5ml/dose of normal saline and 0.5ml/dose of adjuvant respectively on D1, D8 and D15, and safety observation was performed until 4 weeks after the last administration. Test indexes include: clinical observation such as anaphylaxis and local injection reaction, body weight/body temperature/food intake/ophthalmic testing, cliniopathological markers (blood counts, coagulation function, blood biochemistry and urine analysis), immunological indexes (T lymphocyte subsets, cytokines and antibodies) and pathological examination (gross anatomical observation and histopathological examination).

Experimental design after administration of four doses: 80 SD rats (half males and half females) aged 5-6 weeks were selected and randomized into 2 experimental groups according to body weight: negative control group (CN group) and COVID-19 vaccine group (T group), with 40 animals in each group, including 30 animals in the main test group and 10 animals in the satellite group. The doses were administered intramuscularly at a dose of 1,200 SU/0.5 ml/rat/dose. Doses were administered at weeks 0, 1, 2 and 3 respectively, totaling to 4 doses as per 0.5 ml/rat, with a recovery period of 2 weeks. The testing indexes were the same as 3 doses.

Test results: after 3 or 4 doses were administrated, no abnormalities were found in the test animals of each group during index observation; body temperature did not abnormally rise after administration; body weight fluctuated in a small range; no change related to administration was found. After 3 doses were administrated, no definite abnormality related to administration was found in the fibrinogen measured on D2 and D4 and in the blood cell counts measured on D4. At last dose had been administrated during test on 4 doses, 12 test animals showed muscle interstitial inflammatory cell infiltration at the injection sites, including one with fibroblast hyperplasia. At the end of the recovery period, 3 animals showed muscle interstitial inflammatory cell

infiltration at the injection sites.

Test conclusions: The COVID-19 vaccine was given to rats by repeated intramuscular injections once a week for consecutive 3 or 4 times. During administration and at the end of the recovery period, no systemic toxic reactions were observed in animals at doses of 300 SU/rat and 1,200 SU/rat. It was determined that no observed adverse effect level (NOAEL) was 1,200SU/rat. Local irritation related to aluminum adjuvant was seen at some local injection sites, and no immunotoxic reactions were observed.

4.1.4 Toxicity test of repeated administration in macaca fascicularis

Test objective: to evaluate the possible toxicity and target organ in macaca fascicularis after 4 weeks of repeated intramuscular injection of COVID-19 Vaccine, as well as the recovery of toxicity after 4 weeks of withdrawal, so as to provide animal experimental data for clinical research of the test article.

Experimental design: according to the body weight of animals measured before grouping, 40 animals were randomly divided into 4 groups by sex section, i.e. low-dose test group, high-dose test group, negative control group and adjuvant control group, with 10 macaca fascicularis in each group, half males and half females, which were administrated by intramuscular injection of 0.5ml/300SU/dose of test article, 0.5ml/1200SU/dose of test article, 0.5ml/dose of normal saline and 0.5ml/dose of adjuvant respectively on D0, D7 and D14, and safety observation was performed until 14 days after the last administration for euthanasia anatomy. Test indexes include clinical observation such as anaphylaxis and local injection reaction, body weight/body temperature/electrocardiogram/blood pressure/ophthalmic testing, cliniopathological markers (blood counts, coagulation function, blood biochemistry and urine analysis), immunological indexes (T lymphocyte subsets, cytokines, C-reaction protein, alexin and antibodies) and pathological examination (gross anatomical observation and histopathological examination).

Result: During the test, no death or near death was observed in the animals of each group. Clinical observations showed no abnormalities related to drug administration

and no abnormal changes in body weight, body temperature, electrocardiogram, blood pressure or eye examination resulted from drug administration. No abnormal clinicopathological indexes or immunological indexes associated with drug administration were observed. Pathological examination showed that, on Day 3 after the last administration (Day 18), granulomatous inflammation or single cell infiltration was visible locally in 5/6, 6/6 and 5/6 animals in the adjuvant control and low/high-dose groups respectively, with mild to moderate lesions; this change was considered a local reaction caused by accumulation of aluminum adjuvant, which was an expected reaction caused by intramuscular injection of a vaccine containing aluminum adjuvant. At the end of the 2-week recovery period, macrophage granulomatous inflammation was still visible locally in 3/4, 4/4 and 4/4 of the animals in the adjuvant control group and low/high-dose groups respectively, suggesting that the local irritant response of drug administration had not recovered.

Test conclusions: The COVID-19 vaccine was given to machin by repeated intramuscular injections once a week for consecutive 2 weeks, totaling to 3 doses. During administration and at the end of the 2-week recovery period, no systemic toxic reactions were observed in animals at doses of 300 SU/rat and 1,200 SU/rat. It was determined that no observed adverse effect level (NOAEL) was 1,200SU/rat. Local irritation related to aluminum adjuvant was seen at some local injection sites, and no immunotoxic reactions were observed.

4.1.5 Reproductive development toxicity test in rats

Test objectives: evaluate the effects of repeated intramuscular injection of COVID-19 Vaccine in SD rats before mating to duration of pregnancy until lactation period on the fertility of male and female rats, and on the development of pregnant/lactation female rats, fetus and fetal rats, understand the effect of the vaccine on the development of teratogenic fetal rats and offspring rats, and study the antibody level in the blood of fetus or offspring rats, so as to provide reference for the safe administration to special population in the Clinical Trial.

Experimental design: according to the body weight of animals measured before

administration, the animals were randomly divided into 4 groups by sex section, i.e. low-dose test group, high-dose test group, negative control group and adjuvant control group, with 28 males and 56 females in each group, which were administrated with 0.5ml/300SU/dose of test article, 0.5ml/1200SU/dose of test article, 0.5ml/dose of normal saline and 0.5ml/dose of adjuvant respectively. The male rats were administrated for 4 times before mating, on D1, D8 and D15 respectively. Female rats were administrated for 3 times before mating, on D1, D8 and D15 respectively. Female and male rats started mating in a cage one week after the end of the administration to male rats, and female rats were administrated once respectively on gestational day 6 (GD6) and on postnatal day 7 of offspring rats (PND7). 1/2 of the pregnant rats in each group had a cesarean section on GD20 for fetal examination (appearance, viscera and bone examination), and the remaining 1/2 in the same group were given natural delivery and euthanized at the end of lactation period. Clinical observations were made during the test, and the parental male and female rats were examined for body weight, food intake and reproductive capacity; the necropsied male rats had a caesarean section on GD20 to examine the development of embryo-fetal rats, appearance of fetal rats, bone and viscera; the sperm viability, counting and morphology of parental male rats were examined, and the histopathology of main reproductive organs of parental male and female rats were examined; the survival, body weight, body and emission development index of F1 offspring rats after delivery were examined; the serum of male rats and fetal rats necropsied on GD20 and parturient female rats and F1 offspring rats were tested for specific IgG antibodies and neutralizing antibodies against SARS-CoV-2.

Test results: the COVID-19 Vaccine, Inactivated was repeatedly injected into SD rats by means of intramuscular injection at doses of 300 SU/rat and 1,200 SU/rat before mating to the embryonic implantation and parturition, and there were no effects on the fertility of male and female rats as well as growth and development of F1 offspring rats, no obvious adverse reaction on the pregnant / lactant female rats, no developmental toxicity and teratogenicity of embryo-fetal rats and no effects on the growth and development of F1 offspring rats. Besides, the specific IgG antibodies and neutralizing antibodies against SARS-CoV-2 can be detected in the serum of male rats and fetal rats

as well as parturient female rats and F1 neonatal rats necropsied on GD20 in the high/low-dose groups of the test articles, with a detectable rate of 100%.

4.2 Immunogenicity Research

By intraperitoneal immunization of mice and intramuscular immunization of rats from inactivated novel coronavirus using different dosages, adsorption ways and immunization schedules, blood samples were collected at different times to determine the titer of serum neutralizing antibody and IgG antibody after immunization, evaluate the immunogenicity of the COVID-19 Vaccine (Vero Cell), Inactivated (hereinafter referred to as “COVID-19 Vaccine”) and determine the formulation, dosage and immunization schedules of the vaccine according to the immunogenicity results.

Research design:

- Determination of aluminum adsorption and non-aluminum adsorption processes for the vaccine

1200SU/0.5ml, 600 SU/0.5ml, 300 SU/0.5ml and 150 SU/0.5ml COVID-19 Vaccine with aluminum adjuvant and 1200SU/0.5ml, 600 SU/0.5ml and 300 SU/0.5ml COVID-19 Vaccine without aluminum adjuvant were respectively prepared by two different processes, which were used for intraperitoneal immunization of mice as per 10 mice/group and 0.5ml/mouse, and for immunization by one dose, the serum was sampled on D7, D14 and D21 after immunization; for day 0,7 and day 0,14 immunization by two doses, the serum was sampled on D14, D21 and D28 to test the titer of IgG antibody in serum. Negative control animals were also set. By comparing the antibody levels, the immunogenicity of the vaccines prepared by two different processes was compared, with the specific research design shown in the table below:

Table 2 Comparative Research Design for Immunogenicity of Aluminum Adsorption/Non-aluminum Adsorption COVID-19 Vaccine

Dosage (SU/0.5ml)	COVID-19 Vaccine, Inactivated				Non-aluminum adsorption COVID-19 Vaccine, Inactivated			
	Vaccine lot no.	Immunization by one dose	Day 0,7 immunization by two doses	Day 0,14 immunization by two doses	Vaccine lot no.	Immunization by one	Day 0,7 immunization by two doses	Day 0,14 immunization by two doses

						dose		
1200SU	20200303-1	10	10	10	20200303-5	10	10	10
600 SU	20200303-2	10	10	10	20200303-6	10	10	10
300SU	20200303-3	10	10	10	20200303-7	10	10	10
150 SU	20200303-4	10	10	10	/	/	/	/

- Determination of immunization dosage and schedules of COVID-19 Vaccine

Mouse test groups: 4 dose groups with antigen content of 300SU/0.5ml, 600SU/0.5ml, 1200SU/0.5ml and 2400SU/0.5ml (corresponding lot no. as 20200213-1~4) were for intraperitoneal immunization of mice as per 10 mice/group and 0.5ml/mouse by emergency and routine immunization schedules.

Rat test groups: 4 dose groups with antigen content of 300SU/0.5ml, 600SU/0.5ml, 1200SU/0.5ml and 2400SU/0.5ml (corresponding lot no. as 20200213-1~4) were for intramuscular immunization of rats as per 5 rats/group and 0.5ml/rat by emergency and routine immunization schedules. Vaccine diluent was also provided as negative control. The immunization and blood sampling are shown in Table 3.

Table 3 Research Design for Immunization Dosage and Schedules of COVID-19 Vaccine

Immunization Procedure	Immunization Procedure	Blood sampling time	Qty.
Emergency immunization schedule	Day 0 immunization	Blood collection on day 7, 14, 21, 28, 35, 42	10 mice and 5 rats
	Day 0,7 immunization	Blood collection on day 14, 21, 28, 35, 42	10 mice and 5 rats
	Day 0,3,7 immunization	Blood collection on day 7, 14, 21, 28, 35, 42	10 mice and 5 rats
Routine immunization schedule	Day 0,14 immunization	Blood collection on day 21, 28, 35, 42	10 mice and 5 rats
	Day 0,14,28 immunization	Blood collection on day 35, 42	10 mice and 5 rats

The proposed dosage of COVID-19 Vaccine for the Clinical Trial was determined by analyzing the immunization dosage, neutralizing antibody titer and enzyme labelled antibody titer. At the same time, the immunization effects of one dose, two doses and three doses were compared to determine the immunization schedule.

Research results:

- Determination of aluminum adsorption and non-aluminum adsorption

processes for the vaccine

After intraperitoneal immunization of mice by one dose of COVID-19 Vaccine with and without aluminum adjuvant, a certain level of anti-SARS-CoV-2 enzyme labelled antibody could be produced in the mice on D7 after primary immunization. The antibody level of 1200SU/0.5ml vaccine without aluminum adjuvant was comparable to that of 300SU/0.5ml vaccine with aluminum adjuvant. The immunogenicity of the vaccine with aluminum adjuvant was obviously better than that of the vaccine without aluminum adjuvant.

- Determination of immunization dosage and schedules of COVID-19 Vaccine

(1) For the same immunization schedule, different dose groups were for immunization of the same species of animals, and the neutralizing antibody titer was detected at the same blood collection point. There was a good dose-effect relationship between the immunization dosage and the neutralizing antibody titer produced.

(2) For the same dose groups, different immunization schedules (one dose, two doses and three doses) were used for immunization of the same species of animals, and the enzyme labelled antibody titer was detected at the same blood collection point. The immunization effect using the schedules of two and three doses on mice was lower than that using the schedule of one dose. The immunization effect using the schedules of two and three doses on rats was higher than that using the schedule of one dose. The immunization effect using the schedules of two and three doses was equivalent due to the short interval among three doses.

(3) For the same dose groups and the immunization schedule of two doses (day 0,7 and day 0,14) at different time points, the enzyme labelled antibody level of the day 0,14 immunization schedule was one order of magnitude higher than that of the day 0,7 immunization schedule on D21, indicating that the interval of more than 14 days is required between two doses in the Clinical Trial.

(4) For different dose groups and the same immunization schedule of two doses, the neutralizing antibody levels under 1200SU and 2400SU were basically the same.

Research conclusion: the formulation of aluminum adjuvant was selected, the dosages determined to be used for the Clinical Research were 300SU/dose, 600SU/dose

and 1200SU/dose, and the immunization schedule of two doses was selected.

4.3 Challenge Protection Research

Test objectives: to evaluate the animal protective effect of COVID-19 Vaccine under challenge of SARS-CoV-2 after immunizing animals with COVID-19 Vaccine according to different immunization schedules and dosages, and evaluate the existence of Antibody Dependent Enhancement (ADE), so as to provide animal experimental data for clinical research and application.

Experimental design: rhesus monkeys were immunized by COVID-19 Vaccine according to different immunization schedules and dosages, novel coronavirus seed was used to challenge the animals 21~42 days after the first immunization, the protective effect of the vaccine was evaluated according to the results of observation of clinical symptoms, serum detection, viral load test and histopathological examination of the rhesus monkeys, and the existence of ADE under different antibody levels was observed, with the research design shown in the table below:

Table 4 Experimental Design of Challenge Protection Effect

Group S/N	Group	Immunization schedule (day)	Sample dosage	Number of days of challenge after first immunization (day)	Number of days of euthanasia (after challenge)	Qty. of animals
1	3 doses-vaccine	0,7,14	High dose (1200SU/0.5ml)	23	7	4
			Medium dose (600SU/0.5ml)	22	7	4
2	3 doses-adjuvant	0,7,14	/	21	7	2
3	Model group	/	/	21	7	2
4	2 doses-vaccine	0,14	High dose (1200SU/0.5ml)	23	7	4
			Medium dose (600SU/0.5ml)	22	7	4

2-dose test results: no significant rise in body temperature was seen in the model group after the challenge, and high levels of virus were detected in throat swabs, anal swabs and lung tissue. Lung tissue showed severe interstitial pneumonia lesions. There was no significant difference between the adjuvant group and the model group; compared with the model group, 2 (n=4) had a body temperature over 40°C, 3 (n=4)

tested negative for throat swab virus on Day 3, Day 5 and Day 7 after challenge, 4 (n=4) tested negative for lung tissue virus on Day 7 after challenge, and all 4 showed mild interstitial pneumonia in the medium-dose group, suggesting that the medium-dose vaccine had a significant protective effect, and no ADE was observed; in the high-dose group compared with the model group, there was no abnormal body temperature, and 3 (n=4) tested negative for throat swab virus on Day 3, Day 5 and Day 7 after challenge. 4 (n=4) tested negative for lung tissue virus on Day 7 after challenge, and all 4 showed mild interstitial pneumonia, suggesting that the medium-dose vaccine had a significant protective effect, and no ADE was observed.

The changes of antibody levels in each group of rhesus monkeys are shown in Table 5. Based on the results of immune protection in the medium-dose group challenge suggested that neutralizing antibody titers greater than or equal to 1:48 after 2 doses of immunization had significant protective effect.

Table 5 Changes in Antibody Levels of Rhesus Monkeys in Each Group after COVID-19 Vaccination

	Animal No.	Day 0 after immunization	Day 7 after immunization	Day 14 after immunization	Day 21 after immunization	Day 3 after challenge	Day 5 after challenge	Day 7 after challenge
Medium-dose group	K21	<8	<8	4	64	64	48	256
	K22	<8	<8	4	128	48	64	128
	K23	<8	<8	6	48	32	96	1024
	K24	<8	<8	32	64	256	128	1024
GMT	/	/	/	7.4	70.8	70.8	78.4	430.5
High-dose group	K17	<8	<8	16	128	1024	512	512
	K18	<8	<8	16	256	256	512	512
	K19	<8	<8	4	96	512	1024	512
	K20	<8	<8	<4	64	192	256	1024
GMT	/	/	/	6.7	119.1	400.7	512.0	608.9
Adjuvant group	K9	<8	<8	<4	<4	<4	4	8
	K10	<8	<8	<4	<4	<4	<4	<8
GMT	/	/	/	/	/	/	/	/
Model group	K15	<8	<8	<4	<4	<4	6	12
	K16	<8	<8	<4	<4	<4	8	8
GMT	/	/	/	/	/	/	6.9	9.8

3-dose test results: no significant rise in body temperature was observed in the animals of the model group after challenge, and no abnormalities were observed in the adjuvant, medium-dose and high-dose groups in body temperature. WBC decreased and LYMPH% increased after all groups of animals were infected, and there was no

significant difference between the medium/high-dose groups and the model group. Blood biochemical tests on all the groups of animals on D0 and D14 after immunization and when the animals were put to death showed that all the indexes were within the normal range. High levels of virus were detected in throat swabs, anal swabs and lung tissue for the model group. In the medium-dose group, the average level of virus in throat and anal swabs decreased on Day 7 after challenge compared with the model group; in the high-dose group, throat and anal swabs were tested negative for virus on Day 7 after challenge; 3 (n=4) in the medium-dose group were tested negative for virus in lung tissue on Day 7 after challenge, and all 4 in the high-dose group were tested negative for virus in lung tissue on Day 7 after challenge.

Both the model and adjuvant groups were negative for neutralizing antibodies on Day 21 after immunization. In the medium-dose group, the neutralizing antibody GMT was 1:61.3 and reached 1:400.7 on Day 7 after challenge. In the high-dose group, the neutralizing antibody GMT was 1:50.1 and reached 1:145 on Day 7 after challenge, as shown in Table 6.

Table 6 Changes in Antibody Levels of Rhesus Monkeys in Each Group after COVID-19 Vaccination

	Animal No.	Day 0 after immunization	Day 7 after immunization	Day 14 after immunization	Day 21 after immunization	Day 3 after challenge	Day 5 after challenge	Day 7 after challenge
**Medium-dose group**	K5	<8	<8	6	64	32	384	1024
	K6	<8	<8	4	24	32	64	512
	K7	<8	<8	48	384	128	512	768
	K8	<8	<8	6	24	32	64	64
**GMT**		/	/	9.1	61.3	45.3	168.5	400.7
**High-dose group**	K1	<8	<8	12	48	24	96	256
	K2	<8	<8	16	64	96	512	384
	K3	<8	<8	6	32	24	48	96
	K4	<8	<8	6	64	16	48	48
**GMT**		/	/	9.1	50.1	30.7	103.2	145.9
**Adjuvant group**	K9	<8	<8	<4	<4	<4	4	8
	K10	<8	<8	<4	<4	<4	<4	<8
**GMT**		/	/	/	/	/	/	/
**Model group**	K15	<8	<8	<4	<4	<4	6	12
	K16	<8	<8	<4	<4	<4	8	8
**GMT**		/	/	/	/	/	6.9	9.8

Pathological findings of some animals are detailed in Fig. 1-6.

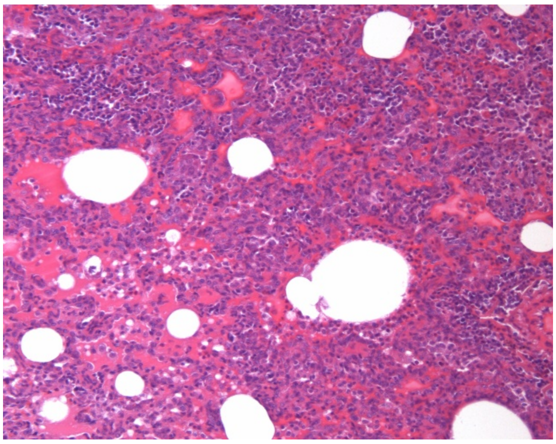

Fig. 1 Model Group K15 Minor Lobe of Right Lung Severe Interstitial Pneumonia H.E.×100

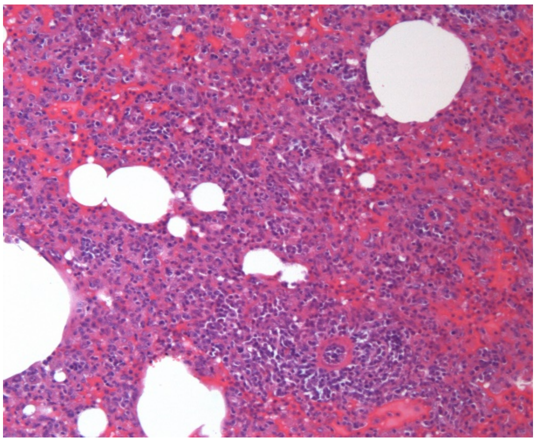

Fig. 2 Adjuvant Group K10 Middle Lobe of Right Lung Severe Interstitial Pneumonia H.E.×100

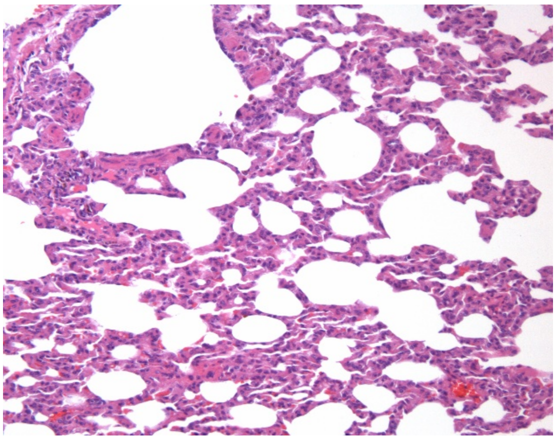

Fig. 3 Medium-dose Group K21 Superior Lobe of Right Lung Mild Interstitial Pneumonia H.E.×100

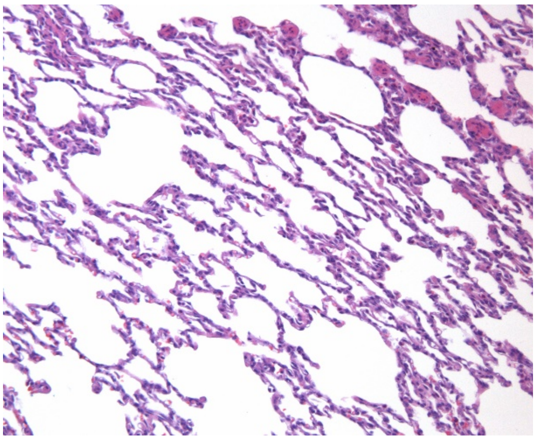

Fig. 4 Medium-dose Group K22 Inferior Lobe of Left Lung No Abnormality Seen H.E.×100

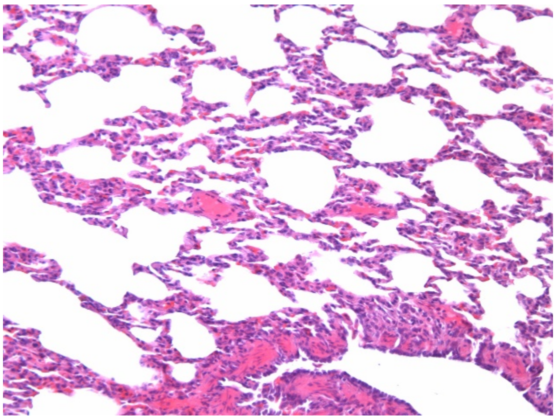

Fig. 5 High-dose Group K17 Inferior Lobe of Right Lung Mild Interstitial Pneumonia H.E.×100

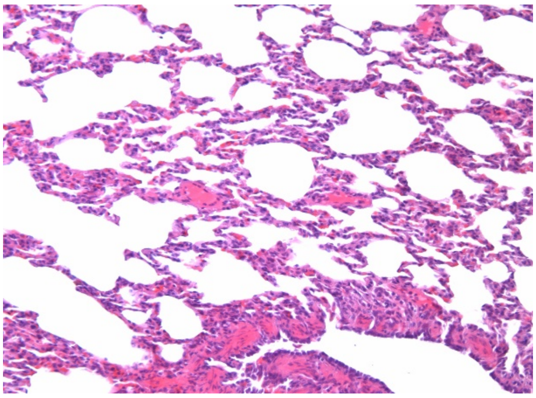

Fig. 6 High-dose Group K19 Inferior Lobe of Left Lung No Abnormality Seen H.E.×100

Test conclusions: The COVID-19 vaccine had apparently protective effects, and no ADE phenomenon was observed.

4.4 Cross Neutralizing Research

A cross neutralizing test of different viruses was conducted on the serum of

patients infected with SARS-CoV-2 in the acute phase and in the recovery phase. The preliminary results showed that the overall neutralizing antibody level in the serum in the acute phase was not high, and a certain proportion of serum neutralizing antibody was negative. The overall neutralizing antibody level in the serum in the recovery phase was higher than that in the acute phase, and the preliminary data showed that 100% serum neutralizing antibody in the recovery phase was positive, which could suggest that the neutralizing antibody played a certain protective role in the recovery process of patients.

The neutralizing antibody titer of the animal serum of the monkeys immunized by vaccine was also preliminarily detected, the results of which showed that good neutralizing antibody titer could be produced by the vaccine when the non-vaccine strain virus was used for detecting the neutralizing antibody, and it had a good dose-effect relationship with different immunization dosages and schedules.

The cross neutralizing test will be continued.

5. Preliminary Clinical Trial

5.1 Safety Evaluation

(1) Phase I clinical trial

Adverse event

In phase I clinical trial, a total of 143 subjects aged 18~59 years were included into the SS set, including 72 in Day 0, Day 14 emergency immunization schedule and 71 in Day 0, Day 28 routine immunization schedule. According to the analysis results, the overall incidence of adverse reaction was 25.00%, and the incidence of adverse reaction was 29.17%, 37.50% and 8.33% respectively in test vaccine group, high-dose group and placebo group from the vaccination to Day 28 after full-course immunization, and the difference in incidence between groups was not statistically significant. Adverse reactions are mainly systemic diseases and various reactions at the administration site according to system organ classes (SOC), mainly manifested as pain at the inoculation site, followed by weakness. The adverse reaction level was mainly grade 1, with an incidence rate of 25.00%, and no Grade 2 adverse reaction occurred.

The incidence rate of Grade 3 adverse reaction was only 1.39%.

The overall incidence of adverse reaction was 14.08% under routine schedule, and the incidence of adverse reaction was 12.50%, 16.67% and 13.04% respectively in medium-dose group, high-dose group and placebo group of test vaccine from the vaccination to Day 28 after full-course immunization, without statistically significant differences between groups Adverse reactions are mainly systemic diseases and various reactions at the administration site, mainly manifested as pain at the inoculation site and the difference in the incidence of each adverse reaction between groups was not statistically significant. All adverse reactions were Grade 1.

In the phase I clinical trial, from the beginning of vaccination to 6 months after full-course immunization, the overall incidence of serious adverse events under the emergency schedule was 1.39% (1/72), i.e., one subject in the placebo group had one serious adverse event, namely colorectal polyp; the overall incidence of serious adverse events under the routine schedule was 1.41% (1/71), i.e., one subject in the high-dose group had 2 serious adverse events, namely goiter and papillary thyroid carcinoma. All of the above adverse events occurred on Day 28 after two doses of vaccine and were not related to vaccination.

Laboratory inspection

For the emergency immunization schedule, the incidence of abnormalities in laboratory indicators of clinical significance was 8.33%, 8.33% and 4.17% respectively in medium/high-dose groups of test vaccine and placebo group on Day 3 after each dose of vaccine, dominant by Grade 1. For the routine immunization schedule, the incidence of abnormalities in laboratory indicators of clinical significance was 8.33%, 8.33% and 4.35% respectively in medium/high-dose groups of test vaccine and placebo group on Day 3 after each dose of vaccine, dominant by Grade 1.

Inflammatory factor

For the first dose of the emergency immunization schedule, the average level of TNF-α in the high-dose group on Day 7 after vaccination was 2 times that before

vaccination (P<0.0006); for the second dose, the average level of IL-2 in the high-dose group on Day 7 after vaccination was 0.5 times that before vaccination (P=0.0102), and the average level of TNF-α was about 1.4 times that before vaccination (P=0.0060); for the second dose of the routine immunization schedule, the average level of serum TNF-α in the high-dose group on Day 7 after vaccination was about 0.5 times that before vaccination (P<0.0001), and the actual changes in all the above inflammatory factors were small, and neither substantial increase in serum inflammatory factors nor signals related to immunopathological reactions were found.

(2) Phase II clinical trial

In phase II clinical trial, a total of 600 subjects aged 18~59 years (C001-C300 & D001~D300) were included into the SS set, including 300 in emergency immunization schedule (Day 0, Day 14, Day 42 or Day 0, Day 14) and 300 in routine immunization schedule (Day 0, Day 28, Day 56 or Day 0, Day 28).

The overall incidence of adverse reaction was 31.67% under emergency schedule (C001-C300), and the incidence of adverse reaction was 33.33%, 35.00% and 21.67% respectively in medium/high-dose groups of test vaccine and placebo group from the vaccination to Day 28 after two doses, without statistically significant differences between groups. Adverse reactions are mainly systemic diseases and various reactions at the administration site according to system organ classes (SOC), mainly manifested as pain at the inoculation site, followed by weakness. The incidence of medium/high-dose groups of test vaccine and placebo group was 20.83%, 25.83% and 10.00% respectively (high-dose group>medium-dose group > placebo group), and the differences were statically significant. The difference in the incidence of the adverse reactions other than pain at the inoculation site between groups was not statistically significant. The overall adverse reaction was mainly Grade 1, without Grade 3 adverse reaction.

The overall incidence of adverse reaction was 19.00% under routine schedule (D001-D300), and the incidence of adverse reaction was 19.17%, 19.17% and18.33% respectively in medium/high-dose groups of test vaccine and placebo group from the

vaccination to Day 28 after two doses, without statistically significant differences between groups. Adverse reactions are mainly systemic diseases and various reactions at the administration site according to system organ classes (SOC), mainly manifested as pain at the inoculation site, followed by weakness. The incidence of medium/high-dose groups of test vaccine and placebo group was 17.50%, 16.67% and 16.67% respectively, and the differences in various adverse reactions between groups were not statistically significant. The adverse reaction was mainly Grade 1, and the incidence was 19.17%, 19.17% and 18.33% respectively in medium/high-dose groups of test vaccine and placebo group, without Grade 3 adverse reaction.

In the phase II clinical trial, from the beginning of vaccination to 6 months after full-course immunization, the overall incidence of serious adverse events under day 0, 14 emergency schedule (C151-C300) was 2.67% (4/150); the incidence of serious adverse events in medium-/high-dose groups was 3.33% (2/60), and that of the placebo group was 0%. In the medium-dose group, 3 serious adverse events occurred in 2 subjects, including 1 subject with hand fracture and nail injury and 1 subject with hemorrhoids; in the high-dose group, 2 adverse events occurred in 2 subjects, including 1 subject with soft tissue injury and 1 subject with glandular cystitis; 0, the overall incidence of serious adverse events was 0.67% under day 0, 28 routine schedule (D151-D300), and only one subject in the medium-dose group had a serious adverse event, namely protrusion of intervertebral disc. All of the above adverse events were not related to vaccination.

5.2 Immunogenicity Evaluation

5.2.1 Immunogenicity of two doses

The results of phase I clinical trial showed that, under the emergency immunization schedule, the positive conversion rate was 45.83%, 50.00% and 0% respectively and neutralizing antibody GMT was 5.6, 7.7 and 2.0 respectively in test vaccine medium-dose group, high-dose group and placebo group on Day 14 after two-dose inoculation; under the routine immunization schedule, the positive conversion rate was 83.33%, 79.17% and 4.35% respectively and neutralizing antibody GMT was 19.0,

29.6 and 2.2 respectively in test vaccine medium-dose group, high-dose group and placebo group on Day 28 after full-course vaccination.

The results of phase II clinical trial showed that, under the emergency immunization schedule (C001-C300), the positive conversion rate was 92.37%, 98.32% and 3.33% respectively and neutralizing antibody GMT was 27.6, 34.5 and 2.3 respectively in test vaccine medium-dose group, high-dose group and placebo group on Day 14 after two-dose inoculation; under the routine immunization schedule (D001-D300), the positive conversion rate was 97.44%, 100% and 0% respectively and neutralizing antibody GMT was 44.1, 65.4 and 2.0 respectively in test vaccine medium-dose group, high-dose group and placebo group on Day 28 after two-dose inoculation, suggesting that both the emergency immunization schedule and the routine immunization schedule of COVID-19 vaccine had good immunogenicity. On Day 14 after two doses under emergency immunization schedule (C001-C300), the positive conversion rate in the high-dose group was 98.32%, slightly higher than that in the middle-dose group (92.37%) (P=0.0296), and GMT 34.5 in the high-dose group was comparable to 27.6 in the middle-dose group (P=0.1051); on Day 28 after two doses under routine immunization schedule (D001-D300), the positive conversion rate was 100% in the high-dose group and 97.44% in the middle-dose group (P=0.1218), and GMT was 65.4 in the high-dose group, higher than that in the middle-dose group (44.1) (P=0.0006). In summary, the difference in neutralizing antibody GMT after immunization was less than 1.5 times between the medium and high dose groups, and the positive conversion rate was more than 90% in both groups.

The immunogenicity from phase II was significantly superior to that from phase I because the manufacturing process of vaccine for phase II clinical trial was optimized based on the vaccine for phase I clinical trial. The cell factory process was upgraded into bioreactor process, so the immunogenicity of the vaccine was significantly improved.

5.2.2 Immunization persistence of two doses

The results of the phase II clinical trial 6 months after immunization under day 0,

14 two-dose primary immunization schedule (C151-C300) showed that the positive rate of the medium/high-dose groups and the placebo group were 16.95%, 24.14% and 0% respectively, and the GMT was 4.1, 4.8 and 2.0 respectively. The difference in the positive rate between the groups was statistically significant (P=0.0056), and the positive rate in the medium/high-dose groups were comparable, with no statistically significant difference (P=0.3356); the GMT difference was statistically significant (P<0.0001) in each group, and the difference was not statistically significant (P=0.3574) in the medium/high-dose groups with comparable GMT. The positive rate of neutralizing antibody and GMT change trends within 6 months after immunization under day 0, 14 two-dose primary immunization schedule are shown in Fig. 7~8.

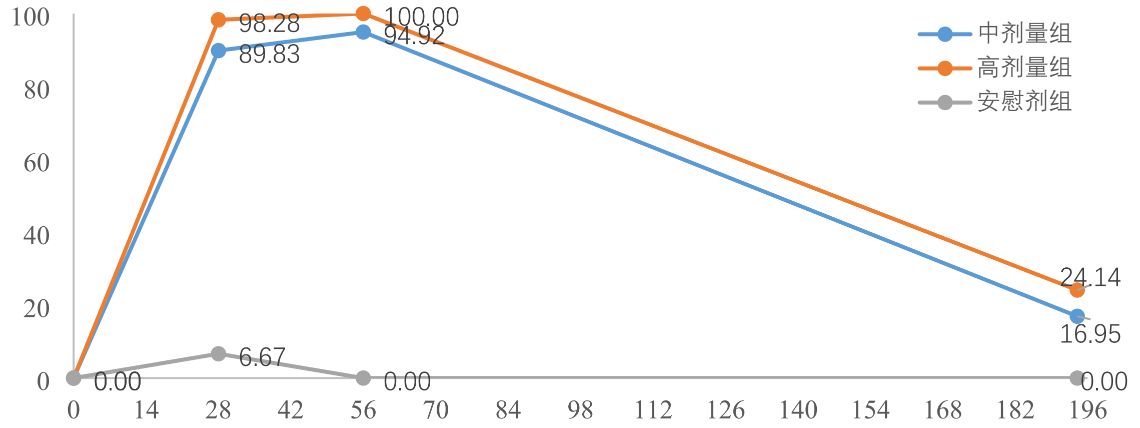

Fig. 7 Positive Rate of Neutralizing Antibody at Different Time Points of Day 0, Day 14 Two-dose Basic Immunization Schedule for Phase II Clinic Trial of COVID-19 Vaccine for Adults

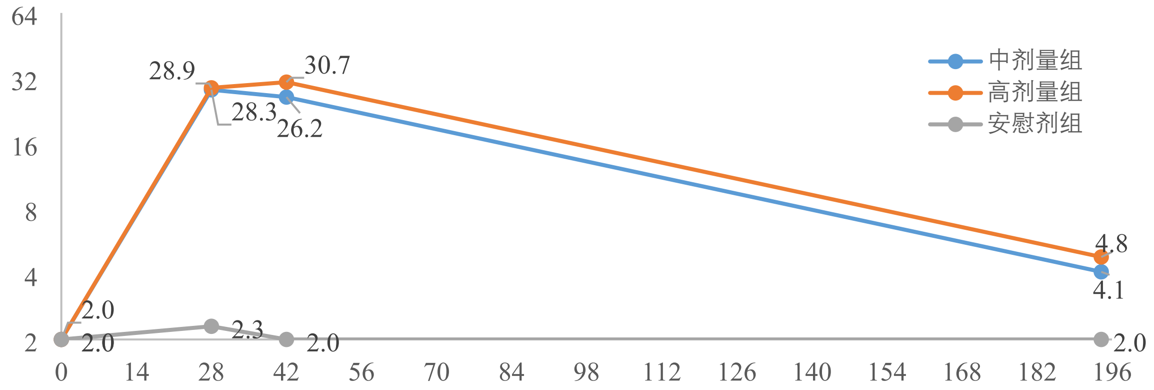

Fig. 8 GMT of Neutralizing Antibody at Different Time Points of Day 0, Day 14 Two-dose Basic Immunization Schedule for Phase II Clinic Trial of COVID-19 Vaccine for Adults

The results of the phase II clinical trial 6 months after immunization under Day 0,

Day 28 two-dose primary immunization schedule (D151-D300) showed that the positive rate of the medium-/high-dose groups and the placebo group were 35.19%, 46.43% and 0% respectively, and the GMT was 6.7, 7.1 and 2.0 respectively. The difference in the positive rate between the groups was statistically significant (P<0.0001), and the positive rate in the medium-/high-dose groups were comparable, with no statistically significant difference (P=0.2305); the GMT difference was statistically significant (P<0.0001) in each group, and the difference was not statistically significant (P=0.7579) in the medium-/high-dose groups with comparable GMT. The positive rate of neutralizing antibody and GMT change trends within 6 months after immunization under day 0, 28 two-dose primary immunization schedule are shown in Fig. 9~10.

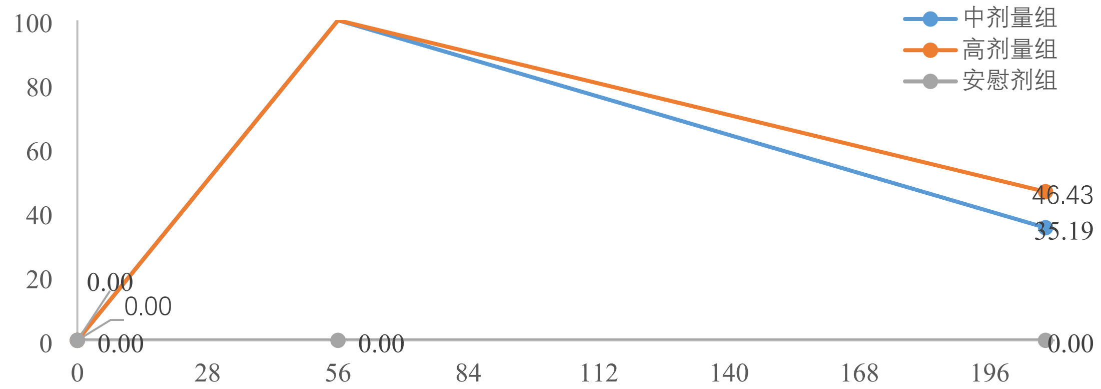

Fig. 9 Positive Rate of Neutralizing Antibody at Different Time Points of Day 0, Day **28 Two-dose Basic Immunization Schedule for Phase II Clinic Trial of COVID-19 Vaccine for Adults**

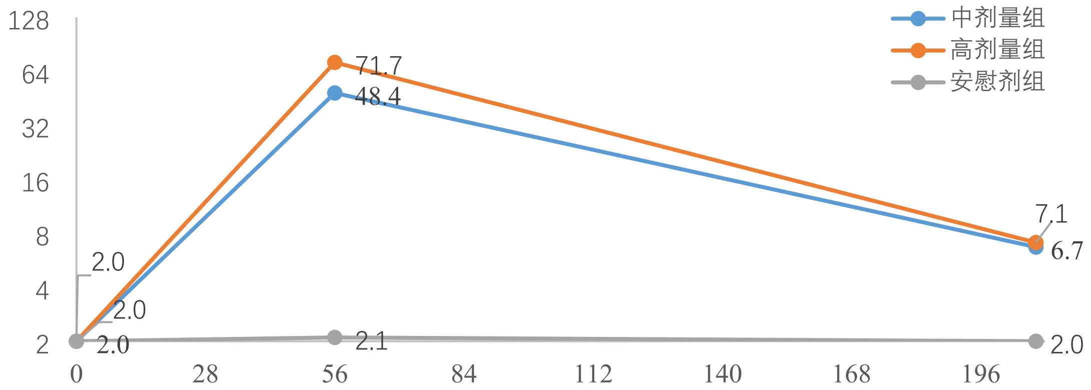

Fig. 10 GMT of Neutralizing Antibody at Different Time Points of Day 0, Day 28 Two-dose Basic Immunization Schedule for Phase II Clinic Trial of COVID-19 Vaccine for Adults

5.2.2 3 Immunogenicity

The immunogenicity results of the phase II clinical trial under Day 0, Day 14, Day

42 three-dose primary immunization schedule showed that all the subjects were negative for neutralizing antibodies before immunization, indicating the baseline antibody level was balanced and comparable. The positive conversion rate/positive rate was 94.83%, 93.22% and 98.15% respectively in the medium-dose group on day 14 after the second dose of immunization, on Day 28 after the second dose of immunization and on day 28 after the third dose of immunization, with the GMT of 27.0, 22.2 and 45.8 respectively; the positive conversion rate/positive rate was 98.33%, 98.33% and 98.28% in the high-dose group, with the GMT of 40.8, 29.1 and 74.2 respectively. The positive conversion rate/positive rate of neutralizing antibody in the medium/high-dose groups was comparable, and the differences were not statistically significant (P=1.0000); the GMT of the high-dose group was higher than the medium-dose group, and the differences were not statistically significant (P=0.0052).

Table 7 Immunogenicity of Phase II Clinic Trial of COVID-19 Vaccine for Adults under Day 0, Day 14, Day 42 Three-dose Basic Schedule (Neutralizing Antibody)

Time	Index	Medium-dose group	High-dose group	Placebo group	P value (3 groups)	P value (Medium vs high)
Before immunization	N	54	58	26		
	Positive rate n (%)	0(0.00)	0(0.00)	0(0.00)	1.0000	1.0000
	GMT	2.0	2.0	2.0	NA	NA
Second dose Day 14 after immunization	N	58	60	30		
	Positive rate n (%)	55 (94.83)	59 (98.33)	0 (0.00)	**<0.0001**	0.3601
	Positive conversion rate n (%)	55 (94.83)	59 (98.33)	0 (0.00)	**<0.0001**	0.3601
	GMT	27.0	40.8	2.2	<0.0001	0.0272
	GMI	13.5	20.4	1.1	<0.0001	0.0272
Second dose Day 28 after immunization	N	59	60	30		
	Positive rate n (%)	55 (93.22)	59 (98.33)	0 (0.00)	<0.0001	0.2068
	Positive conversion rate n (%)	55 (93.22)	59 (98.33)	0 (0.00)	<0.0001	0.2068
	GMT	22.2	29.1	2.0	<0.0001	0.0762
	GMI	11.1	14.6	1.0	<0.0001	0.0762
Third dosage Day 28 after immunization	N	54	58	26		
	Positive rate n (%)	53 (98.15)	57 (98.28)	0 (0.00)	<0.0001	1.0000
	Positive conversion rate n (%)	53 (98.15)	57 (98.28)	0 (0.00)	<0.0001	1.0000
	GMT	45.8	74.2	2.0	<0.0001	0.0052
	GMI	22.9	37.1	1.0	<0.0001	0.0052

The immunogenicity results of the phase II clinical trial under Day 0, Day 28, Day 56 three-dose primary immunization schedule showed that all the subjects were negative for neutralizing antibodies before immunization, indicating the baseline

antibody level was balanced and comparable. The positive conversion rate/positive rate of neutralizing antibody was 94.92% and 98.11% respectively in the medium-dose group on Day 28 after the second dose of immunization and on Day 28 after the third dose of immunization, with the GMT of 39.6 and 49.7 respectively; the positive conversion rate/positive rate was 100% in the high-dose group, with the GMT of 58.4 and 51.9 respectively. The positive conversion rate/positive rate of neutralizing antibody in the medium/high-dose groups was comparable on Day 28 after third dose, and the differences were not statistically significant (P=1.0000); the GMT of the medium/high-dose groups was comparable, and the differences were not statistically significant (P=0.7794).

Table 8 Immunogenicity of Phase II Clinic Trial of COVID-19 Vaccine for Adults under Day 0, Day 28, Day 56 Three-dose Basic Immunization Schedule (Neutralizing Antibody)

Time	Index	Medium-dose group	High-dose group	Placebo group	P value (3 groups)	P value (Medium vs high)
Before immunization	N	53	48	25		
	Positive rate n (%)	0(0.00)	0(0.00)	0(0.00)	1.0000	1.0000
	GMT	2.0	2.0	2.0	NA	NA
Second dose Day 28 after immunization	N	59	60	30		
	Positive rate n (%)	56 (94.92)	60 (100.00)	0 (0.00)	<0.0001	0.1187
	Positive conversion rate n (%)	56 (94.92)	60 (100.00)	0 (0.00)	<0.0001	0.1187
	GMT	39.6	58.4	2.0	<0.0001	0.0292
	GMI	19.8	29.2	1.0	<0.0001	0.0292
Third dosage Day 28 after immunization	N	53	48	25		
	Positive rate n (%)	52 (98.11)	48 (100.00)	0 (0.00)	<0.0001	1.0000
	Positive conversion rate n (%)	52 (98.11)	48 (100.00)	0 (0.00)	<0.0001	1.0000
	GMT	49.7	51.9	2.0	<0.0001	0.7794
	GMI	24.8	26.0	1.0	<0.0001	0.7794

5.3 Conclusion

COVID-19 Vaccine, Inactivated produced by SINOVAC has good safety and immunogenicity, and can produce antibodies rapidly after two doses of vaccination according to Day 0, Day 14 or Day 0, Day 28 schedule. However, the antibody level have dropped to a low level 6 months after two doses of vaccination. The antibody level after three doses of primary immunization on Day 0, Day 14, Day 42 or Day 0, Day 28, Day 56 were significantly higher than that after two doses of primary immunization, for which evidence of immunization persistence has not been obtained. Besides, the

cellular immune response of the COVID-19 Vaccine, Inactivated remains to be further investigated. No obvious change in the inflammatory factor before and after inoculation was caused; no signal related to immunopathological effect was observed.

According to the CDE's requirements for conditional marketing authorization of COVID-19 vaccine: "If the results of the subsequent clinical trials suggest that the existing immunization schedules and doses are not optimal, research on optimization of immunization schedules and doses should be continued". Considering that the neutralizing antibody level dropped to a low level 6 months after two doses of primary immunization, it was decided to give one dose of booster immunization 6 months after primary immunization and further investigate the immunization effect of the booster immunization schedule, thus providing a basis for the optimal immunization strategy for the subjects who had got two doses of primary immunization (C151-C300 and D151-D300) on Day 0, Day 14 and Day 0, Day 28 in Phase II of this study The above additional studies have been approved by DMC.

6 Introduction of Product Features

6.1 Preparation Technology and Formulation of the Vaccine

The COVID-19 Vaccine, Inactivated is prepared by vaccinating African green monkey kidney cell (referred to as “Vero Cell”) with novel coronavirus (CZ02 strain) through the culture, harvesting of virus solution, virus inactivation, concentration, purification and adsorption of aluminum hydroxide. It is milky white suspension liquid, which may be stratified due to precipitation and is easy to shake off. Its main component is inactivated novel coronavirus (SARS-CoV-2), and its excipients are aluminum hydroxide, disodium hydrogen phosphate, sodium dihydrogen phosphate, sodium chloride, etc. It contains no preservatives. It is packed in penicillin bottles or pre-filled syringes, each with 0.5ml. The vaccination can induce the body to produce the immunity against SARS-CoV-2 to prevent the disease caused by SARS-CoV-2 infection.

The test vaccine is prepared by Sinovac Research & Development Co., Ltd. and has been verified by National Institutes for Food and Drug Control as conforming to

the requirements of the *Regulations for Production and Verification of COVID-19 Vaccine, Inactivated (Draft)*. The vaccine is of injection type, with the specification of 0.5ml/dose. It contains respectively 300SU/0.5ml, 600SU/0.5ml and 1200SU/0.5ml SARS-CoV-2 antigen.

6.2 Vaccine Stability

The thermal accelerated stability study at 25±1°C on Day 56 and at 37±1°C on Day 42 has been completed for 6 batches of final products produced by the bioreactor. When stored at 25±1°C, the antigen content after the dissociation at different time points within 42 days (inclusive) met the quality standard, while the antigen content after the dissociation on Day 56 was lower than the quality standard, then the test was terminated; when stored at 37±1°C, the antigen content after the dissociation at different time points within 28 days (inclusive) met the quality standard, while the antigen content after the dissociation at the monitoring point on Day 42 was lower than 50% of the labelled amount for some batches, then the test was terminated.

The long-term stability observation at 2-8°C for 6 months has been completed for 3 batches of final products produced by the cell factory and no obvious reduction was observed in the antigen content after the dissociation of different batches of final products of COVID-19 vaccine. The long-term stability observation for 6 months has been completed for 3 of 9 batches of final products produced by the bioreactor, and no obvious reduction was observed in the test data. Another 6 batches received 3-month long-term stability observation, no obvious reduction was observed in the antigen content after the dissociation of different batches of final products of COVID-19 vaccine.

According to the results of accelerated stability test, the validity period of vaccine is tentatively determined to be 3 years when stored at 2-8°C.

6.3 Control Vaccine

The Research applies placebo control, which is produced by Sinovac Research & Development Co., Ltd., with its component as aluminum hydroxide diluent and a trace

of milky white precipitated liquid. Its appearance is the same as that of the test vaccine.

It has been verified by National Institutes for Food and Drug Control as conforming to the requirements of the *Regulations for Production and Verification of COVID-19 Vaccine, Inactivated (Draft)*. The vaccine is of injection type, with the specification of 0.5ml/dose. It contains no SARS-CoV-2 antigen.

6.4 Transportation and Storage of Vaccine

The vaccine shall be kept and transported away from light at 2~8℃.

6.5 Inoculation Route and Procedure

The qualified subjects receive intramuscular injection at the lateral deltoid of upper arm, each with 0.5ml test vaccine or control vaccine for single dose. The subjects were inoculated according to emergency immunization schedule (Day 0, Day 14, Day 42 or Day 0, Day 14) or routine immunization schedule (Day 0, Day 28, Day 56 or Day 0, Day 28) as per 0.5ml/dose/times, and the vaccine was shook well before use. The subjects under Day 0, Day 14 and Day 0, Day 28 primary immunization schedules were given 1 dose of booster immunization 6 months after the second dose of vaccination.

6.6 Information of Test Product

Information of test product is shown in the following figure:

Table 9 Information of Test Product

Group	Vaccine name	Packaging	Antigen content	Manufacturer	Phase	Lot No.	Valid until
Medium-dose test Vaccine	COVID-19 Vaccine, Inactivated	Prefilled syringe	600SU/ 0.5ml	SINOVAC	Phase I	20200304	2023.02.28
					Phase II	20200308	2023.03.20
High-dose test Vaccine	COVID-19 Vaccine, Inactivated	Prefilled syringe	1200SU/ 0.5ml	SINOVAC	Phase I	20200310	2023.03.25
					Phase II	20200309	2023.03.21
Placebo Control	Aluminum hydroxide diluent	Prefilled syringe	0SU/ 0.5ml	SINOVAC	Phase I	2020022801	2023.02.27
					Phase II	2020022801	2023.02.27

6.7 Vaccine Packaging

The vaccine will be packed in a labelled box, with the label style shown as follows, and the rules of vaccine numbering on the label can be found in “8.4 Randomization and double-blinding”.

I/II Clinical Trial for COVID-19 Vaccine (Vero Cell), Inactivated<br>PRO-nCOV-1001<br>**A001**<br>For clinical trial only, stored at 2-8℃<br>Expiration date:

The packing box should be as follows:

I/II Clinical Trial for COVID-19 Vaccine (Vero Cell), Inactivated<br>PRO-nCOV-1001<br>Number segment:<br>For clinical trial only, stored at 2-8℃<br>Expiration date:

7 Purpose

To evaluate the safety and immunogenicity of COVID-19 Vaccine, Inactivated developed by SINOVAC for vaccination in adults.

7.1 Phase I Clinical Trial

To evaluate the safety, tolerance and primary immunogenicity of different doses of test vaccine for vaccination in adults according to different immunization schedules.

7.2 Phase II Clinical Trial

To evaluate the immunogenicity and safety of different doses of test vaccine for vaccination in adults according to different immunization schedules, and determine the appropriate dosage and vaccination schedule.

8 Test Design

8.1 Design

8.1.1 Overall design

The randomized, double-blinded and placebo-controlled design is applied.

8.1.2 Sample size and power of test

Phase I clinical trial: according to the *Technical Guidelines for Clinical Trial of Vaccines*[3] and *Provisions of Drug Registration*[1], Phase I clinical trial is a small-scale research (20~30 persons), focusing on the evaluation of vaccine safety. The total sample size of this phase of clinical trial is 144, with 72 for each immunization schedule.

The total number of subjects vaccinated with medium-does and high-dose test vaccine under each immunization schedule is 48. The number of subjects vaccinated with the test vaccine meets the requirement of phase I clinical trial.

Phase II clinical trial: according to the *Technical Guidelines for Clinical Trial of Vaccines*[3] and the *Provisions of Drug Registration*[1], Phase II clinical trial is to observe the immunization effect and safety of different doses of vaccine in the target population. The endpoint is to evaluate the immunogenicity and safety of the test vaccine. The number of cases in the test group is not less than 300. The total sample size of this phase of clinical trial is 600, the number of subjects vaccinated with medium-dose test vaccine, high-dose test vaccine and placebo is respectively 240, 240 and 120, and the number of cases in the test vaccine group is 480. The number of subjects vaccinated with the test vaccine meets the basic requirements of phase II clinical trial.

8.2 Endpoint

8.2.1 Endpoints of phase I trial

8.2.1.1 Primary endpoint

- Incidence of adverse reactions occurred from the beginning of the vaccination to 28 days after the whole-schedule vaccination.

8.2.1.2 Secondary endpoint

- Incidence of adverse reactions 0~7 days after each dose of vaccination;
- Incidence of abnormal laboratory indexes (blood routine test, blood biochemistry test and urine routine test) on the 3rd day after each dose of vaccination;
- Incidence of serious adverse event from the inoculation to 6 months after full-course vaccination;
- The seroconversion rate, seropositive rate, GMT, and GMI of neutralizing antibodies 7/14/21/28/42 days after the first dose of vaccination (emergency schedule);
- The seropositive rate of IgG and IgM antibodies 7/14/21/28/42 days after the first dose of vaccination (emergency schedule);

- The seroconversion rate, seropositive rate, GMT, and GMI of neutralizing antibodies 28/35/42/56 days after the first dose of vaccination (routine schedule);
- The seropositive rate of IgG and IgM antibodies 28/35/42/56 days after the first dose of vaccination (routine schedule).

8.2.1.3 Exploratory endpoint

- Positive rate of T cell response 14 days after vaccination (IFN-γ detection using Elispot);
- Positive rate and GMT of neutralizing antibody 6 months after full-course vaccination of test vaccine.
- Positive rate and GMT of IgG and IgM antibodies 6 months after full-course vaccination of test vaccine.
- The change of interleukin-6 (IL-6), interleukin-2 (IL-2) and tumor necrosis factor-α (TNF-α) in serum 7 days after each dose of vaccination.
- Positive rate of antinuclear antibody on Day 7/14/21/28/42/194 after the first dose of test vaccine (emergency immunization schedule);
- Positive rate of antinuclear antibody on Day 28/35/42/56/208 after the first dose of test vaccine (routine immunization schedule).

8.2.2 Endpoints of phase II trial

8.2.2.1 Primary endpoint

- Positive conversion rate of serum neutralizing antibody on Day 14 (emergency immunization schedule)/Day 28 (routine immunization schedule) after two doses of test vaccine;
- Incidence of adverse reaction on Day 0~28 (Day 0~14 for the first dose under emergency immunization schedule) after each dose;

8.2.2.2 Secondary endpoint

- positive rate, GMT and GMI of serum neutralizing antibody 14 days (emergency immunization schedule)/28 days (routine immunization schedule) after two doses of test vaccine;
- Positive conversion rate, positive rate, GMT and GMI of serum neutralizing

antibody on Day 28 after two dose of test vaccine under emergency immunization schedule;

- Positive conversion rate, positive rate, GMT and GMI of serum neutralizing antibody on Day 28 after three doses of test vaccine for primary immunization (only Day 0, Day 14, Day 42 and Day 0, Day 28, Day 56 schedules);
- Incidence of adverse reactions 0~7 days after each dose of vaccination;
- Incidence of serious adverse event from the inoculation to 6 months after full-course vaccination;

8.2.2.3 Exploratory endpoint

- Positive rate and GMT of neutralizing antibody 6 months after two doses of test vaccine (only Day 0, Day 14 or Day 0, Day 28 schedule);
- Positive rate and GMT of neutralizing antibody 12 months after three doses of test vaccine for primary immunization (only Day 0, Day 14, Day 42 and Day 0, Day 28, Day 56 schedules);
- Positive rate, GMT and GMI of serum neutralizing antibody on Day 14 (emergency immunization schedule)/Day 28 (routine immunization schedule) after booster inoculation of test vaccine (only Day 0, Day 14 and Day 0, Day 28 schedules);
- Positive rate and GMT of neutralizing antibody 6 months after booster immunization of test vaccine (only Day 0, Day 14 and Day 0, Day 28 schedules);
- Positive rate of antinuclear antibody on Day 28/42/70 after the first dose of test vaccine (Day 0, Day 14, Day 42 schedule);
- Positive rate of antinuclear antibody on Day 28/42/194 after the first dose of test vaccine (Day 0, Day 14 schedule);
- Positive rate of antinuclear antibody on day 56/84 after the first dose of test vaccine (Day 0, Day 28, Day 56 schedule);
- Positive rate of antinuclear antibody on day 56/208 after the first dose of test vaccine (Day 0, Day 28 schedule).

8.3 Study Plan

8.3.1 Phase I Study Plan

The clinical trial is a single-center, randomized, double-blind and placebo-controlled one. A total of 144 healthy adults aged 18-59 years are selected as the subjects. Upon informed consent, physical examination and screening based on the inclusion/exclusion criteria, the subjects are enrolled to receive different doses of the test vaccine or placebo according to different immunization schedules and meanwhile receive the test vaccine sequentially in accordance with the principle of medium dose to high dose. The purpose is to evaluate the safety, tolerance and primary immunogenicity of the test vaccine.

The subjects are enrolled according to the emergency immunization schedule of day 0,14 and the routine immunization schedule of day 0,28. A total of 72 subjects are enrolled for each immunization schedule in stages of medium and high doses respectively, each with 36 subjects who are vaccinated by the test vaccine or placebo respectively at the ratio of 2:1. According to the occurrence of the solicited and non-solicited adverse events, and of the abnormal indexes of blood routine examination, blood biochemistry and routine urine test, the vaccination in the stage of high dose may be conducted only 0~7 days after the first dose in the stage of medium dose is vaccinated when the safety observation is completed and the safety is confirmed.

The immediate reaction within 30 minutes after each dose of vaccination is observed, the local and systemic solicited adverse events within 0~7 days and the non-solicited adverse events from the beginning of the vaccination to 28 days after the whole-schedule vaccination are collected, and the SAE monitoring from the beginning of the vaccination to 6 months after the whole-schedule vaccination is completed.

The blood of volunteers was sampled at different times before and after immunization to test the blood routine, blood biochemistry, urine routine, serum inflammatory factors and anti-nuclear antibodies and evaluate the safety of the vaccine; test the serum neutralizing antibody, IgG and IgM antibodies and IFN-γ secretory reaction of specific T cells and evaluate the immunogenicity and immunization persistence of the vaccine.

See Table 10 for details on the Phase I clinical trial study plan.

Table 10 Phase I Clinical Trial Study Plan

Schedule (day)	Medium dose	High dose	Placebo	Total	Blood sampling time (day)	Antibody test/T cell reaction (day)	Blood routine examination, blood biochemistry and routine urine test (day)	Inflammatory factor (day)
0,14	24		12	36	0(-7),3,7,14,17, 21,28, 42,194¶	0*,7,14*,21, 28*, 42,194	0(-7),3, 14,17	0,7,14,21
		24	12	36	0(-7),3,7,14,17, 21,28, 42,194¶	0*,7,14*,21, 28*, 42,194	0(-7),3,14,17	0,7,14,21
0,28	24		12	36	0(-7),3,7,28,31, 35,42,56,208¶	0*,28*,35,42*, 56,208	0(-7),3,28,31	0,7,28,35
		24	12	36	0(-7),3,7,28,31, 35,42,56,208¶	0*,28*,35,42*, 56,208	0(-7),3,28,31	0,7,28,35
**Total**	**48**	**48**	**48**	**144**				

Remarks: * Test time-point of specific T cells secreting IFN-γ.

8.3.2 Phase II Study Plan

The clinical trial is a single-center, randomized, double-blind and placebo-controlled one. According to the occurrence of the solicited and non-solicited adverse events, and of the abnormal indexes of blood routine examination, blood biochemistry and routine urine test, phase II clinical trial may be started only 0~7 days after the first high dose of phase I clinical trial is vaccinated when the safety observation is completed and the safety is confirmed by DMC. 600 healthy adults aged 18~59 years were selected as the subjects of the clinical trial. Upon informed consent, physical examination and screening based on the inclusion/exclusion criteria, the subjects are enrolled to be vaccinated according to the emergency immunization schedule (Day 0, Day 14, Day 42 or Day 0, Day 14) or routine immunization schedule (Day 0, Day 28, Day 56 or Day 0, Day 28). 300 subjects were enrolled for each immunization schedule, randomized into 3 groups by a ratio of 2:2:1 and were vaccinated by the medium dose, high dose or placebo respectively according to the corresponding immunization schedule. The immunization schedule for the subjects numbered C001~C150 was Day 0, Day 14 and Day 42, while that for the subjects numbered C151~C300 was Day 0, Day 14. The immunization schedule for the subjects numbered D001~D150 was Day 0, Day 28 and

Day 56, while that for the subjects numbered D151~D300 was Day 0 and Day 28. The subjects given the third dose of Day 0, Day 14, Day 42 and Day 0, Day 28, Day 56 immunization schedule were enrolled in phases, and a total of 30 subjects numbered C001 ~ C030 were given the third dose first, and 30 subjects numbered D001 ~ D030 were given the third dose based on the abnormalities in blood routine and blood biochemical indexes and the occurrence of adverse events on Day 0 ~3 after vaccination. After it was preliminarily confirmed safe, a total of 30 subjects numbered D001~D030 were given the third dose. It is confirmed safe through assessment, the subjects numbered C031 ~ C150 and D031 ~ D150 may be given the third dose based on the abnormalities in blood routine and blood biochemical indexes and the occurrence of adverse events of 30 subjects in each of the above groups on Day 0~7 after vaccination.

The immediate reaction within 30 minutes after each dose of vaccination was observed; the local and systemic solicited adverse events on Day 0~7 and the non-solicited adverse events on Day 0~28 (Day 0~14 for the first dose under the emergency immunization schedule) after each vaccination were collected, and the SAE monitoring from the beginning of the vaccination to 6 months after the full-course vaccination was completed. The blood of volunteers is sampled at different times before and after immunization to test the serum neutralizing antibody for evaluating the immunogenicity and immunization persistence. Volunteers numbered C001~C030 and D001~D030 were required to have blood collected on D3 before and after the third dose for laboratory testing to evaluate the safety of the vaccine. If the blood routine and blood biochemical indexes before the immunization for the third dose were abnormal and had a clinical significance (Grade 2 or higher), the subjects would not be given the third dose.

Based on the immunogenicity results 6 months after 2 doses of vaccination in this study, the subjects under Day 0, Day 14 (C151~C300) and Day 0, Day 28 (D151~D300) 2-dose primary immunization schedules were given 1 dose of booster immunization 6 months after primary immunization. The adverse immediate reactions within 30 min after booster immunization were observed; local and systemic solicited adverse events

on Day 0~7 and the non-solicited adverse events on Day 0~28 after inoculation were collected; and the SAE monitoring 6 months after inoculation was completed.

See Table 11 for details on the Phase II clinical trial study plan.

Table 11 Phase II Clinical Trial Study Plan

	**Immunization Procedure (day)**	**Medium dose**	**High dose**	**Placebo**	**Total**	**Neutralizing antibody & anti-nuclear antibody test (day)**	**Blood routine examination and blood biochemistry * (day)**
Emergency immunization schedule	0,14,42	60	60	30	150	0,28,42,70,222†,402†	42,45
	0,14¶	60	60	30	150	0,28,42,194, 208†,374†	-
Routine immunization schedule	0,28,56	60	60	30	150	0,56,84,236†,416†	56,59
	0,28¶	60	60	30	150	0,56,208, 236†,388†	-
	**Total**	**240**	**240**	**120**	**600**		

* Only the subjects numbered as C001~C030 and D001~D030 underwent laboratory testing. ¶ The subjects (C151-C300, D151-D300) under Day 0, Day 14 and Day 0, Day 28 immunization schedules underwent 6-month booster immunization. Only neutralizing antibody was tested.

8.4 Randomization and Double-blind

8.4.1 Randomization

Randomized statisticians adopt the block randomization method. The SAS software (Version 9.4) is used to generate randomized blind codes for the subjects in different immunization schedules of Phase I and Phase II clinical trials. The blind code of the research vaccine is a "List of Corresponding Relationships between Random Numbers and Research Vaccines or Placebos", which is made in duplicate and sealed after blind coding is completed. The original is kept by the investigator for unblinding in the test, and the copy is kept by the Sponsor. The numbers of the vaccines used under the emergency immunization schedule and routine immunization schedule in Phase I clinical trial are A001-A072 and B001-B072 respectively. The numbers of the vaccines used under the emergency immunization schedule and routine immunization schedule in Phase II clinical trial are C001-C300 and D001-D300 respectively.

The randomized statistician uses the SAS software (Version 9.4) to generate a blind code of spare vaccines which are prepared as per the ratio of 1:1:1 for medium dose, high dose, and placebo in phase I clinical trial. The number of spare vaccines is X01-X36. Spare vaccines are prepared according to the ratio of 2:2:1 for medium dose, high dose, and placebo in phase II clinical trial and the number of spare vaccines is Y01-Y60. In case of any discoloration or damage of test vaccines, the vaccinator should report to the responsible person and principal investigator at site who should start the spare vaccine enablement procedure to obtain the number of spare vaccines through the online spare vaccine acquisition system, and replace the research vaccine with the spare vaccine.

All test vaccines and placebos will be labeled blind as described in "6.7 Vaccine Packaging". After enrollment, subjects were inoculated with the blinding vaccine consistent with their study number.

8.4.2 Double-blind

Double-blind design is adopted in this trial. Randomized statisticians and other blind coding personnel who are not involved in the trial are employed for blind coding of the vaccine, that is, paste the printed label on the designated position of each vaccine/placebo according to the blind code. Randomized statisticians supervise the vaccine blind coding, and guide the blind coding operators to label according to the blind code. After the blind coding is completed, the blind code shall be sealed by randomized statisticians. The whole blind coding process must be recorded in writing. The blind coding personnel shall neither participate in other related work of this clinical trial, nor disclose any information about the blind code to any person participating in this clinical trial.

8.4.3 Emergency unblinding

Randomized statisticians shall prepare emergency letters during blind coding. Each letter shall contain a random password for unblinding, and each random password can correspond to any study number. The group of the study number can be fed back through the online unblinding system. Each random password represents an opportunity

for unblinding. Only one study number can be subject to unblinding, and then it will become invalid. It is invalid for the study number that has been subject to unblinding. In this trial, 5 emergency envelopes are prepared for Phase I emergency and routine schedules, 10 emergency envelopes are prepared for Phase II emergency and routine schedules, and such envelopes shall be kept by the site personnel-in-charge. The blind review personnel shall check the opening and closing status of emergency envelopes.

During the study period, if emergency unblinding is jointly decided by the principal investigator, the Sponsor and DMC, the site personnel-in-charge shall open and read the emergency letter, log in to the online emergency unblinding system with the random unblinding password in the letter, perform emergency unblinding according to the prompt information, and make relevant records. The subjects with this study number will suspend the trial for withdrawal treatment, and the investigator will record the reasons for suspension in the CRF. The emergency letters that have been opened and read shall be kept properly and returned to the Sponsor after the trial.

8.4.4 Unblinding regulations

The unblinding of Phase I and Phase II clinical trials shall follow the time points below: upon obtaining the serum detection results 14 days after two doses of immunization in emergency immunization schedule; upon obtaining the serum detection results 28 days after two doses of immunization in routine immunization schedule. It shall be implemented jointly by the Sponsor, the principal investigator and the statistician, and the unblinding record shall be kept. After the unblinding, the personnel responsible for observing the subjects, judging the results and validating the data shall remain blind until the final database is locked.

8.4.5 Flow chart

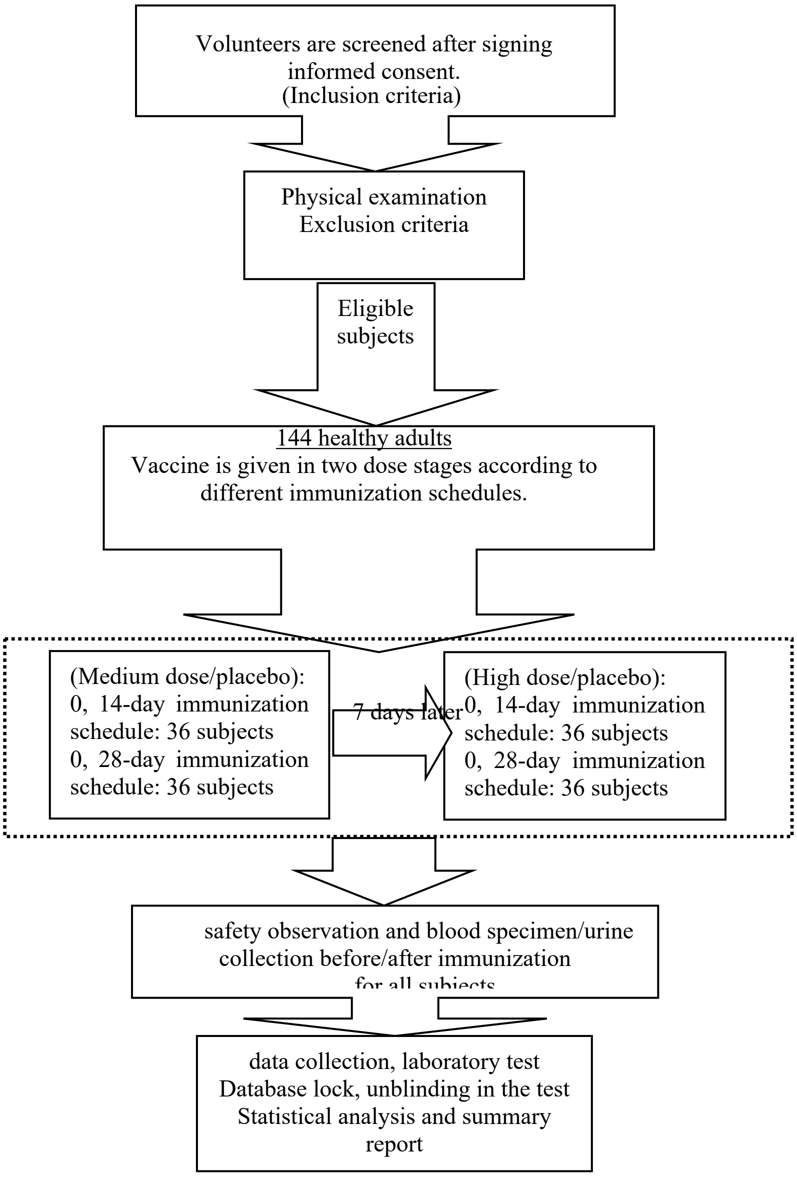

Flow Chart 7 Phase I clinical trial of COVID-19 Vaccine, Inactivated

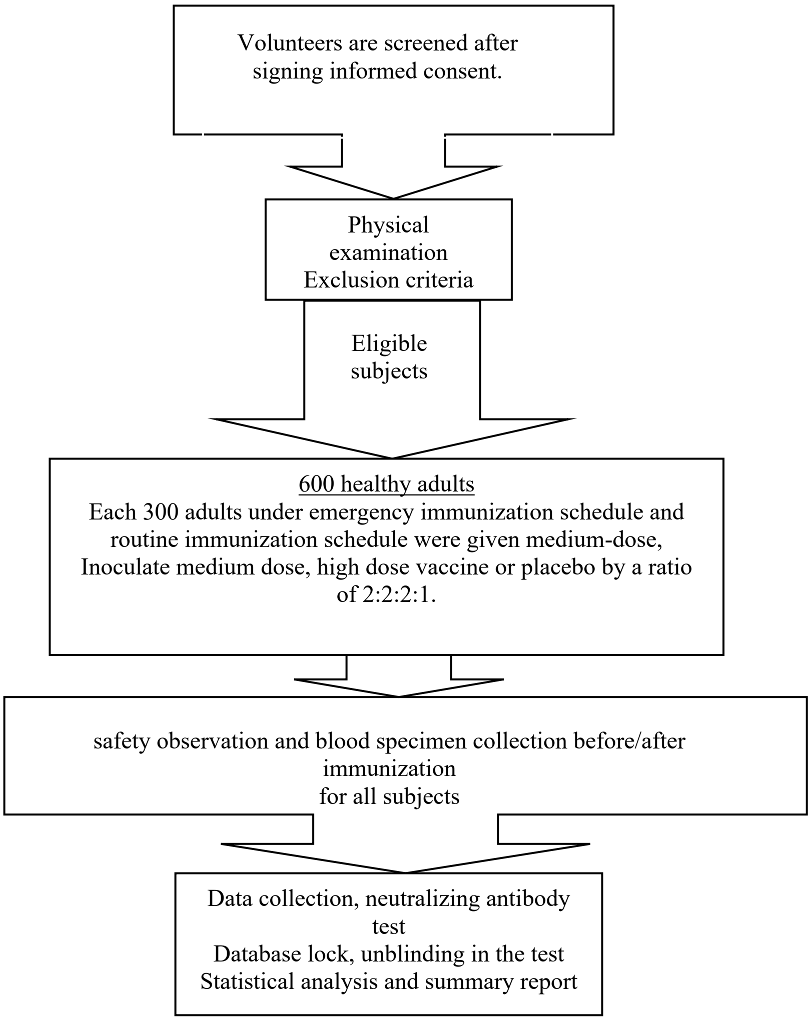

Flow Chart 8 Phase II clinical trial of COVID-19 Vaccine, Inactivated

8.5 Research Duration

8.5.1 Duration of Clinical Trial

Table 12 Duration of Clinical Trial

	Immunization Procedure	Time*
**Phase I**	Emergency schedule (Day 0, Day 14)	7.5 months
	Routine schedule (Day 0, Day 28)	8 months
**Phase II**	Emergency schedule (Day 0, Day 14, Day 42)	13.5 months
	Emergency schedule (Day 0, Day 14) +6 month booster immunization	13.5 months
	Routine schedule (Day 0, Day 28, Day 56)	14 months
	Routine schedule (Day 0, Day 28) +6 month booster immunization	14 months

8.5.2 Expected duration of participation for subjects

It is expected to take up to 14 months.

8.6 Test Suspension and Early Termination

After each dose of vaccination, statistics were given on adverse reaction of the subjects, and the test was suspended or terminated according to the following criteria.

Criteria for test suspension:

- One or more Grade-4 adverse reactions (local, systemic) related to vaccination;
- Over 15% of the subjects have Grade-3 or higher adverse reactions, including local reaction, systemic reaction, and vital signs.

Criteria for early termination:

- After the clinical trial is suspended, the investigator, sponsor and DMC will discuss and decide whether to terminate the trial;
- The sponsor requests a complete termination of the test and gives reasons;
- Independent Ethics Committee requests a complete termination of the test and gives reasons;
- The competent administrative department requests a complete termination of the test and gives reasons.

8.7 Protocol Violation and Deviation

The following is regarded as protocol violation (including but not limited to):

- Subjects do not meet the inclusion criteria or meet the exclusion criteria;
- Subjects received the wrong vaccine;
- SAE is not reported within the required time.

The following is regarded as protocol deviation (including but not limited to):

- Test vaccine is given not within the window period specified in the protocol;
- Blood sampling is conducted not within the window period specified in the protocol;
- Intervals with vaccination of other vaccines did not meet protocol requirements (excluding rabies vaccination or tetanus vaccination in case of emergency).

9 Subjects

9.1 Inclusion Criteria for Subjects

(1) Healthy subjects aged 18-59;

(2) Able to understand and sign the Informed Consent Form voluntarily;

(3) Provide legal proof of identity.

9.2 Exclusion Criteria for Subjects

(1) Having traveled to or lived in Wuhan or surrounding areas or communities where confirmed cases have been reported within the previous 14 days;

(2) Having contact with patients who were infected with COVID-19 (who have tested positive for nucleic acid detection) within the previous 14 days;

(3) Having contact with patients who have fever and symptoms of respiratory infections from Wuhan or surrounding areas or communities where confirmed cases have been reported within the previous 14 days;

(4) Having been in places such as houses, offices and classrooms where over 2 cases of fever and/or symptoms of respiratory infections have been reported within the previous 14 days;

(5) SARS record in self-report;

(6) Infection with COVID-19 recorded in self-report;

(7) IgG or IgM screening results were positive;

(8) The RT-PCR test results of throat and anal swabs were positive;

(9) Women who are breastfeeding, pregnant, or planning to become pregnant during the study period (Judgment is made based on subjects' self-report and urine pregnancy test results);

(10) Body mass index (BMI) $\geq$35 kg/m^2;

(11) Having a history of asthma and allergy to vaccine or vaccine ingredients and having serious adverse reactions to the vaccine such as urticaria, dyspnea and angioneurotic edema;

(12) Subjects with congenital malformations or developmental disorders, genetic defects, severe malnutrition, etc.;

(13) Subjects with autoimmune diseases or immune deficiency/immune inhibition;

(14) Subjects with severe chronic diseases, severe cardiovascular diseases and hypertension, diabetes, liver and kidney diseases and malignant tumors that can not be controlled by drugs;

(15) Subjects with serious neurological diseases (epilepsy, convulsions or tic) or mental diseases;

(16) Subjects with thyropathy or having a history of thyroidectomy, subjects with an absent or dysfunctional spleen and subjects with an absent spleen or splenectomy;

(17) Subjects with coagulation disorders diagnosed by a doctor (such as deficiency of coagulation factors, coagulation diseases and blood platelet disorders) or significant bruising or coagulation disorder;

(18) Having received the immunosuppressive therapy, cytotoxic therapy, and inhale corticosteroids (excluding corticosteroid spray in treatment of allergic rhinitis and surface corticosteroid treatment of acute non-concurrent dermatitis) in the past six months;

(19) Subjects with abnormal laboratory test results such as in hematology and biochemistry which are beyond the range of reference values and of clinical significance in physical examination (applicable for Phase I clinical trial only):

1) Blood routine test: white blood cell count, hemoglobin and platelet count.

2) Blood biochemistry: alanine aminotransferase (ALT), aspartate aminotransferase (AST), total bilirubin (TBIL), creatinine (CR) and creatine phosphokinase (CPK);

3) Routine urine indexes: urine protein (PRO), urine sugar and urine erythrocyte

(20) Chronic alcoholics or those having a history of drug abuse;

(21) Subjects who have received blood products within 3 months before vaccination with the test vaccine;

(22) Subjects who have received other study drugs within 30 days before vaccination with the test vaccine;

(23) Subjects who have received live attenuated vaccines within 14 days before

vaccination with the test vaccine;

(24) Subjects who have received subunit or inactivated vaccines within 7 days before vaccination with the test vaccine;

(25) Subjects having an attack of various acute or chronic diseases within 7 days;

(26) Subjects with axillary temperature >37.0°C before vaccination with the test vaccine;

(27) Subjects who are not suitable for participating in this clinical trial according to the investigator.

9.3 Exclusion Criteria for Second and Third Doses of Vaccination

Subjects with any adverse event listed in (1) to (4) are forbidden to continue vaccination but they can finish other research based on the investigator's judgment. For subjects with any adverse event listed in (5) and (6), it is up to the investigator to decide whether or not to vaccinate. For subjects with any adverse event listed in (7) to (10), the vaccination can be delayed within the time window specified in the schedule.

(1) Vaccines of the same type other than the test vaccine are used during the study period;

(2) Any serious adverse reactions that have a causal relationship with the test vaccine;

(3) Allergic shock or hypersensitivity after vaccination (including urticaria/rash that appears within 30 minutes after vaccination);

(4) Any confirmed or suspected autoimmune diseases or immunodeficiency diseases, including human immunodeficiency virus (HIV) infection;

(5) Acute or newly developed chronic diseases after vaccination;

(6) Other reactions as determined by the investigator (including severe pain, severe swelling, severe limitation of motion, persistent high fever, severe headache, or other systemic or local reactions);

(7) With acute illness (moderate or severe illness with or without fever) at the time of vaccination;

(8) Axillary temperature >37.0°C at the time of vaccination;

(9) Having received subunit or inactivated vaccines within 7 days and having received live attenuated vaccines within 14 days.

(10) Any other causes for which subjects are not suitable for vaccination according to the investigator.

9.4 Withdrawal and Termination Criteria for Subjects

(1) Subjects requested withdrawal from the clinical trial;

(2) An intolerable adverse event occurs whether it is relevant to the test drug or not;

(3) The health conditions of the subject make the trial not applicable to him or her;

(4) The investigator will decide whether the clinical abnormality (if any) of the subject is related to the vaccine, and whether the clinical trial shall be suspended ahead of schedule;

(5) Any other reasons considered by the investigator.

If a subject dropping out of the trial ahead of schedule has been inoculated with the test vaccine, the clinical trial data of the subject will be used for safety analysis. Subjects cannot be replaced during the study. When the subject inoculated with the vaccines used in the clinical trial withdraws or suspends the trial, the investigator shall provide necessary treatment to eliminate the clinical conditions related to the trial for the subject, and follow up until the diagnosis has been accomplished/subject is stable/subject is totally improved.

10 Method and Schedule

10.1 Visit Plan

10.1.1 Phase I Clinical Trial (0, 14-day Immunization Schedule)

Table 13 Visit Schedule for Phase I Clinical Trial (0, 14-day Immunization Schedule)

Follow-up		0	1	2	3	4	5	6	7	8	9
Follow-up period	D-21～D-1	D-7～D0	D0	D3[e]	D7[e]	D14[e]	D17[e]	D21[e]	D28[e]	D42[e]	D194[e]
Preliminary notice, subject recruitment	**X**										
Informed consent		**X**									

Demographic analysis			X									
Blood collection	Blood routine examination, blood biochemistry		X		X		X	X				
	IgG and IgM screening		X									
	Throat and anal swabs RT-PCR test		X									
	Serum antibody detection (Neutralizing antibody, IgG, IgM and anti-nuclear antibody)			X		X	X		X	X	X	X
	Inflammation factor test			X		X	X		X			
	IFN-γ secretion by T cell response			X			X			X		
Routine urine test			X		X		X	X				
Female urine pregnancy test				X			X					
General examination				X								
Screening based on inclusion/exclusion criteria[a]				X			X					
Inoculation[b]				X			X					
Subjects record the safety observation results on their daily diary/contact cards[c].				X	X	X	X	X	X	X	X	
Monitoring of adverse reactions/events (including adverse events of grade 3 and above and SAE)[cd]				X	X	X	X	X	X	X	X	X
Usage records of concomitant drugs/vaccines[cd]				X	X	X	X	X	X	X	X	X

a) Screening shall be conducted based on inclusion/exclusion criteria before vaccination of every dose.

b) Subjects will be observed in the observation room for 30 minutes to ensure no adverse events, especially acute allergic reactions, and then followed up regularly as required.

c) Safety observations include assessment of adverse reactions/events and body temperature measurements. Body temperature should be taken daily 0-7 days after inoculation and when fever is suspected. Subjects will record safety observation data in the Diary Card within 14 days after vaccination of the first dose and 28 days after vaccination of the second dose. Subjects will be interviewed regularly by the investigator to verify and record adverse events and concomitant drugs/vaccines.

d) During Day 42 to Day 194, SAE and the usage records of its concomitant drugs are only collected.

e) See "Visit Plan" for window period.

Visit plan:

Visit 0- Day -7-0, informed consent, **blood collection, urine collection, collection of throat and anal swabs**, laboratory indexes (blood routine examination, blood biochemistry, routine urine test, IgG & IgM, nucleic acid).

Visit 1- Day 0, Qualified subjects were enrolled; **blood collection, urine collection**

(only in female), vaccination of the first dose.

Visit 2- 3 days (±1 day) after 1st dose - Safety observation, drug use and other vaccination records were verified, and **blood and urine were collected.**

Visit 3- Day 7 (+3 days) after 1st dose - Safety observation, drug use and other vaccination records were verified, and **blood was collected.**

Visit 4- Day 14 (+5 days) after 1st dose - Safety observation, drug use and other vaccination records were verified, **blood and urine were collected** and the 2nd dose was given.

Visit 5- Day 3 (±1 day) after 2nddose - Safety observation, drug use and other vaccination records were verified, and **blood and urine were collected;** Examination.

Visit 6- Day 7 (±3 days) after 2nd dose - Safety observation, drug use and other vaccination records were verified, and **blood was collected.**

Visit 7- Day 14(+5 days) after 2nd dose - Safety observation, drug use and other vaccination records were verified, and **blood was collected.**

Visit 8- Day 28 (+10 days) after 2nd dose - Safety observation, drug use and other vaccination records were verified, and **blood was collected.**

Visits 8~9 - SAE observations, SAE concomitant drug use record and other special cases were verified.

Visit 9 - Day 180 (+30 days) after the 2nd dose - SAE observations, concomitant drug use record and other special cases were verified, and **blood was collected.**

10.1.2 Phase I Clinical Trial (0,28-day Immunization Schedule)

Table 14 Visit Schedule for Phase I Clinical Trial (0,28-day Immunization Schedule)

Follow-up			0	1	2	3	4	5	6	7	8	9
Follow-up period		D-21～D0	D-7～D0	D0	D3[e]	D7[e]	D28[e]	D31[e]	D35[e]	D42[e]	D56[e]	D208[e]
Preliminary notice, subject recruitment		**X**										
Informed consent			**X**									
Demographic analysis			**X**									
Blood collection	Blood routine examination, blood biochemistry		**X**		**X**		**X**	**X**				
	IgG and IgM screening		**X**									
	Throat and anal swabs RT-PCR test		**X**									

Follow-up			0	1	2	3	4	5	6	7	8	9
Follow-up period		D-21～D0	D-7～D0	D0	D3[e]	D7[e]	D28[e]	D31[e]	D35[e]	D42[e]	D56[e]	D208[e]
	Serum antibody detection (Neutralizing antibody, IgG, IgM and anti-nuclear antibody)			X			X		X	X	X	X
	Inflammation factor test			X		X	X		X			
	IFN-γ secretion by T cell response			X			X			X		
Routine urine test			X		X		X	X				
Female urine pregnancy test				X			X					
General examination				X								
Screening based on inclusion/exclusion criteria[a]				X			X					
Inoculation[b]				X			X					
Subjects record the safety observation results on their daily diary/contact cards[c].				X	X	X	X	X	X	X	X	
Monitoring of adverse reactions/events (including adverse events of grade 3 and above and SAE)[cd]				X	X	X	X	X	X	X	X	X
Usage records of concomitant drugs/vaccines[cd]				X	X	X	X	X	X	X	X	X

a) Screening shall be conducted based on inclusion/exclusion criteria before vaccination of every dose.

b) Subjects will be observed in the observation room for 30 minutes to ensure no adverse events, especially acute allergic reactions, and then followed up regularly as required.

c) Safety observations include assessment of adverse reactions/events and body temperature measurements. Body temperature should be taken daily 0-7 days after inoculation and when fever is suspected. Subjects will record safety observation data in the Diary Card within 28 days after vaccination of every dose. Subjects will be interviewed regularly by the investigator to verify and record adverse events and concomitant drugs/vaccines.

d) During Day 56 to Day 208, SAE and the usage records of its concomitant drugs are only collected.

e) See “Visit Plan” for window period.

Visit plan:

Visit 0- Day -7-0, informed consent, **blood collection, urine collection, collection of throat and anal swabs**, laboratory indexes (blood routine examination, blood biochemistry, routine urine test, IgG & IgM, nucleic acid).

Visit 1- Day 0, Qualified subjects were enrolled; **blood collection, urine collection (only in female),** vaccination of the first dose.

Visit 2- 3 days (±1 day) after 1st dose - Safety observation, drug use and other

vaccination records were verified, and **blood and urine were collected.**

Visit 3- Day 7 (+3 days) after 1st dose - Safety observation, drug use and other vaccination records were verified, and **blood was collected.**

Visit 4- Day 28 (+10 days) after 1st dose - Safety observation, drug use and other vaccination records were verified, **blood and urine were collected** and the 2nd dose was given.

Visit 5- Day 3 (±1 day) after 2nddose - Safety observation, drug use and other vaccination records were verified, and **blood and urine were collected;** Examination.

Visit 6- Day 7 (±3 days) after 2nd dose - Safety observation, drug use and other vaccination records were verified, and **blood was collected.**

Visit 7- Day 14 (+5 days) after 2nd dose - Safety observation, drug use and other vaccination records were verified, and **blood was collected.**

Visit 8- Day 28 (+10 days) after 2nd dose - Safety observation, drug use and other vaccination records were verified, and **blood was collected.**

Visits 8~9 - SAE observations, SAE concomitant drug use record and other special cases were verified.

Visit 9 - Day 180 (+30 days) after the second dose of vaccination - SAE observations, SAE concomitant drug use record and other special cases were verified, and blood was collected.

10.1.3 Phase II clinical trial (emergency immunization schedule)

Table 15 Visit Schedule for Phase II Clinical Trial (0, 14, 42-day Immunization Schedule)

Follow-up		0	1	2	3	4	5	6	7	8	9	10	11	12
Follow-up period	D-21～D0	D-7～D0	D0	D7[e]	D14[e]	D21[e]	D28[e]	D42[e]	D42[e]	D45[e]	D49[e]	D70[e]	D222[e]	D402[e]
Preliminary notice, subject recruitment	**X**													
Informed consent		**X**												
Demographic analysis		**X**												
<u>Blood routine examination, blood biochemistry</u>									**X**	**X**				
Neutralizing antibody test & anti-nuclear			**X**				**X**	**X**				**X**	**X†**	**X†**

antibody test														
Throat and anal swabs RT-PCR test		X												
IgG and IgM screening		X												
General examination			X											
Female urine pregnancy test			X		X				X					
Screening based on inclusion/exclusion criteria[a]			X		X				X					
Inoculation[b]			X		X				X					
Subjects record the safety observation results on their daily diary/contact cards[c].			X	X	X	X	X	X	X	X	X	X		
Monitoring of adverse reactions/events (including adverse events of grade 3 and above and SAE)[cd]			X	X	X	X	X	X	X	X	X	X	X	
Usage records of concomitant drugs/vaccines[cd]			X	X	X	X	X	X	X	X	X	X	X	

† Only neutralizing antibody was tested.

a) Screening shall be conducted based on inclusion/exclusion criteria before vaccination of every dose.
b) Subjects will be observed in the observation room for 30 minutes to ensure no adverse events, especially acute allergic reactions, and then followed up regularly as required.
c) Safety observations include assessment of adverse reactions/events and body temperature measurements. Body temperature should be taken daily 0-7 days after inoculation and when fever is suspected. Subjects will record safety observation data in the Diary Card within 14 days after vaccination of the first dose and 28 days after vaccination of the second and third dose. Subjects will be interviewed regularly by the investigator to verify and record adverse events and concomitant drugs/vaccines.
d) During Day 70 to Day 222, SAE and the usage records of its concomitant drugs are only collected.
e) See "Visit Plan" for window period.

Visit plan:

Visit 0 - Day -7~0 - IgG/IgM antibodies and nucleic acid were screened upon informed consent.

Visit 1- Day 0, Qualified subjects were enrolled; **blood collection,** vaccination of the first dose.

Visit 2- 7 days (+3 days) after 1st dose - Safety observation, drug use and other vaccination records were verified.

Visit 3- 14 days (+5 days) after 1st dose - Safety observation, drug use and other vaccination records were verified, and the 2nd dose was given.

Visit 4- 7 days (+3 days) after 2nd dose - Safety observation, drug use and other vaccination records were verified.

Visit 5- Day 14 (+5 days) after 2nd dose - Safety observation, drug use and other vaccination records were verified, and **blood was collected.**

Visit 6- Day 28 (+10 days) after 2nd dose - Safety observation, drug use and other vaccination records were verified, and **blood was collected.**

Visit 7 - Day 28 (+30 days) after second dose of vaccination - Subjects numbered C001~C030 were required to have blood collected, and those whose blood routine and blood biochemical indexes met the vaccination conditions would be given the third dose of vaccine; subjects numbered C031~C150 were not required to have blood collected and were directly given the third dose of vaccine.

Visit 8- Day 3 (±2 days) after the third dose of vaccination- only subjects numbered C001~C030 received this visit to verify safety observation, drug use and other vaccination records, and blood was collected.

Visit 8- Day 7 (+3 days) after the 3rd dose - Safety observation, drug use and other vaccination records were verified.

Visit 10- 28 days (+10 days) after 3rd dose - Safety observation, drug use and other vaccination records were verified, and **blood was collected.**

Visits 10~11 - SAE observations, SAE concomitant drug use record and other special cases were verified. **Visit 11** - Day 180 (+30 days) after the third dose of vaccination- SAE observations, SAE concomitant drug use record and other special cases were verified, and blood was collected.

Visit 12 - Day 360 (+30 days) after the third dose of vaccination - blood collection.

Table 16 Visit Schedule for Phase II Clinical Trial (Day 0, Day 14 Immunization Schedule + 6-month booster immunization)

Follow-up	Primary immunization									Booster immunization				
		0	1	2	3	4	5	6	7	8	9	10	11	12
Follow-up period	D-21～D0	D-7～D0	D0	D7[e]	D14[e]	D21[e]	D28[e]	D42[e]	D194[e]	D194[e]	D201[e]	D208[e]	D222[e]	D374[e]
Preliminary notice, subject recruitment	X													
Informed consent		X												
Demographic analysis		X												
Neutralizing antibody test & anti-nuclear antibody test			X				X	X	X			X†		X†
Pharyngeal and anal swabs RT-PCR test		X												
IgG and IgM screening		X												
General examination			X											
Female urine pregnancy test			X		X					X				
Screening based on inclusion/exclusion criteria[a]			X		X					X				
Inoculation[b]			X		X					X				
Subjects record the safety observation results on their daily diary/contact cards[c].			X	X	X	X	X	X		X	X	X	X	
Adverse reaction/event monitoring (including level 3 or higher adverse events and SAEs)[cd]			X	X	X	X	X	X	X	X	X	X	X	X
Usage records of concomitant drugs/vaccines[cd]			X	X	X	X	X	X	X	X	X	X	X	X

† Only neutralizing antibody was tested.

a) Screening shall be conducted based on inclusion/exclusion criteria before vaccination of every dose.

b) Subjects will be observed in the observation room for 30 minutes to ensure no adverse events, especially acute allergic reactions, and then followed up regularly as required.

c) Safety observations include assessment of adverse reactions/events and body temperature measurements. Body temperature should be taken daily 0-7 days after inoculation and when fever is suspected. Subjects recorded safety observation data in the Diary Card on Day 14

after first dose of vaccination and on Day 28 after the second dose of vaccination and booster inoculation, and were interviewed regularly by the investigator to verify and record adverse events and concomitant drugs/vaccines.

d) Only records of SAE and SAE concomitant drug use were collected from the end of visit 6 to visit 8 and from the end of visit 11 to visit 12.

e) See “Visit Plan” for window period.

Visit plan:

Visit 0 - Day -7~0 - IgG/IgM antibodies and nucleic acid were screened upon informed consent.

Visit 1- Day 0, Qualified subjects were enrolled; **blood collection,** vaccination of the first dose.

Visit 2- 7 days (+3 days) after 1st dose - Safety observation, drug use and other vaccination records were verified.

Visit 3- 14 days (+5 days) after 1st dose - Safety observation, drug use and other vaccination records were verified, and the 2nd dose was given.

Visit 4- 7 days (+3 days) after 2nd dose - Safety observation, drug use and other vaccination records were verified.

Visit 8- Day 14 (+5 days) after 2nd dose - Safety observation, drug use and other vaccination records were verified, and **blood was collected.**

Visit 6- Day 28 (+10 days) after 2nd dose - Safety observation, drug use and other vaccination records were verified, and **blood was collected.**

Visits 6~7 - SAE observations, SAE concomitant drug use record and other special cases were verified.

Visit 7 - Day 180 (+30 days) after the second dose of vaccination - SAE observations, SAE concomitant drug use record and other special cases were verified, and blood was collected.

Visit 8 - Day 180 (+60 days) after the second dose of vaccination - SAE observations, SAE concomitant drug use record and other special cases were verified, and booster immunization were given.

Visit 9 - Day 7 (+3 days) after booster immunization -safety observation, drug use and other vaccination records were verified.

Visit 10 - Day 14 (+5 days) after booster immunization -safety observation, drug use and other vaccination records were verified, and blood was collected.

Visit 11 - Day 28 (+10 days) after booster immunization -safety observation, drug use and other vaccination records were verified.

Visits 11~12 - SAE observations, SAE concomitant drug use record and other special cases were verified.

Visit 12 - Day 180 (+30 days) after booster immunization - SAE observations, SAE concomitant drug use record and other special cases were verified, and blood was collected.

10.1.4 Phase II clinical trial (routine immunization schedule)

Table 17 Visit Schedule for Phase II Clinical Trial (0, 28, 56-day Immunization Schedule)

Follow-up		0	1	2	3	4	5	6	7	8	9	10	11
Follow-up period	D-21～D0	D-7～D0	D0	D7[e]	D28[e]	D35[e]	D56[e]	D56[e]	D59[e]	D63[e]	D84[e]	D236[e]	D416[e]
Preliminary notice, subject recruitment	**X**												
Informed consent		**X**											
Demographic analysis		**X**											
<u>Blood routine examination, blood biochemistry</u>								**X**	**X**				
Neutralizing antibody test/ anti-nuclear antibody test			**X**				**X**				**X**	**X†**	**X†**
Throat and anal swabs RT-PCR test		**X**											
IgG and IgM screening		**X**											
General examination			**X**										
Female urine pregnancy test			**X**		**X**			**X**					
Screening based on inclusion/exclusion criteria[a]			**X**		**X**			**X**					
Inoculation[b]			**X**		**X**			**X**					
Subjects record the safety observation results on their daily			**X**	**X**	**X**	**X**	**X**	**X**	**X**	**X**	**X**		

diary/contact cards[c].													
Monitoring of adverse reactions/events (including adverse events of grade 3 and above and SAE)[cd]			X	X	X	X	X	X	X	X	X	X	
Usage records of concomitant drugs/vaccines[cd]			X	X	X	X	X	X	X	X	X	X	

† Only neutralizing antibody was tested.

a) Screening shall be conducted based on inclusion/exclusion criteria before vaccination of every dose.

b) Subjects will be observed in the observation room for 30 minutes to ensure no adverse events, especially acute allergic reactions, and then followed up regularly as required.

c) Safety observations include assessment of adverse reactions/events and body temperature measurements. Body temperature should be taken daily 0-7 days after inoculation and when fever is suspected. Subjects will record safety observation data in the Diary Card within 28 days after vaccination of every dose. Subjects will be interviewed regularly by the investigator to verify and record adverse events and concomitant drugs/vaccines.

d) During Day 84 to Day 236, SAE and the usage records of its concomitant drugs are only collected.

e) See "Visit Plan" for window period.

Visit plan:

Visit 0 - Day -7~0 - IgG/IgM antibodies and nucleic acid were screened upon informed consent.

Visit 1- Day 0, Qualified subjects were enrolled; **blood collection,** vaccination of the first dose.

Visit 2- 7 days (+3 days) after 1st dose - Safety observation, drug use and other vaccination records were verified.

Visit 3- 28 days (+10 days) after 1st dose - Safety observation, drug use and other vaccination records were verified, and the 2nd dose was given.

Visit 4- 7 days (+3 days) after 2nd dose - Safety observation, drug use and other vaccination records were verified.

Visit 5- Day 28 (+10 days) after 2nd dose - Safety observation, drug use and other vaccination records were verified, and **blood was collected.**

Visit 6 - Day 28 (+30 days) after second dose of vaccination - Subjects numbered

D001~D030 were required to have blood collected, and those whose blood routine and blood biochemical indexes met the vaccination conditions would be given the third dose of vaccine; subjects numbered D031~D150 were not required to have blood collected and were directly given the third dose of vaccine.

Visit 7- Day 3 (±2 days) after the third dose of vaccination - only subjects numbered D001~D030 received this visit to verify safety observation, drug use and other vaccination records, and blood was collected.

Visit 8- Day 7 (+3 days) after the 3rd dose - Safety observation, drug use and other vaccination records were verified.

Visit 8- Day 28 (+10 days) after the 3rd dose - Safety observation, drug use and other vaccination records were verified, and **blood was collected.**

Visits 9 ~10 - SAE observations, SAE concomitant drug use record and other special cases of the subjects were verified.

Visit 10 - Day 180 (+30 days) after the third dose of vaccination - SAE observations, SAE concomitant drug use record and other special cases were verified, and blood was collected.

Visit 11 - Day 360 (+30 days) after the third dose of vaccination - blood collection.

Table 18 Visit Schedule for Phase II Clinical Trial (Day 0, 28 Immunization Schedule + 6-month booster immunization)

	Primary immunization								Booster immunization			
Follow-up		0	1	2	3	4	5	6	7	8	9	10
Follow-up period	D-21～D0	D-7～D0	D0	D7e	D28e	D35e	D56e	D208e	D208e	D215e	D236e	D388e
Preliminary notice, subject recruitment	X											
Informed consent		X										
Demographic analysis		X										
Neutralizing antibody test & anti-nuclear antibody test			X				X	X			X†	X†
Throat and anal swabs RT-PCR test		X										
IgG and IgM screening		X										
General examination			X									
Female urine pregnancy test			X		X				X			

Screening based on inclusion/exclusion criteria[a]			X		X				X			
Inoculation[b]			X		X				X			
Subjects record the safety observation results on their daily diary/contact cards[c].			X	X	X	X	X		X	X	X	
Monitoring of adverse reactions/events (including adverse events of grade 3 and above and SAE)[cd]			X	X	X	X	X	X	X	X	X	X
Usage records of concomitant drugs/vaccines[cd]			X	X	X	X	X	X	X	X	X	X

† Only neutralizing antibody was tested.

a) Screening shall be conducted based on inclusion/exclusion criteria before vaccination of every dose.

b) Subjects will be observed in the observation room for 30 minutes to ensure no adverse events, especially acute allergic reactions, and then followed up regularly as required.

c) Safety observations include assessment of adverse reactions/events and body temperature measurements. Body temperature should be taken daily 0-7 days after inoculation and when fever is suspected. Subjects will record safety observation data in the Diary Card within 28 days after vaccination of every dose. Subjects will be interviewed regularly by the investigator to verify and record adverse events and concomitant drugs/vaccines.

d) Only records of SAE and SAE concomitant drug use were collected from the end of visit 5 to visit 7 and from the end of visit 9 to visit 10.

e) See "Visit Plan" for window period.

Visit plan:

Visit 0 - Day -7~0 - IgG/IgM antibodies and nucleic acid were screened upon informed consent.

Visit 1- Day 0, Qualified subjects were enrolled; **blood collection,** vaccination of the first dose.

Visit 2- 7 days (+3 days) after 1st dose - Safety observation, drug use and other vaccination records were verified.

Visit 3- 28 days (+10 days) after 1st dose - Safety observation, drug use and other vaccination records were verified, and the 2nd dose was given.

Visit 4- 7 days (+3 days) after 2nd dose - Safety observation, drug use and other vaccination records were verified.

Visit 5- Day 28 (+10 days) after 2nd dose - Safety observation, drug use and other

vaccination records were verified, and **blood was collected.**

Visits 5 & 6 - SAE observations, SAE concomitant drug use record and other special cases of the subjects were verified.

Visit 6- Day 180 (+ 30 days) after second dose of vaccination - safety observation, drug use and other vaccination records were verified, and blood was collected.

Visit 7- Day 180 (+ 60 days) after second dose of vaccination- safety observation, drug use and other vaccination records were verified, and blood was collected.

Visit 8 - Day 7 (+3 days) after booster immunization -safety observation, drug use and other vaccination records were verified.

Visit 9 - Day 28 (+10 days) after booster immunization -safety observation, drug use and other vaccination records were verified, and blood was collected.

Visits 9 ~10 - SAE observations, SAE concomitant drug use record and other special cases of the subjects were verified.

Visit 10 - Day 180 (+30 days) after booster immunization - SAE observations, SAE concomitant drug use record and other special cases were verified, and blood was collected.

10.2 Recruitment and Informed Consent

Recruitment notices will be issued to volunteers who meet the enrollment criteria. The informed consent will be explained to the volunteers in detail. Under the condition of voluntary participation, the volunteers and the study doctors sign the informed consent which is in duplicate, and the copy is reserved by the volunteer.

10.3 Screening and Random Enrollment

Subjects who are normal in physical examination (Women undergo a urine pregnancy test to rule out pregnancy.) and screened qualified as per other inclusion/exclusion criteria (Screening No. consists of S and screening order, such as "S0001".) will be enrolled and given Research Number based on enrollment order. The study numbers of the subjects under the emergency immunization schedule and routine immunization schedule in Phase I are A001-A072 and B001-B072 respectively. The study numbers of the subjects under the emergency immunization schedule and routine immunization schedule in Phase II are C001-C300 and D001-D300 respectively.

10.4 Vaccination

According to the Research Numbers of the subjects, the vaccinator takes the vaccine with the corresponding number, open it and check the numbers on the label of the vaccine bottle, on the movable label in the packaging box and on the label outside the vaccine package. After confirmation, the vaccinator performs vaccination. Then, he tears off the movable label in the packaging box, paste it in the corresponding position of the original record sheet, and fill in the vaccination information in the original record book.

See “8.3 Study Plan” for immunization schedules.

10.5 Safety Follow-up and Observation

Diary Card was issued to subjects after vaccination of each dose. Subjects were required to record the solicited (systemic, local) adverse events 0 to 7 days after vaccination and the non-solicited adverse events 0 to 28 days (0 to 14 days for the first dose under emergency immunization schedule) after vaccination. Subjects were required to record any clinical symptoms, drugs and usage of other vaccines in detail on their diary cards 0 to 28 days (0 to 14 days for the first dose under emergency immunization schedule) after vaccination. The investigators verified the adverse events reported by subjects.

Systematic observation was conducted on Day 7 after vaccination. Subjects observed their own symptoms and signs and filled in the Diary Card on a daily basis; meanwhile, the investigator paid a visit (no less than 2 face-to-face visits in Phase I), and collected safety observation data on Day 0 ~ 7; the occurrence of adverse events was recorded on Day 8 ~ 28 (Day 8 ~ 14 for the first dose of the emergency immunization schedule) by combining active reporting by subjects with investigator regular follow-up. Safety follow-up was observed until the 28th day after vaccination.

Subjects were informed that any adverse events should be recorded at any time and that acute allergic reaction, grade 3 or higher adverse events, and SAE should be reported to the investigator at any time. The investigator should conduct investigation for verification and follow-up until those problems are resolved, and finally complete

detailed investigation and follow-up records which should include the following contents:

- Description of adverse events
- Start time and end time of adverse events
- Severity of adverse events
- Correlation with vaccination
- Laboratory test results
- Processing measures

If subjects develop acute allergic reaction and grade 3 or higher adverse events after vaccination, treatment should be provided in time to relieve the pain of the subjects as soon as possible; If subjects develop SAE after vaccination, a medical green passage should be initiated for immediate medical treatment. Medication and medical treatment at each follow-up should be recorded in detail.

10.6 Sampling

- **Sampling plan**

Sampling before/after immunization should be conducted according to “10.1 Visit Plan” for subjects. The sampling plan is as follows:

Table 19 Sampling Plan for Phase I Clinical Trial (0, 14-day Immunization Schedule)

Sample type	No. of follow-up	0	1	2	3	4	5	6	7	8	9
Venous blood (ml)	Follow-up period	D-7～D0	D0	D3[e]	D7[e]	D14[e]	D17[e]	D21[e]	D28[e]	D42[e]	D194[e]
	Blood routine	**3**		**3**		**3**	**3**				
	Blood biochemistry	**5**		**5**		**5**	**5**				
	Inflammatory factor		**5**		**5**	**5**		**5**			
	Neutralizing antibody, IgG antibody, IgM and antinuclear antibody		**5**		**5**	**5**		**5**	**5**	**5**	**5**
	T cell reaction		**10**			**10**			**10**		
	Total	**8**	**20**	**8**	**10**	**28**	**8**	**10**	**15**	**5**	**5**
Fingertip blood (ml)	IgG and IgM screening	**Proper dose**									
Urine (ml)	Urine routine test	**5~10**		**5~10**		**5~10**	**5~10**				
	Urine pregnancy test (female)		**5~10**			**5~10**					

Throat and anal swabs	RT-PCR test	**Proper dose**									

e: See "10.1 Visit Plan" for window period.

Table 20 Sampling Plan for Phase I Clinical Trial (0,28-day Immunization Schedule)

Sample type	No. of follow-up	0	1	2	3	4	5	6	7	8	9
Venous blood (ml)	Follow-up period	D-7～D0	D0	D3[e]	D7[e]	D28[e]	D31[e]	D35[e]	D42[e]	D56[e]	D208[e]
	Blood routine	**3**		**3**		**3**	**3**				
	Blood biochemistry	**5**		**5**		**5**	**5**				
	Inflammatory factor		**5**		**5**	**5**		**5**			
	Neutralizing antibody, IgG antibody, IgM and antinuclear antibody		**5**			**5**		**5**	**5**	**5**	**5**
	T cell reaction		**10**			**10**			**10**		
	Total	**8**	**20**	**8**	**5**	**28**	**8**	**10**	**15**	**5**	**5**
Fingertip blood (ml)	IgG and IgM screening	**Proper dose**									
Urine (ml)	Urine routine test	**5~10**		**5~10**		**5~10**	**5~10**				
	Urine pregnancy test (female)		**5~10**			**5~10**					
Throat and anal swabs	RT-PCR test	**Proper dose**									

e: See "10.1 Visit Plan" for window period.

Table 21 Sampling Plan for Phase II Clinical Trial (0, 14, 42-day Immunization Schedule)

Sample type	No. of follow-up	0	1	2	3	4	5	6	7	8	9	10	11	12
	Follow-up period	D-7～D0	D0	D7[e]	D14[e]	D21[e]	D28[e]	D42[e]	D42[e]	D45[e]	D49[e]	D70[e]	D222[e]	D402[e]
Fingertip blood (ml)	IgG and IgM screening	**Proper dose**												
Throat and anal swabs	RT-PCR test	**Proper dose**												
Venous blood (ml)	Blood routine examination*								**3**	**3**				
	blood biochemistry #								**5**	**5**				
	Neutralizing antibody & antinuclear antibody		**3**				**3**	**3**				**3**	**3†**	**3†**
	Total		**3**				**3**	**3**	**8**	**8**		**3**	**3**	**3**
Urine (ml)	Urine pregnancy test		**5~10**		**5~10**				**5~10**					

	(female)													

† Only neutralizing antibody was tested; e: See “10.1 Visit Plan” for window period.

* Only the subjects numbered as C001~C030 underwent blood routine test;

* Only the subjects numbered as C001~C030 underwent blood biochemistry test;

Table 22 Sampling Plan for Phase II Clinical Trial (Day 0, Day 14 Immunization Schedule +6-month Booster Immunization)

Sample type	No. of follow-up	Primary immunization								Booster immunization				
		0	1	2	3	4	5	6	7	8	9	10	11	12
	Follow-up period	D-7～D0	D0	D7[e]	D14[e]	D21[e]	D28[e]	D42[e]	D194[e]	D194[e]	D201[e]	D208[e]	D222[e]	D374[e]
Fingertip blood (ml)	IgG and IgM screening	**Proper dose**												
Throat swab + anal swab	RT-PCR test	**Proper dose**												
Venous blood (ml)	Neutralizing antibody & antinuclear antibody		**3**				**3**	**3**	**3**			**3†**		**3†**
Urine (ml)	Urine pregnancy test (female)		**5~10**		**5~10**					**5~10**				

† Only neutralizing antibody was tested; e: See “10.1 Visit Plan” for window period.

Table 23 Sampling Plan for Phase II Clinical Trial (0, 28, 56-day Immunization Schedule)

Sample type	No. of follow-up	0	1	2	3	4	5	6	7	8	9	10	11
	Follow-up period	D-7～D0	D0	D7[e]	D28[e]	D35[e]	D56[e]	D56[e]	D59[e]	D63[e]	D84[e]	D236[e]	D416[e]
Fingertip blood (ml)	IgG and IgM screening	**Proper dose**											
Throat and anal swabs	RT-PCR test	**Proper dose**											
Venous blood (ml)	Blood routine examination*							**3**	**3**				
	blood biochemistry #							**5**	**5**				
	Neutralizing antibody & antinuclear		**3**				**3**				**3**	**3†**	**3†**

	antibody												
	Total		**3**				**3**	**8**	**8**		**3**	**3**	**3**
Urine (ml)	Urine pregnancy test (female)		**5~10**		**5~10**			**5~10**					

† Only neutralizing antibody was tested; e: See "10.1 Visit Plan" for window period.

* Only the subjects numbered as D001~D030 underwent blood routine test;

* Only the subjects numbered as D001~D030 underwent blood biochemistry test;

Table 24 Sampling Plan for Phase II Clinical Trial (Day 0, Day 28 Immunization Schedule +6-month Booster Immunization)

Sample type	No. of follow-up	Primary immunization							Booster immunization			
		0	1	2	3	4	5	6	7	8	9	10
	Follow-up period	D-7～D0	D0	D7[e]	D28[e]	D35[e]	D56[e]	D208[e]	D208[e]	D215[e]	D236[e]	D388[e]
Fingertip blood (ml)	IgG and IgM screening	**Proper dose**										
Throat and anal swabs	RT-PCR test	**Proper dose**										
Venous blood (ml)	Neutralizing antibody & antinuclear antibody		**3**				**3**	**3**			**3†**	**3†**
Urine (ml)	Urine pregnancy test (female)		**5~10**		**5~10**				**5~10**			

† Only neutralizing antibody was tested; e: See “10.1 Visit Plan” for window period.

- **Sample numbering principle**

During screening, samples are numbered as “screening No. + serial number of sampling” and the samples of enrolled subjects are numbered as “study number + serial number of sampling”.

- **Sample management**

All samples collected on site should be sent to the laboratory in time to complete the handover with laboratory personnel.

Serum shall be separated from blood samples for serum antibody (neutralizing antibody/IgG/IgM) test, placed into 2 tubes (no less than 1ml for serum tube A in phase I and no less than 0.5ml in phase II, and tube B for backup serum), and recorded. After separation, serum should be kept below -20 ºC. Blood samples used for IFN-γ secretory reaction of specific T cells should be kept below -20 ºC. Record should be kept for sample handover, serum separation and sample preservation.

For all submitted samples, specimen submission record should be made and temperature control record during submission should be kept.

10.7 Safety Evaluation

10.7.1 Safety Observation Indexes

Solicited local adverse events: pain, induration, swelling, vaccinal areola, skin rash and pruritus.

Solicited systemic adverse events (including vital signs): fever (axillary temperature), acute allergic reaction, abnormal skin and mucosa, diarrhea, anorexia, vomiting, nausea, muscle pain, headache, cough and fatigue.

Phase I laboratory test:

Blood routine examination: white blood count, hemoglobin and platelet count;

Blood biochemistry: alanine aminotransferase (ALT), aspartate aminotransferase (AST), total bilirubin (TBIL), creatinine (CR) and creatine phosphokinase (CPK).

Routine urine test: urine protein (PRO), urine sugar and urine erythrocyte.

Phase II laboratory test (only subjects numbered C001～C030 and D001～D030 were given the third dose):

Blood routine examination: white blood count, hemoglobin and platelet count;

Blood biochemistry: alanine aminotransferase (ALT), aspartate aminotransferase (AST), total bilirubin (TBIL), creatinine (CR), creatine phosphokinase (CPK), and glucose.

10.7.2 Definition of adverse event/reaction

The safety of the vaccine will be evaluated based on the range, intensity and severity of local adverse events, systemic adverse events, vital signs and adverse events for laboratory test indicators and on correlation of adverse events with vaccination.

All adverse medical events occurring during the trial (i.e., from the signing of the informed consent) will be collected, recorded, and reported by the investigator to the sponsor and the inspector.

(1) Adverse event (AE): any untoward medical occurrence in subjects after vaccination with the test vaccine in a clinical trial, which does not necessarily associated with the test vaccine.

(2) Adverse reaction: adverse events related to vaccination with the test vaccine that occur during vaccination at prescribed doses and schedules.

(3) Serious adverse events (SAE): events arising during the clinical trial which lead to hospitalization or prolonged hospitalization, injury/disability, working ability damage, life-threat conditions, death and congenital malformation or other events.

(4) Solicited/non-solicited adverse events: the solicitation period (within 0-7 days after vaccination with each dose) and non-solicitation period (8-28 days after vaccination with each dose). The solicited symptoms occurring during the solicitation period are referred to as solicited adverse events, and other symptoms occurring during the solicitation period and all symptoms occurring during the non-solicitation period are referred to as non-solicited adverse events.

10.7.3 Outcome of Adverse Events

The outcomes of adverse events include: (1) recovery, (2) not recovered, (3) recovered with sequela, (4) death, (5) loss to follow-up/unknown.

10.7.4 Judgment on Clinical Significance of Laboratory Indexes

Judgment on clinical significance includes: (1) within the range of reference values; (2) There was no clinical significance if not within the range of reference values; (3) There was clinical significance if not within the range of reference values.

10.7.5 Correlation Between Adverse Events and Vaccines

Investigators should try their best to explain AE and assess the possible causal links, i.e., the causal link with the inoculation of the research vaccine and the superseding causes (such as the medical history and combined treatment of underlying diseases). It is applicable to all AEs, including the severe and non-severe ones.

The causal link assessment will be determined by the degree of the reasonable explanation of events obtained in the following one or more aspects:

As for the preparations of such kind, reactions of similar properties had been observed previously;

For preparations of similar kinds, similar events had been reported on literature;

From the perspective of time, the events occur with the inoculation of the research vaccine and re-occur after the re-inoculation of the research vaccine.

According to the definition, all solicited AEs (i.e., local adverse reactions in solicited reports) occurred at the vaccination site will be deemed as related to vaccination.

The causal links of AE shall be assessed by investigators according to the

following questions, and whether there are reasonable probabilities that the AE is caused by vaccination:

(1) Definitely irrelevant: Adverse events may be caused by other factors, such as subjects' clinical conditions, other treatment or concomitant medication.

(2) Possibly irrelevant: Adverse events may be caused by other factors, such as subjects' clinical conditions, other treatment or concomitant medication; inconsistent with the known information of the test vaccine.

(3) Possibly relevant: Adverse events are consistent with the known information of the test vaccine. They are related causally to the test vaccine and may be related to other factors.

(4) Probably relevant: Adverse events are consistent with the known information of the test vaccine. They are related causally to the test vaccine and cannot be interpreted by other factors, such as subjects' clinical conditions, other treatment or concomitant drugs.

(5) Definitely relevant: Adverse events are consistent with the known information of the test vaccine. They are related causally to the test vaccine and cannot be interpreted by other factors, such as subjects' clinical conditions, other treatment or concomitant drugs. In addition, adverse events are repeated when the test vaccine is administered to subjects again.

10.7.6 Handling of Adverse Events

Reactions below grade 2 such as vaccinal areola, swelling, pain, or (and) fever and general malaise after vaccination can generally disappear spontaneously without special treatment.

The investigator should make investigation and medical follow-up on the adverse reactions/events of grade 3 and above that occur in subjects from the start of immunization to 28 days after immunization, including medical history, physical examination and necessary laboratory examination, treatment and tracking until the event is solved, and detailed investigation records should be completed. Investigation records should include symptoms, signs, diagnosis, and laboratory results.

In the event of a serious adverse event, the investigator should promptly take the necessary actions and report it within 24 hours. If any of the female subjects became pregnant during the study period, they will be treated as those with SAE. During test observation, subjects who developed fever, with cough and other respiratory symptoms should be seen immediately at the designated hospitals, throat swabs/sputum and anal swabs should be collected if necessary. Besides, imaging examinations such as CT should be performed to analyze and determine if the disease was caused by novel coronavirus infection. In case of COVID-19 infection, it shall be treated according to SAE, and the presence of ADE phenomenon was especially analyzed.

10.7.7 Report on Serious Adverse Events

(1) The responsible organization established an emergency plan for SAE. After the investigator was informed of the SAE, appropriate action should be taken for the subject and documented immediately. He should report the SAE to the sponsor within 24 hours after being informed, and immediately provide a detailed and written follow-up report. SAE reports and follow-up reports should indicate the subject identification code in the clinical trial, rather than the real name, citizenship number, address, and other identity information of the subjects.

For reports on death events, the investigator should report to the sponsor and the ethics committee in written form and timely provide other necessary materials, such as autopsy report and final medical report.

Upon the reception of safety information related to the clinical trial from the sponsor, the investigator should sign for confirming the reception and read the information within 24 hours, consider whether corresponding adjustment is necessary to the treatment of subjects, and promptly report to the ethics committee the suspicious and unexpected serious adverse reactions provided by the sponsor.

(2) The sponsor should analyze and assess vaccine safety information received from any source, including the severity, correlation with the test vaccine, and whether it is an unexpected event.

During drug clinical trial, the sponsor should report the suspected unexpected

serious adverse reaction (SUSAR) rapidly to all the investigators participating in the clinical trial and to the clinical trial institution and ethics committee; the sponsor should report SUSAR rapidly to the national drug regulatory department and health authority in the form of individual case safety report in accordance with the *Standards and Procedures for Rapid Reporting of Safety Data during Drug Clinical Trials*.

For suspicious and unexpected serious adverse reactions (SUSAR) resulting in death or threatening the life, the sponsor shall soon report them upon being first informed within 7 nature days and report related follow-up information in next 8 nature days (The day on which the applicant is first informed is Day 0). For SUSAR not resulting in death or threatening the life, the sponsor shall soon report them upon being first informed within 15 nature days. For information on other potentially serious safety risks, the sponsor should also report to the national drug evaluation agency as soon as possible, while a medical and scientific judgment should be made for each case. After the initial report, the sponsor shall continue to track SAE and report related new information or change information to the previous report in the form of follow-up reports within 15 days after receiving new information. The sponsor is not allowed to change the investigator's judgment of the correlation between SAE and the vaccine. In case of disagreement between the sponsor and the investigator, the opinions of the sponsor and the investigator should be showed in detail in the report, and reported according to the higher management requirements.

In exceptional cases, the investigator and sponsor should timely provide SAE related information and safety reports as required by regulatory authorities and the independent ethics committee.

(3) The contacts and contact information of the sponsor, the ethics committee and Jiangsu Medical Products Administration are as follows:

24h contact of Sinovac Life Sciences Co., Ltd.:

Wang Jiayi, Mobile: 18518337983； Fax: 010-82890408.

Contact of Jiangsu Provincial Center for Disease Control and Prevention:

Ba Lu, Mobile: 025-83759406； Fax: 025-83759406.

Jiangsu Medical Products Administration

Mobile: 025-83273714; Fax: 025-83273714.

10.7.8 Safety Evaluation Criteria

Solicited local adverse events, systemic adverse events and vital signs: the grading of solicited adverse events mainly refers to the Guidelines of the National Medical Products Administration for Adverse Event Classification Standards for Clinical Trials of Preventive Vaccines (2019)[14], as shown in the following table. Solicited adverse events and non-solicited adverse events of the same symptom are graded according to the following criteria.

Table 25 Grading of (Local) Adverse Events at Inoculation Site

	Grade 1	Grade 2	Grade 3	Grade 4
Pain	Having no or marginal effect on limb activity	Having an effect on limb activity	Having an effect on daily life	Loss of basic living skills or hospitalization
Induration * #	Diameter 2.5-＜5 cm or area 6.25-＜ 25 cm² and having no or marginal effect on daily life	Diameter 5-＜10 cm or area 25-＜100 cm² or having an effect on daily life	Diameter ≥10 cm or area ≥100 cm² or fester or secondary infection or phlebitis or sterile abscesses or wound drainage or having serious effect on daily life	Abscess, exfoliative dermatitis, dermal or deep tissue necrosis
Swelling #	Diameter 2.5-＜5 cm or area 6.25-＜ 25 cm² and having no or marginal effect on daily life	Diameter 5-＜10 cm or area 25-＜100 cm² or having an effect on daily life	Diameter ≥10 cm or area ≥100 cm² or fester or secondary infection or phlebitis or sterile abscesses or wound drainage or having serious effect on daily life	Abscess, exfoliative dermatitis, dermal or deep tissue necrosis
Vaccinal areola#	Diameter 2.5-＜5 cm or area 6.25-＜ 25 cm² and having no or marginal effect on daily life	Diameter 5-＜10 cm or area 25-＜100 cm² or having an effect on daily life	Diameter ≥10 cm or area ≥100 cm² or fester or secondary infection or phlebitis or sterile abscesses or wound drainage or having serious effect on daily life	Abscess, exfoliative dermatitis, dermal or deep tissue necrosis
Rash* #	Diameter 2.5-＜5 cm or area 6.25-＜25 cm² and having no or marginal effect on daily life	Diameter 5-＜10 cm or area 25-＜100 cm² or having an effect on daily life	Diameter ≥10 cm or area ≥100 cm² or fester or secondary infection or phlebitis or sterile abscesses or wound drainage or having serious effect on daily life	Abscess, exfoliative dermatitis, dermal or deep tissue necrosis
Pruritus	Pruritus at inoculation site, mitigated spontaneously or within 48 hours after treatment	Pruritus at inoculation site, not mitigated within 48 hours after treatment	Having an effect on daily life	NA

*Induration and rash: In addition to the grading and evaluation by measuring the diameter directly, the change of measurements should also be recorded.

#Induration and swelling, rash and vaccinal areola: The maximum measured diameter or area should be used; The grading and evaluation should be based on the function grade and actual measurements and indicators with a higher grade should be chosen.

Table 26 Grading of (Systemic) Adverse Events and Vital Signs Not at Inoculation Site

	Grade 1	Grade 2	Grade 3	Grade 4
Acute allergic reaction*	Local urticaria (blister), no treatment required	Local urticaria, requiring for treatment or mild angioedema, no treatment required	Extensive urticaria or angioedema requiring for treatment or mild bronchospasm	Allergic shock or life-threatening bronchospasm or laryngeal edema
Abnormal skin & mucous	Erythema/pruritus/ color change	Diffuse rash/maculopapule/xerosis cutis/desquamation	Herpes zoster/exudation/desquamation/ulceration	Exfoliative dermatitis (involving mucosa) or erythema multiforme or suspected Stevens-Johnsons syndrome
Diarrhoea	Mild or transient, 3 or 4 times per day, abnormal poop or mild diarrhea lasting less than a week	Moderate or persistent, 5 to 7 times per day, abnormal poop, or diarrhea lasting over 1 week	Over 7 times per day, abnormal poop, or bloody diarrhea, orthostatic hypotension, electrolyte imbalance, venous transfusion >2L indicated	Hypotensive shock, hospitalization indicated
Anorexia	Loss of appetite, but normal food intake	Loss of appetite, decreased food intake, but no significant weight loss	Loss of appetite, and significant weight loss	Need for intervention (such as tube feeding and parenteral nutrition)
Vomiting	1 - 2 times /24 hours and daily activities not affected	3 to 5 times /24 hours or limited activity	Over 6 times within 24 hours or requiring intravenous infusion	Hospitalization or other nutrition channels indicated due to hypotensive shock
Nausea	Transient (<24 hours) or intermittent, and basically normal food intake	Persistent nausea leads to reduced food intake (24-48 hours)	Persistent nausea leads to almost no food intake (>48 hours) or requiring intravenous infusion	Life-threatening (such as hypotensive shock)
Muscular pain (not at the inoculation site)	Daily activities not affected	Daily activities marginally affected	Severe muscle pain, and daily activities severely affected	Urgent intervention or hospitalization indicated
Headache	Daily activities not affected, and treatment not required	Transient, daily activities marginally affected, treatment or intervention probably required	Daily activities severely affected, treatment or intervention required	Refractory, urgent intervention or hospitalization required

	Grade 1	Grade 2	Grade 3	Grade 4
Cough	Transient, no treatment required	Continuous cough which can be treated effectively	Paroxysmal cough which can not be controlled by treatment	Urgent intervention or hospitalization indicated
Fatigue and weakness	Hypoergia <48 hours, no impact on activity	Hypoergia for 20% to 50% >48 hours, with slight impact on activity	Hypoergia for >50%, with heavy impact on activity	Incapable of taking care of oneself, and emergency treatment or hospitalization
**Vital signs**				
Fever (axillary temperature)	37.3～<38.0	38.0～<38.5	≥38.5	≥39.5, lasting over 3 days

The sign * indicates type I hypersensitivity

Laboratory indexes: The first step is to determine the clinical significance. When there are "abnormality and clinical significance", the grading mainly refers to the Guidelines of the National Medical Products Administration for Adverse Event Classification Standards for Clinical Trials of Preventive Vaccines (2019)[14], the Guidelines for Adverse Event Classification Standards for Clinical Trials of Preventive Vaccines (2005) (Only Creatinine) [15] and the Grading Criteria of the National Institute of Allergy and Infectious Diseases (NIAID) under the National Institutes of Health (NIH) for Clinical Assessment (Only Platelets)[16], as shown in the following table:

Table 27 Grading of Blood Routine Indexes

Indicators/grading	Grade 1	Grade 2	Grade 3	Grade 4
Leukocyte increase (WBC, 10^9/L)	11～<13	13～<15	15～<30	≥30
Leukocyte decrease (WBC, 10^9/L)	2.000～2.499	1.500～1.999	1.000～1.499	<1.000
Low hemoglobin (g/dL)-male	10.0～10.9	9.0～<10.0	7.0～<9.0	<7.0
Low hemoglobin (g/dL)-female	9.5～10.4	8.5～<9.5	6.5～<8.5	<6.5
Platelet (10^9/L)	75-99.999	50-74.999	20-49.999	<20

Table 28 Grading of Blood Biochemical Indexes

Indicators/grading	Grade 1	Grade 2	Grade 3	Grade 4
Liver function (ALT,AST)	1.25～<2.5 ×ULN	2.5～<5.0×ULN	5.0～<10×ULN	≥10×ULN
Increase of total bilirubin (mg/dL; μmol/L)	1.1～<1.6×ULN	1.6～<2.6×ULN	2.6～5.0×ULN	≥5.0×ULN

Creatinine (CR)	1.1～1.5×ULN	1.6～3.0×ULN	3.1～6×ULN	>6×ULN
Creatine phosphokinase (CPK)	1.25～＜1.5×ULN	1.5～＜3.0×ULN	3.0～＜10×ULN	≥10×ULN
Glu (mmol/L)	6.11～＜6.95	6.95～＜13.89	13.89～＜27.75	≥27.75

Note: The ULN refers to the upper limit of normal; blood glucose shall be measured on an empty stomach.

Table 29 Grading of Routine Urine Test Indexes

Indicators/grading	Grade 1	Grade 2	Grade 3	Grade 4
Urine protein (PRO) (Urine test strip)	1+	2+	3+ orhigher	NA
Urine glucose (Urine test strip)	Little - 1+ or ≤250mg	2+ or >250 - ≤500mg	＞2+ or >500mg	NA
RBC (microscopy) [Red blood cells /high power field (rbc/hpf) (excluding women's periods)]	6～＜10	≥10	gross hematuria, with or without blood coagulum; or barrel-type urinary erythrocyte; or treatment required	Urgent intervention or hospitalization indicated

Adverse events not included in above grading table should be graded and evaluated according to the following standards:

Grade 1 mild: short time (<48h) or slight discomfort; daily activities not affected, and treatment not required;

Grade 2 moderate: mild or moderate limited activity, medical attention probably required, treatment not required or mild treatment required;

Grade 3 severe: obvious limited activity, treatment required, hospitalization probably required;

Grade 4 critical: probably deadly, severe limited activity, monitoring and treatment required.

Grade 5: death

10.8 Concomitant Medication and Vaccination

10.8.1 Concomitant Medication

— If any adverse event (AE) occurs during the trial, the drug therapy and medical treatment should be allowed if necessary.

— In case of severe allergic reaction or life threatening events, first aid measures should be taken immediately.

— The investigator should record any concomitant medication information,

including name, dosage form, dosage, and duration of use.

10.8.2 Concomitant Vaccination

- Other vaccines can be administered at least 7 days after the test vaccine is administered.
- During the trial, subjects can be vaccinated with such vaccines as rabies vaccine and tetanus vaccine in case of emergency.
- Detailed information should be recorded, including the name of the vaccine, the use of the vaccine and the time of vaccination if concomitant vaccination.

10.9 Immunogenicity evaluation

Humoral immunity: Blood samples collected at different time points should be subject to neutralizing antibody test, IgG test and IgM test (IgG and IgM tests are only applicable to Phase I clinical trial.) The positive conversion rate and positive rate of neutralizing antibody, GMT, GMI, and the positive rate of IgG and IgM antibodies should be calculated.

Cellular immunity (applicable to Phase I clinical trial): Blood samples collected at different time points should be subject to IFN-γ secretory reaction test of specific T cells to calculate the positive rate.

10.9.1 Evaluation Standards

- Evaluation standards for serum neutralizing antibody

  The evaluation standards for positive serum antibody are as follows:

  - If antibody titer≥1:8, it is positive.

  The evaluation standards for the positive conversion of serum antibody are as follows:

  - If neutralizing antibody titer is less than 1:8 before immunization and no less than 1:8 after immunization, the positive conversion of antibody is considered. Or if neutralizing antibody is no less than 1:8 before immunization and neutralizing antibody titer increases 4 times after immunization, the positive conversion of antibody is considered.

- Evaluation standards for IgG and IgM

See kit instructions.

10.9.2 Laboratory Test Methods

- Serum antibody test:
- Neutralizing antibody test - micro neutralization test;
- IgG/IgM test - Enzyme-Linked Immune-sorbent Assay (ELISA)
- IFN-γ secretory reaction test of specific T cells: Enzyme-Linked Immune Absorbent Spot (ELISPOT) (mononuclear cell)

10.10 Data Management

10.10.1 Original Data

The original data should include the informed consent form, diary cards and original record books. The following basic data should be recorded.

- Test name and subject's code
- Demographic data
- Inclusion/exclusion criteria
- Vaccination record
- Follow-up date and date when the subject stops the test
- Adverse event/reaction and its processing and outcome
- Accompanying medical treatment and other vaccinations

All data should be recorded in the original form, and stored in a special room properly by the investigator. The original data will be filed in the research center, because it can prove that the subject has participated in the clinical trial and the data is true and complete.

The investigator should make original records in an earnest, accurate and timely manner, and all collected original data shall be recorded on the day when the original data are acquired. A black sign pen should be used. In case of any writing error, cross out the wrong words and write correct ones next to the wrong words while signing and giving the date.

10.10.2 Case Report Form (CRF)

In this trial, "Electronic Data Capture (EDC)" is used to establish the electronic

CRF. As an important component of the clinical trials and research reports, the electronic CRF is used to record clinical trial data. Information should be inputted with standard language according to the EDC instructions and CRF filling instructions.

The data on the electronic CRF should be derived from and consistent with the original data. The input, verification, modification, cleanup and quality control of any electronic CRF data will be recorded in the EDC system. Upon completion of the data cleanup, the investigator should confirm the data in each electronic CRF and create an electronic signature for each electronic CRF.

Only the investigator and authorized staff will be allowed to access the EDC system during the trial.

10.10.3 Data Locking

After input, verification and cleanup of all data, the final data verification is carried out. According to the evaluation criteria, the analyzed population should be determined, and the situations that deviate from the schedule as well as their impact on data group analysis should be confirmed. Then, the database should be locked.

10.10.4 Privacy Protection for Subjects and Data Utilization Range

Any information regarding the identity of the subject will be confidential and the name will not appear in any publication or report of the study. Study records will be made available to the sponsor‘s representatives in the presence of the investigator for the purpose of medical data collection. Besides, the monitor and inspector of the study and the representatives from Vaccine Clinical Trial Ethics Committee, Jiangsu Provincial Center for Disease Control and Prevention and National Medical Products Administration (NMPA) can, as required, review the subjects' original data related to the study to confirm the accuracy of the data collected in the study. The original data obtained in this study will only be used for publication of papers or results related to this project.

10.11 Statistical Analysis

10.11.1 Analysis Set

10.11.1.1 Phase I clinical trial

(1) Full Analysis Set, FAS

The Intent to Treat (ITT) principle is followed, including all subjects who were randomized to groups, are given at least 1 dose, have the blood collected at least once before and after immunization and have effective antibody titer values. Among them, the subjects who were incorrectly vaccinated were evaluated for immunogenicity according to ITT principle and the original random grouping.

(2) Per protocol set (PPS) on Day 14 after second dose of immunization

It is a subset of FAS, including all subjects who follow the inclusion/exclusion criteria, are randomized into groups, are given the first and second doses of vaccination within the time window period according to the protocol, have the blood collected on Day 14 before immunization and after the second dose of immunization, and have effective antibody titer values. Among them, subjects who meet the following conditions cannot enter PPS:

- Violate the trial protocol;
- The wrong vaccination number;
- subjects that are given the second dose of vaccination or have the blood collected on Day 14 after the second dose of immunization out of visit window.
- Use vaccines or drugs prohibited by the protocol:

  ①Other research or unregistered products (drugs or vaccines) that are not research vaccine

  ②Long-term use (lasting for more than 14 days) of immunosuppressants or other immunomodulatory drugs (inhaled or topical steroids are allowed);

  ③Immunoglobulin and/or blood preparations;
- Newly diagnosed autoimmune diseases, including human immunodeficiency virus (HIV) infection;
- Other conditions affecting the evaluation of vaccine immunogenicity on Day 14 after second dose of immunization.

(3) Per protocol set 2B (PPS–2B) on Day 28 after second dose of immunization

It is a subset of FAS, including all subjects who follow the inclusion/exclusion criteria, are randomized into groups, are given the first and second doses of vaccination within the time window period according to the protocol, have the blood collected on Day 28 before immunization and after the second dose of immunization, and have effective antibody titer values. Among them, subjects who meet the following conditions cannot enter PPS:

- Violate the trial protocol;
- The wrong vaccination number;
- subjects that are given the second dose of vaccination or have the blood collected on Day 14 after the second dose of immunization out of visit window.
- Use vaccines or drugs prohibited by the protocol:

  ①Other research or unregistered products (drugs or vaccines) that are not research vaccine

  ②Long-term use (lasting for more than 14 days) of immunosuppressants or other immunomodulatory drugs (inhaled or topical steroids are allowed);

③Immunoglobulin and/or blood preparations;

- Newly diagnosed autoimmune diseases, including human immunodeficiency virus (HIV) infection;
- Other conditions affecting the evaluation of vaccine immunogenicity on Day 14 after second dose of immunization.

(4) Immune persistence set (IPS-6)

Include all subjects who have received full-course vaccination and have blood collected 6 months after full-course immunization and have effective antibody titer values.

(5) Safety set (SS)

Include all subjects randomized in groups and given at least one dose of vaccine. Among them, according to ASaT (All Subjects As Treated) principle, the safety evaluation of subjects with wrong vaccination is carried out according to the actual vaccination group of subjects.

The safety dataset is divided into total safety dataset, first dose safety set and second dose safety set. The safety of each dose is analyzed based on the actual number of people vaccinated each time. The first dose safety set includes all subjects who have given the first dose of vaccination, which is recorded as SS1; the second dose safety set includes subjects who have given the second dose of vaccination, which is recorded as SS2.

10.11.1.2 Phase II clinical trial

Immunogenicity and persistence analysis set

<u>Day 0, Day 14, Day 42 immunization schedule</u>

(1) Full Analysis Set, FAS

The Intent to Treat (ITT) principle is followed, including all subjects who were randomized to groups, are given at least 1 dose, have the blood collected at least once before and after immunization and have effective antibody titer values. Among them, the subjects who were incorrectly vaccinated were evaluated for immunogenicity according to ITT principle and the original random grouping.

(2) Per protocol set (PPS) on Day 14 after second dose of immunization

It is a subset of FAS, including all subjects who follow the inclusion/exclusion criteria, are randomized into groups, are given the first and second doses of vaccination within the time window period according to the protocol, have the blood collected on Day 14 before immunization and after the second dose of immunization, and have effective antibody titer values. Among them, subjects who meet the following conditions cannot enter PPS:

- Violate the trial protocol;
- The wrong vaccination number;
- subjects that are given the second dose of vaccination or have the blood collected on Day 14 after the second dose of immunization out of visit window.
- Use vaccines or drugs prohibited by the protocol:

①Other research or unregistered products (drugs or vaccines) that are not

research vaccine

②Long-term use (lasting for more than 14 days) of immunosuppressants or other immunomodulatory drugs (inhaled or topical steroids are allowed);

③Immunoglobulin and/or blood preparations;

- Newly diagnosed autoimmune diseases, including human immunodeficiency virus (HIV) infection;
- Other conditions affecting the evaluation of vaccine immunogenicity on Day 14 after second dose of immunization.

(3) Per protocol set 2B (PPS–2B) on Day 28 after second dose of immunization

It is a subset of FAS, including all subjects who follow the inclusion/exclusion criteria, are randomized into groups, are given the first and second doses of vaccination within the time window period according to the protocol, have the blood collected on Day 28 before immunization and after the second dose of immunization, and have effective antibody titer values. Among them, subjects who meet the following conditions cannot enter PPS:

- Violate the trial protocol;
- The wrong vaccination number;
- Subjects that are given the second dose of vaccination or have the blood collected on Day 28 after the second dose of immunization out of visit window.
- Use vaccines or drugs prohibited by the protocol:

①Other research or unregistered products (drugs or vaccines) that are not research vaccine

②Long-term use (lasting for more than 14 days) of immunosuppressants or other immunomodulatory drugs (inhaled or topical steroids are allowed);

③Immunoglobulin and/or blood preparations;

- Newly diagnosed autoimmune diseases, including human immunodeficiency virus (HIV) infection;
- Other conditions affecting the evaluation of vaccine immunogenicity on Day 28 after second dose of immunization.

(4) Per protocol set (PPS3) on Day 28 after third dose of immunization

It is a subset of FAS, including all subjects who follow the inclusion/exclusion criteria, are randomized into groups, are given the third dose of vaccination within the time window period according to the protocol, have the blood collected after immunization, and have effective antibody titer values. Among them, subjects who meet the following conditions cannot enter PPS:

- Violate the trial protocol;
- The wrong vaccination number;
- Subjects that are given the second and third doses of vaccination or have the blood collected on Day 28 after the third dose of immunization out of visit window.
- Use vaccines or drugs prohibited by the protocol:

①Other research or unregistered products (drugs or vaccines) that are not research vaccine

②Long-term use (lasting for more than 14 days) of immunosuppressants or other immunomodulatory drugs (inhaled or topical steroids are allowed);

③Immunoglobulin and/or blood preparations;

- Newly diagnosed autoimmune diseases, including human immunodeficiency virus (HIV) infection;
- Other conditions affecting the evaluation of vaccine immunogenicity on Day 28 after third dose of immunization.

(5) Immune persistence set 6B (IPS-6B)

Include all subjects who have completed the full-course vaccination according to Day 0, Day 14, Day 42 immunization schedule, have blood collected 6 months after full-course immunization, and have effective antibody titer values.

(6) Immune persistence set 12 (IPS-12)

Include all subjects who have completed the full-course vaccination according to Day 0, Day 14, Day 42 immunization schedule, have blood collected 12 months after full-course immunization, and have effective antibody titer values.

Day 0, Day 14 immunization schedule (+6-moth booster immunization)

(1) Full Analysis Set, FAS

The Intent to Treat (ITT) principle is followed, including all subjects who were randomized to groups, are given at least 1 dose, have the blood collected at least once before and after immunization and have effective antibody titer values. Among them, the subjects who were incorrectly vaccinated were evaluated for immunogenicity according to ITT principle and the original random grouping.

(2) Per protocol set (PPS) on Day 14 after the second dose of immunization at the primary immunization stage

It is a subset of FAS, including all subjects who follow the inclusion/exclusion criteria, are randomized into groups, are given the first and second doses of vaccination within the time window period according to the protocol, have the blood collected on Day 14 before immunization and after the second dose of immunization, and have effective antibody titer values. Among them, subjects who meet the following conditions cannot enter PPS:

- Violate the trial protocol;
- The wrong vaccination number;
- subjects that are given the second dose of vaccination or have the blood collected on Day 14 after the second dose of immunization out of visit window.
- Use vaccines or drugs prohibited by the protocol:

①Other research or unregistered products (drugs or vaccines) that are not research vaccine

②Long-term use (lasting for more than 14 days) of immunosuppressants or other immunomodulatory drugs (inhaled or topical steroids are allowed);

③Immunoglobulin and/or blood preparations;

- Newly diagnosed autoimmune diseases, including human immunodeficiency virus (HIV) infection;
- Other conditions affecting the evaluation of vaccine immunogenicity on Day

14 after second dose of immunization.

(3) Per protocol set 2B (PPS-2B) on Day 28 after the second dose of immunization at the primary immunization stage

It is a subset of FAS, including all subjects who follow the inclusion/exclusion criteria, are randomized into groups, are given the first and second doses of vaccination within the time window period according to the protocol, have the blood collected on Day 28 before immunization and after the second dose of immunization, and have effective antibody titer values. Among them, subjects who meet the following conditions cannot enter PPS:

- Violate the trial protocol;
- The wrong vaccination number;
- Subjects that are given the second dose of vaccination or have the blood collected on Day 28 after the second dose of immunization out of visit window.
- Use vaccines or drugs prohibited by the protocol:

  ①Other research or unregistered products (drugs or vaccines) that are not research vaccine

  ②Long-term use (lasting for more than 14 days) of immunosuppressants or other immunomodulatory drugs (inhaled or topical steroids are allowed);

  ③Immunoglobulin and/or blood preparations;
- Newly diagnosed autoimmune diseases, including human immunodeficiency virus (HIV) infection;
- Other conditions affecting the evaluation of vaccine immunogenicity on Day 28 after second dose of immunization.

(4) Immune persistence set 6A (IPS-6A) for primary immunization

Include all subjects who have received primary vaccination according to Day 0, Day 14 immunization schedule, have blood sampling 6 months after primary immunization, and have effective antibody titer values.

(5) Full analysis set for booster (bFAS)

The Intent to Treat (ITT) principle is followed, including all subjects who are randomized to groups, receive booster immunization after primary immunization, are given booster vaccine, have blood collected at least once before and after booster immunization and have effective antibody titer values. Among them, the subjects who were incorrectly vaccinated were evaluated for immunogenicity according to ITT principle and the original random grouping.

(6) Per Protocol Set (bPPS) on Day 14 after booster immunization

It is a subset of FAS, including all subjects who follow the inclusion/exclusion criteria, are randomized into groups, have received primary immunization and 6-month booster immunization during the time window period according to the protocol, have the blood collected on Day 14 before and after booster immunization, and have effective antibody titer values. Among them, subjects who meet the following conditions cannot enter PPS:

- Violate the trial protocol;
- The wrong vaccination number;
- Subjects that receive booster immunization or have the blood collected on Day 14 after booster immunization out of visit window.

- Use vaccines or drugs prohibited by the protocol:

  ①Other research or unregistered products (drugs or vaccines) that are not research vaccine

  ②Long-term use (lasting for more than 14 days) of immunosuppressants or other immunomodulatory drugs (inhaled or topical steroids are allowed);

  ③Immunoglobulin and/or blood preparations;
- Newly diagnosed autoimmune diseases, including human immunodeficiency virus (HIV) infection;
- Other conditions affecting the evaluation of vaccine immunogenicity on Day 14 after booster immunization.

(7) Immune persistence set 6 for booster (bIPS-6)

Include all subjects who have received primary vaccination according to Day 0, Day 14 immunization schedule, received booster immunization 6 months after primary immunization, have blood collected 6 months after booster immunization, and have effective antibody titer values.

Day 0, Day 28, Day 56 immunization schedule

(1) Full Analysis Set, FAS

The Intent to Treat (ITT) principle is followed, including all subjects who were randomized to groups, are given at least 1 dose, have the blood collected at least once before and after immunization and have effective antibody titer values. Among them, the subjects who were incorrectly vaccinated were evaluated for immunogenicity according to ITT principle and the original random grouping.

(2) Per protocol set 2B (PPS-2B) on Day 28 after second dose of immunization

It is a subset of FAS, including all subjects who follow the inclusion/exclusion criteria, are randomized into groups, are given the first and second doses of vaccination within the time window period according to the protocol, have the blood collected on Day 28 before immunization and after the second dose of immunization, and have effective antibody titer values. Among them, subjects who meet the following conditions cannot enter PPS:

- Violate the trial protocol;
- The wrong vaccination number;
- Subjects that are given the second dose of vaccination or have the blood collected on Day 28 after the second dose of immunization out of visit window.
- Use vaccines or drugs prohibited by the protocol:

  ①Other research or unregistered products (drugs or vaccines) that are not research vaccine

  ②Long-term use (lasting for more than 14 days) of immunosuppressants or other immunomodulatory drugs (inhaled or topical steroids are allowed);

  ③Immunoglobulin and/or blood preparations;
- Newly diagnosed autoimmune diseases, including human immunodeficiency virus (HIV) infection;

- Other conditions affecting the evaluation of vaccine immunogenicity on Day 28 after second dose of immunization.

(3) Per protocol set 3 (PPS3) on Day 28 after third dose of immunization

It is a subset of FAS, including all subjects who follow the inclusion/exclusion criteria, are randomized into groups, are given the third dose of vaccination within the time window period according to the protocol, have the blood collected on Day 28 after immunization, and have effective antibody titer values. Among them, subjects who meet the following conditions cannot enter PPS:

- Violate the trial protocol;
- The wrong vaccination number;
- Subjects that are given the second and third doses of vaccination or have the blood collected on Day 28 after immunization out of visit window.
- Use vaccines or drugs prohibited by the protocol:

  ①Other research or unregistered products (drugs or vaccines) that are not research vaccine

  ②Long-term use (lasting for more than 14 days) of immunosuppressants or other immunomodulatory drugs (inhaled or topical steroids are allowed);

  ③Immunoglobulin and/or blood preparations;
- Newly diagnosed autoimmune diseases, including human immunodeficiency virus (HIV) infection;
- Other conditions affecting the evaluation of vaccine immunogenicity on Day 28 after third dose of immunization.

(4) Immune persistence set 6B (IPS-6B)

Include all subjects who have completed the full-course vaccination according to Day 0, Day 28, Day 56 immunization schedule, have blood collected 6 months after full-course immunization, and have effective antibody titer values.

(5) Immune persistence set 12 (IPS-12)

Include all subjects who have completed the full-course vaccination according to Day 0, Day 28, Day 56 immunization schedule, have blood collected 12 months after full-course immunization, and have effective antibody titer values.

Day 0, Day 28 immunization schedule (+6-moth booster immunization)

(1) Full Analysis Set, FAS

The Intent to Treat (ITT) principle is followed, including all subjects who were randomized to groups, are given at least 1 dose, have the blood collected at least once before and after immunization and have effective antibody titer values. Among them, the subjects who were incorrectly vaccinated were evaluated for immunogenicity according to ITT principle and the original random grouping.

(2) Per protocol set 2B (PPS-2B) on Day 28 after the second dose of immunization at the primary immunization stage

It is a subset of FAS, including all subjects who follow the inclusion/exclusion criteria,

are randomized into groups, are given the first and second doses of vaccination within the time window period according to the protocol, have the blood collected on Day 28 before immunization and after the second dose of immunization, and have effective antibody titer values. Among them, subjects who meet the following conditions cannot enter PPS:

- Violate the trial protocol;
- The wrong vaccination number;
- Subjects that are given the second dose of vaccination or have the blood collected on Day 28 after the second dose of immunization out of visit window.
- Use vaccines or drugs prohibited by the protocol:

  ①Other research or unregistered products (drugs or vaccines) that are not research vaccine

  ②Long-term use (lasting for more than 14 days) of immunosuppressants or other immunomodulatory drugs (inhaled or topical steroids are allowed);

  ③Immunoglobulin and/or blood preparations;

- Newly diagnosed autoimmune diseases, including human immunodeficiency virus (HIV) infection;
- Other conditions affecting the evaluation of vaccine immunogenicity on Day 28 after second dose of immunization.

(3) Immune persistence set 6A (IPS-6A) for primary immunization

Include all subjects who have received primary vaccination according to Day 0, Day 28 immunization schedule, have blood collected 6 months after primary immunization, and have effective antibody titer values.

(4) Full analysis set for booster (bFAS)

The Intent to Treat (ITT) principle is followed, including all subjects who are randomized to groups, receive booster immunization after primary immunization, are given booster vaccine, have blood collected at least once before and after booster immunization and have effective antibody titer values. Among them, the subjects who were incorrectly vaccinated were evaluated for immunogenicity according to ITT principle and the original random grouping.

(5) Per Protocol Set (bPPS) on Day 28 after booster immunization

It is a subset of FAS, including all subjects who follow the inclusion/exclusion criteria, are randomized into groups, have received primary immunization and 6-month booster immunization during the time window period according to the protocol, have the blood collected on Day 28 before and after booster immunization, and have effective antibody titer values. Among them, subjects who meet the following conditions cannot enter PPS:

- Violate the trial protocol;
- The wrong vaccination number;
- Subjects that receive booster immunization or have the blood collected on Day 28 after booster immunization out of visit window.
- Use vaccines or drugs prohibited by the protocol:

  ①Other research or unregistered products (drugs or vaccines) that are not research vaccine

②Long-term use (lasting for more than 14 days) of immunosuppressants or other immunomodulatory drugs (inhaled or topical steroids are allowed);

③Immunoglobulin and/or blood preparations;

- Newly diagnosed autoimmune diseases, including human immunodeficiency virus (HIV) infection;
- Other conditions affecting the evaluation of vaccine immunogenicity on Day 28 after booster immunization.

(6) Immune persistence set 6 for booster (bIPS-6)

Include all subjects who have received primary vaccination according to Day 0, Day 28 immunization schedule, received booster immunization 6 months after primary immunization, have blood collected 6 months after booster immunization, and have effective antibody titer values.

Safety Set

Include all subjects randomized in groups and given at least one dose of vaccine. Among them, according to ASaT (All Subjects As Treated) principle, the safety of subjects with wrong vaccination was evaluated according to the actual vaccination group of subjects. The safety dataset is divided into total safety dataset and safety sets for doses. The safety of each dose is analyzed based on the actual number of people vaccinated each time.

10.11.2 Statistical Analysis Method

10.11.2. General principles

The measurement data are statistically described with average, standard deviation, median, maximum and minimum; the enumeration data or ranked data are presented by frequency and relative frequency.

All statistical analysis will be completed with the help of the statistical software SAS 9.4.

10.11.2.2 Characteristics of Subjects

The number of subjects who are screened and enrolled into each group and complete the test and the number of subjects in each analysis set are summarized; and the reasons for subjects' drop-out are analyzed. The list of subjects who fail in screening, the list of withdrawal subjects and the list of subjects who do not enter each analysis set are listed respectively.

10.11.2.3 Immunogenicity evaluation

The positive conversion rate and positive rate of antibodies after immunization in

medium-dose group, high-dose group and placebo group are calculated respectively. The bilateral 95% confidence interval is calculated by Clopper-Pearson. Chi-square test/Fisher exact probability test is used to statistically test the differences between groups.

The GMT and GMI of antibodies after immunization in the test group and control group are calculated by geometric mean and 95% confidence interval, and logarithmic analysis of variance is used to statistically test the differences between groups.

The positive rates of IgG and IgM antibodies and IFN-γ secretion by specific T cells after immunization are analyzed by the same statistical method as the positive rate of neutralizing antibodies.

10.11.2.4 Safety Evaluation

Adverse events are medically coded by MedDRA. In this trial, the treatment emergent adverse events are statistically analyzed, and the adverse events occurring before inoculation are listed in the form of a list.

The times, number and incidence of all adverse events, adverse events related to the research vaccine and adverse events unrelated to the research vaccine are calculated for each group. Fisher exact probability test is used to statistically compare the differences between the groups. The severity, dose and occurrence time of adverse events and adverse events related to vaccine are subject to statistical analysis. Make a list of adverse events related to research vaccine and a list of adverse events unrelated to research vaccine. The adverse events after vaccination with each dose are statistically analyzed. Adverse events for each dose will be analyzed based on the safety set for each dose.

The times, number and incidence of all serious adverse events, serious adverse events related to research vaccine, and serious adverse events unrelated to research vaccine are calculated for each group respectively. Fisher exact probability test should be used to statistically compare the differences between the groups. Make a list of serious adverse events.

The changes of blood routine, blood biochemical and urine routine indexes before

and after inoculation are described statistically. The changes in the clinical significance of blood routine, blood biochemical and urine routine indexes are statistically described in the form of cross table before and after inoculation.

10.11.2.5 Processing of missing data

In the statistical analysis of the full analysis set, for those with missing serum test results after immunization, the method of last observation carried forward (LOCF) is used to fill the data. For those with missing serum results before immunization but after immunization, the maximum value of the serum antibody before immunization is used to fill the value of antibody after immunization of all subjects and the corresponding immunogenicity endpoint is further derived and calculated. Missing data in exploratory endpoints and safety endpoints are not processed in this trial.

11 Clinical Trial Monitoring

11.1 Sponsor’s Responsibility

The sponsor should execute and maintain the quality assurance and control system and prepare quality management documents to make sure that the test is executed according to regulations. Meanwhile, data, record and report should meet the requirements of GCP and other regulations.

11.2 Investigator’s Responsibility

The main investigator should manage and clearly divide the roles of all participants in the clinical trial. The investigator should keep the subject’s individual data secret. The document provided to the sponsor should be identified only with the subject identification code and subject number. The investigator keeps a list of subjects' identifications in the investigator's file. In accordance with GCP principle, each subject’s original data is allowed to be monitored, audited, and reviewed.

11.3 Personnel Training

Before the start of the test, the sponsor and the principal investigator should conduct training to the participants in the form of a meeting. The training should include: clinical trial protocol, trial procedures, time arrangement, operational precautions,

filling of trial data, etc. If new monitors or investigators participate in the trial, they should be trained separately. Retraining can be conducted if the sponsor or principal investigator deems it necessary. Records should be made for each training.

11.4 Compliance Guaranteeing of Subjects

According to the clinical trial protocol, a concise and well-organized volunteer recruitment letter and an informed consent form are prepared.

Responsible physicians should be told to communicate with volunteers in plain language so as to make subjects fully informed.

Subjects are screened strictly according to the criteria for inclusion and exclusion.

Follow-up personnel should have a high sense of responsibility and professionalism. Training is required to improve their communication skills and affinity. In the process of safety follow-up, measures should be taken to ensure effective contact between subjects and investigators and timely deal with adverse reactions found. Subjects should be provided with relevant health consultation.

11.5 Reported on Protocol Deviations/Violations

The on-site investigator authorized by the principal investigator should immediately report to the principal investigator after finding any deviations/violations of the clinical trial protocol or receiving any report on deviations/violations of the clinical trial protocol. Stop the current protocol deviations/violations (excluding beyond-period inoculation/sample collection); for the protocol deviations/violations that have occurred, wait for the written/e-mail reply from the sponsor and the principal investigator.

11.6 Management of Test Vaccine

11.6.1 Definition and Treatment of Cold Chain Damage

Once the refrigerator storing vaccine shows the temperature of <2°C or >8°C, it should be recorded as cold chain damage. Upon the cold chain damage, the investigator should transfer the vaccine to a dark place at the temperature of 2-8°C as soon as possible, timely report it to the sponsor, and then decide to stop or continue using vaccine in accordance with the written opinions of /e-mail reply by the sponsor.

11.6.2 Acceptance of Test Vaccine

The sponsor sends the vaccines used in clinical trial to the test site. The investigator must sign the vaccine acceptance receipt which should briefly show information about the received vaccine (completeness of the package and normal indication of the cold chain system).

When the investigator finds that the vaccine package is damaged, that the vaccine goes bad or that there is any blocky substance that cannot be shaken, the vaccine cannot be used and should be returned to the sponsor. If the cold chain system is damaged during transportation and storage or the vaccine is frozen, the vaccine cannot be used, and should be stored separately, marked with "×" on the outer package, managed by the designated person, and returned to the sponsor.

11.6.3 Management of Test Vaccine

The test vaccine should be managed by the designated person and monitored by the monitor. The Warehouse Standing Book for Vaccines should include the amount of vaccines received, the amount of vaccines given to subjects, the amount of vaccines left, or the amount of vaccines lost. The investigators will count all the test vaccines at the end of the trial. When research is finished, the remaining test vaccines are counted and returned to the sponsor.

11.7 Sample Management in Clinical Trials

Specimens for routine blood, blood biochemistry, urine routine, inflammatory factors, anti-nuclear antibody and T-cell reaction tests are disposed of by the testing organization as medical waste after test. The backup serum should be temporarily kept by the test organization on site until the test organization issues the immunogenicity test report which should be verified correct. The backup serum can be stored or processed by the sponsor after the project is over. If backup serum is required, the approval from the independent ethics committee and the consent from the subject should be obtained.

11.8 Storage of Data on Clinical Test

Data in the clinical trial must be stored as per the requirements in GCP Appendix

2. The investigator should retain the clinical trial data for at least 5 years after the clinical trial ends. The sponsor should maintain clinical trial data for at least 5 years after the marketing of the drug.

11.9 Finished Criteria for Clinical Trial

- The test samples are sent to the corresponding test organization, and the test report should be issued;
- All subjects complete the required visits, and the original data and documents of the clinical trial are handed over to the archivist for archiving and preservation;
- The remaining number of test vaccines is accurate and the remaining test vaccines are handed over to the sponsor;
- The statistical analysis report and summary report meet the requirements of the sponsor.

12 Ethical Approval

12.1 Review and Approval

The clinical trial protocol should be approved by the local independent ethics committee. The principal investigator submits the clinical protocol and all necessary additional documentation to the independent ethics committee, After the approval of the committee, the investigator provides the sponsor with a certificate of approval from the committee.

12.2 Field Supervision

Throughout the trial, the independent ethics committee should supervise if there's any ethical damage to the subjects and if the subjects obtain treatment or compensation and corresponding medical insurance measures when they are badly influenced by the study. What's more, the independent ethics committee should evaluate the risks borne by the subjects.

12.2.1 Informed Consent and Informed Consent Form

Ensure that the subject enrollment method and the relevant data provided to

subjects are comprehensive and understandable, and that the method of obtaining informed consent is appropriate. Throughout the trial, the independent ethics committee should periodically review the progress of the trial and assess the risks and benefits of the subjects.

12.2.2 Potential Risks and Risk Minimization

If an adverse reaction is identified as associated with the vaccination (abscess at vaccination site and rash after vaccination), treatment will be provided timely for subjects in accordance with relevant provisions. In case of life-threatening event, the subject will be escorted to the hospital for treatment immediately and report should be made.

Under strict supervision, the trained, experienced medical personnel could carry out vaccination and collect venous blood in accordance with the rules and procedures, so as to minimize the pain of the subject suffering from vaccination and blood collection (including pain and rare local infection in vein puncture site).

12.2.3 Protection Measures for Subjects

The clinical trial should be performed in centers for disease control and prevention with vaccination qualification at county or municipal level. The sponsor should assess the study site in strict accordance with the GCP requirements prior to the start of the trial. The environmental and facilities of the test site should meet the requirements stipulated in Guidelines for Quality Management in Clinical Trial of Vaccines (Trial). An emergency plan for prevention and handling of damages and emergencies of subjects should be made on test site. In physical examination room and blood collection room, qualified and experienced doctors and nurses should be in place to strictly follow the inclusion/exclusion criteria and to collect blood smoothly. Proper first-aid facilities, equipment and medicines should be available in the emergency room and emergency physicians should be qualified and competent. After adverse events occur at test site, subjects should be treated immediately in an emergency room on the site and sent to contracting hospitals by ambulances on site after the condition is stable if emergency hospitalization is required. Ambulances should be equipped with the necessary first aid

facilities and drugs. The trial site should sign a Green Channel Agreement with local county-level or higher general hospitals. During the enrollment of subjects, contracting hospitals should be notified for timely treatment. Staff responsibilities should be clarified. Contact numbers and rescue routes should be available to ensure the timely treatment of sudden adverse events and the effective contact between the subject and investigator so that any adverse events are promptly reported and dealt with. When subjects experience serious adverse events and need to be hospitalized for emergency treatment, contracting hospitals can provide green channel services including medical treatment, hospitalization and medical security to ensure that subjects can be treated in time. The investigator follows the progress of the events and completes the investigation records until the end of the serious adverse events.

12.3 Confidentiality

The subjects' privacy should be kept during the study and the collection of biological samples, as well as during the reporting and publication of the study. Only subject code, sample number, collection time and test index are recorded for the test sample. It is strictly restricted that only principal test personnel can obtain electronic and printed copies.

13 Modification of Clinical Trial Protocol

After the sponsor and investigator have signed the clinical trial protocol, if there are any modifications to the protocol, all protocols modified should be re-signed and dated by the principal investigator and sponsor, with the protocols not modified attached.

All modified protocols should be reported to the independent ethics committee and approved by the independent ethics committee before being executed. When a protocol is modified, it should be pointed out whether it is necessary to modify the informed consent and electronic CRF form.

14 Disclosure and Publication of Data

After the completion of this clinical trial, if the results of the trial need to be

disclosed and/or published, the positive results will be disclosed and/or published together with the negative results.

15 References

1. The former China Food and Drug Administration, Provisions of Drug Registration, 2007.
2. The former China Food and Drug Administration, Good Clinical Practice, 2003.
3. The former China Food and Drug Administration, Technical Guidelines for Clinical Trial of Vaccines, 2004.
4. The former China Food and Drug Administration, Guidelines for Quality Management in Clinical Trial of Vaccines (Trial), 2013.
5. Center for Drug Evaluation. Technical Guidelines for Research and Development of Vaccines for Prevention of COVID-19 (Trial). March 2020.
6. Lu R, Zhao X, Li J, Niu P, Yang B, Wu H, Wang W, Song H, Huang B, Zhu N, et al. Genomic characterisation and epidemiology of 2019 novel coronavirus: implications for virus origins and receptor binding. Lancet (London, England). 2020; 6736(20): 1-10.
7. Chen Y, Liu Q, Guo D. Coronaviruses: genome structure, replication, and pathogenesis. Journal of medical virology. 2020: 0-2.
8. Information Office of the State Council, Press Conference on Joint Prevention and Control of Novel Coronavirus–Infected Pneumonia. Beijing, January 26, 2020.
9. China's Novel Coronavirus Pneumonia Diagnosis and Treatment Plan (Provisional 7th Edition)
10. Holshue ML, DeBolt C, Lindquist S, et al. First Case of 2019 Novel Coronavirus in the United States. N Engl J Med. 2020 Mar 5; 382(10): 929-936. .
11. Special Expert Group for Control of the Epidemic of Novel Coronavirus Pneumonia of the Chinese Preventive Medicine Association. An update on the epidemiological characteristics of novel coronavirus pneumonia (COVID-19). Chinese Journal of Epidemiology, 02/14/2020.
12. https: //www.who.int/emergencies/diseases/novel-coronavirus-2019/situation-reports.
13. Department Of Diseases Control, National Health Commission of People's Republic of China. Joint Investigation Report of China-WHO on Novel Coronavirus Pneumonia (COVID-19).
14. National Medical Products Administration, Guidelines for Adverse Event Classification Standards for Clinical Trials of Preventive Vaccines, 2019.
15. The former National Medical Products Administration Institute, Guidance on Graded Standard of Adverse Effect in Clinical Trial for Prevention, 2005.
16. Division of microbiology and infections diseases (DMID) adults toxicity table. November 2007. NIH.

Project Title: Clinical Study of Phase I/II of SARS-CoV-2 Vaccine (Vero cell), Inactivated in Elderly Population

Protocol Title: A Randomized, Double-blind, Placebo-controlled Clinical Trial, to Evaluate Safety and Immunogenicity of Inactivated SARS-CoV-2 Vaccine (Vero cell), in Healthy Elderly Aged 60 Years and above

Name of Investigational Product: SARS-CoV-2 Vaccine (Vero cell), Inactivated

Sponsor: Sinovac Life Sciences Co., Ltd.

Study Institution: Hebei Provincial Center for Disease Control and Prevention

Statistical Company: Beijing Key Tech Statistics Technology Co., Ltd.

Protocol No.: PRO-nCOV-1002

Protocol Version Date: February 08, 2021

Version No.: 1.4

Protocol Approver: Qiang GAO

Signature of the Approver:

Approval Date:

北京科兴中维生物技术有限公司
SINOVAC RESEARCH & DEVELOPMENT CO., LTD.

Signature of thePrincipal Investigator

I agree to:

- Take the responsibility to correctly guide the conduct of the clinical study in the region.
- Ensure that thisstudy can be conducted in accordance with the trial protocoland the standard operating procedures for clinical studies
- Ensure that the personnel involved in the project fully understand the information of the investigational product and other responsibilities and obligationsrelated to the study specified in the project.
- Ensure that any modificantions of the trial protocol will not be conducted without the without the review and written approval of the sponsor and the Independent Ethics Committee (IEC), unless the immediate hazard for the subjects needs to be eliminated or follow the requirements of the registration regulatory authorities (e.g., administrative management aspect of the project).
- I am completely familiar with the method to correctly use the vaccine described in the trial protocol, have fully understood other information provided by the sponsor, including but not limited to the following contents: current investigator'sbrochure (IB) or equivalent documents and supplements of the IB (if any).
- I am familiar with and will abide by *Good Clinical Practice* (GCP), *Guidances on Vaccine Clinical Trial Quality Management (Trial)* and all current regulations.

Name of the Princeple Investigator: Yuliang ZHAO
Signature:
Date:

Study Team

Sponsor

Sinovac Life Sciences Co., Ltd.

Add.: No. 21, TIANFU Street, Daxing Biomedicine Industrial Base, Zhongguancun Science Park, Daxing District, Beijing, P. R ChinaName of the Contact Person: Qiang GAO

Tel: 13693092396 Fax: 010-62979669 Zip Code: 100085

E-mail：gaoq@sinovac.com

Study Institution

Hebei Provincial Center for Disease Control and Prevention

Add.: No. 97, Huaiandong Road, Shijiazhuang, Hebei Province

Department: Vaccine Clinical Evaluation Institute

Name of the Principle Investigator (PI): Yuliang ZHAO

Tel.: 13315290538 Fax: 0311-86573212 Zip Code: 050021

Email：yuliang_zh@163.com

Clinical Trial Site

Institution: Renqiiu Center for Disease Control and Prevention

Add.: North Side of Taishan Road, Renqiu

Contact Person: Hui JIN

Tel.: 13930778699 Fax: 0317-2267709 Zip Code: 062550

E-mail：1206618652@qq.com

Person in Charge of Monitoring

Name: Yuansheng HU

Sinovac Biotech Co., Ltd.

Add.: No. 39, Shangdi Xi Road, Haidian District, Beijing

Tel.: 13436950182,010-82799318 Fax: 010-82890408 Zip Code: 100085

E-mail：huys@sinovac.com

Institution Responsible for Serum Antibody Testing

Institution: National Institute for Food and Drug Control

Add.: No.31, Huatuo Road, Daxing District, Beijing

Tel.: 010-53851770 Zip Code: 102629

Data Management Institution

Institution: Meta Clinical Technology Co., Ltd.

Add.: Room A, 20 / F, Oriental International Technology building, 58 Xiangcheng Road, Pudong New Area, Shanghai

Contact Person: Hualong SUN

Tel.: 13816706496 Zip Code: 200031 E-mail：hualong.sun@meta-clinical .com

Statistical Analysis Institution

Institition: Beijing Key Tech Statistics Technology Co., Ltd.

Add.: 1018-1119w, Sihui building, Huihe Nan Street, Chaoyang District, Beijing

Contact Person: Zhiwei JIANG

Tel.: 18618483152 Zip Code: 100025 E-mail：zhi.wei.jiang@ktstat.com

Revision Record

No.	Original Version/Date/Revised content	Current Version/Date/Revised content
1	**When Version 1.4 is approved, Version 1.3 is invalid.**	
2	Version 1.3/May 11, 2020/4.1.3 Repeated Dose Toxicity Test in Rats	Version 1.4/February08, 2021/ Updated Repeated Dose Toxicity Test in Rats
3	Version 1.3/May 11, 2021/4.1.5 Reproductive and Development Toxicity Study in Rats	Version 1.4/February 08, 2021/ Updated Reproductive and Development Toxicity Study in Rats
4	Version 1.3/May 11, 2020/4.4 Study of Virus Challenge	Version 1.4/February 08, 2021/ Updated Study of Virus Challenge
5	Version 1.3/May 11, 2020/	Version 1.4/February 08, 2021/ Added "5 Preliminary Clinical Study"
6	Version 1.3/May 11, 2020/ Stability of Vaccine 6.2	Version 1.4/February 08, 2021/ Supplemented the results of stability study
7	Version 1.3/May 11, 2020/6.5 Administration Rout and Schedule	Version 1.4/February 08, 2021/ The immunization program was modified from " at the two doses schedule of day 0,28" to " Two doses of primary immunization were given at 0,28 days, respectively. One dose of booster immunization was given 6 months or 1 year after the second dose (1 year for the phase Ⅰ, 6 months for the phase Ⅱ)".
8	Version 1.3/May 11, 2020/8.2 Study Endpoint	1.4/February 08, 2021/ Added the study endpoint of booster immunization
9	Version 1.3/May 11, 2020/8.3 Study Plan &10.1 Visit Plan	1.4/February 08, 2021/ Added "one dose of booster immunization was given one year after the second dose of immunization in phase Ⅰ", and "one dose of booster immunization was given six months after the second dose of immunization was added in phase Ⅱ".At the same time, corresponding visits and sample collection were added to the visit plan.
10	Version 1.3/May 11, 2020/10.7.2 Defination of Adverse events/Reactions	1.4/February 08, 2021/The definition of adverse events/Reactions were revised according to the Chinese GCP (2020).
11	Version 1.3/May 11, 2020/10.7.6 Reporting of Serious Adverse Events	1.4/February 08, 2021/The reporting process for serious adverse events added "The report object can be adjusted according to existing regulations and the requirements of local regulatory authorities and ethics committees"; 24-hour contact of sponsor has changed.
12	Version 1.3/May 11, 2020/10.11.1 Analysis Set	1.4/February 08, 2021/ According to the revision of this vaccination procedure, the partition of the data analysis set was revised.
13	Version 1.3/May 11, 2020/11.7 Preservation of clinical trial documents	1.4/February 08, 2021/According to the requirements of Chinese GCP (2020), the content fo clinical documents preservation was revised as "The clinical trial documents must be kept according to the requirements of Chineses GCP. The sponsor, study institution and studysite should keep the clinical trial data for at least 5 years after the drug is marketed.
14	**When Version 1.3 is approved, Version 1.2 is invalid.**	
15	Version 1.2/May 4, 2020/5.6 Information of Investigation vaccine	Version 1.3/May 11, 2020/ The batch numbers of medium and high dose trial vaccines are changed, and the corresponding validity period are revised
16	Version 1.2/May 4, 2020/9.1 Visit plan	Version 1.3/May 11, 2020/ The time of Visit 2 from "the 7th day after the first dose" to "the 8th day after the first dose; the time of Visit 4 from "the 7th day after the first dose" to "the

No.	Original Version/Date/Revised content	Current Version/Date/Revised content
		8th day after the second dose
17	**When Version 1.2 is approved, Version 1.1 is invalid.**	
18	Version 1.1/April 29, 2020/1 Introduction	Version 1.2/May 4, 2020/ Updated the results of clinical trials in adults aged 18-58 years
19	Version 1.1/April 29, 2020/7.7 Protocol Violation/Deviation	Version 1.2/May 4, 2020/ Added "protocol deviation / violation report submitted to Ethics Committee for review and approval
20	Version 1.0/April 4, 2020/7.7 Protocol Violation/Deviation:<br>Conditions of protocal violation are listed as follows (including but not limited to):<br>- Subjects do not meet the inclusion criteria or meet exclusion criteria;<br>- Subjects are vaccinated with wrong vaccine;<br>- SAE is not reported within the specified time。<br>Conditions of protocal deviation are listed as follows (including but not limited to):<br>- Not receiving the investigational vaccine within the protocol-required time window;<br>- Not receiving the blood sampling within the protocol-required time window;<br>- The interval time with other vaccines can not meet the requirement of protocal (Except for rabies or tetanus vaccination in case of emergency).	Version 1.1/April 29, 2020/ Revised as:<br>Refers to any change and non-compliance with the clinical trial protocol design or process. The behavior that does not affect the rights and interests, safety and benefits of the subjects, or the integrity, accuracy and reliability of the data as well as the safety or primary indications belong to the protocol deviation; those that affect the rights and interests, safety and benefits of the subjects, or the integrity, accuracy and reliability of the data and the safety or primary indications belong to serious protocol deviation (i.e. protocol violate).<br>For the protocal deviation / violation during the study, the on-site investigators shall report the fact, process, causes and impact of the incident to the research responsible institution. The principle investigator shall give opinions on the handling of the incident.<br>The investigators should carry out targeted training for relevant staff in the related links of the violation of the protocol to prevent the recurrence of similar incidents, and record the training process.
21	Version 1.0/April 4, 2020/8.2 Exclusion criterion	Version 1.1/April 29, 2020/ Delete the BMI exclusion criteria; Add "History of SARS".
22	Version 1.0/April 4, 2020/9.5 Safety Follow-up Observation	Version 1.1/April 29, 2020/ The safety follow-up observation method was revised to "Subjects will be observed for 30 minutes on site after each dose of vaccination. Diary cards and contact cards are distributed to subjects to record the adverse events within 0~7 days and 8~28 days respectively. The investigators explain the judgment, measurement, recording, precautions and reporting method of adverse events. Systematic observation is carried out within 7 days after vaccination. Subjects are required to closely observe their own symptoms and vital signs and fill in the diary card every day. The investigators verify the adverse events on the 8th days after vaccination through face-to-face interviews on all subjects (those who do not face-to-face interviews are conducted by telephone), collect diary cards and distribute contact cards to record the adverse events within 8~28 days. The investigators verify the adverse events on the 28th days and collect contact cards.
23	Version 1.0/April 4, 2020/9.6 Blood sample collection	Version 1.1/April 29, 2020/ The blood volume is changed from 3ml to 2.5~3.5ml.

No.	Original Version/Date/Revised content	Current Version/Date/Revised content
24	Version 1.0/April 4, 2020/9.7.4 Correlation of Adverse events with Vaccination	Version 1.1/April 29, 2020/The correlation between adverse events and vaccination is modified, as shown in the protocol.
25	Version 1.0/April 4, 2020/2 Participanting Institutions and Responsibilities	Version 1.1/April 29, 2020/ Add the Data Monitoring Committee (DMC) and responsibilities
26	Version 1.0/April 4, 2020/	Version 1.1/April 29, 2020/ Delete the content related to the 0,14 day immunization program
27	Version 1.0/April 4, 2020/3.5 R&D vaccine	Version 1.1/April 29, 2020/ Updated the global vaccine research and development progress
28	Version 1.0/April 4, 2020/7.3.1 Study Plan of Phase I Clinical Trail	Version 1.1/April 29, 2020/<br>Add "Blood collection 28 days after the first dose";<br>The sample size is changed from 120 to 72.
29	Version 1.0/April 4, 2020/7.3.2 Study Plan of Phase II Clinical Trail	Version 1.1/April 29, 2020/<br>The sample size is changed from 600 to 350；<br>Add "the low dosage group".
30	Version 1.0/April 4, 2020/9.7.5 Treatment of Adverse Events	Version 1.1/April 29, 2020/ During the obervation of the study, subjects with a fever and cough and other respiratory symptoms ，as needed to fixed-point hospital, pharyngeal swab collected/sputum and anal swab, and CT and other imaging examination for analysis to determine whether will be infected by the COVID-19, in the event will be infection, the process for problems according to the SAE, especially analysis the existence of ADE phenomenon.

Protocol Summary

PROTOCOL TITLE	A Randomized, Double-blind, Placebo-controlled Clinical Trial, to Evaluate Safety and Immunogenicity of Inactivated SARS-CoV-2 Vaccine (Vero cell), in Healthy Elderly Aged 60 Years and above
SPONSOR	Sinovac Life Sciences Co., Ltd.
PROJECT PHASE	Phase I/II
OBJECTIVE(S)	To evaluate the safety and immunogenicity of SARS-CoV-2 vaccine in elderly
EXPERIMENTAL DESIGN OF THE TRIAL	A randomized, Double-blinded, Placebo-Controlled, Phase I/II Clinical Trial
PLANNED SAMPLE SIZE	Total of 422 subjects， with 72 in the phase I and 350 in the phase II clinical trial
SUBJECT SELECTION CRITERIA	Healthy older adults aged ≥60 years, with equal percentage of each gender
NAME AND FORMULATION OF DRUG	SARS-CoV-2 Inactivated Vaccine<br>-Inactivated SARS-CoV-2:<br>low dosage: 300SU/0.5ml; medium dosage: 600SU/0.5ml; high dosage: 1200SU/0/5ml<br>-Aluminum hydroxide, disodium hydrogen phosphate, sodium dihydrogen phosphate, sodium chloride, etc.
DOSAGE AND SCHEDULE	Dosage: 0.5ml/dose<br>Primary Immunization: two doses (Day 0,28)<br>Booster immunization: a booster dose 6 months or 1 year after the second dose.
ROUTE OF ADMINISTRATION	Intramuscularly, deltoid region
CHALLENGE SCHEDULE, if applicable	None
BLOOD SAMPLE COLLECTION	Phase I: day 0,28, 56,208, 388, 416<br>Phase II: day 0,56,208, 215 (or 222), 236, 388, 568
PARAMETERS OF SAFETY	Primary Endpoint<br>− Incidence of adverse reactions within 28 days after each dose of vaccination;<br>Secondary Endpoints<br>− Incidence of adverse reactions within 7 days after each dose vaccination;<br>− Incidence rate of SAEs from the beginning of the vaccination to 6 months after the booster immunization (Phase Ⅰ);<br>− Incidence of SAEs from the beginning of the vaccination to 12 months after booster immunization(Phase Ⅱ).
PARAMETERS OF IMMUNOGENICITY	Primary Endpoint<br>− The seroconversion rate of neutralizing antibodies 28 days after the second dose vaccination.<br>Secondary Endpoints<br>− The seropositive rate, GMT, and GMI of neutralizing antibodies 28 daysafter the second dose vaccination;<br>− The seroconversion rate, seropositive rate, GMT, and GMI 28 days after the first dose vaccination (Phase I).<br>Exploratory Endpoints<br>− The seropositive rate and GMT 6 months after the second dose vaccination<br>− The seropositive rate and GMT 12 months after the second dose vaccination (Phase I);<br>− The seropositive rate, GMT, and GMI 28 days after the booster vaccination (Phase I).<br>− The seropositive rate, GMT, and GMI 7 days (or 14 days) and 28 days after the booster vaccination (Phase Ⅱ).<br>− The seropositive rate and GMT 6 months after the booster vaccination.<br>− The seropositive rate and GMT 12 months after the booster vaccination (Phase Ⅱ).

Glossary

ADE	Antibody Dependent Enhancement
AE	Adverse Event
ALB	Albumin
ALT	Alanine Aminotransferase
AST	Aspartate Aminotransferase
CDC	Center for Disease Control and Prevention
CDE	Center for Drug Evaluation
CFDA	China Food and Drug Administration
CK	Creatine Kinase
COVID-19	Corona Virus Disease 2019
CPK	Creatine Phosphokinase
NMPA	National Medical Products Administration
CFDI	Center for Food and Drug Inspection
CRF	Case Report Form
eCRF	Electronic Case Report Form
ELISA	Enzyme-Linked Immune-sorbent Assay
ELISPOT	Enzyme-Linked Immuno-spot Assay
EDC	Electronic Data Capture
FAS	Full Analysis Set
GCP	Good Clinical Practice
IEC	Independent Ethics Committee
ITT	Intention-to-Treat
LDH	Lactate Dehydrogenase
MedDRA	Medical Dictionary for Regulatory Activities
PI	Principal Investigator
PPS	Per Protocol Set
PT	Preferred Term
SAE	Serious Adverse Event
SARS	Severe Acute Respiratory Syndrome
SARS-CoV-2	Severe Acute Respiratory Syndrome Coronavirus 2
SOC	System Organ Class
SOP	Standard Operation Procedure
SS	Safety Set
SUSAR	Suspected Unexpected Serious Adverse Reaction
TP	Total Protein

Abstract

SARS-CoV-2 Vaccine (Vero Cell), Inactivated developed by Sinovac Life Sciences Co., Ltd. (hereinafter referred as to "Sinovac (R&D)") can induce active immunity and prevent diseases caused by the SARS-CoV-2. According to the preliminary immunogenicity studies, the vaccine can produce good neutralizing antibody responses, and has a good effecitiveness in anmals. At the same time, comprehensive safety evaluations were carried out on animals, showing that the new vaccine is safe. This protocol is drafted on the basis of *Regulation of Drug Registration*[1], *Good Clinical Practice (GCP)* [2], *Guidance on Vaccine Clinical Trial* [3], *Guidance on Vaccine Clinical Trial Quality Management (Trial)*[4] and *Guidance on SARS-CoV-2 Vaccine (Trail)*, etc.

A randomized, double-blind, placebo-controlled design is adopted in Phase I and phase II. In the Phase I clinical trial, 72 healthy older adults aged ≥60 will be selected as study subjects. After the informed consents are signed, subjects who pass the physical examination, meet the inclusion criteria and didn't meet the exclusion criteria will be enrolled into the study. Each enrolled subject will receive two doses of primary immunization according to the immunization schedule of day 0, 28. The dose-escalating manner is used in Phase I, with 36 at medium dosage stage, and 36 at high dosage stage. The sujects enrolled at each dosage stage will be assigned in a 2:1 ratio to receive investigational vaccine or placebo respectively. The medium stage vaccination should be carried out firstly. The high dosage stage vaccination will start only with the condition that safety observation 0~7 days after the first dose of the medium dosage stage vaccination is finished, and the good safety profiles is confirmed. All enrolled subjects will receive one booster dose 1 year after primary immunization.

The phase II will be initiated only with the condition that the safety observation 0~7 days after the first dose of the high dosage stage vaccination in Phase I is finished, and the good safety profiles is confirmed the Data Monitoring Committee (DMC). A total of 350 healthy older adults aged ≥60 will be selected in the phase II clinical trial. On the premise that informed consents are signed. Subjects who pass the physical examination, meet the inclusion criteria and didn't meet the exclusion criteria will be enrolled. Each enrolled subject will receive two doses of primary immunization according to the immunization schedule of day 0,28. The subjects will be assigned in a ratio of 2:2:2:1 to receive the low dosage, medium dosage, high dosage vaccine, or placebo. All enrolled subjects will receive one booster dose 6 months after primary immunization.

The immediate reactions occur 30 minutes after each dose of vaccination will be observed on site. The local and systemic solicited AEs occurred 0~7 days after each dose vaccination, as well as the unsolicited AEs occurred 0~28 days will be collected. Additionally, the SAEs during the study periodwill be collected to evaluate the safety. Venous blood will be collected from all subjects at different time points before and after vaccination for the neutralizing antibody test, to evaluate primary immune effect, booster immune effect and immune persistence.

The clinical protocol will be independently undertaken by the investigator after being approved by independent ethics committee (IEC). The clinical research associates designated by the sponsor will monitor the whole process of the study to ensure the safety of the trial.

Contents

1 Introduction 11
2 Participanting Institutions and Responsibilities 11
2.1 Sponsor 11
2.2 Institution in charge of clinical trial 12
2.3 Study Site Institution 13
2.4 Sample Testing Institute 13
2.5 Monotroing Institution 14
2.6 Data Management 14
2.7 Statistics Analysis 15
2.8 Data Monitoring Committee 15
3 Background and Principle 15
3.1 Summary 15
3.2 Virology 16
3.3 Clinical Manifestations 16
3.4 Epidemiologic Feature 16
3.5 R&D of Vaccines 17
4 Preclinical Study and Laboratory Evaluation of Vaccines 17
4.1 Safety Study 17
4.2 Immunogenicity Study 21
4.3 Study of Virus Challenge 22
4.4 Study of Cross Neutralization 25
5 Preliminary Clinical Study 26
5.1 Phase I/ II clinical trial in adults aged 18-59 years 26
5.2 Phase I/ II clinical trial in elderly aged 60 years and older 29
6 Product Features 30
6.1 Preparation Process and Formula of Vaccine 30
6.2 Stability 30
6.3 Control Vaccine 30
6.4 Storage and Transportation 30
6.5 Administration Rout and Schedule 30
6.6 Information of Investigational Vaccine 31
6.7 Package 31
7 Objective 31
7.1 Phase I Clinical Trial 31
7.2 Phase II Clinical trial 32
8 Design 32
8.2 Study Endpoint 32
8.3 Study Plan 33
8.4 Randomization and Blinding 34
8.5 Study Time 38
8.6 Trial Suspension and Early Termination 38
8.7 Protocal Violation and Deviation 38

9 Study Population 38
9.1 Inclusion Criteria 38
9.2 Exclusion Criteria 38
9.3 Exclusion Criteria for the Second Dose and the Tird Dose 39
9.4 Subject Withdraw and Suspending Criteria 39
10 Method and Procedure 40
10.1 Visit Plan 40
10.2 Recruitment and Informed Consent 42
10.3 Screening and Random Enrollment 42
10.4 Vaccination 42
10.5 Safety Follow-up Observation 43
10.6 Blood Sample Collection 43
10.7 Safety Assessment 43
10.8 Concomitant Drugs and Vaccines 49
10.9 Immunogenicity Evaluation 49
10.10 Data Management 49
10.11 Statistical Analysis 50
11 Monitoring of Clinical Trials 56
11.1 Responsibilities of the Sponsor 56
11.2 Responsibilities of the Investigator 56
11.3 Personnel Training 56
11.4 Subject Compliance Guarantee 56
11.5 Vaccine Management in Clinical Trials 56
11.6 Management of Clinical Trial Sample 57
11.7 Preservation of Clinical Trial Documents 57
11.8 Ending Criteria for Clinical Trials 57
12 Ethical Approval 57
12.1 Review and Approval 57
12.2 Implementation of On-site Supervision 57
12.3 Confidentiality 58
13 Revision of Clinical Trial Protocol 58
14 The Publicity and Publication of Data 58
15 References 59

1 Introduction

Inactivated SARS-CoV-2 Vaccine (Vero cell), developed by Sinovac Life Sciences Co., Ltd. (hereinafter referred to as "Sinovac (R&D)"). can induce the body to produce active immunity and prevent the diseases caused by SARS-CoV-2. Preliminary immunogenicity studies showed the SAR-Cov-2 vaccine can induce good neutralizing antibody responses, and have a good effectiveness in animals. Comprehensive safety evaluations showed the SARS-CoV-2 vaccine was safety in animals.

The Phase I/II clinical trial in healthy adults aged 18~59 years was conducted in April, 2020 in Suining County, Jiangsu Province. A total of 144 subjects in Phase I and 600 subjects in Phase II have been enrolled. In Phase I of medium dosage stage, 14 subjects had reported 16 local and systemic adverse events (AEs) during 0~7 days after the first vaccination. The incidence rate of AEs was 19.44% (14/72), and pain at injection site was the most frequently reported symptoms. The most AEs were grade 1 by severity, and there were only 1 case of grade 2 AE. No grade 3 or above AEs occurred. On the 3rd day after the first dose in medium dosage stage, a total of 4 subjects had abnormal and clinically significant laboratory indicators, and the incidence rate was 5.6% (2/72). In Phase I of high dosage stage, 12 subjects had reported 21 local and systemic AEs during 0~7 days after the first vaccination. The incidence rate of AEs rate was 16.90% (12/71). The most AEs were grade 1 by severity, and there were only 1 case of grade 3 AE. On the 3rd day after the first dose in the high dosage stage, a total of 2 subjects had abnormal and clinically significant results laboratory indicators, and the incidence rate was 2.8% (2/72). Based on the above results, the DMC confirmed that the inactivated SARS-CoV-2 vaccine (vero cell) was safe and meets the requirements for Phase II. Phase II in healthy adults aged 18~59 years is currently in progress. In order to evaluate the safety and immunogenicity of the inactivated SARS-CoV-2 vaccine (vero cell) in older adults aged 60 years and above, this clinical trial protocol is designed.

2 Participanting Institutions and Responsibilities

2.1 Sponsor

2.1.1 Responsibilities

The sponsor is Sinovac (R&D), Ltd..Its responsibilities are:
-Provide the preliminary clinical trial plan, sign and seal to approve the final plan.
-Provide clinical study approval documents, investigator brochure (pre clinical safety information of products), product implementation standards and other field application documents.
- Provide trial vaccine, and issue vaccine qualified report.
- Evaluate and select the institution in charge of the clinical trial and the study site, appoint the CRA to assess the study site and preform the monitor responsibilities according the requirements of GCP; take ultimate responsibility for the quality of the clinical trial.
-Participate in the investigation and treatment of AEs , and provide the medical treatment and related compensation costs for the confirmed vaccine-related AEs according to relevant regulations; disposal of other conditions is specified in the work agreement.
-Provide clinical study funds.

2.1.2 Profile

Sinovac (R&D), formerly known as the R & D center of Sinovac Biotech Co., Ltd., is a biological high-tech enterprise wholly owned by Sinovac (Hong Kong) Co., Ltd. and registered in 2009 with a registered capital of 9.6 million US dollars. The company is Zhongguancun high-tech enterprise and Zhongguancun gold seed enterprise.

Sinovac (R&D) specializes in the research, development and technical services of human vaccines and related products, providing technical support for the prevention and control of major infectious diseases. Relying on the advantages of the group in vaccine R & D and industrialization for many years, the company has gradually formed a R & D mode with the enterprise as the main body and the combination of production, learning and research. It has

constructed a virus separation and identification technology platform, a cell factory platform, a micro carrier fermentation technology platform, a virus pure chemical technology platform, a bacterial fermentation and purification platform, a polysaccharide protein combination technology platform, and a freeze-drying technology platform, animal evaluation platform, quality control platform, diagnostic reagent raw material development platform, each platform's professional and technical advantages complement each other, cross penetrate, and promote the company's research and development to move forward steadily and efficiently.
Sinovac (R&D) has undertaken 2 national major new drug development projects and 1 Beijing Science and technology plan, and has obtained 12 Chinese invention patents. The 23 valent pneumonia polysaccharide vaccine developed by the company has successfully completed clinical studies and industrialization in Sinovac (Beijing). At present, the company is developing Poliomyelitis Hib series combined vaccine, 13 valent pneumococcal combined vaccine, Recombinant hepatitis B vaccine, etc.

2.2 Institution in charge of clinical trial

2.2.1 Responsibilities

The Institution in charge of clinical trials is Hebei Provincial Center for Disease Control and Prevention. Its responsibilities are:
-Participate in making the clinical trial protocol and required forms and cards;
-Participate in the drafting of informed consent form for vaccination, preparation of SOP for clinical trial on-site operation, and application for approval of Ethics Committee; be responsible for organizing and implementing the selection of clinical trial site that meets the requirements of GCP, organizing the evaluation of the trial site, and filing in the "Drug clinical trial organization filing management information platform" of the State Drug Administration;
-Organize the implementation of clinical trials, and control the quality of the implementation process of clinical trials;
-To be responsible for guiding the site to report the serious adverse events (SAEs) occurred during the clinical trial to the provincial drug administration, as well as the sponsor and the ethics committee in a timely manner, and to carry out investigation and disposal of SAEs;
-Participate in database locking and save the locked database backup for inspection;
-Responsible for reporting the progress of clinical trial implementation to relevant administrative departments, and writing the clinical trial summary report.

2.2.2 Profile

Hebei Provincial Center for Disease Control and Prevention (CDC) is a public welfare institution directly under the health and Health Commission of Hebei Province, which was jointly established by the former provincial health and epidemic prevention station, Provincial Institute of endemic disease control, Provincial Institute of occupational disease control, Provincial Institute of radiological health, Provincial Academy of Medical Sciences and Provincial tuberculosis control center in August, 2001. Hebei COC is the provincial technical guidance center for disease prevention, with adding four function fuctions inculding Hebei health testing center, Hebei Academy of Medical Sciences, Hebei Institute of occupational disease prevention and control, and National food safety risk monitoring of Hebei center.
The Hebei CDC currently has 40 institutes (Departments and Offices), including 14 administrative and management offices and 26 departments. The authorized staff member is 558, and there are 472 employees at present, including 144 with master's degree or above, accounting for 30.5% of the total number of employees, 159 with senior titles, accounting for 44.28% of the total number of employees, 22 provincial management experts, provincial young and middle-aged experts, and personnel with special government subsidies, and 4 provincial model workers.
The center has two provincial key medical disciplines, including cardiovascular and cerebrovascular disease prevention and control discipline and food safety risk monitoring laboratory. It is the teaching base of preventive medicine in four universities, including Hebei Medical University, Hebei University, North China University of Science and Technology, and Shanxi Medical University, which undertakes the teaching work of about 30-50 students every year. The Hebei CDC has undertaken more than 250 scientific research projects, including 11 national

science and technology major projects, 30 national cooperation projects and 2 international cooperation projects. It has won more than 110 scientific research awards at department level and above, and 25 of them have won provincial and ministerial level science and technology progress awards.

Since 2008, Hebei CDC began to carry out vaccine clinical trials according to GCP standards. Up to now and it has undertaken 28 clinical trial projects (19 have been completed), and established a relatively complete vaccine clinical trial management and quality control system. Since the implementation of *The Guiding Principles for Quality Management of Vaccine Clinical Trials (Trial)*, the center has adjusted in accordance with relevant requirements. On November 15, 2013, the vaccine clinical trial institution was established, and on November 25, 2013, a specialized management department of vaccine clinical trials, the Vaccine Clinical Research Institute, was established to be specifically responsible for the organization, management and implementation of vaccine clinical trials. There are 8 professionals in Vaccine Clinical Research Institute , including 2 chief doctors and 7 master's degrees. All personnel have received GCP training organized by NMPA system.

2.3 Study Site Institution

2.3.1 Responsibilities

The clinical study site is Renqiu City and the study site institute is Renqiu CDC. Its responsibilities are:
-Cooperate in the evaluation and filing of the test site;
-Organize the personnel with corresponding professional technology and clinical research experience to participate in the work of the research site, and all participants should thoroughly read and understand the contents of the study protocol, and strictly follow the protocol to ensure that there is sufficient time to complete the clinical study within the protocol-specified period;
-Organize on-site implementation, including the organization and selection of the subjects, obtaining the informed consent signed by the subjects, screening and enrollment, vaccination, safety visit, blood sample collection, serum separation, sample freezing and submission, etc;
-Be responsible for data input to ensure that all the collected data are true, accurate, complete and legal;
-Accept the supervision and audit of the CRA or inspector designated by the sponsor and the inspection of drug regulatory anthorities to ensure the quality of clinical research;
-Ensure that the subjects get proper treatment in case of adverse reactions / events during the study period. In case of serious adverse reactions/events, handle and report immediately according to the relevant operation procedures.
-Be responsible for the storage of relevant clinical trial data during the clinical trial.

2.3.2 Profile

Renqiu CDC is located in the North Road of Taishan, adjacent to Zhonghua Road in Renqiu city, covering an area of 10,000 m^2 with a building area of 3800 m^2. Renqiu CDC has 10 departments, 74 staff, 3 deputy chief doctors, 17 with intermediate technical titles, 27 with primary technical titles, 17 doctors, 6 assistant doctors and 9 nurses. It possessed provincial screening laboratory for HIV / AIDS confirmation, P2 laboratory and precise instruments and equipment including biosafety cabinet, autoclave sterilizer, constant temperature incubator and benchtop.

2.4 Sample Testing Institute

2.4.1 Responsibilities

National Institute of Food and Drug Control (NIFDC), its repsonsibities are:
-Be responsible for the detection of serum neutralizing antibody.

2.4.2 Profile

NIFDC is an institution directly under the National Medical Products Administration. It is the legal institution and the highest technical arbitration institution for the quality inspect of pharmaceutical biological products. It is also the designated "World Health Organization (WHO) drug quality assurance cooperation center". According to the regulatory requirements, the NIFDC perform the inspection for registration, import, supervision, safety evaluation, and batch release of biological products of multi-field products including drugs, biological products, medical devices, foods, health foods, cosmetics, laboratory animals, packaging materials, etc. Additionally, the NIFDC is responsible for the national research, distribution and management of the drug, medical device standard substances and the bacterial strains for production verification, and carry out relevant technical research work.

2.5 Monotroing Institution

2.5.1 Responsibilities

The Clinical Research Department of Sinovac Biotech Co., Ltd. is responsible for the supervision of clinical trials.
-Carry out clinical trial supervision according to GCP, protocol and SOP;
-Assist the sponsor to undertake the screening, training of the study institution, and hold the kick-off meeting;
-Check the trial process and progress;
-Check the signing of informed consent;
-Check the qualification of investigators and effectiveness of the implementation equipment;
-Check the transportation, storage, distribution, use, return and treatment of clinical trial vaccine;
-Check the collection, storage and transportation of biological samples;
-Check the handling of adverse events;
-Check the logicality of original records and report documents;
-Complete the monitoring work after the study completion, etc.

2.5.2 Profile

Since its establishment in 2002, Clinical Research Department of Sinovac Biotech Co., Ltd. has independently conducted the organization, implementation, monitoring, data management and statistical analysis of multiple studies such as inactivated hepatitis A vaccine, combined hepatitis A and hepatitis B vaccine, SARS vaccine, influenza A vaccine, H5N1 vaccine, EV71 vaccine, 23 valent pneumococcal vaccine, varicella vaccine, inactivated polio vaccine and quardrivalent influenza vaccine, and has rich experiences in clinical trial organization, implementation and management.

2.6 Data Management

2.6.1 Responsibilities

Meta Clinical Technology Co., Ltd. is responsible for data management of this clinical trial.
-Develop data management plan and data verification plan according to the requirements of the protocol;
-Provide EDC and other related online services;
-Carry out data management in accordance with the *technical guidelines for clinical trial data management* during the trial, and confirm that all data reports and records are correct and complete;
-Clean up the data, raise questions about the research data, and assist the investigators to verify and clarify;
-Write data management report.

2.6.2 Profile

Meta Clinical Technology Co., Ltd. is a contract research organization (CRO) which mainly undertakes data related service outsourcing business in clinical trials of domestic and foreign pharmaceutical enterprises. It was founded in September 2014. Now, there are offices in Shanghai, Beijing, Xi'an and Shenyang etc. The company has a strategic partnership with Colin Likang, which is a CRO providing comprehensive service. Meta Clinical Technology Co., Ltd.has provided data management, statistical analysis and drug pharmacovigilance services for phase I~IV and

bioequivalence clinical trial of innovative drugs and generic drugs of dozens of domestic and foreign pharmaceutical companies. It has:
-Standard operating procedure (SOP) and strict quality management system that meet the requirements of ICH-GCP, FDA 21 CFR Part 11, and other international or domestic requirements of clinical trial;
-Personnel who have experiences in clinical trial design, implementation, data management and statistical analysis in China, United States, European Union, Japan, South Korea, etc., and familiar with relevant drug management regulations and implementation rules;
-A complete education and training system.

2.7 Statistics Analysis

2.7.1 Responsibilities

Beijing Key Tech Statistics Technology Co., Ltd. is responsible for statistical analysis of clinical trials.
- Writing the section of randomization, sample size and statistical analysis section if the clinical trial protocol;
- Prepare statistical analysis plan according to clinical trial protocol;
- Impletation of randomization and blinding;
- Carry out statistical analysis according to the proposed statistical analysis plan and write statistical analysis report.

2.7.2 Profile

Beijing Key Tech Statistics Technology Co., Ltd. (hereinafter referred to as "Key Tech") was registered and established in Beijing in August 2017. It is a domestic funded company specializing in clinical trial data management and statistical analysis services. It takes the biostatistics service of clinical research as the core, mainly for the registration of clinical trials, and provides the statistical strategy consultation, statistical design and statistical analysis throughout the whole clinical trial process. At present, Key Tech has established offices in Beijing, Xi'an and Nanning, with 43 employees, mainly graduated from the Fourth Military Medical University, Peking University, Sichuan University and other domestic first-class universities. Among them, at present, there are 21 statisticians / statistical programmers, 18 data managers, 1 quality control personnel and 3 other non business personnel in the on-the-job employees; according to the education background distribution, there are 3 doctors, 6 masters and 34 undergraduates.

Since its establishment, Key Tech has assisted the applicants to obtain 8 clinical trial approvals, completed 18 new drug applications, including 5 new biological products of class I, and 5 products already approved for marketing, including the first 13 valent pneumonia vaccine, first nasal spray influenza vaccine, the second adamutumab product, the third quadrivalen influenza vaccine and varicella vaccine in China.In 2019, Key Tech signed an agreement with Abbott on statistical consulting services in the Asia Pacific region, and established a long-term partnership with domestic and foreign major innovative pharmaceutical enterprises.

2.8 Data Monitoring Committee

The data monitoring committee is composed of experts in clinical medicine, epidemiology and statistics. Its main responsibilities are:
- Be responsible for reviewing safety data and conducting risk assessment of clinical trials to ensure the safety of the trials.

3 Background and Principle

3.1 Summary

Since December 8, 2019, Hubei Province has reported several cases of unexplained pneumonia, most of whom work or live in the South China seafood market where live animal sales exist. The early stage of pneumonia presents severe symptoms of acute respiratory infection, and some patients develop rapidly into acute respiratory distress syndrome (ARDS). The pneumonia was confirmed to be human to human transmission, and the epidemic escalated rapidly in

early January. There were cases in all provinces of China, Japan, Singapore, the United States and more than 20 countries. A novel coronavirus was detected in the throat swab samples of patients in January 7, 2020 by the China Center for Disease Control and Prevention (CDC). The novel coronavirus pneumonia epidemic was declared as a public health emergency in January 31, 2020 by WHO. In March 12, 2020, WHO declared the epidemic entered the international pandemic stage.

The novel coronavirus gene sequences are most closely related to the two SARS like coronavirus (bat-SL-CoVZC45 and bat-SL-CoVZXC21) [6] derived from bat. The International Committee on Taxonomy of Viruses (ICTV) announced that the official classification name of this noval coronavirus was Severe Acute Respiratory Syndrome Coronavirus 2 (SARS-CoV-2) in February 12, 2020, and the World Health Organization announced on the samen day that the official name of the disease caused by the virus was COVID-19.

3.2 Virology

Coronavirus (COV) is an important pathogen of human and vertebrate. It can infect respiratory tract, gastrointestinal tract, liver and central nervous system of human, livestock, birds, bats, mice and many other wild animals. Since the outbreak of severe acute respiratory syndrome (SARS) in 2003 and the outbreak of Middle East respiratory syndrome (MERS) in 2012, the possibility of CoVs spreading from animals to humans has been proved. CoVs belong to the coronavirinae family of Nidovirales coronavirus family, which includes four genera: α-coronavirus, β- oronavirus, γ-coronavirus and δ-coronavirus [7].

SARS-CoV-2, enveloped, with a diameter of 60-140nm, harbors a linear single-stranded positive sense RNA genome, encoding 4 structural proteins. The genetic characteristics of SARSr-COV and MERSr-COV are significantly different. At present, the homology with bat-SL-COVZC45 is more than 85%. In vitro, the virus could be detected in respiratory epithelial cells in 96 hours, while in Vero E6 and Hun-7 cell lines, it takes about 6 days.

So far, the whole genome sequences of virus are comparable, showing that there is no obvious mutation in the virus. Close monitoring on novel coronavirus also indicated no significant variation existed, from virus isolated from the environment or from early-stage patients or from recent patients [8]. However, novel coronavirus is a positive-strand RNA, hence, mutation and recombination are still possible in the future which would increase or decrease the virulent.

The understanding of the physical and chemical characteristics of coronavirus mostly comes from the literatures of SARS-CoV and MERS-COV. It is sensitive to UV and heat, and it could be inactivated under the condition ether of 56 °C for 30 minutes or 75% ethanol, chlorine containing disinfectant, etc. While chlorhexidine cannot effectively inactivate the virus [9].

3.3 Clinical Manifestations

Based on the current epidemiological survey, the latent period of the COVID-19 is from 1 to 14 days, mostly 3 to 7 days. Fever, fatigue and cough are the main manifestations. A small number of patients with nasal obstruction, runny nose, sore throat, myalgia and diarrhoea. Severe patients arise with dyspnoea and/ or hypoxemia one week after the onset of the disease, among which could rapidly progress to acute respiratory distress syndrome, septic shock, metabolic acidosis and coagulation dysfunction. What is noteworthy is that patients with severe or critical condition may be accompany with moderate to low fever, or even without fever.

Atypical symptoms, such as vomiting, diarrhoea, weakness and short breath arise in children and new-borns, moreover, patients with mild manifestation were accompany with symptoms of fever, slight fatigue, without pneumonia. Most patients have a good prognosis, while few of them are in a critical condition. Patient were elderly or with chronic diseases have poor prognosis. The clinical manifestations between pregnant women and non-pregnant were similar[9].

3.4 Epidemiologic Feature

Transmission route and Susceptible population

COVID-19 patients are the main source of infection, and asymptomatic patients may also be contagious. Respiratory secretions through droplets and intimate contact contributed to person-to-person transmission. The virus is transmitted through the droplets produced by patients' coughing, sneezing and talking. The susceptible people are generally susceptible to infection after inhalation. Aerosol transmission is possible if under a relatively closed environment for long time.

The fecal-oral route remains to be determined. Recently, novel coronavirus was detected in faces of patients diagnosed in Wuhan, Shenzhen and even the United States. It indicated that the virus could be duplicated and existed in the digestive tract, suggesting the possibility of fecal-oral route transmission[10] However, the possibility of transmission through intake food contaminated by virus is still undetermined. Others pointed out that aerosol transmission is possible through feces droplets, while further investigation still needed.

It's reported that new-borns, whose delivered by positive pregnant patients, were diagnosed as positive 30 hours after birth, which indicated possibility of maternal-neonatal transmission [11].

Epidemic situation of COVID-19 in China

As of 10:00 on April 27st, 2020 (CEST), there have been 84,341 cases and 4,643deaths in China[12]. *China-WHO Novel Coronavirus Pneumonia (COVID-19) Joint Investigation Report* [13] pointed out that of 55,924 confirmed patients, the median age is 51 years (2 days to 100 years), and the interquartile spacing is 39 to 63 years old. 77.8% of patients are aged between 30 and 69 years old. Among them, 51.1% are male, 77% are from Hubei Province, 21.6% are farmers or manual workers.

In China, person-to-person transmission of novel coronavirus pneumonia occurs mainly in families according to the cluster case investigation and some family transmission studies in several provinces. A total of 1836 reported cases from Guangdong Province and Sichuan Province, of them1,308 patients were reported in 344 clusters, 78%-85% of which occurred in family members. The research for family internal transmission is in progress, but the preliminary results in Guangdong Province estimate that the second attack rate of family members is about 3%-10%. As the epidemic continues, although familial cluster infection dominates, community cluster infection also increases within hospital[13].

Global Epidemic situation of COVID-19

As of 10:00 on April 27st, 2020 (CEST), there have been 2,878,196 cases and 85,530 deaths were reported globally[12]. The countries with high incidence are the United States (931,698confirmed cases in total), Spain (207,634 confirmed cases in total), Italy (197,675 confirmed cases in total), , Germany (155,193 confirmed cases in total), the United Kingdom (152,844 confirmed cases in total), France (123,279 confirmed cases in total) and Turkey (110,130 confirmed cases in total) etc. The outbreak has influenced 209 countrie all around the world, and has caused global COVID-19 pandemic.

3.5 R&D of Vaccines

At present, there is no approved treatment or vaccine for COVID-19 in the world. According to report of WHO, as of April 26th, 2020, a total of 82 candidate vaccines are under preclinical research stage, and 7 candidate vaccines are under clinical trials, including mRNA-1273 from Modern, INO-4800 from Inovi, mRNA vaccine from Pfizer, inactivated vaccine from Wuhan Institute of Biological Products, ChAdOx1-nCOV from University of Oxford, Ad5-nCOV from CanSino Biological Inc. and inactivated vaccine from Sinovac Research & Development Co., Ltd.

4 Preclinical Study and Laboratory Evaluation of Vaccines

4.1 Safety Study

The single dose toxicity study in rats, active systemic anaphylaxis study in guinea pigs, repeated dose toxicity study on rats, repeated dose toxicity study on cynomolgus monkey and reproductive development toxicity study in rats

were carried out for the experimental vaccine. The results are as follows:

4.1.1 Single Dose Toxicity Study on Rats

Objective: To evaluate the acute toxicity of SARS-CoV-2 Vaccine on Sprague-Dawley (SD) rats within 14 days after a single dose, so as to provide toxic data for acute poisoning.

Design: 20 quarantined SD rats with equal gender and weight, were selected and randomized into vaccine group and control group to receive intramuscularly high dosage vaccine (0.5mL/1200SU [SARS-CoV-2 Unite]/rat) or saline 0.5mL/dose. Acute toxicity was observed for 14 days after injection, then perform anatomical observation.

Results: No death or near-death rats was observed in two groups, and also no clinical abnormal reaction was observed. The body weight in each group showed a normal increasing trend, and compared with the negative control animals of the same gender in the same period, there was no statistical difference in the body weight of the animals in the sample group, and there was no significant effect of the drug on the food intake of the animals. Gross anatomical observation shows no abnormality in the main organs and tissues of animals in each group.

Conclusion: when the SD rats were injected with the vaccine in high dose intended for clinical use, no abnormal changes related to drug administration were observed, and the maximum tolerated dose (MTD) of SD rats was greater than or equal to 1200SU/dose.

4.1.2 Active Systemic Anaphylaxis Test in Guinea Pigs

Objective: Observed the rapid active systemic anaphylaxis of guinea pig by sensitization via intramuscular injection of SARS-CoV-2 vaccine (once every two days for three times) and booster at D19/D26 via intravenous injection, provide animal data for the clinical trials of the tested product.

Design: According to the weight of the animal before administration, choose 36 Hartley guinea pigs of similar weight and randomly divide them into 4 groups, the low dosage group, high dosage group, negative control and positive control group, sensitized by vaccine of 0.5mL/1200SU/dose, saline and human hemoglobin. On D1, D3 and D5, the animals were intramuscularly sensitized, on D19 and D26, booster the animals via intravenous injection. The first three animals in each group received booster vaccination via foot vein, the booster dosage is twice of the sensitization dosage. Perform clinical observation after administration, the design of the study is shown in the following table:

Table 1 Design of Active Systemic Anaphylaxis Study in Guinea Pigs

Group	Sample/ Control	Number of animals	Sensitization (i.m) D1, D3, D5		Booster (i.v) D19, D26	
			Dosage	Volume (mL/GP)	Dosage	Volume (mL/GP)
1	Negative control	9	0	0.5	0	1
2	Positive control	9	20 mg/animal	0.5	40 mg/ animal	1
3	Low dose of test sample	9	0.1dose/ animal	0.05	0.2 dose / animal	0.1
4	High dose of test sample	9	1dose/ animal	0.5	2 dose / animal	1

Results: No abnormal reaction observed in regular clinical observation, the weight of animals was weighted before grouping, before last sensitization and before administration on the day of booster, the increase of weight in each group was normal. The anaphylaxis reaction in low dosage group, high dosage group and negative control group were all negative. The positive control showed positive in anaphylaxis after booster on D19 and D26.

Conclusion: No allergic reaction was observed after the guinea pigs was injected with high dosage vaccine intended for clinical use.

4.1.3 Repeated Dose Toxicity Test in Rats

Objective: To evaluate the possible toxicity reactions of SARS-CoV-2 vaccine after repeatded intramuscular injection in SD rats for 4 weeks. The recovery of the target organs of toxicity and the toxicity reactions were determined, and the safe dose of vaccine was determined, so as to provide basic data for clinical trial and clinical application of the investigational products

Triple administration trial design: According to the animal weight measured before grouping, 150 animals with qual ified quarantine and similar body weight were selected and randomly divided into 7 groups according to the gender section, which were used in the main test group (1-4 groups, low-dose group of test sample, high-dose group of test sample, negative control group and adjuvant control group) and satellite group (5-7 groups, low-dose group of test sample, high-dose group of test sample and negative control group). There were 15 animals of each sex in the main experimental group, 15 animals of each sex in the satellite group and 5 animals of each sex in the satellite group. The low-dose group, high-dose group, negative control group and adjuvant control group were treated with 0.5mL/300SU/dose, 0.5mL/1200SU/dose of test samples, 0.5mL/dose of normal saline, 0.5mL/dose of adjuvant respectively. The safety of the drug was observed by intramuscular injection on the 1st, 8th and 15th day, and the recovery period was 2 week.

Four administration test design: 80 SD rats at 5-6 weeks were selected, half male and half female, and randomly divided into two groups according to body weight:Negative control group (CN group) and SARS-CoV-2 vaccine group (T group) with 40 animals in each group, including 30 animals in the main experimental group and 10 animals in the satellite group, were given intramuscular injection once at week 0, 1, 2 and 3 with a dose of 1200SU/0.5ml per animal per time, and the recovery period was 2 weeks.

Triple administration test results: During the experiment, there were no death or near-death rats, no clinical abnormal reaction, no abnormal change in body weight and no abnormalities in body temperature and ophthalmic examination. No abnormalities in blood coagulation indicators, blood biochemical indicators, urine test indicators, T lymphocyte subsets and cytokines related to drug administration were observed. Compared with the rats in negative control group of same gender in the same period, on Day 4, basophils elevated among female rats in the low dosage group. And 3 days post last dose, neutrophils elevated among male rats and eosinophils elevated among female rats in the low dosage group, and neutrophils elevated among male rats in the high dosage group. Combined with the mode and mechanism of the reaction, it's considered that those changes may be related to the immune responses and/or local irritation induced by the test sample. In addition, compared with the rats in negative control group of same gender in the same period, 3 days (Day 18) post last dose, lymphocytes elevated among male rats in the low dosage group, while decreased in adjuvant groups. At the end of the convalescent period (Day 29), reticulocyte decreased among female rats in adjuvant group, monocytes elevated among female rats in the low dosage group. Since all those changes were not significant and only observed in a single gender, which indicated there was no correlation with the dose, it is not considered to have toxicological significance.

Pathological examination showed that there were no regular changes of toxicological significance in organ weight and organ coefficient of toxicological significance, and no obvious abnormal changes were observed in general, Observation under microscope 3 days after administration (D18), granulomatous inflammation was observed in 17/20, 13/20, and 10/20 animals in the adjuvant control group and the low-dose and high-dose groups, respectively, ranging from mild to moderate. This change was considered to be a local reaction caused by the accumulation of aluminum preparation, which is the expected reaction caused by intramuscular injection of vaccine containing aluminum adjuvant.At the end of the 2-week recovery period (D29), granulomatous inflammation was still observed at the injection site in7/10, 6/10 and 6/10animals in the adjuvant control group and the low-dose and high-dose groups, respectively, suggesting that the local irritation reaction of administration had not been recovered

Four administration test results: During the experiment, there were no death or near-death rats, no clinical abnormal reaction, no abnormal change in body weight and no abnormalities in body temperature and ophthalmic examination. At the end of the last administration and the end of the recovery period, no abnormalities in blood coagulation indicators, blood biochemical indicators, urine test indicators, T lymphocyte subsets and cytokines (IL-2, IL-10 and TNF-α) related to drug administration were observed.

Pathological examination showed that at the end of the last administration and the end of the recovery period, there were no regular changes of toxicological significance in organ weight and organ coefficient of toxicological significance, and no obvious abnormal changes were observed in general.During the whole experiment, there no local reactions visible to the naked eye such as hyperaemia, oedema, induration and necrosisoccurred at the injection site of the rats in the administration group. Microscopic observation showed that at the end of the last administration, there were 12 cases (12/20) with Infiltration of inflammatory cells in the muscle stroma at the injection site, one of which was accompanied by fibroblast proliferation. At the end of the recovery period.

Conclusion: The inactivated SARS-COV-2 vaccine were intramuscularly administered to rats for 3 or 4 doses with one-week interval between doses. During the administration period and at the end of the recovery period, no significant systemic toxic reactions were observed in rats with 300SU/dose or 1200SU/dose. No Observed Adverse Effect Level (NOAEL) was considered to be 1200 SU/rat. Irritation reaction which may be related to the aluminum adjuvant was observed at the injection site, two weeks after drug withdrawal, the degree of irritation was partially reversible, and no immune toxic reaction was observed.

4.1.4 Repeated Dose Toxicity Test on Cynomolgus Monkey

Objective: Evaluate the possible toxicity reaction and target organ after repeat dosing in cynomolgus monkeys for 4 weeks via intramuscular injection of SARS-CoV-2 vaccine and the recovery of the toxicity reaction for 4 weeks after vaccination, providing animal data for clinical trials.

Design: According to the weight of animal before grouping, 40 quarantined animals with similar weight were selected and randomized according to gender into 4 groups, which are low dosage, high dosage, negative control and adjuvant control groups. 10 Macaca fascicularis each, half male and half female. The animals in low dosage group, high dosage group, negative control and adjuvant control groups were administered by 0.5mL/300SU/dose vaccine, 0.5mL/1200SU/dose vaccine, 0.5mL/dose saline and 0.5mL adjuvant solution on D0, D7 and D14 intramuscularly. The safety observation is conducted until 14 days after the last administration. The indicators including: clinical observation including allergenic reaction and local irritation, etc., weight/temperature/food consumption/ophthalmic testing, clinicopathologic indicator (blood cell count, coagulation function, bloodchem, urine analysis), immunological indicators (T-lymphocyte subsets, cell factors, C-reaction protein, alexin, antibodies), pathology testing (gross anatomical observation, histopathological examination).

Results: During the experiment, there were no death or near-death rats, no clinical abnormal reaction. No abnormalities were observed in body weight, body temperature electrocardiogram, blood pressure and ophthalmic examination. No abnormalities in clinicopathologic indicator and immunological indicators were observed. Pathological examination was conducted 3 days post administration (Day 18), local granulomatous inflammation was observed in 5/6, 6/6 and 5/6 of animals in adjuvant group, low dosage group and high dosage group, respectively, with pathological changes ranging from mild to moderate. The change was considered to be local reaction induced by aluminum adjuvant, which belongs to the expected reaction induced by intramuscular injection of aluminum-containing vaccine. At the end of the 2-week recovery period (Day 29), local granulomatous inflammation was observed in 3/4, 4/4 and 4/4 of animals in adjuvant group, low dosage group and high dosage group, respectively, indicating that the local irritation reaction of administration had not yet recovered.

Conclusion: During the administration period and at the end of the two-week recovery period, no significant systemic toxic reactions were observed in Macaca fascicularis using 300 SU and 1200 SU, so the No Observed Adverse Effect Level (NOAEL) was considered to be 1200 SU/ Macaca fascicularis. Irritation reaction which may be related to the aluminum adjuvant was observed at the injection site, and no immune toxic reaction was observed.

4.1.5 Reproductive and Development Toxicity Study in Rats

Objective: To evaluate the effect of the SARS-COV-2 vaccine on the fertility of male and female rats, the

development of pregnant / lactating female rats, embryos and fetuses, to understand the effect of the vaccine on teratogenesis and offspring development of rats, and to investigate the antibody level in the blood of embryo or offspring and to provide reference for safe drug use in special populations.

Design: According to the weight of the animal before administration, the animals were randomized into 4 groups according to gender. 28 male rats and 56 female rats were randomized in low dosage group, high dosage group, negative control group and adjuvant group and administered with 0.5mL/300SU/dose vaccine, 0.5mL/1200SU/dose vaccine, 0.5mL/dose saline and 0.5mL adjuvant solution. The male rats were administered three times before mating on D1, D8, D15 and D29 while females were administered three times before mating on D1, D8 and D15. 1 week after last administration of male rates, the males and females were mated. The female rats are administered on GD6 and PND7. ON GD20, 1/2 pregnant mice in each group were caesarean for inspection of the foetus (appearance, viscera, skeleton), the other 1/2 of the pregnant rats had a normal labor and feed until the end of lactation.

Results: After repeated intramuscular injection of SARS-CoV-2 vaccine at the doses of 300SU/mouse or 1200SU/mouse from pre mating to embryo inplantation and delivery in SD rats, there was no effect on the fertility of parental female and male rats, no obvious adverse reactions in pregnant/lactating female rats, no embryo fetal development toxicity and teratogenicity, and no effect on the growth and development of F1 offspring.

4.2 Immunogenicity Study

In order to evaluate inactivated SARS-CoV-2 Vaccine (Vero cell), mice and rats were immunized intraperitoneally and intramuscularly with vaccine of different dosage, and different adsorption methods at different immunization schedules. Blood samples were collected at different time points for the testing of serum neutralizing antibody titer and IgG antibody titer after immunization, to determine the immunogenicity of the vaccine. The formulation, dosage and immune schedule of the vaccine are determined according to the immunogenicity results.

Study Design:

- **Determination of aluminum adsorption and non-aluminum adsorption processes for vaccine**

Two different processes were employed to prepare aluminium-containing SARS-CoV-2 vaccines of 1200 SU/0.5 ml, 600 SU/0.5 ml, 300 SU/0.5 ml and 150 SU/0.5 ml, and aluminium-free SARS-CoV-2 vaccines of 1200 SU/0.5 ml, 600 SU/0.5 ml and 300 SU/0.5 ml. Mice were intraperitoneally immunized by the above vaccines, 10 mice per group, 0.5 ml per mouse. For the mice immunized with one injection, serum was collected on Day 7, Day 14 and Day 21 after immunization; for the mice immunized with two injections on Day 0, 7 and Day 0, 14, serum was collected on Day 14, Day 21 and Day 28, and serum IgG antibody titer was determined separately. Negative animal control was set. Immunogenicity of vaccines prepared by two different processes was compared via a comparison of the antibody titers, the specific study design is shown in the table below:

Table 2 Study design of the comparison between immunogenicity of aluminium-adsorbed and non-aluminium adsorbed SARS-CoV-2 vaccine

Dosage (SU/0.5 ml)	Inactivated SARS-CoV-2 vaccine				Inactivated non-aluminium adsorbed SARS-CoV-2 vaccine			
	Batch No.	One dose	Two doses (D0, 7)	Two doses (D0, 14)	Batch No.	One dose	Two doses (D0, 7)	Two doses (D0, 14)
1200 SU	20200303-1	10	10	10	20200303-5	10	10	10
600 SU	20200303-2	10	10	10	20200303-6	10	10	10
300 SU	20200303-3	10	10	10	20200303-7	10	10	10
150 SU	20200303-4	10	10	10	/	/	/	/

- **Determination of Immunization Dosage and Immunization Schedule**

Mice group: Fifty mice were randomized into five groups to intraperitoneally receive three kinds of emergency schedule and two kinds of routine schedule using four antigen content vaccine of 300SU/0.5mL, 600SU/0.5mL,

1200SU/0.5mL and 2400SU/0.5mL, respectively.

Rats group: Twenty-five rats were randomized into five groups to intraperitoneally receive three kinds of emergency schedule and two kinds of routine schedule using four antigen content vaccine of 300SU/0.5mL, 600SU/0.5mL, 1200SU/0.5mL and 2400SU/0.5mL, respectively.

Details of immunization and blood sample collection are shown in Table 5.

The proposed dose was determined by the analysis of the immune dose, neutralization antibody titer and enzyme labeled antibody titer. The immune schedule was determined by comparing the immune effects of one, two or three doses.

Table 3 StudyDesign of the Immunization Dosage and Immunization Schedule of SARS-COV-2 vaccine

Immunization Schedule	Immunization Schedule	Date of blood sampling	Amount
Emergency schedule	Day 0	Day 7, 14, 21, 28, 35, 42	10 Mice, 5 Rat
	Day 0, Day7	Day 14, 21, 28, 35, 42	10 Mice,5 Rat
	Day 0, Day 3, Day 7	Day 7, 14, 21, 28, 35, 42	10 Mice,5 Rat
Routine Schedule	Day 0, Day 14	Day 21, 28, 35, 42	10 Mice,5 Rat
	Day 0, Day 14, Day 28	Day 35, 42	10 Mice,5 Rat

Study result:

- **Determination of Aluminum adsorption and non aluminum adsorption**

The vaccines containing aluminum adjuvant and the vaccine free from aluminum in mice are able to produce a certain level of novel coronavirus antibody on the 7th day after initial immunization. The vaccine of 1200SU/0.5mL free from aluminium adjuvant with was the same as that of 300SU/0.5mL with aluminum adjuvant. The immunogenicity of the vaccine containing aluminum is better than that of the vaccine without aluminum.

- **Determination of Immunization dosage and Immunzation schedule**

(1) For the same species of animals immunized by different doses via the same procedure, the neutralizing antibody titer was determined at the same blood sampling time point. The results showed that the immunization doses and produced neutralizing antibody titers showed a good dose-response relationship.

(2) For the same species of animals immunized by the same dose via different procedures (one-injection, two-injection and three-injection), the enzyme-labelled antibody titer was determined at the same blood sampling time point. The results showed that the immunization effect of two-injection and three-injection procedures were both not inferior to that of one-injection procedure in mice, and the immunization effect of two-injection and three-injection procedures were both superior to that of one-injection procedure in rats. Because of the short interval between three injections, the immunization effect of two-injection procedure was comparable to that of the three-injection procedure.

(3) For the two-injection immunization procedure of the same dose at different time points (Day 0, 7 and Day 0, 14), the enzyme-labelled antibody level of Day 0, 14 immunization procedure was an order of magnitude higher than that of Day 0, 7 immunization procedure on Day 21, indicating that the interval between two injections should be more than 14 days in clinical trials.

(4) For two-injection immunization by different doses via the same immunization procedure, the neutralizing antibody titers of 1200 SU and 2400 SU are almost the same.

Conclusion: the formulation containing aluminium adjuvant was selected, the expected doses in clinical trials were determined to be 300 SU/dose, 600 SU/dose and 1200 SU/dose, and the immunization procedure was determined to be two injections.

4.3 Study of Virus Challenge

Objective: Use the SARS-Cov-2 to attack animals that have been immunized with the inactivated SARS-COV-2 vaccine according to different immunization procedures and doses to evaluate the animal protective effect of the inactivated SARS-COV-2 vaccine and to evaluate the existence of antibody-mediated infection enhancement (ADE), so as to provide animal data for clinical research and application.

Design: The inactivated SARS-COV-2 vaccine was used to immunize rhesus monkeys according to different immunization procedures and doses, try the virus seed to attack animals 21 to 42 days after the first immunization of the inactivated SARS-COV-2 vaccine,the protective effect of the vaccine was evaluated according to clinical symptoms observation, serum antibody detection and hiopathological examination results of rhesus monkeys, and the presence of ADE under different antibody levels was observed. The study design was shown in the table below:

Table 4 Study Design of Protection Effect of Virus Challenge

Number	Group	Schedule (day)	Dose	Days after the first dose	Pleasant days (After virus challenge)	Number of animals
1	3 doses - vaccine	0,7,14	High dosage (1200SU/0.5ml)	23	7	4
			Miedum dosage (600SU/0.5ml)	22	7	4
2	3 doses - adjuvant	0,7,14	/	21	7	2
3	Model group	/	/	21	7	2
4	2 doses - vaccine	0,14	High dosage (1200SU/0.5ml)	23	7	4
			Miedum dosage (600SU/0.5ml)	22	7	4

Results of two-dose schedule:

In the model group, there was no significant increase in temperature. 2 out of 4 macaques in medium dosage group had high fever over 40°C, and there was no abnormal body temperature in the high dosage group. High viral load was detected in throat swab, anal swab and lung tissues in the model group. Compared with the model group, all of the macaques in medium dosage group was negative in throat swab virus test on Day 3, 5, 7 post virus challenge, all of the macaques were negative in lung tissue virus test on Day 7 post virus challenge, and all of 4 macaques showed mild interstitial pneumonia, suggesting that the medium dosage vaccine had significant protective effect. Compared with the model group, 3 out of 4 macaques in high dosage group was negative in throat swab virus test were negative on Day 3, 5, 7 post virus challenge, all of the macaques were negative in lung tissue virus test on Day 7 post virus challenge and all of 4 macaques showed mild interstitial pneumonia, suggesting that the high dosage vaccine had significant protective effect. The changes of antibody levels in rhesus monkeys in each group were shown in Table 5. According to the results of immune protection of medium dosage group and high dosage group and the level of neutralizing antibody before challenge, it is suggested that the neutralizing antibody titer after 2 doses is greater than or equal to 1:48 had significant protective effect based on the results of immune protection of

Table 5 Changes of antibody levels in rhesus monkeys in each group after administration with the SARS-COV-2 vaccine

	Animal No.	Day 0	Day 7	Day 14	Day 21	Day 3 post virus challenge	Day 5 post virus challenge	Day 7 post virus challenge
Medium dosage group	K21	<8	<8	4	64	64	48	256
	K22	<8	<8	4	128	48	64	128
	K23	<8	<8	6	48	32	96	1024
	K24	<8	<8	32	64	256	128	1024
GMT	/	/	/	7.4	70.8	70.8	78.4	430.5
High dosage group	K17	<8	<8	16	128	1024	512	512
	K18	<8	<8	16	256	256	512	512
	K19	<8	<8	4	96	512	1024	512
	K20	<8	<8	<4	64	192	256	1024
GMT	/	/	/	6.7	119.1	400.7	512.0	608.9

Adjuvant group	K9	<8	<8	<4	<4	<4	4	8
	K10	<8	<8	<4	<4	<4	<4	<8
GMT	/	/	/	/	/	/	/	/
Model group	K15	<8	<8	<4	<4	<4	6	12
	K16	<8	<8	<4	<4	<4	8	8
GMT	/	/	/	/	/	/	6.9	9.8

Results of three-dose schedule:

After the virus challenge, the body temperature of the animals in the model group did not increase significantly, and there was no abnormality in the body temperature of the adjuvant group, the medium-dosage group and the high-dosage group. The WBC count decreased and the percentage of lymphocytes increased after the animals in each group were infected, and there was no statistically significant difference between the medium-dosage group or high-dosage group and the model group. The blood biochemical index values of animals in each group were within the normal range on day 0 and day 14 after immunization, and when the animals were sacrificed. High levels of viral load were detected in throat swabs, anal swads and lung tissues in the model group. Compared with the model group, the average viral load of throat swabs and anal swabs decreased in the medium-dosage group 7 days after challenge. In the high-dosage group, 7 days after challenge, throat swabs and anal swabs tested negative for virus; 3 out of 4 in the middle-dosage group tested negative for virus in the lung tissue 7 days after the challenge, and 4 in the high-dosage group tested negative for virus in the lung tissue 7 days after the challenge.

The neutralizing antibody in the model group and the adjuvant group were both negative and the neutralizing antibody GMT was 61.3 in the medium-dosage group and 50.1 in the high-dosage group 21 days after immunization. 7 days after the challenge, the GMT was 400.7 in the medium-dosage group and 145 in the high-dosage group.

Table 6 Changes of antibody levels in rhesus monkeys in each group after administration with the SARS-COV-2 vaccine

	**Animal No.**	**Day 0**	**Day 7**	**Day 14**	**Day 21**	**Day 3 post virus challenge**	**Day 5 post virus challenge**	**Day 7 post virus challenge**
Medium dosage group	K5	<8	<8	6	64	32	384	1024
	K6	<8	<8	4	24	32	64	512
	K7	<8	<8	48	384	128	512	768
	K8	<8	<8	6	24	32	64	64
GMT		/	/	9.1	61.3	45.3	168.5	400.7
High dosage group	K1	<8	<8	12	48	24	96	256
	K2	<8	<8	16	64	96	512	384
	K3	<8	<8	6	32	24	48	96
	K4	<8	<8	6	64	16	48	48
GMT		/	/	9.1	50.1	30.7	103.2	145.9
Adjuvant group	K9	<8	<8	<4	<4	<4	4	8
	K10	<8	<8	<4	<4	<4	<4	<8
GMT		/	/	/	/	/	/	/
Model group	K15	<8	<8	<4	<4	<4	6	12
	K16	<8	<8	<4	<4	<4	8	8
GMT		/	/	/	/	/	6.9	9.8

The pathological results of some animals are shown in Figure 1-6.

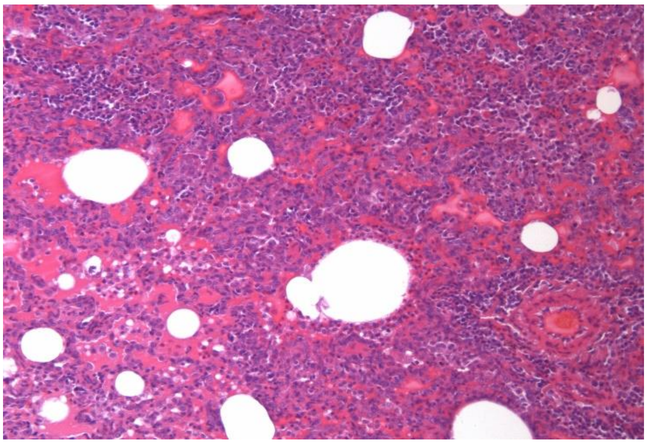

Figure 1 Model group K15 Deputy right lung lobe Severe interstitial pneumonia H.E.×100

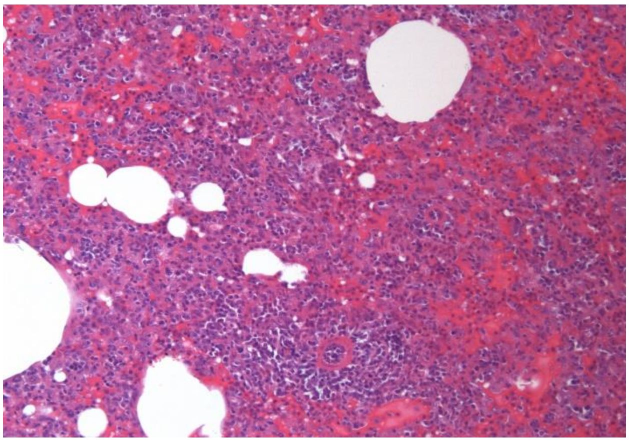

Figure 2 Adjuvant group K10 middle lobe of right lung Severe interstitial pneumonia H.E.×100

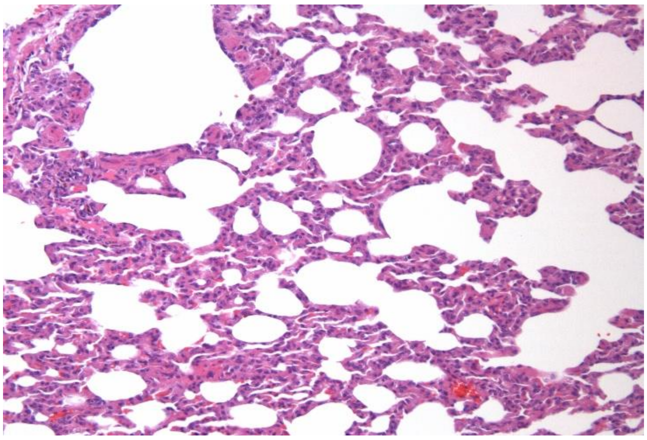

Figure 3 Miedum dosage group K21 superior lobe of right lung mild interstitial pneumoniaH.E.×100

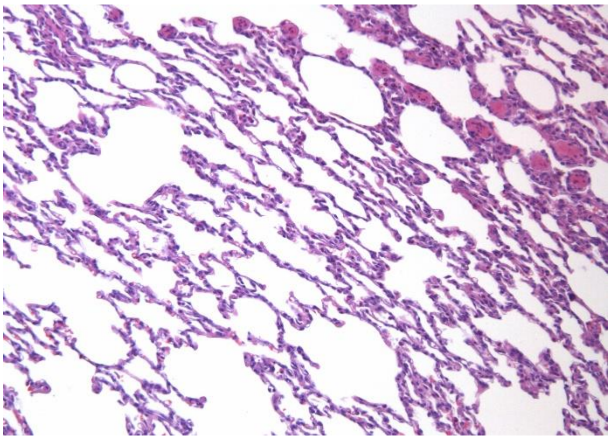

Figure 4 Miedum dosage group K2 superior lobe of left lung nothing abnormal detected H.E.×100

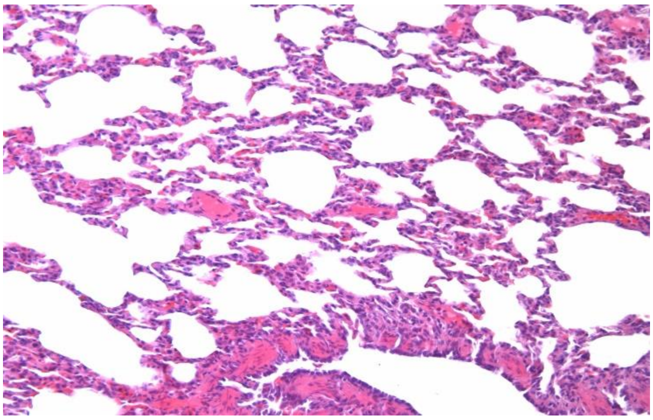

Figure 5 High dosage group K17 inferior lobe of right lung mild interstitial pneumonia H.E.×100

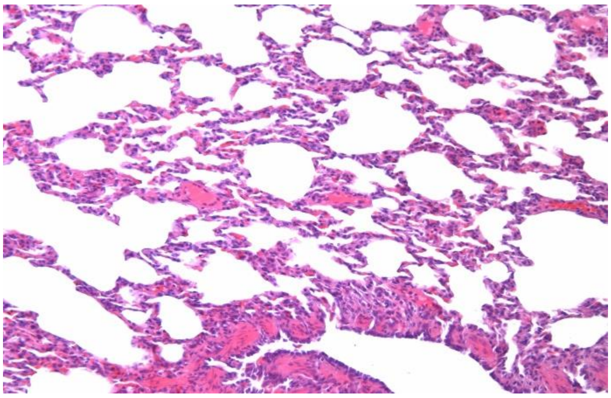

Figure 6 High dosage group K19 inferior lobe of left lung nothing abnormal detected H.E.×100

Conclusion:

The SARS-COV-2 vaccine had significant protective effect in rhesus monkeys. And ADE phenomenon was not observed.

4.4 Study of Cross Neutralization

Objective: To evaluate the cross-neutralizing effect of SARS-CoV-2 immunized serum against different virus strains.

Design: SARS-CoV-2 strain of different time and place of isolation was selected for cross-neutralizing test with SARS-CoV-2 immunized serum from different sources (including serum from convalescent patients and animal serum immunized by inactivated CZ02 strain SARS-CoV-2). The selected SARS-CoV-2 strains are shown in the table below:

Table 7 Basic information of the virus strains

Virus strain	Batch No.	Source
CW01	CW01-W-202002-01	Wuhan (China)
WXY-C3	WXY-202002-01	Zhejiang (China)
CZ01	CZ01-W-202002-01	Zhejiang (China)
CB01	CB01-P5-202002-001	Virus institute (China)
V34	V34-P4（D3）	Military academy of science (China)
CZ12	CZF-202003-01	Zhejiang (China)
CZ30	WGF-202003-01	Zhejiang (China)
HAC	HAC-202003-01	Imported from Italy
HJL	HJL-202003-01	Imported from Italy
QHF	QHF-202003-01	Imported from Spain
SSH	SSH-202003-01	Imported from Switzerland
ZYF	ZYF-202003-01	Imported from Italy

Results: The convalescent serum from patients infected with different sources of novel coronavirus could produce cross-neutralizing reaction to different domestic and foreign coronavirus strains, and the results were basically consistent. The immune serum of novel coronavirus inactivated vaccine prepared with CZ02 novel coronavirus as the candidate strain showed good cross-reaction to different domestic and foreign isolates, which was consistent with the trend of serum reaction in clinically confirmed patients infected with novel coronavirus.

Conclusion: The immune serum of novel coronavirus from different sources can produce cross-neutralizing reaction to novel coronavirus in different situations, and the results of cross-neutralizing reaction is basically consistent.

5 Preliminary Clinical Study

5.1 Phase I/ II clinical trial in adults aged 18-59 years

5.1.1 Safety Assessment

- **Adverse Reactions**

Within 28 days after two doses of the emergency immunization schedule (0,14 days) in 372 subjects of phase Ⅰ/II clinical trial of inactivated SARS-COV-2 vaccine in adults aged 18-59 years, the incidence rates of adverse reactions in the medium dosage group , high dosage group and placebo group were 27.08%,31.94% and15.48% ,respectively, and there was significant statistical difference among groups (P=0.0136), with the high dosage group> medium dosage group> placebo group. Most adverse reactions were mild (grade 1) in intensity, and only one grade 3 adverse reaction occurred in the high-dose group. All adverse reactions occurred within 7 days .The incidence of adverse reactions after the second dose was significantly lower than that after the first dose.The incidence of adverse events was 16.67%, 16.67% and 15.66% in the medium dosage, high dosage and placebo groups, respectively, within 28 days after two doses (0,28 days) of the routine immunization schedule in 371 subjects of Phase Ⅰ/II clinical trial and there was no significant statistical difference in the incidence rates among groups (P=0.9546).Adverse reactions were mainly mild, and there was no grade 3 adverse reactions. Adverse reactions occurred within 7 days after vaccination. The incidence of adverse reactions after the second dose was significantly lower than that after the first dose.

In the three-dose emergency schedule (Day 0,14 and 42) of phase Ⅱ, the incidence rates of adverse reactions were

30.00%, 31.67% and 13.30% in the medium dosage group, high dosage group and placebo group, respectively from the first vaccination to 28 days after the third dose in 150 subjects. There was no significant statistical difference in the incidence rates among groups (P=0.2520). In the three-dose routine schedule (Day 0,28 and 56) of phase II, the incidence rates of adverse reactions were 18.33%, 18.33% and 23.33% in the medium dosage group, high dosage group and placebo group, respectively from the first vaccination to 28 days after the third dose in 150 subjects. There was no significant statistical difference in the incidence rates among groups (P=0.6210). Most adverse reactions were grade 1, and there was no grade 3 adverse reactions occurred. All adverse reactions occurred within 7 days after vaccination. The incidence of adverse reactions after the third dose was significantly lower than that after the first dose and the second dose.

The main symptom of adverse reactions was injection site pain, followed by fatigue. Up to 6 months after the two doses of immunization, a total of 13 serious adverse events occurred in 9 subjects in the phase Ⅰ/II clinical trial, including 3 serious adverse events in 2 subjects in phase Ⅰ clinical trial and 10 serious adverse events in 7 cases in phase Ⅱ clinical trial, respectively, and all were not related to vaccination.

➢ **Laboratory test index**

The blood routine test, blood biochemical test and urine routine test were carried out before and 3 days after vaccination among all subjects aged 18-59 years in Phase I.

The results showed that the incidence of abnormal laboratory indexes (mainly grade 1) of clinical significance was low. In the emergency immunization schedule, the incidence of clinically significant abnormal laboratory index on the 3th day after each dose of vaccination was 8.33%, 8.33% and 4.17% in the medium dose group, high dose group and placebo group, separately. In the routine immunization schedule, they are 8.33%, 8.33% and 4.35%, respectively. There was no significant difference in the incidence rate among the groups with different immune procedures.

➢ **Inflammatory factors**

The inflammatory factors of all subjects aged 18-59 years in Phase I were detected before and after immunization. The changes of IL-6, IL-2 and TNF - α in emergency and routine immunization schedules were small, and no significant increase of serum inflammatory factors was found, indicating that the risk of immune pathological reaction induced by vaccine was low.

5.1.2 Immunogenicity Evaluation

5.1.2.1 Immunogenicity results of two-dose schedule

In phase Ⅰ of adults aged 18-59 years, the seroconversion rates of neutralizing antibody to SARS-CoV-2 were 45.83%, 50.00% and 0%, respectively,in the medium dosage group , high dosage group and placebo group at 14 days after two doses accoding to the emergency immunization schedule (Day 0,14), and the GMT (1:1) were 5.6, 7.7 and 2.0, respectively. The seroconversion rates were 83.33%, 79.17% and 4.35% in the medium dosage group, high dosage group and placebo group, respectively, at 28 days after two doses accoding to the routine immunization schedule (Day 0,28), and the GMT were 19.0, 29.6 and 2.2, respectively.

In phase Ⅱ of adults aged 18-59 years, the seroconversion rate of neutralizing antibodies were 92.37%, 98.32% and 3.33%, respectively, in the medium dosage group , high dosage group and placebo group at 14 days days after two doses accoding to the emergency immunization schedule (Day 0,14) (Subject No.: C001-C300), and the GMT were 27.6, 34.5 and 2.3, respectively. The seroconversion rate were 97.44%, 100% and 0% in the medium dosage group, high dosage group and placebo group, respectively at 28 days after two doses according to routine immunization schedule (Day 0,28) (Subjects No: D001-D300), and the GMT of neutralizing antibodies were 44.1, 65.4 and 2.0, respectively. The results showed that the inactivated SARS-COV-2 vaccine had good immunogenicity according to the emergency or routine immunization schedule. The seroconversion rate (98.32%) in high dosage group was slightly higher than that of medium dosage group (92.37%, P=0.0296) 14 days after second dose vaccination of the

emergency schedule. 28 days after the second dose vaccination of the routine schedule, the GMT of neutralizing antibody in high dosage group (65.4) was higher than that of medium dosage group (44.1, P=0.0006). The immunogenicity in medium and high dosage group were comparable given the GMT difference was less than 1.5 fold and the seroconversion rates were all higher than 90%.

The immunogenicity in the phase II was significantly better than that in phase I, showed better immune effect from improved new technology. Analysis the protein composition of purified virus particles showed the S-protein content in vaccine produced with new technology was about twice of it in vaccine produced with old technology. The reason was, compared to cell factory, the bioreactor provides a highly automatic cultivation environment, with dissolved oxygen, pH and CO_2/O_2 under strict control. Based on that, it was assumed that the cultivation environment provided by bioreactor enhanced the S-protein content and improved immunogenicity.

5.1.2.2 Immune persistence of two-dose schedule

In phase Ⅱ, the seroconversion rates of neutralizing antibodies were 16.95%, 24.14% and 0% in the medium dosage group , high dosage group and placebo group at 6 months after the second dose and the GMTs were 4.1, 4.8 and 2.0, respectively, according to two-dose immunization schedule. The seroconversion rates of neutralizing antibodies were 35.19%, 46.43% and 0% in the medium dosage group, high dosage group and placebo group at 6 months after immunization and the GMTs were 6.7, 7.1 and 2.0, respectively. The results showed that neutralizing antibodies dropped to low levels according to different immunization schedule.

5.1.2.3 Immunogenicity results of three-dose schedule

In phase II, the seroconversion rates of neutralizing antibody in the medium dosage group were 94.83%, 93.22% and 98.15% at 14 days after the second dose, 28 days after the second dose and 28 days after the third dose, respectively, and the GMTs were 27.0, 22.2 and 45.8, respectively, according to the three-dose emergency schedule (Day 0, 14, 42); the seroconversion rates in the high dosage group were 98.33%, 98.33% and 98.28%, respectively, at 14 days after the second dose, 28 days after the second dose and 28 days after the third dose and the GMTs were 40.8, 29.1 and 74.2, respectively. The seroconversion rates of neutralizing antibody on the 28th day after the third dose in the medium and high dosage groups were similar, but GMT in the high dosage group was higher than that in the medium dosage group (P=0.0052).

In phase II, the seroconversion rates of neutralizing antibody in the medium dosage group were 94.92% and 98.11% at 28 days after the second dose and 28 days after the third dose, respectively, and the GMTs were 39.6 and 49.7, respectively, according to the three-dose routine schedule (Day 0,28,56). The seroconversion rates in the high dosage group were 100% and 100% t 28 days after the second dose and 28 days after the third dose, respectively, and the GMTs were 58.4 and 51.9, respectively. The seroconversion rates and GMT at 28 days after the third dose in the medium and high dosage groups were similar.

5.1.3 Conclusion

The inactivated SARS-CoV-2 vaccine (CoronaVac) manufactured by Sinovac has good safety and immunogenicity, and can produce antibodies rapidly after two doses according to 0,14 days and 0,28 days.According to CDE's post-marketing requirements for conditional approval of the inactivated SARS-CoV-2 vaccine:"If the results of subsequent clinical trials suggest that the existing immunization schedule and dose are not optimal, further studies should be carried out to optimize the immunization schedule and dose." Given that the neutralizing antibodies at 6 months after the two doses of primary immunization have decreased to a low level,at present, one dose of booster immunization in 6 months after primary immunization has been carried out in population aged 18 to 59 years in phase Ⅱ clinical trial with different immunization schedule, so as to further explore the immune effect of booster immunization

schedule and provide a basis for the formulation of the optimal immunization strategy.At present, the results of booster immunization in 6 months after primary immunization are not available.

5.2 Phase I/ II clinical trial in elderly aged 60 years and older

5.2.1 Safety Assessment

A total of 421 subjects received at least one dose of vaccine or placebo in the phase I/II clinical trial. In the safety populations from the Phase I and Phase II, the incidence rates of adverse reactions from the beginning of the first dose to 28 days after the second dose in low dosage group, medium dosage group, high dosage group, and placebo group were 20.00%, 20.00%, 21.95% and 20.55%, respectively. There was no significant difference in the overall adverse reactions among the four groups. All adverse reactions were mild and moderate. No grade 3 adverse reactions occurred. Adverse reactions mainly occurred within 7 days after vaccination. The incidence of adverse reactions after the first and second doses of the low-dose and high-dose groups was similar, and the incidence of adverse reactions after the first dose of the medium-dose group and the placebo group were slightly higher than the second dose, there is no obvious trend of increasing or decreasing adverse reactions with the increase of doses. Pain at injection site was the most frequently reported symptoms, with the incidence rates of 11.00%, 11.20%, 8.94% and 4.11%, respectively. The headache and mucocutaneous eruption in high dosage group were slightly higher than that in other three groups. The incidence of hypoesthesia in the placebo group was slightly higher than that of the other three groups, and the differences in other symptoms were not statistically significant.

A total of 7 subjects reported 8 serious adverse events from the beginning of the first dose to 28 days after the second dose , the incidence rates of serious adverse events were 4.00%, 0.80%, 1.63%, and 0.00%, respectively, in the low-dosage, medium dosage, and high dosage and placebo groups ,and all of the serious adverse events were unrelated to vaccination.

5.2.2 Immunogenicity Evaluation

In phase I, the seroconversion rates (≥1:8) in medium dosage group, high dosage group and placebo group were 100.00%, 95.65% and 0.00% respectively 28 days after the second dose vaccination. GMTs (1:) were 54.9, 64.4 and 2.0, and GMTs were 27.5, 32.2 and 1.0, respectively. In phase II, the seroconversion rates (≥1:8) in low dosage group, medium dosage group, high dosage group and placebo group were 90.72%, 97.96%, 98.98% and 0.00% respectively 28 days after the second vaccination. GMTs (1:) were 23.4, 42.2, 49.9 and 2.1, and GMIs were 11.7, 20.9, 24.2 and 1.0, respectively. The results of the study showed that in both phase I and phase II clinical trials, the seroconversion rate reached more than 95% 28 days after the second dose immunization of the medium-dose group and the high-dose group of the experimental vaccine. The medium and high dose groups did not show a dose-response relationship and the results of phase I and phase II were similar. Phase II clinical trials increased the low dosage group. The results of the study showed that the immunogenicity results of the medium and high-dose groups were significantly better than those of the low dosage group, showing a significant dose-effect relationship between those groups.

5.2.3 Conclusion

The inactivated SARS-CoV-2 vaccine (CoronaVac) manufactured by Sinovac has good safety in people aged 60 years and older according to the 0,28 day immunization schedule. It had better immunogenicity in the elderly in the medium dosage and high dosage groups and the results were similar and significantly better than that in low dosage group. The results were similar to that of the phase II clinical trial of the inactivated SARS-CoV-2 vaccine in adults. Based on the research results in the population aged from18 to 59 and the requirements for drug registration certificate (certificate number: 2021S00156), the subjects of this study will receive one dose of booster immunization in 6 months or 1 year after primary immunization, so as to further explore the immune effect of booster immunization

schedules, to provide evidence for the optimal immune strategy.

6 Product Features

6.1 Preparation Process and Formula of Vaccine

Inactivated SARS-CoV-2 Vaccine (Vero Cell), is prepared from novel coronavirus (CZ02 Strain), which is inoculated on African green monkey kidney cells (Vero Cells), then cultured, harvested, inactivated, concentrated, purified and finally aluminium absorbed. The finished vaccine is a milky white suspension liquid, which can be layered due to precipitation and easily dispersed. The main component of the vaccine is the inactivated noval coronavirus (SARS-COV-2), with the excipients of aluminum hydroxide, disodium hydrogen phosphate, sodium dihydrogen phosphate, sodium chloride, etc., and the vaccine is preservative-free. The vaccine is packaged with prefilled syringes or vials, 0.5ml for each container. The vaccine can induce the immunity against the SARS-COV-2, which can prevent the disease caused by the SARS-COV-2 infection.

The investigational vaccine is manufactuted by Sinovac Research & Development Co., Ltd. and tested eligible by National Institute for Food and Drug Control according to *Manufacturing and Quality Control Requirements of Inactivated SARS-CoV-2 Vaccine（Vero Cell) (Draft Version)* . The vaccine is injectable with the specificantion of 0.5mL/container. The antigen content of low, medium and high dosage vaccine is 300SU, 600SU, and 1200SU/0.5mL respectively.

6.2 Stability

Six lots of vaccine products produced in the bioreactor have completed the thermal accelerated stability studies at 25±1℃ for 56 days and 37±1℃ for 42 days. When stored at 25±1℃, the antigen content at each time point including 42 days meets the quality standard after disintegration,the antigen content is lower than the quality standard after 56 days of disintegration,, and the study will be terminated.When stored at 37±1℃, the antigen content at each time point including 28 days meets the quality standard after disintegration. The antigen content of some lots is less than 50% of the labeled amount after disintegration at the monitoring point on 42 days, and the test will be terminated.
3 lots of products at 2-8℃ in the cell factory has been completed the long-term stability observation for 6 months, and the antigen content of each lots products of the inactivated SARS-CoV-2 vaccine did not significantly decrease after disintegration.Among the 9 lots products under the bioreactor process, 3 lots have completed the long-term stability observation for 6 months with no significant changes in the data of each test item have been found.The other 6 lots have completed the long-term stability observation for 3 months, and the antigen content of each lot products of the inactivated SARS-CoV-2 vaccine has not decreased significantly after disintegration.
The validity of the vaccine was tentatively set at 2-8℃ for storage for 2 years according to the results of accelerated stability.

6.3 Control Vaccine

In this study, placebo produced by Sinovac Research & Development Co., Ltd. was adpopted as the control. The placebo is aluminum hydroxide diluent with trace of milkly white precipitation. The appearance is consistent with the investigational vaccine.

It is tested eligible by National Institute for Food and Drug Control according to the *Manufacturing and Quality Control Requirements of Inactivated SARS-CoV-2 Vaccine（Vero Cell）(Draft Version)*. The vaccine is injectable with the specification of 0.5mL/container. It contains no SARS-CoV-2 antigen.

6.4 Storage and Transportation

Vaccines should be stored and transported at 2~8°C, preventing from light.

6.5 Administration Rout and Schedule

Eligible subjects are intramuscularly injected at the lateral deltoid muscle of the upper arm, with a single dose of 0.5ml investigational vaccine or control vaccine,twodoses of primary immunization schedule will be given at 0,28 days, respectively, one dose of booster immunization will be given 6 months or 1 year after the second dose (1 year for the phase Ⅰ clinical tria, 6 months for the phase II clinical trial 0.5ml per dose ,the vaccine should be shaken well before inoculation.

6.6 Information of Investigational Vaccine

The information of Investigational vaccine is as below:

Table 8 Information of Investigational Vaccine

Group	Name	Pacakge	Antigen content	Manufacturer	Batch number	Expiration date
Low dosage vaccine	Inactivated SARS-CoV-2 Vaccine (Vero Cell)	Pre-filled syringe	300SU/ 0.5ml	Sinovac (R&D)	20200307	2023.03.20
Medium dosage vaccine	Inactivated SARS-CoV-2 Vaccine (Vero Cell)	Pre-filled syringe	600SU/ 0.5ml	Sinovac (R&D)	20200412	2023.04.08
High dosage vaccine	Inactivated SARS-CoV-2 Vaccine (Vero Cell)	Pre-filled syringe	1200SU/ 0.5ml	Sinovac (R&D)	20200411	2023.04.07
Placebo	Aluminum hydroxide diluent	Pre-filled syringe	0SU/ 0.5ml	Sinovac (R&D)	2020022801	2023.02.27

6.7 Package

The vaccine will be packed in a box with a label. The label of the vaccine is shown below. See "8.4 randomization and blinding" for the vaccine numbering rules on the label.

Phase I/II of Clinical Trial of SARS-CoV-2 Vaccine (Vero Cell), Inactivated<br>PRO-nCOV-1002<br>**E001**<br>Only for clinical study, stored at 2-8°C<br>Expiration date:

The box diagram is as below:

Phase I/II of Clinical Trial of SARS-CoV-2 Vaccine (Vero Cell), Inactivated<br>PRO-nCOV-1002<br>Serial No.:<br>Only for clinical study, stored at 2-8℃<br>Expiration date:

7 Objective

To evaluate the safety and immunogenicity of inactivated SARS-CoV-2 Vaccine (Vero cell) in older adults aged ≥60 years old.

7.1 Phase I Clinical Trial

To evaluate the safety, tolerance and preliminary immunogenicity of different dosage vaccine in older adults aged ≥60 years old.

7.2 Phase II Clinical trial

To evaluate the safety and preliminary immunogenicity of different dosage vaccine in older adults aged ≥60 years old so as to determine the appropriate dosage for further clinical evalution.

8 Design

8.1.1 Overall Design

Randomized, double blind and placebo control clincal trial.

8.1.2 Sample Size Considerations

Phase I Clinical trial: according to the requirements of the *Good Clincial Practic* and *Provisions for Drug Registration*, phase I clinical trial is a small-scale study with a sample size of 20-30 people to preliminarily evaluate the safety. The total sample size of phase I are 72 subjects, with 48 sujects receiving medium or high dosage investigational vaccine. The sample size meets the requirements of phase I clinical trial.

Phase Ⅱ Clincal trial: according to the requirments of the *Good Clincial Practic* and *Provisions for Drug Registration*, the phase II clincal trial mainly evaluates the immunogenicity and safety of different dosage vaccine in the targeted population and the sample size is more than 300.
The total number of sujects in phase II is 350, with subjects receiving low, medium, high dosage vaccine and placebo as 100, 100, 100 and 50, respectively. A total of 300 subjects receive the low, medium and high dosage vaccines, which meets the requirement of phase II clinical trial.

8.2 Study Endpoint

8.2.1 Endpoint of Phase I

8.2.1.1 Primary Endpoint

- Incidence of adverse reactions within 28 days after each dose of vaccination.

8.2.1.2 Secondary Endpoint

- Incidence of adverse reactions within 7 days after each dose of vaccination;
- Incidence of SAEs within 6 months after t booster immunization;
- Seroconversion rate, seropositive rate, GMT and GMI of neutralizing antibody on the 28th day after primary immunization;
- Seroconversion rate, seropositive rate, GMT and GMI of neutralizing antibody on the 28th day after the first dose vaccination.

8.2.1.3 Exploratory Endpoint

- Seropositive rate and GMT 6 months and 12 months after primary immunization;
- Seropositive rate, GMT and GMI on 28 days after booster immunization;
- Seropositive rate and GMT 6 months after booster immunization.

8.2.2 Endpoint of Phase II

8.2.2.1 Primary Endpoint

- Seroconversion rate of neutralizing antibody 28 days after two doses of vaccination;

8.2.2.2 Secondary Endpoint

- Seropositive rate, GMT, and GMI of the neutralizing antibody 28 days after two doses of vaccination;

- Incidence of adverse reactions within 28 days after each dose vaccination;
- Incidence of adverse reactions within 7 days after each dose vaccination;
- Incidence of SAEs from the beginning of the vaccination to 12 months after booster immunization.

8.2.2.3 Exploratory Endpoint

- Seropositive rate and GMT of neutralizing antibody 6 months after two doses of vaccination;
- Seropositive rate, GMT, and GMI of the neutralizing antibod at 7 days (or 14 days) and 28 days after booster immunization;
- Seropositive rate and GMT 6 months and 12 months after booster immunization.

8.3 Study Plan

8.3.1 Study Plan of Phase I Clinical Trial

Single centered, randimized, double blinded and placebo controlled clinical trial design is adopted in Phase I. A total of 72 healthy older adults aged≥60 years old are selected as subjects. After informed consent, subjects who pass the physical examination, meet the inclusion criteria and did not meet the exclusion criteria will be enrolled into the study. Enrolled subjects receive two doses of injection according to primary immunization schedule (day 0,28). Subjects are enrolled with a dose-escalation manner, with 36 at medium dosage stage which will run-in first, following by 36 at high dosage stage. The subjects enrolled in each dosage stage will be randomly assigned in a 2:1 ratio to receive vaccine or placebo. The high dosage stage vaccination will start only with the condition that safety observation 0~7 days after the first dose of the medium dosage stage vaccination is finished, and the good safety profiles is confirmed. All enrolled subjects received 1 dose of booster immunization 1 year after primary immunization.

To evaluate the safety, the immediate reactions occur within 30 minutes after each dose of vaccination will be observed on site; the local and systemic solicited adverse events (AEs) occur withi 0~7 days after each dose vaccination, as well as the unsolicited AEs from the beginning of the vaccination to 28 days after the whole schedule vaccination will be collected; additionally, the SAEs from the beginning of the vaccination until 6months after booster immunization will be collected. Venous blood will be collected from all subjects at different time points before and after vaccination for serum neutralizing antibody test, to evaluate the primary immune effect , booster immune effect and immune persistence of the vaccine.

The detailed study plan of phase I clinical trial is shown in the table 6

Table 9 Study Plan of Phase I Clinical Trial

Primary immunization (day)	Booster immunization (day)	Phase	Sample size				Blood sampling time (day)
			Miedum dosage	High dosage	Placebo	Total	
0,28	388	Medium dosage stage	24	-	12	36	0,28,56,208,388,416,568
		High dosage stage	-	24	12	36	
		**Total**	**24**	**24**	**24**	**72**	

Note: Please refer to "10.1 Visit Plan" for the time window.

8.3.2 Study Plan of Phase II Clinical Trial

Single centered, randomized, double blinded and placebo controlled clinical trial design is adopted. The phase II clinical trial will start only with the condition that safety observation 0~7 days after the first dose of the high dosage stage vaccination in phase I is finished, and the good safety profiles is confirmed by the DMC. A total of 350 healthy older adults aged ≥60 years old are selected as subjects. After informed consent, subjects who pass the physical examination, meet the inclusion criteria and didn't meet the exclusion criteria will be enrolled into the study. Subjects

will receive two doses of injection at the primary immunization schedule of day 0,28. The subjects will be randomly assigned in a 2:2:2:1 ratio to receive the low dosage, medium dosage, high dosage vaccine or placebo. All enrolled subjects received 1 dose of booster immunization 6 months after primary immunization.

The immediate reactions occur within 30 minutes after each dose of vaccination will be observed on site. The local and systemic solicited adverse events (AEs) occur withi 0~7 days after each dose vaccination, as well as the unsolicited AEs from the beginning of the vaccination to 28 days after the whole schedule vaccination will be collected. Additionally, the SAEs from the beginning of the vaccination until 12 months after booster immunization will be collected. Venous blood is collected at different time points before and after the vaccination for serum neutralizing antibody test, to evaluate the primary immune effect, booster immune effect and immune persistence.

The detailed study plan of phase II clinical trial is shown in the table 7.

Table 10Study Plan of of Phase II Clinical Trail

Primary immunization (day)	Booster immunization (day)	Sample Size					Blood sampling time (day)
		Low dosage	Medium dosage	High dosage	Placebo	Total	
0,28	208	100	100	100	50	350	0,56,208,215(or 222)*,236,388,568

* Subjects with study numbers E101 to E275 were tested on the 7th day (Day 215) after booster immunization; Subjects with study numbers E276 to E450 were tested on the 14th day (Day 222) after booster immunization.

Note: Please refer to "10.1 Visit Plan" for the time window.

8.4 Randomization and Blinding

8.4.1 Randomization

In phase I and phase II clinical trial, the blinding code should be generated separately by the randomization statistitian by the method of block randomization using SAS software (version 9.4). The blinding code refers to the list of the correspondence between the random number and the trial products (i.e., vaccine or placebo), which is prepared in duplicate and should be sealed after the completion of the blind coding. The original copy should be kept by the investigator for unblinding of the trial, and the duplicate copy should be kept by the sponsor. In the phase I clinical trial, the vaccine (or placebo) numbers are E001-E072. In the phase II clinical trial, the vaccine (or placebo) number numbers are E101-E450.

The blinding code of the backup vaccine (or placebo) is also generated by the randomization statistian using SAS software (version 9.4). In the phase I clinical trial, the backup vaccine (or placebo) is prepared in a 1:1:1 ratio of medium dosage, high dosage vaccine, and placebo, and the backup vaccine (or placebo) numbers are X001-X012. In phase II clinical trial, the backup vaccine (or placebo) is prepared in a 2:2:2:1 ratio of low dosage, medium dosage, high dosage vaccine, and placebo, and the backup vaccine (or placebo) numbers are Y001-Y056.

In case of the circumstances such as color change and damage of the trial products, the inoculation personnel should report to the person in charge of the site and principle investigator, the initiation procedure of the backup vaccine should be started up, a backup vaccine (or placebo) number should be obtained through the online backup vaccine acquisition system, and the corresponding backup vaccine should be used instead of the problem vaccine.

All trial vaccines and placebos will be pasted with blind labels. See "6.7 vaccine packaging" for label style. The subjects should be inoculated with the vaccine labelled with the number which is in accordance with their study number assigned at the enrolment.

8.4.2 Blinding

In this study, a blind design is adopted, in which the randomization statistician and other personnel who do not participate in the trial will engaged in vaccine (or placebo) blinding, i.e. pasting the printed number label to the

specified location of the vaccine (or placebo), according to the generated blinding code. The whole process of vaccine (or placebo) blinding will be supervised by the randomization statistician. The blinding code should be sealed after the completion of the blind coding. The whole process of blinding must be recorded in writting. Personnel who conduct blinding are forbidden to participate in other relevant work of this clinical trial, and should not disclose the blinding code to any person participating in this clinical trial.

8.4.3 Emergency Unblinding

Except for the blinding, the statistician should prepare emergency envelopes reserved for the potential emergency unblinding. In each envelope, there is a random password which can correspond to any study number, and the actual group of this study number can be disclosed through the online unblinding system. Each random password represents a chance of unblinding, that is to say, only one study number can be unblinded using a certain password, and then it will be invalid, and it is also invalid for the already unblended study number. In this study, three emergency envelopes are prepared in the phase I clinical trial, and 10 emergency envelopes are prepared for the phase II clinical trial. All the emergency envelopes are kept by the personnel in charge of the study site. Sealed status of the emergency envelopes should be checked during the blind audit process.

During the study, if the principle investigator and the sponsor jointly decide it is necessary to unblind in an emergency, the person in charge of the site shall open the emergency letter, log in to the online emergency unblinding system with the random code of unblinding in the envelope and conduct the emergency unblinding following the operation prompts, and make relevant records. Subjects with this study number will discontinue the trial and be treated as dropout, and the principle investigator will record the reason for discontinuation in the case report form (CRF). The opened emergency envelope should be properly kept and returned to the sponsor after the study is completed.

8.4.4 Unblinding Regulations

The phase I and phase II clinical trials will be unblinded according to the following time points: the unblinding will be conducted after the serum antibody test results of the 28th day after the whole schedule vaccination are obtained. The unblinding will be jointly implemented by the sponsor, the principle investigator and the statistical party, and a record of unblinding should be kept. After unblinding, the investigators responsible for the observation and evaluation of the subjects and the CRAs responsible for the source data verification should be kept blind until the database is finally locked.

8.4.5 Flow Chart

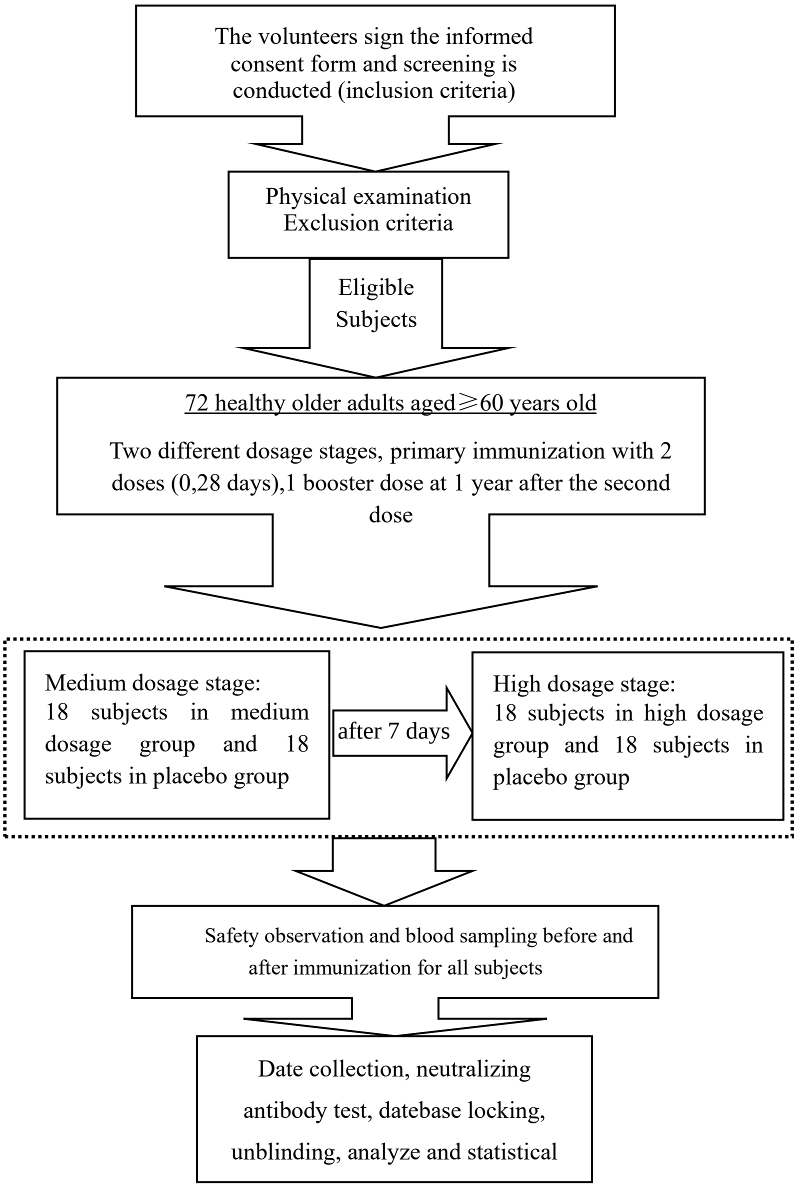

Figure 7 Flow Chart of Phase I Clinical Trial of Inactivated SARS-COV-2 Vaccine (Vero cell)

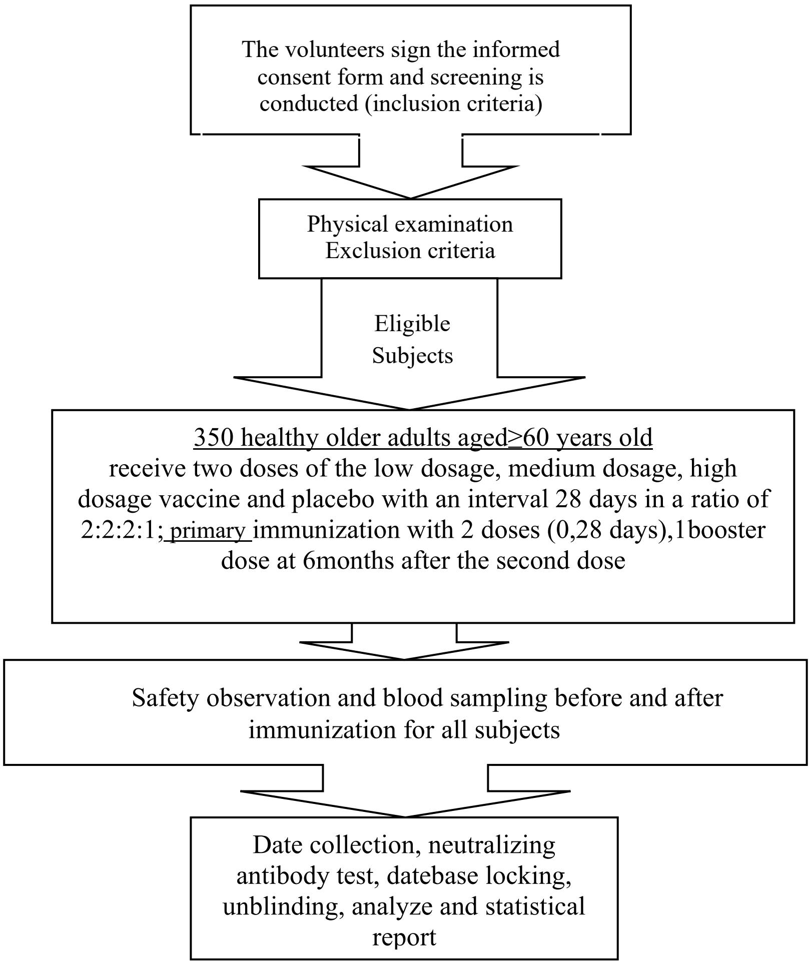

Figure 8 Flow chart of clinical trial phase II of Inactivated SARS-COV-2 vaccine (Vero cell)

8.5 Study Time

8.5.1 Duration of Clinical Trial

Clinical trial is estimated that the study is 22 months

8.5.2 Estimated Time for Subjects to Participate in the Trial

It is estimated that the maximum study duration is 20 months for each subject.

8.6 Trial Suspension and Early Termination

Criteria of trial suspension:

- One or more than one case of the grade 4 adverse events related to vaccination (local, systmic) occur;
- More than 15% of the subjects have grade 3 and above adverse events related to vaccination , including local reaction, systemic reaction and vital signs.

Early Termination Criteria of the Trial:

- After the clinical trial is suspended, the principle investigator and sponsor will jointly discuss and decide whether to early terminate the trial;
- The sponsor requests to fully terminate the trial and has explained reasons;
- The ethics committee requests to fully terminate the trial and has explained reasons;
- The administrative departments require to the fully terminate the trial and has explained reasons.

8.7 Protocal Violation and Deviation

Refers to any change and non-compliance with the clinical trial protocol design or process. The behavior that does not affect the rights and interests, safety and benefits of the subjects, or the integrity, accuracy and reliability of the data as well as the safety or primary indicators belong to the protocol deviation; those that affect the rights and interests, safety and benefits of the subjects, or the integrity, accuracy and reliability of the data and the safety or primary indicators belong to serious protocol deviation (i.e. protocol violate).

For the protocal deviation / violation during the study, the on-site investigators shall report the fact, process, causes and impact of the incident to the research responsible institution. The principle investigator shall give opinions on the handling of the incident. The protocol deviation / violation report shall be submitted to the Ethics Committee for review and approval.

The investigators should carry out targeted training for relevant staff in the related links of the violation of the protocol to prevent the recurrence of similar incidents, and record the training process.

9 Study Population

9.1 Inclusion Criteria

(1) Healthy subjects aged ≥60 years old;
(2) Be able to understand and sign the informed consent voluntarily;
(3) Provide legal identification.

9.2 Exclusion Criteria

(1) Travel / residence history of Wuhan city and surrounding areas or other communities with case reports within 14 days prior to the entry;

(2) Contact with SARS-CoV-2 infected persons (positive for nucleic acid detection) within 14 days prior to the netry;

(3) Contact patients with fever or respiratory symptoms from Wuhan city and surrounding areas, or from communities with case reports within 14 days prior to the entry;

(4) Two or more cases of fever and / or respiratory symptoms in a small cantact area of subjects, such as family, office, school class or other places within 14 days prior to the entry;

(5) History of SARS;
(6) History of SARS-CoV-2 infection;
(7) History of asthma, allergy to vaccines or vaccine ingredients, and serious adverse reactions to vaccines, such as urticaria, dyspnea, angioneuroedema;
(8) Congenital malformation or developmental disorder, genetic defect, severe malnutrition, etc;
(9) Autoimmune disease or immunodeficiency / immunosuppression;
(10) Serious chronic disease, serious cardiovascular disease, hypertension and diabetes that cannot be controlled by drugs, hepatorenal disease, malignant tumor, etc;
(11) Serious nervous system disease (epilepsy, convulsion or convulsion) or psychosis;
(12) Thyroid disease or history of thyroidectomy, spleenlessness, functional spleenlessness, spleenlessness or splenectomy resulting from any condition;
(13) Diagnosed abnormal blood coagulation function (eg, lack of blood coagulation factors, blood coagulopathy, abnormal platelets) or obvious bruising or blood coagulation;
(14) Immunosuppressive therapy, cytotoxic therapy, inhaled corticosteroids (excluding allergic rhinitis corticosteroid spray therapy, acute non-complicated dermatitis superficial corticosteroid therapy) in the past 6 months;

(15) Long history of alcohol or drug abuse;
(16) Receipt of blood products in the past 3 months;
(17) Receipt of other investigational drugs in the past 30 days;
(18) Receipt of attenuated live vaccines in the past 14 days;
(19) Receipt of inactivated or subunit vaccines in the past 7 days;
(20) Acute diseases or acute exacerbation of chronic diseases in the past 7 days;
(21) Axillary temperature >37.0°C;
(22) According to the investigator's judgment, the subject has any other factors that are not suitable for the clinical trial.

9.3 Exclusion Criteria for the Second Dose and the Tird Dose

The subjects who experience any of events in the following (1) to (4) are forbidden to continue vaccination, but they can continue other study steps according to the investigator' judgement. For the subjects who experience any of the events in the following (5) to (6), the investigator will judge whether vaccination will be continued. For the subjects who experience any of the events in the following (7) to (10), the vaccination can be delayed within the protocol-permitted time window.
(1) Similar vaccines other than the investigational vaccines were used durng the study;
(2) Any serious adverse reactions which have a causal relationship with the vaccination;
(3) Severe anaphylaxis or hypersensitivity after vaccination (including urticaria/rash appears within 30 minutes after vaccination)；
(4) Any confirmed or suspected autoimmune disease or immunodeficiency disease, including human immunodeficiency virus (HIV) infection；
(5) Acute or newly onset chronic disease after vaccination；
(6) Other reactions (including severe pain, severe swelling, severe limitation of movement, persistent high fever, severe headache or other systemic or local reactions) judged by the investigators;
(7) Acute diseases occur during vaccination (acute disease means moderate or severe disease with or without fever);
(8) Axillary temperature >37.2°C during vaccination;
(9) Have vaccinated with subunit vaccine or inactivated vaccine within 7 days, immuned with live attenuated vaccine within 14 days;
(10) According to the investigator's judgment, the subject has any other factors that affect vaccination.

9.4 Subject Withdraw and Suspending Criteria

(1) Subjects request to withdraw；

(2) Intolerable adverse events, whether or not related to the investigational product;

(3) Subjects are not allowed to participate in this trial due to their health status;

(4) In case of any abnormal clinical manifestations of the subjects, the researcher should determine whether it is related to the vaccine, and judge whether the subjects suspend the clinical trial;

(5) Any other reasons considered by the investigator.

If the trial vaccine has been inoculated to the subject before suspending, the clinical trial data of the subject will be used for safety analysis. Subjects could not be replaced in the trail. After the subjects who have been vaccinated in the clinical trial withdraw or suspend the trial, the researcher should provide necessary guidance for any clinical situation related to the trial, and follow up until the a definitive diagnosis is obtained, or the health condition stabilizes or recovers.

10 Method and Procedure

10.1Visit Plan

(1) Phase Ⅰ clinical trial visit Plan

Table 11 Follow-up Visits Schedule in Phase Ⅰ

**Visit No.**		**1**	**2**	**3**	**4**	**5**	**6**	**7**	**8**	**9**	**10**
Date of Visit	D-14 ～D0	D0	D8[e]	D28[e]	D36[e]	D56[e]	D208[e]	D388[e]	D395[e]	D416[e]	D568[e]
Preliminary notification, subject enrolment	X										
Informed consent		X						X			
Demographic information		X									
Regular examination		X									
Inclusion/exclusion criteria screening[a]		X		X				X			
Neutralizing antibody test				X[f]		X	X	X		X	X
Vaccination[b]		X		X				X			
Subject self-recording of the safety observation on diary cards[c]		X	X	X	X	X			X	X	
Adverse reaction/event monitoring (including level 3 or higher, SAE)[cd]		X	X	X	X	X	X	X	X	X	X
Records of concomitant use of drug/vaccine[cd]		X	X	X	X	X	X	X	X	X	X

a) Before each dose vaccination, inclusion/exclusion criteria screening is required.

b) Subjects will be observed for 30 minutes on site to determine the situation of adverse events, especially acute allergic reactions, and then followed by regular follow-ups as required.

c) Safety observation includes assessment of adverse reactions/events and temperature measurement. Body temperature should be measured every day within 0~7 days after each dose vaccination and whenever fever is suspected. Safety observation data are required to be recorded in the diary cards after each dose administration. The investigator regularly interviews the subjects to verify and record adverse events and concomitant use of drugs / vaccines.

d) During D56-D388 and D416-D568, only SAE and drug use associated with SAE are collected.

e) See "Visit Plan" for the time window.

Visit Plan:

Visit1—Day 0—eligible subjects enrolled, collect blood, and the first dose administration.

Visit 2—Day 8 after the first dose - verify the safety observations and concomitant use of drug and other vaccine.

Visit 3—Day 28 day (+10 day) after the first dose- verify safety observations and concomitant use of drug and other

vaccine, collect blood and the second dose administration.
Visit 4—Day8 after the second dose- verify the safety observations and concomitant use of drug and other vaccine.
Visit 5—Day28 (+10 day) after the second dose - verify the safety observations and concomitant use of drug and other vaccine, and collect blood.
Visit 5~Visit 6—verify SAE observation, drug use associated with SAE, and other special circumstances.
Visit 6—Day 180 (+30 day) after the second dose-verify SAE observation, drug use associated with SAE, and other special circumstances, and collect blood.
Visit 6~Visit 7—verify SAE observation, drug use associated with SAE, and other special circumstances.
Visit 7—Day 360 (+30 day) after the second dose-verify SAE observation, drug use associated with SAE, and other special circumstances, collect blood, and booster immunization.
Visit 8—Day 8 after booster immunization - verify the safety observations and concomitant use of drug and other vaccine.
Visit 9—Day 28 (+10 day) after booster immunization - verify the safety observations and concomitant use of drug and other vaccine, and collect blood.
Visit 9~Visit10— verify SAE observation, drug use associated with SAE, and other special circumstances.
Visit 10—Day180 (+30 day) after booster immunization - verify SAE observation, drug use associated with SAE, and other special circumstances, and collect blood.

（2）Phase Ⅱ clinical trial visit Plan

Table 12 Follow-up Visits Schedule ofPhase Ⅱ

		**Primary immunization**						**Booster immunization**					
Visit No.		1	2	3	4	5	6	7	8	9	10	11	12
Date of Visit	D-14～D0	D0	D8[e]	D28[e]	D36[e]	D56[e]	D208[e]	D208[e]	D215[e]	D222[e,g]	D236[e]	D388[e]	D568[e]
Preliminary notification, subject enrolment	X												
Informed consent		X							X				
Demographic information		X											
Regular examination		X											
Inclusion/exclusion criteria screening[a]		X		X				X					
Neutralizing antibody test		X				X	X		X[f]	X	X	X	X
Vaccination[b]		X		X				X					
Subject self-recording of the safety observation on diary cards[c]		X	X	X	X	X		X	X	X	X		
Adverse reaction/event monitoring (including level 3 or higher, SAE) [cd]		X	X	X	X	X	X	X	X	X	X	X	X
Records of concomitant use of drug/vaccine [cd]		X	X	X	X	X	X	X	X	X	X	X	X

a) Before each dose vaccination, inclusion/exclusion criteria screening is required.
b) Subjects will be observed for 30 minutes on site to determine the situation of adverse events, especially acute allergic reactions, and then followed by regular follow-ups as required.
c)Safety observation includes assessment of adverse reactions/events and temperature measurement. Body temperature should be measured every day within 0~7 days after each dose vaccination and whenever fever is

suspected. Safety observation data are required to be recorded in the diary cards after each dose administration . The investigator regularly interviews the subjects to verify and record adverse events and concomitant use of drugs / vaccines.

d) During D56-D208 and D236-D568, only SAE and drug use associated with SAE are collected.

e) See "Visit Plan" for the time window.

f) Only applicable to subjects with study numbers E101 to E275.

g) Only subjects with study numbers E276 to E450 were visited.

Visit Plan:

Visit1—Day 0—eligible subjects enrolled, collect blood, and the first dose administration.

Visit 2—Day 8 after the first dose - verify the safety observations and concomitant use of drug and other vaccine.

Visit 3—Day 28 day (+10 day) after the first dose- verify safety observations and concomitant use of drug and other vaccine, collect blood and the second dose administration.

Visit 4—Day 8 day after the second dose- verify the safety observations and concomitant use of drug and other vaccine.

Visit 5—Day 28 (+10 day) after the second dose - verify the safety observations and concomitant use of drug and other vaccine, and collect blood.

Visit 5~Visit 6—verify SAE observation, drug use associated with SAE, and other special circumstances.

Visit 6—Day 180 (+30 day) after the second dose-verify SAE observation, drug use associated with SAE, and other special circumstances, and collect blood.

Visit 7—Day 180 (+90 day) after the second dose-verify SAE observation, drug use associated with SAE, and other special circumstances, collect blood, and booster immunization.

Visit 8—day 7 (+3 day) after booster immunization - verify the safety observations and concomitant use of drug and other vaccine, in addition, collect blood (only for subjects E101 to E275).

Visit 9—Day 14 (+3 day) after booster immunization - verify the safety observations and concomitant use of drug and other vaccine, and collect blood (Only for subjects E276-E450).

Visit10—Day 28 (+10 day) after booster immunization - verify the safety observations and concomitant use of drug and other vaccine, and collect blood.

Visit 10~Visit11 -verify SAE observation, drug use associated with SAE, and other special circumstances.

Visit 11—Day 180(+30 day) after booster immunization - verify SAE observation, drug use associated with SAE, and other special circumstances and collect blood.

Visit 11~Visit12 -verify SAE observation, drug use associated with SAE, and other special circumstances.

Visit12—Day 360 (+30 day) after booster immunization- verify SAE observation, drug use associated with SAE, and other special circumstances and collect blood.

10.2Recruitment and Informed Consent

Recruitment notices will be issued to volunteers who meet the recruitment criteria. The informed consent should be explained to the volunteers in detail. In the premise of voluntary participation, the volunteers and the investigator jointly sign the informed consent in duplicate, and the volunteers keep the copies.

10.3Screening and Random Enrollment

The subjects who meet the inclusion criteria and don't meet the exlusion criteria are eligible to be enrolled into the study. The screening number is ES+ screening sequence number, such as "ES0001". The enrolled subject will be assigned a study number in the order of enrollment. In the phase I clinical trial and the phase II clinical trial, study numbers of subjects are E001-E072 and E101-E450 respectively.

10.4Vaccination

According to the study number of the subject, the vaccinator takes out the corresponding vaccine labelled with the same number and opens the package box, checks the information of label on the syringe, label in the package box,

and label on the outer surface of package box, the vaccination should be carried outs with the condition that information on the three labels are confirmed consistency. After vaccination, the label in the backage box should be removed and pasted on the specific location of the original logbook, simultaneously, the vaccination information should be recorded in the original logbook.
See "8.3 Study plan" for immunization schedules.

10.5Safety Follow-up Observation

Subjects will be observed for 30 minutes on site after each dose of vaccination. Diary cards and contact cards are distributed to subjects to record the adverse events within 0~7 days and 8~28 days respectively. The investigators explain the judgment, measurement, recording, precautions and reporting method of adverse events. Systematic observation is carried out within 7 days after vaccination. Subjects are required to closely observe their own symptoms and vital signs and fill in the diary card every day. The investigators verify the adverse events on the 8th days after vaccination through face-to-face interviews on all subjects (those who do not face-to-face interviews are conducted by telephone), collect diary cards and distribute contact cards to record the adverse events within 8~28 days. The investigators verify the adverse events on the 28th days and collect contact cards.
The subjects are informed to record the adverse events at any time. Acute allergic reactions, serverity level 3 and above adverse events and SAE should be reported to the investigators timely. After the investigators are informed, they should conduct investigation, verification and follow-up until the adverse event is solved, and finally complete the detailed investigation and follow-up records, which should include the following contents:

- Description of adverse events
- Start time and end time of adverse events
- Severity level
- Relevance to vaccination
- Laboratory testing results
- Treatment measures

Timely treatment should be provided with regard to the acute allergic reactions and severity level 3 and above advere events, in order to relieve the sufferings of the subjects as soon as possible; drug treatment and medical treatment during each follow-up should be recorded in detail.

10.6Blood Sample Collection

- **Sample Collection Plan & Numbering Principle**

Blood samples (2.5-3.5ml each time) from subjects are collected before and after immunization according to “10.1 Visit Plan”. The sample numbering rule is “study number + collection serial number”.

- **Sample Management**

All the samples collected on site should be sent to laboratory timely, completing the handover with the laboratory personnel. Serum should be isolated timely and placed in two tubes (i.e. A tube for detection and B tube for backup, the amount of serum in A tube should be no less than 0.5ml). The serum isolation process should be recorded, and the serum should be stored under -20℃ or lower temperature. All the processes of sample handover, serum insolation, and sample preservation should be recorded. Sample submission record should be filled in for all samples, and the temperature control record of the submission process should be kept.

10.7Safety Assessment

10.7.1 Safety Obervation Index

Solicited local adverse events: pain, induration, swelling, redness, rash, pruritus
Solicited systemic adverse events (including vital signs): fever (axillary temperature), acute allergic reaction, skin and mucosa abnormality, diarrhea, anorexia, vomiting, nausea, muscle pain, headache, cough, fatigue.

10.7.2 Definition of Adverse Events/Reactions

The safety of vaccines will be evaluated according to the scope, intensity, and severity of the local adverse events, systemic adverse events as well as abnormality of vital signs, and the correlation of the above events with vaccination. All adverse medical events occurring during the trial (since signing of the informed consent form) should be collected and recorded.

- Adverse events (AE): Adverse medical events that occur after vaccination, but are not necessarily causally related to the trial vaccine.
- Adverse reactions: the adverse events related to the trial vaccination during the vaccination according to the prescribed dose and procedure.
- Serious adverse event (SAE): it refers to the events during the clinical trial that need hospitalization treatment, prolong hospitalization time, disability, affect working ability, endanger life or death, cause congenital malformation, etc.
- Solicitation/non-solicitation adverse events: In this trial, the solicitation period is 0-7 days after each dose of vaccination, and the non-solicitation period is 8-28 days. The solicited adverse events refer to the solicited symptoms occur within the solicitation period, and the unsolicited adverse events refer to the unsolicited symptoms occur within the solicitation period, and any symptoms occur within the non-solicitation period.

10.7.3 Outcome of adverse events

The outcomes of adverse events included: (1) recovered (2) not yet recovered (3) recoverd but has sequela (4) death (5) loss of follow-up/unknown.

10.7.4 Correlation of Adverse Events with Vaccines

The investigators should try their best to explain the adverse events, and assess the possible casual relationship, i.e. the causal relationship between investigational vaccine and alternative causes (e.g. history of underlying disease, concomitant treatment). This applies to all AEs including serious and non-serious ones.
Causality assessment will be determined by the extent to which an event can be reasonably explained in one or more of the following areas:
Reactions with similar nature have been observed for the similar products;
The same event has been reported in the literatures of the drug products of the similar type;
The event appears with vaccination of the investigational vaccine and recurs after re-vaccination of the investigational vaccine.
Causality of AE with vaccination should be assessed by the investigator on the basis of the following questions to determine whether there is a reasonable possibility that the AE is caused by vaccination:
a. Certainly related : there is evidence of vaccination of experimental vaccine; the time sequence of adverse events and vaccination is reasonable; the occurrence of adverse events is more reasonable than other reasons; repeated vaccination is positive; adverse events are consistent with previous knowledge of this or this kind of vaccine..
b. Probably related: there is evidence of vaccination of experimental vaccine; the time sequence of adverse events and vaccination is reasonable. It is more reasonable to explain adverse events by experimental vaccine than by other reasons.
c. Possibly related: there is evidence of vaccination of experimental vaccine; the time sequence of adverse events and vaccination is reasonable. The causes of adverse events can not be excluded from the experimental vaccine, but also may be caused by other reasons.
d. Possible unrelated: there is evidence of vaccination of experimental vaccine; adverse events are more likely to be caused by other reasons; repeated vaccination are negative or uncertain.
e. Definitely unrelated: the subjects do not use the experimental vaccine; or the occurrence of adverse events was illogical with the time sequence of vaccination; or there were other significant reasons that could lead to adverse events

10.7.5 Treatment of Adverse Event

The reactions such as redness, swelling, pain or (and) fever and general discomfort below grade 2 usually can

spontaneously disappear and special treatment is not needed.
The investigators will carry out the investigation and medical follow-up such as disease history, physical examination, necessary laboratory test, and necessary treatment if the subjects experience any adverse events of grade 3 and higher occurred within 28 days after the whole-schedule immunization, until the adverse events are solved. The corresponding investigation records including the symptoms, vital signs, diagnosis, and laboratory test results should be completed.
In case of the serious adverse event, investigator should promptly take the necessary measures and report within 24 hours.
During the study period, subjects with fever and respiratory symptoms such as cough should immediately go to the designated hospital for treatment. The throat swabs and anal swabs should be collected for nucleic acid testing, and CT examination should also be conducted to deterimine whether it is COVID-19. Once a COVID-19 occurs, it will be treated as SAE, especially it should be analysed that whether there is ADE phenomenon.

10.7.6 Reporting of Serious Adverse Events

(1) The study institution should establish the emergency plan for handling of SAE. The investigator should immediately take measures and make records after he/she is informed of SAE.
The investigator should report to the sponsor, the ethics committee and the local provincial drug regulatory authorities within 24 hours after the SAE is informed and submit the subsequent report.
The study institution/ the investigator should timely transfer the latest safety information report related to the clinical trial of the sponsor to the ethics committee. The report object can be adjusted according to existing regulations and the requirements of local regulatory authorities and ethics committees.
The ethics committee should receive the safety information reports such as SAE reports, timely grasp the occurrence and handling situations of SAE of the whole clinical trial, and carry out follow up review of the handling and reporting of SAE in the process of the clinical trial.

(2) When the sponsor receives information on vaccine safety from any source, an analysis and evaluation should be conducted, including the severity, relevance to the investigational vaccine, and whether it is an unexpected event.
During the drug clinical trial, the sponsor should quickly report the suspected unexpected serious adverse reactions (SUSARs) which are considered definitely or suspiciously related to the investigational drug in a maner of case safety report, according to the *Standards and Procedures for Rapid Reporting of Safety Data during Drug Clinical Trials*.

With regard to the fatal or life-threatening SUSARs, the sponsor should report them as soon as possible after beining informed within 7 natural days, and the relevant follow-up information should be reported within the subsequent 8 days (the day on which the sponsor is firstly informed is day 0);
with regard to non-lethal or life-threatening SUSARs, the sponsor should report them as soon as possible within 15 naturak days; For other information indicating serious safety risk, the sponsor should also report them to the national drug evaluation institution, and make a medical and scientific judgment on each situation.

After the initial report, the sponsor should continue to follow up the SAE and submit new information or changes to the previous report in the form of follow-up report within 15 days since the date of obtaining new information. The sponsor should not arbitrarily change the investigator's judgment on the correlation between SAE and vaccine. If the opinions of the sponsor and the investigator are inconsistent, opinions of both parties should be recorded in detail in the repor, and the adverse event should be reported according to higher management requirements.

Under special circumstances, the invesgator and sponsor should promptly provide SAE-related information and safety reports as required by regulatory authorities and ethics committees.

(3) The contact person of sponsor, ethics committee and Hebei Provincial Drug Administration is as follows:

The contact person of Sinovac：Jiayi Wang；Telephone：18518337983；Email:wangjy1755@sinovac.com；Fax:010-82890408.

The contact person of Ethics Committee of Hebei Center for Disease Control and Prevention: Yong doctor；Telephone：0311-86573167； Fax:0311-86573167.

Hebei Provincial Drug Administration: Email:hbfdasae@163.com.

10.7.7 Safety Evaluation Criteria

Solicited local adverse events, systemic adverse events and vital signs: The grading standard of solicited adverse events mainly refer to the *Guiding Principles for Grading Standards of Adverse Events in Clinical Trials of Vaccines for Prevention* (2019) [14] of NMPA. As shown in the table below, solicited adverse events and non-Solicited adverse events with the same symptoms are graded according to the following criteria:

Table 13 Severity Grading Criteria for Local Adverse Events

	Grade 1	Grade 2	Grade 3	Grade 4
Pain	Not affecting or slightly affecting physical activity	Affecting physical activity	Affecting daily life	Loss of basic self-care ability, or hospitalization
Induration*#	Diameter 2.5 to <5 cm or area 6.25 to <25 cm^2 without affecting or slightly affecting daily life	5 to <10 cm in diameter or 25 to <100 cm^2 in area or affecting daily life	Diameter ≥10 cm or area ≥100 cm^2 or ulceration or secondary infection or phlebitis or aseptic abscess or wound drainage or seriously affecting daily life	Abscess, exfoliative dermatitis, dermal or deep tissue necrosis
Swelling #	Diameter 2.5 to <5 cm or area 6.25 to <25 cm^2 without affecting or slightly affecting daily life	5 to <10 cm in diameter or 25 to <100 cm^2 in area or affecting daily life	Diameter ≥10 cm or area ≥100 cm^2 or ulceration or secondary infection or phlebitis or aseptic abscess or wound drainage or seriously affecting daily life	Abscess, exfoliative dermatitis, dermal or deep tissue necrosis
Redness#	Diameter 2.5 to <5 cm or area 6.25 to <25 cm^2 without affecting or slightly affecting daily life	5 to <10 cm in diameter or 25 to <100 cm^2 in area or affecting daily life	Diameter ≥10 cm or area ≥100 cm^2 or ulceration or secondary infection or phlebitis or aseptic abscess or wound drainage or seriously affecting daily life	Abscess, exfoliative dermatitis, dermal or deep tissue necrosis
Rash* #	Diameter 2.5 to <5 cm or area 6.25 to <25 cm^2 without affecting or slightly affecting daily life	5 to <10 cm in diameter or 25 to <100 cm^2 in area or affecting daily life	Diameter ≥10 cm or area ≥100 cm^2 or ulceration or secondary infection or phlebitis or aseptic abscess or wound drainage or seriously affecting daily life	Abscess, exfoliative dermatitis, dermal or deep tissue necrosis
Pruritus	Itching at injection site, relieved within 48 hours	Itching at injection site, did not alleviate within 48 h after treatment	Affecting daily life	NA

* In addition to directly measuring the diameter for grading evaluation, sclerosis and rash should also record the progress of measurement results.

# The maximum measured diameter or area should be used for induration and swelling, rash and red; evaluation and grading should be based on functional grade and actual measurement results, and higher grading indicators should be selected.

Table 14 Severity Grading Criteria for Systemic Adverse Events

	Grade 1	Grade 2	Grade 3	Grade 4
Acute allergic reaction*	Local urticaria (blisters), no treatment required	Local urticaria need treatment or mild angioedema, no treatment required	Extensive urticaria or angioedema treated or mild bronchospasm	Anaphylactic shock or life-threatening bronchospasm or laryngeal edema
Skin and mucosa abnormality	Erythema/pruritus/color change	Diffuse rash/maculopapular rash/dryness/desquamation	Blister/exudation/desquamation/ulcer	Exfoliative dermatitis involving mucosa, erythema multiforme, or suspected Stevens-Johnsons syndrome
Diarrhea	Mild or transient, 3-4 times/day, abnormal stool, or mild diarrhea lasting less than 1 week	Moderate or persistent, 5-7 times/day, abnormal stool, or diarrhea >1 week	>7 times/day, abnormal stool, or hemorrhagic diarrhea, orthostatic hypotension, electrolyte imbalance, requiring intravenous infusion >2L	Hypotensive shock, hospitalization
Anorexia	Decreased appetite, not affecting food intake	Decreased appetite, reduced food intake, not affecting body weight	Decreased appetite, and significantly reduced body weight	Need intervention (such as gastric tube feeding, parenteral nutrition)
Vomiting	1-2 times/24 hours without affecting activity	3-5 times/24 hours or affecting activity	>6 times within 24 hours or requiring intravenous fluid infusion	Hospitalization or other nutrition routes due to hypotensive shock
Nausea	Transient (<24 hours) or intermittent and basically normal food intake	Persistent nausea leads to reduced food intake (24-48 hours)	Persistent nausea leads to almost no food intake (>48 hours) or requires intravenous fluids	life threatening (e.g., hypotensive shock)
Muscle pain (non-inoculated site)	Does not affect daily activities	Slightly affects daily activities	Severe muscle pain, seriously affects daily activities	Emergency or hospitalization
Headache	Not affecting daily activities, no treatment required	Transient, slightly affecting daily activities, may need treatment or intervention	Seriously affecting daily activities, need treatment or intervention	Intractability, need emergency or hospitalization
Cough	Transient, no treatment required	Persistent cough, effective treatment	Paroxysmal cough, uncontrolled treatment	Emergency or hospitalization
Fatigue	Normal activity is weakened <48 hours, without affecting the activity	Normal activity is weakened by 20%~50%>48 hours, slightly affecting the activity	Normal activity is weakened by >50%, seriously affecting daily activities, unable to work	unable to take care of oneself, emergency or hospitalization
Vital Signs				
Fever, axillary temperature	37.3～<38.0	38.0～<38.5	≥38.5	≥39.5, Lasting more than 3 days

* Refers to type I hypersensitivity

For adverse events not covered in the above grading table, the intensity of adverse events was graded according to the following criteria:
Grade 1 (Mild): transient (<48 hours) or mild discomfort; no medical intervention/therapy required.

Grade 2 (Moderate): mild to moderate limitation in activity; some assistance may be needed; no or minimal medical intervention/ therapy required.

Grade 3 (Severe): Marked limitation in activity; some assistance usually required; medical intervention/therapy required, hospitalizations possible.

Grade 4 (Life threatening): Extreme limitation in activity, significant assistance required; significant medical intervention/therapy required, hospitalization or hospice care probable

Grade 5: Death.

10.8Concomitant Drugs and Vaccines

10.8.1 Concomitant Drugs

— In case of adverse events during the trial, necessary drug treatment and medical treatment are allowed.
— In case of serious allergic reactions or life-threatening events, first aid measures should be taken immediately.
— Investigators should record any information of concomitant drugs, including name, dosage form, dosage and administration route, administration time, etc.

10.8.2 Concomitant Vaccines

— Other vaccines should be administered at least 7 days apart after the investigational vaccine administration.
— Subjects can be administered with other vaccine such as rabies vaccinbe and tetanus vaccine against the emergency events during the clinical trial.
— Relevant information of the concomitant vaccine should be recorded in detail, including the vaccine name, administration, vaccination time etc.

10.9Immunogenicity Evaluation

Blood samples collected at different time points were tested for neutralizing antibody, and the seroconversion rate, seropositive rate, GMT and GMI of neutralizing antibody will be calculated.

10.9.1 Evaluation Criteria

The criteria for determining serum antibody positivity are as follows:
- The seropositivity is defined as neutralizing antibody titer is≥1:8
The criteria for determining serum antibody seroconversion are as follows
- The seroconversion is defined as a post-vaccination Nab titer ≥1:8 if seronegative (<1:8) at beseline, or a 4 fold increase of Nab titer if seropositive (≥1:8) at baseline.

10.9.2 Laboratory Test Methods

Microneutralization test method.

10.10 Data Management

10.10.1 Original Materials

The original materials include informed consent form, diary card, original logbook, etc., recording the following basic data:
- Trial name, subject number

- Demographic data
- Inclusion/exclusion criteria
- Vaccination records
- Follow-up date and date of discontinuation of the trial discontinuation date of the subject
- Adverse events/reactions and the corresponding treatment and outcome
- Concomitant medical treatment and other vaccinations

All data should have original records, which should be properly kept by investigaors in a dedicated space. The original data should be archived in the study site, which is the true and complete evidence for the particpantion of the subjects in the clinical trial.

The investigators should carefully, accurately and timely make the original records. All the collected original data should be recoreded on the same day with that of the data collection. Additionally, the raw data should be recorded using the black sign pen, and the mistake record should be crossed out with the correct content being written beside it along with the signature of the modifier, instead of be altered directly.

10.10.2 Case Report Forms (CRF)

"Electronic Data Capture (EDC) System" is adopted to establish the electronic CRF in this trial.

Electronic CRF is used to record the data of clinical trials, which is an important component of clinical trials and study reports. The electronic CRF is required to be inputted according to the system using instructions and CRF filling-in instructions, using the normative language.

All data on the electronic CRF are derived from the original material and are consistent with original data. Any entry, verification, modification, cleaning and quality control processes of electronic CRF data will be recorded in the EDC system. After data cleaning, the principle investigator should confirm the data in each CRF and sign with electronic signature.

Only investigator and approved staff are allowed to access to the EDC system during the trial period.

10.10.3 Data Lock

Final data verification should be carried out after the completion of all the data entry, verification and data cleaning work. The analyzed population, the situation of protocol violation as well as its impact on the analyzed population should be determined according to the assessment indicators, and then the database is locked.

10.10.4 Subject Privacy Protection and Data Utilization Scope

All information concerning the identity of the subject will be kept confidential and the name of the subject will not appear in any publication or report of the study. The study records will be provided to the sponsor representative in the presence of the investigator for the purpose of collecting medical data. In addition, the CRA, auditors, representatives of the Ethics Committee of the Hebei CDC, and representatives of the National Drug Administration (NMPA) can review the original material of subjects related to this study as required, to confirm the accuracy of the data collected in this study. The original data obtained in this study are only for publication of papers or results related to this project.

10.11 Statistical Analysis

10.11.1 Analysis Set

10.11.1.1 Safety Analysis Set (Safety Set, SS)

All randomized subjects who completed at least one vaccination were included in the safety evaluation set. Subjects who are vaccinated with the wrong vaccine will be tansfered to the group of actually administered vaccine according to the ASaT principle (All Subjects as Treated), for the safety evaluation. Safety sets in this study include generl safety set (SS), safety set of each dose. safety set of each dose was carried out according to the actual number of people vaccinated in each dose.

10.11.1.2 Immunogenicity Analysis Set

__Phase Ⅰ clinical trials__

Full Analysis Set of Primary Immunization (FAS): A population defined according to the principle of intent analysis (ITT), including all the subjects who have been randomized, have received at least one dose of vaccination, have finished blood collection before/after vaccination for at least one time with the corresponding antibody assay results provided. The subjects who are vaccinated with the wrong vaccine will be kept in the originally assigned group according to the ITT principle, for the immunogenicity evaluation.

28 days after 1st dose vaccination per-protocol set（Per-Protocol Set 1，PPS1）: A subset of FAS, including all the subjects who meet the inclusion criteria and don't meet the exclusion criteria, have been randomized, have finished the 1st dose vaccination within the protocol required time window, and have finished the blood collection before and 28 days after 1st dose vaccination with the corresponding antibody assay results provided. Subjects who meet the following conditions are not allowed to enter PPS1:

- Those who violate the study protocol;
- Those who are vaccinated with the wrong vaccine;
- Blood collection on 28 days after 1st dose vaccination beyond the protocol-required time window;
- Use of protocol prohibited vaccines or drugs:
  ① Other investigational or unlicensed products (drugs or vaccines);
  ② Long-term use (lasting more than 14 days) of immunosuppressive or other immunomodulatory drugs (inhaled or topical steroids are allowed);
  ③ Immunoglobulin and/or blood preparations.
- Newly diagnosed autoimmune diseases, including human immunodeficiency virus (HIV) infection;
- Other situations affecting the evaluation of immunogenicity of 28 days after 1st dose vaccination.

28 days after second dose vaccination per-protocol set（Per-Protocol Set 2，PPS2）: A subset of FAS, including all the subjects who meet the inclusion criteria and don't meet the exclusion criteria, have been randomized, have finished the 1st and 2nd dose vaccination within the protocol required time window, and have finished the blood collection before immunization and 28 days after 2nd dose vaccination with the corresponding antibody assay results provided. Subjects who meet the following conditions are not allowed to enter PPS2:

- Those who violate the study protocol;
- Those who are vaccinated with the wrong vaccine;
- 2nd dose vaccination or blood collection on 28 days after 2nd dose vaccination beyond the protocol-required time window;
- Use of protocol prohibited vaccines or drugs:
  ① Other investigational or unlicensed products (drugs or vaccines);
  ② Long-term use (lasting more than 14 days) of immunosuppressive or other immunomodulatory drugs (inhaled or topical steroids are allowed);
  ③ Immunoglobulin and/or blood preparations.
- Newly diagnosed autoimmune diseases, including human immunodeficiency virus (HIV) infection;
- Other situations affecting the evaluation of immunogenicity of 28 days after second dose vaccination.

Immune Persistence Set of Primary Immunization, 6 months after second dose vaccination (IPS-6): includes all the subjects who have received the 1st and 2nd dose vaccination, and blood sample was collected 6 months after the second dose vaccination, and there was an effective antibody titer value.

Immune Persistence Set of Primary Immunization, 12 months after second dose vaccination (IPS-12): includes all the subjects who have received the 1st and 2nd dose vaccination, and blood sample is collected 12 months after the second dose vaccination, and there is an effective antibody titer value.

Full Analysis Set for Booster (bFAS): A population defined according to the principle of intent analysis (ITT), including all the subjects who have been randomized, completed primary immunization, entered the booster immunization process, and have received the booster dose vaccination, and have finished blood collection before booster dose vaccination with the corresponding antibody assay results provided. The subjects who are vaccinated with the wrong vaccine will be kept in the originally assigned group according to the ITT principle, for the immunogenicity evaluation.

28 days after booster dose vaccination per-protocol set（Per-Protocol Set for Booster, bPPS）: A subset of bFAS, including all the subjects who meet the inclusion criteria and don't meet the exclusion criteria, have been randomized, have finished the 1st and 2nd dose vaccination and the booster dose 12 months after the second dose within the protocol required time window, and have finished the blood collection before booster immunization and 28 days after booster dose vaccination with the corresponding antibody assay results provided. Subjects who meet the following conditions are not allowed to enter bPPS:

- Those who violate the study protocol;
- Those who are vaccinated with the wrong vaccine;
- Booster dose vaccination or blood collection on 28 days after booster dose vaccination beyond the protocol-required time window;
- Use of protocol prohibited vaccines or drugs:
  ① Other investigational or unlicensed products (drugs or vaccines);
  ② Long-term use (lasting more than 14 days) of immunosuppressive or other immunomodulatory drugs (inhaled or topical steroids are allowed);
  ③ Immunoglobulin and/or blood preparations.
- Newly diagnosed autoimmune diseases, including human immunodeficiency virus (HIV) infection;
- Other situations affecting the evaluation of immunogenicity of 28 days after booster dose vaccination.

Immune Persistence Set of Booster Immunization, 6 months after booster dose vaccination (bIPS-6): includes all the subjects who have received the 1st and 2nd dose vaccination, completed the booster dose vaccination 1 year after the second dose, and blood sample is collected 6 months after the booster dose vaccination, and there is an effective antibody titer value.

<u>Phase II clinical trials</u>

Full Analysis Set of Primary Immunization (FAS): A population defined according to the principle of intent analysis (ITT), including all the subjects who have been randomized, have received at least one dose of vaccination, have finished blood collection before/after vaccination for at least one time with the corresponding antibody assay results provided. The subjects who are vaccinated with the wrong vaccine will be kept in the originally assigned group according to the ITT principle, for the immunogenicity evaluation.

Per-protocol set of Primary Immunization（Per-Protocol Set，PPS）: A subset of FAS, including all the subjects who meet the inclusion criteria and don't meet the exclusion criteria, have been randomized, have finished the 1st and 2nd dose vaccination within the protocol required time window, and have finished the blood collection before immunization and 28 days after 2nd dose vaccination with the corresponding antibody assay results provided. Subjects who meet the following conditions are not allowed to enter PPS:

- Those who violate the study protocol;
- Those who are vaccinated with the wrong vaccine;
- 2nd dose vaccination or blood collection on 28 days after 2nd dose vaccination beyond the protocol-required time window;
- Use of protocol prohibited vaccines or drugs:

  ① Other investigational or unlicensed products (drugs or vaccines);

  ② Long-term use (lasting more than 14 days) of immunosuppressive or other immunomodulatory drugs (inhaled or topical steroids are allowed);

  ③ Immunoglobulin and/or blood preparations.
- Newly diagnosed autoimmune diseases, including human immunodeficiency virus (HIV) infection;
- Other situations affecting the evaluation of immunogenicity of 28 days after second dose vaccination.

Immune Persistence Set of Primary Immunization, 6 months after second dose vaccination (IPS-6): includes all the subjects who have received the 1st and 2nd dose vaccination, and blood sample is collected 6 months after the second dose vaccination, and there is an effective antibody titer value.

Full Analysis Set for Booster (bFAS): A population defined according to the principle of intent analysis (ITT), including all the subjects who have been randomized, completed primary immunization, entered the booster immunization process, and have received the booster dose vaccination, and have finished blood collection before booster dose vaccination with the corresponding antibody assay results provided. The subjects who are vaccinated with the wrong vaccine will be kept in the originally assigned group according to the ITT principle, for the immunogenicity evaluation.

7 days after booster dose vaccination per-protocol set（Per-Protocol Set 1 for Booster, bPPS1）: A subset of bFAS, including all the subjects who meet the inclusion criteria and don't meet the exclusion criteria, have been randomized, have finished the 1st and 2nd dose vaccination and the booster dose 6 months after the second dose within the protocol required time window, and have finished the blood collection before booster immunization and 7 days after booster dose vaccination with the corresponding antibody assay results provided. Subjects who meet the following conditions are not allowed to enter bPPS1:

- Those who violate the study protocol;
- Those who are vaccinated with the wrong vaccine;
- Booster dose vaccination or blood collection on 7 days after booster dose vaccination beyond the protocol-required time window;
- Use of protocol prohibited vaccines or drugs:

  ① Other investigational or unlicensed products (drugs or vaccines);

  ② Long-term use (lasting more than 14 days) of immunosuppressive or other immunomodulatory drugs (inhaled or topical steroids are allowed);

  ③ Immunoglobulin and/or blood preparations.

- Newly diagnosed autoimmune diseases, including human immunodeficiency virus (HIV) infection;
- Other situations affecting the evaluation of immunogenicity of 7 days after booster dose vaccination.

14 days after booster dose vaccination per-protocol set（Per-Protocol Set 2 for Booster, bPPS2）: A subset of bFAS, including all the subjects who meet the inclusion criteria and don't meet the exclusion criteria, have been randomized, have finished the 1st and 2nd dose vaccination and the booster dose 6 months after the second dose within the protocol required time window, and have finished the blood collection before booster immunization and 14 days after booster dose vaccination with the corresponding antibody assay results provided. Subjects who meet the following conditions are not allowed to enter bPPS2:

- Those who violate the study protocol;
- Those who are vaccinated with the wrong vaccine;
- Booster dose vaccination or blood collection on 14 days after booster dose vaccination beyond the protocol-required time window;
- Use of protocol prohibited vaccines or drugs:

  ① Other investigational or unlicensed products (drugs or vaccines);

  ② Long-term use (lasting more than 14 days) of immunosuppressive or other immunomodulatory drugs (inhaled or topical steroids are allowed);

  ③ Immunoglobulin and/or blood preparations.
- Newly diagnosed autoimmune diseases, including human immunodeficiency virus (HIV) infection;
- Other situations affecting the evaluation of immunogenicity of 14 days after booster dose vaccination.

28 days after booster dose vaccination per-protocol set（Per-Protocol Set for Booster, bPPS）: A subset of bFAS, including all the subjects who meet the inclusion criteria and don't meet the exclusion criteria, have been randomized, have finished the 1st and 2nd dose vaccination and the booster dose 6 months after the second dose within the protocol required time window, and have finished the blood collection before booster immunization and 28 days after booster dose vaccination with the corresponding antibody assay results provided. Subjects who meet the following conditions are not allowed to enter bPPS:

- Those who violate the study protocol;
- Those who are vaccinated with the wrong vaccine;
- Booster dose vaccination or blood collection on 28 days after booster dose vaccination beyond the protocol-required time window;
- Use of protocol prohibited vaccines or drugs:

  ① Other investigational or unlicensed products (drugs or vaccines);

  ② Long-term use (lasting more than 14 days) of immunosuppressive or other immunomodulatory drugs (inhaled or topical steroids are allowed);

  ③ Immunoglobulin and/or blood preparations.
- Newly diagnosed autoimmune diseases, including human immunodeficiency virus (HIV) infection;
- Other situations affecting the evaluation of immunogenicity of 28 days after booster dose vaccination.

Immune Persistence Set of Booster Immunization, 6 months after booster dose vaccination (bIPS-6): includes all the subjects who have received the 1st and 2nd dose vaccination, completed the booster dose vaccination 6 months after the second dose, and blood sample is collected 6 months after the booster dose vaccination, and there is an

effective antibody titer value.

Immune Persistence Set of Booster Immunization, 12 months after booster dose vaccination (bIPS-12): includes all the subjects who have received the 1st and 2nd dose vaccination, completed the booster dose vaccination 6 months after the second dose, and blood sample is collected 12 months after the booster dose vaccination, and there is an effective antibody titer value..

10.11.2 Statistical Analysis Methods

10.11.2.1 General Principles

Measuring data are described by means, standard deviations, medians, maximum, and minimum value; counting data or grade data are described by frequencies. All statistical analyses are performed using statistical software SAS 9.4.

10.11.2.2 Characteristics of Subject Population

The number of subjects who were screened, enrolled and completed the trial, and the number of subjects in each statistical analysis data set should be summarized, and the reasons for the dropout should be analyzed. The list of subjects who failed in screening, who dropped out and who did not enter each analysis set were listed separately.

10.11.2.3 Evaluation of Immunogenicity

The seroconversion rate and positive rate of antibodies in the medium dosage group, high dosage group, and placebo group should be calculated respectively, and Clopper-Pearson method will be adopted to calculate the corresponding 95% confidence interval. Chi-square test/Fisher exact probability method will be used to conduct statistical test on difference between groups.

Geometric mean titer (GMT) and geometric mean increase (GMI) of the serum antibody, as well as the corresponding 95%CI in the experimental group and the control group should be calculated, and

and the difference between groups was statistically tested using the ANOVA after log-transfermation of the anytibody titer.

10.11.2.4 Evaluation of Safety

Adverse events were medically coded using MedDRA. This study mainly analyzes the adverse events after vaccination, and the adverse events before vaccination will be listed.

The number of episodes, number of involved subjects, as well as the incidence rate of overall AEs, the vaccine-related AEs, and the vaccine-unrelated AEs in all the groups should be calculated separately, and the differences between groups will be statistically tested using the Fisher exact probability method. The severity, dose distribution and time distribution of general AEs as well as the vaccine-related AEs should be statistically analyzed. The list of the vaccine-related AEs, vaccine-unrelated AEs should be made separately. The AEs after each dose should be statistically analyzed based on the safety set of each dose respectively.

The number of epidoses, number of involved subjects, as well as the incidence rate of overall SAEs, the vaccine-related SAEs, and the vaccine-unrelated SAEs in all groups should be calculated separately, and the differences between groups will be statistically tested using the Fisher exact probability method. The list of the SAEs should be made.

10.11.2.5 Processing of Missing Data

With regard to the statistical analysis of FAS, the missing data of the post-vaccination antibody test result will be filled by the method of Last Observation Carried Forward (LOCF). The missing data of the pre-vaccination antibody result will be filled with the maximum of pre-vaccination antibody results among all the subjects. In terms of the evaluation of the exploratory and safety endpoints,

missing data will not be filled.

11 Monitoring of Clinical Trials

11.1 Responsibilities of the Sponsor

The sponsor executes and maintains the quality assurance and quality control system, compiles quality management documents to ensure that the clinical trial is carried out in accordance with regulations, and that data, records and reports meet the requirements of GCP, other regulations and the protocol.

11.2 Responsibilities of the Investigator

The principal investigator should manage and clearly divide all personnel involved in the clinical trial. The personal data of the subjects should be kept confidential by the investigators. Documents provided to the sponsor should be identified only by the subject number. The identification list of subjects is kept in the investigator documents. In accordance with GCP principles, the original materials of each subject are allowed to be monitored, audited and verified.

11.3 Personnel Training

Before the start of the trial, the staff should be trained. The training contents include GCP principles, clinical trial protocol, SOP, etc. If the sponsor or principal investigator deems it necessary, retraining may be conducted. Each training should have training records.

11.4 Subject Compliance Guarantee

According to the clinical trial protocol, a concise, clear and well organized volunteer recruitment form and informed consent form were formulated.
Train the doctor responsible for the informed explaination to communicate with volunteers in plain and understandable language so as to be fully informed.
Screen the subjects strictly according to the inclusion and exclusion criteria.
The Follow-up personnel should have a high sense of responsibility and dedication to improve their communication skills and affinity through training. In the process of safety follow-up, measures should be taken to ensure effective contact between subjects and investigators, and adverse reactions should be disposed timely with the related health consultation provided.

11.5 Vaccine Management in Clinical Trials

11.5.1 Definition and Treatment of Cold Chain Failure

Once the refrigerator storing the vaccine has a temperature of <2℃ and >8°C, it is recorded as cold chain failure. Once there is a cold chain failure, the vaccine should be transported to a light-protected environment for storage as soon as possible, with reprting to the sponsor in time. The decision of whether stop or continue using the vaccine should be made according to the written/e-mail responses from sponsor.

11.5.2 Receiving of Vaccine for Trial

When the sponsor delivers the trial vaccine to the study site, the investigator must sign the vaccine receipt form, on which the information (e.g. complete package, and normal cold chain system indication etc.) should be described briefly. When the investigator finds that the vaccine is damaged, spoiled, or has lumps that cannot be shaken in it, such vaccine should be prohibited to be used, and should be returned to the sponsor. In the case of cold chain failure or freeze during the transport or storage process, the vaccine can not be used. The vaccine with the above problems should be marked with '×' on the surface of the outer package and stored separately, managed by a dedicated staff and finally returned to the sponsor.

11.5.3 Management of Trial Vaccines

Trial vaccines should be managed by a dedicated staff and supervised by CRA. The vaccine receipt and transfer record should include the number of received vaccine, the number of vaccinated subjects, the number of remaining vaccine and the number of losses. The investigators will calculate the number of all the trial vaccine.When the field work is completed, the remaining trial vaccines are counted and returned to the sponsor when the study site.

11.6 Management of Clinical Trial Sample

Specimens used for neutralizing antibody test should be disposed by the testing institution as medical waste after completion of the testing. The backup serum is temporarily stored by the study site institution, until a verified immunogenicity test report is issued by the testing institution. The backup serum can be stored or processed by the sponsor after the project is completed, and its use needs the approval of the ethics committee and the informed consent of the subjects.

11.7 Preservation of Clinical Trial Documents

The clinical trial documents must be kept according to the requirements of Chineses GCP. The sponsor, study institution and studysite should keep the clinical trial data for at least 5 years after the drug is marketed.

11.8 Ending Criteria for Clinical Trials

-Samples collected in the clinical trial are sent to the testing institution, and the corresponding testing reports are issued.
-All subjects completed the required visit, and the original data and documents of the clinical trial are transferred to the archivist for archiving and preservation;
-The remaining amount of the trial vaccine is accurate and handed over to the sponsor;
-The statistical analysis report and summary report meet the requirements of the sponsor.

12 Ethical Approval

12.1 Review and Approval

This clinical trial protocol should be approved by the local ethics committee. The principle investigator submits the clinical trial protocol and all the necessary additional documents to the ethics committee. After the approval of the ethics committee, the investigator provides the sponsor with a certificate of approval from the ethics committee.

12.2 Implementation of On-site Supervision

In the whole process of the trial, the ethics committee should supervise whether there are ethical problems of harming the subjects, whether the subjects get treatment, compensation and corresponding measures when they are harmed by the trial, and evaluate the degree of risk they bear.

12.2.1 Informed Consent Form and Informed Consent

It should be ensured that the method of selection of subjects and relevant information provided to subjects are complete and easy to understand, and the method of obtaining informed consent is appropriate. During the whole process of the trial, the ethics committee should regularly review the progress of the trial and assess the risks and benefits of the subjects.

12.2.2 The Potential Hazards and Hazard Minimization

If the adverse reactions are determined to be related to vaccination (the injection site abscess and rash after vaccination), the subjects will be treated in time according to relevant regulations.

If a life-threatening event occurs, the subject will be escorted to the hospital for treatment immediately and the corresponding report should be made.

Under strict supervision, the trained and experienced medical personnel conduct vaccination and venous blood collection in accordance with the prescribed procedures, so as to minimize the injury and suffering caused by vaccination and blood collection (including pain and local infection at the venipuncture site with little probability).

12.2.3 Protection Measures for Subjects

Clinical trials were conducted in county/city centers for Disease Control and prevention with vaccination qualifications. The sponsor examine the study site strictly according to the requirement of the GCP, before the start of the clinical trial. The environment and facilities of the study site should meet the requirements of *The Guiding Principles for Quality Management of Vaccine Clinical Trials (Trial)*. The emergency plan for the damage and emergency of the subjects should be prepared by the study site. Doctors and nurses with corresponding qualifications and experience should be arranged in the physical examination room and blood collection room in order to strictly grasp the inclusion/exclusion criteria and collect blood smoothly. The first-aid room should be equipped with appropriate first-aid facilities, equipment and drugs, and the first-aid doctors shall have corresponding qualifications and capabilities. When the subjects have adverse events at the study site, they should be treated in the on-site emergency room in time. If they need emergency hospitalization treatment, the ambulance equipped on the study site will send the subjects to the agreement hospital for treatment after the condition is stable though the on-site treatment. The ambulance should also be equipped with necessary first-aid facilities and drugs.

The study site signs a green channel agreement with a local county-level and above general hospital.

During the enrollment of the subjects, the agreement hospital should be notified to prepare for timely treatment. Measures shall be taken to ensure that the emergency adverse events can be dealt with in a timely manner, such as personnel responsibilities, telephone number and rescue route. The effective contact between the subjects and the investigator should be maintained, so that any adverse events can be reported and disposed quickly. When the subjects need to be hospitalized for emergency treatment after serious adverse events, the agreement hospital can provide green channel services such as medical treatment, hospitalization and medical security, to ensure that the subjects can be treated in time. The investigator followed up the progress of the event and completed the investigation record until the end of the serious adverse event.

12.3Confidentiality

It should be ensured that the personal secrets of the subjects are not disclosed under the conditions of the trial enrollment, biological sample collection, report and publication. The recorded information of the test samples only includes the subject number, sample number, sampling time, and testing indicators. Only the main personnel of the study have the anthority to obtain electronica or written copies.

13 Revision of Clinical Trial Protocol

After the sponsor and investigator sign the clinical trial protocol, if there is any modification to the protocol, all the modified protocols shall be re signed and dated by the main investigator and sponsor, and the protocol before modification shall be attached.

All modification plans shall be reported to the ethics committee and approved by the ethics committee before implementation. When modifying the scheme, it is necessary to point out whether it is necessary to modify the informed consent form and electronic CRF form.

14 The Publicity and Publication of Data

After the completion of this clinical trial, if the test results need to be open and/or published, the positive results and the negative results will be open and/or published together.

15 References

1. Former CFDA, *Measures for the Administration of Drug Registration*, 2007.
2. Former CFDA, *Code for Quality Management of Drug Clinical Trials*, 2003.
3. Former CFDA, *Technical guidelines for clinical trials of vaccines*, 2004.
4. Former CFDA, *Guidelines for quality management of vaccine clinical trials (Trial)*, 2013.
5. Drug Evaluation Center. *Guidelines for Novel Coronavirus Preventive Vaccine Research and Development Technology (Trial)* .March, 2020.
6. Lu R, Zhao X, Li J, Niu P, Yang B, Wu H, Wang W, Song H, Huang B, Zhu N, et al. Genomic characterisation and epidemiology of 2019 novel coronavirus: implications for virus origins and receptor binding. Lancet (London, England). 2020;6736(20):1-10.
7. Chen Y, Liu Q, Guo D. Coronaviruses: genome structure, replication, and pathogenesis. Journal of medical virology. 2020:0-2.
8. Information Office of the State Council. Novel coronavirus infection prevention and control work conference. Beijing, Jan 26, 2020.
9. Diagnosis and treatment of novel coronavirus infection pneumonia (trial version 7) .
10. Holshue ML, DeBolt C, Lindquist S, et al. First Case of 2019 Novel Coronavirus in the United States. N Engl J Med. 2020 Mar 5;382(10):929-936. .
11. Expert group on novel coronavirus pneumonia prevention and control of China Preventive Medicine Association . The latest understanding of novel coronavirus pneumonia epidemiology . Chinese Journal of Epidemiology, 2020-02-14.
12. https://www.who.int/emergencies/diseases/novel-coronavirus-2019/situation-reports.
13. Disease control and Prevention Bureau of national health and Health Commission . China - A novel coronavirus pneumonia in WHO (COVID-19) Joint investigation report .
14. Former SFDA, *Guidelines for the classification of adverse events in clinical trials of preventive vaccines*, 2019.
