# Supplementary material for: Six-month follow-up of a booster dose of CoronaVac in two single-centre phase 2 clinical trials
Source: Nat Commun. 2022 Jun 3;13:3100. doi: 10.1038/s41467-022-30864-w (PMC9166693; doi:10.1038/s41467-022-30864-w)
Supplement: Supplementary file 3 — Reporting Summary [file 41467_2022_30864_MOESM3_ESM.pdf]

nature portfolio

Corresponding author(s): Minjie Li, uliang Zhao, Hongxing Pan, Hongjie Yu, Lin Wang

Last updated by author(s): May 8, 2022

Reporting Summary

Nature Portfolio wishes to improve the reproducibility of the work that we publish. This form provides structure for consistency and transparency in reporting. For further information on Nature Portfolio policies, see our Editorial Policies and the Editorial Policy Checklist.

Statistics

For all statistical analyses, confirm that the following items are present in the figure legend, table legend, main text, or Methods section.

n/a	Confirmed	
☐	☒	The exact sample size (n) for each experimental group/condition, given as a discrete number and unit of measurement
☒	☐	A statement on whether measurements were taken from distinct samples or whether the same sample was measured repeatedly
☐	☒	The statistical test(s) used AND whether they are one- or two-sided<br>*Only common tests should be described solely by name; describe more complex techniques in the Methods section.*
☒	☐	A description of all covariates tested
☐	☒	A description of any assumptions or corrections, such as tests of normality and adjustment for multiple comparisons
☐	☒	A full description of the statistical parameters including central tendency (e.g. means) or other basic estimates (e.g. regression coefficient) AND variation (e.g. standard deviation) or associated estimates of uncertainty (e.g. confidence intervals)
☐	☒	For null hypothesis testing, the test statistic (e.g. F, t, r) with confidence intervals, effect sizes, degrees of freedom and P value noted<br>*Give P values as exact values whenever suitable.*
☒	☐	For Bayesian analysis, information on the choice of priors and Markov chain Monte Carlo settings
☒	☐	For hierarchical and complex designs, identification of the appropriate level for tests and full reporting of outcomes
☒	☐	Estimates of effect sizes (e.g. Cohen's d, Pearson's r), indicating how they were calculated

Our web collection on statistics for biologists contains articles on many of the points above.

Software and code

Policy information about availability of computer code

Data collection	In this trial, Electronic Data Capture (RIEHEN, Version: 2.1.1608) is used to establish the electronic CRF. As an important component of the clinical trials and research reports, the electronic CRF is used to record clinical trial data. Information should be inputted with standard language according to the EDC instructions and CRF filling instructions.<br><br>Randomisation codes for each vaccination schedule cohort were generated individually and randomly assigned using block randomization developed with SAS version 9.4.
Data analysis	All data cleaning, statistical analyses, and visualizations were performed in R (version 4.0.2).

For manuscripts utilizing custom algorithms or software that are central to the research but not yet described in published literature, software must be made available to editors and reviewers. We strongly encourage code deposition in a community repository (e.g. GitHub). See the Nature Portfolio guidelines for submitting code & software for further information.

Data

Policy information about availability of data

All manuscripts must include a data availability statement. This statement should provide the following information, where applicable:

- Accession codes, unique identifiers, or web links for publicly available datasets
- A description of any restrictions on data availability
- For clinical datasets or third party data, please ensure that the statement adheres to our policy

To protect participants' confidentiality, the individual participant data that underlie the results reported in this article (text, tables, figures and extended data) will only be shared after de-identification. Due to the clinical trial in adults aged 60 years and older is ongoing, in order to maintain the blind status of this trial, the data

will be available following clinical study report (CSR) of immune persistence analysis (September 2022). Researchers who provide a scientifically sound proposal will be allowed to access to the individual participant data. Proposals should be directed to wanglin@sinovac.com.

The infectious SARS-CoV-2 virus strain used in the neutralization assay: SARS-CoV-2/human/CHN/CN1/2020, GenBank accession number MT407649.1.

Field-specific reporting

Please select the one below that is the best fit for your research. If you are not sure, read the appropriate sections before making your selection.

- [x] Life sciences
- [ ] Behavioural & social sciences
- [ ] Ecological, evolutionary & environmental sciences

For a reference copy of the document with all sections, see nature.com/documents/nr-reporting-summary-flat.pdf

Life sciences study design

All studies must disclose on these points even when the disclosure is negative.

Sample size

The sample size was determined following requirements of the National Medical Products Administration, China's regulatory authority for vaccines.

Data exclusions

Exclusion Criteria for Subjects:
(1) Having traveled to or lived in Wuhan or surrounding areas or communities where confirmed cases have been reported within the previous 14 days;
(2) Having contact with patients who were infected with COVID-19 (who have tested positive for nucleic acid detection) within the previous 14 days;
(3) Having contact with patients who have fever and symptoms of respiratory infections from Wuhan or surrounding areas or communities where confirmed cases have been reported within the previous 14 days;
(4) Having been in places such as houses, offices and classrooms where over 2 cases of fever and/or symptoms of respiratory infections have been reported within the previous 14 days;
(5) SARS record in self-report;
(6) Infection with COVID-19 recorded in self-report;
(7) IgG or IgM screening results were positive;
(8) The RT-PCR test results of throat and anal swabs were positive;
(9) Women who are breastfeeding, pregnant, or planning to become pregnant during the study period (Judgment is made based on subjects' self-report and urine pregnancy test results);
(10) Body mass index (BMI) ≥35 kg/m2;
(11) Having a history of asthma and allergy to vaccine or vaccine ingredients and having serious adverse reactions to the vaccine such as urticaria, dyspnea and angioneurotic edema;
(12) Subjects with congenital malformations or developmental disorders, genetic defects, severe malnutrition, etc.;
(13) Subjects with autoimmune diseases or immune deficiency/immune inhibition;
(14) Subjects with severe chronic diseases, severe cardiovascular diseases and hypertension, diabetes, liver and kidney diseases and malignant tumors that can not be controlled by drugs;
(15) Subjects with serious neurological diseases (epilepsy, convulsions or tic) or mental diseases;
(16) Subjects with thyropathy or having a history of thyroidectomy, subjects with an absent or dysfunctional spleen and subjects with an absent spleen or splenectomy;
(17) Subjects with coagulation disorders diagnosed by a doctor (such as deficiency of coagulation factors, coagulation diseases and blood platelet disorders) or significant bruising or coagulation disorder;
(18) Having received the immunosuppressive therapy, cytotoxic therapy, and inhale corticosteroids (excluding corticosteroid spray in treatment of allergic rhinitis and surface corticosteroid treatment of acute non-concurrent dermatitis) in the past six months;
(19) Subjects with abnormal laboratory test results such as in hematology and biochemistry which are beyond the range of reference values and of clinical significance in physical examination (applicable for Phase I clinical trial only):
  1) Blood routine test: white blood cell count, hemoglobin and platelet count.
  2) Blood biochemistry: alanine aminotransferase (ALT), aspartate aminotransferase (AST), total bilirubin (TBIL), creatinine (CR) and creatine phosphokinase (CPK);
  3) Routine urine indexes: urine protein (PRO), urine sugar and urine erythrocyte
(20) Chronic alcoholics or those having a history of drug abuse;
(21) Subjects who have received blood products within 3 months before vaccination with the test vaccine;
(22) Subjects who have received other study drugs within 30 days before vaccination with the test vaccine;
(23) Subjects who have received live attenuated vaccines within 14 days before vaccination with the test vaccine;
(24) Subjects who have received subunit or inactivated vaccines within 7 days before vaccination with the test vaccine;
(25) Subjects having an attack of various acute or chronic diseases within 7 days;
(26) Subjects with axillary temperature >37.0خ before vaccination with the test vaccine;
(27) Subjects who are not suitable for participating in this clinical trial according to the investigator.

Exclusion Criteria for Second and Third Doses of Vaccination:
Subjects with any adverse event listed in (1) to (4) are forbidden to continue vaccination but they can finish other research based on the investigator's judgment. For subjects with any adverse event listed in (5) and (6), it is up to the investigator to decide whether or not to vaccinate. For subjects with any adverse event listed in (7) to (10), the vaccination can be delayed within the time window specified in the

schedule.
(1) Vaccines of the same type other than the test vaccine are used during the study period;
(2) Any serious adverse reactions that have a causal relationship with the test vaccine;
(3) Allergic shock or hypersensitivity after vaccination (including urticaria/rash that appears within 30 minutes after vaccination);
(4) Any confirmed or suspected autoimmune diseases or immunodeficiency diseases, including human immunodeficiency virus (HIV) infection;
(5) Acute or newly developed chronic diseases after vaccination;
(6) Other reactions as determined by the investigator (including severe pain, severe swelling, severe limitation of motion, persistent high fever, severe headache, or other systemic or local reactions);
(7) With acute illness (moderate or severe illness with or without fever) at the time of vaccination;
(8) Axillary temperature >37.0خ at the time of vaccination;
(9) Having received subunit or inactivated vaccines within 7 days and having received live attenuated vaccines within 14 days.
(10) Any other causes for which subjects are not suitable for vaccination according to the investigator.

Replication

This study represents the phase II clinical trial about the immune response induced by CoronaVac, an inactivated vaccine, no replication is performed in this study. We have the protocol with the manuscript to ensure the reproducibility of this study.

Randomization

Randomisation codes for each vaccination schedule cohort were generated individually and randomly assigned using block randomization developed with SAS version 9.4. Adults aged 18-59 years were assigned with a block size of five and adults aged 60 years and older were assigned with a block size of fourteen. Concealed random group allocations and blinding codes were kept in signed and sealed envelopes. The randomisation code was assigned to each participant in sequence in the order of enrolment by investigators, who were involved in the rest of the trial.

Blinding

Investigators, participants, and laboratory staff were masked to group assignment.

Reporting for specific materials, systems and methods

We require information from authors about some types of materials, experimental systems and methods used in many studies. Here, indicate whether each material, system or method listed is relevant to your study. If you are not sure if a list item applies to your research, read the appropriate section before selecting a response.

Materials & experimental systems

n/a	Involved in the study	
x		Antibodies
	x	Eukaryotic cell lines
x		Palaeontology and archaeology
x		Animals and other organisms
	x	Human research participants
	x	Clinical data
x		Dual use research of concern

Methods

n/a	Involved in the study	
x		ChIP-seq
x		Flow cytometry
x		MRI-based neuroimaging

Eukaryotic cell lines

Policy information about cell lines

Cell line source(s)

The Vero cells used in neutralization assay are obtained from kidney tissue of adult African green monkey (W.H.O.VERO SEED LOT 10-87), which was the 10th generation passaged from original ATCC Vero CCL-81.

Authentication

Cell line was authenticated by cell morphology examination and species identification (PCR).

Mycoplasma contamination

Cell line was confirmed to be tested negative for mycoplasma by culture method and indicator cell culture medium (DNA staining).

Commonly misidentified lines
(See ICLAC register)

Not available.

Human research participants

Policy information about studies involving human research participants

Population characteristics	For the adults aged 18-59 years old, mean ages of participants included in the immune persistence analysis were between 40.4 years (SD 10.3) and 45.7 years (SD 9.7) in four vaccination cohorts (cohort 1a-14d-2m, cohort 1b-14d-8m, cohort 2a-28d-2m, cohort 2b-28d-8m). The proportion of males were from 35% to 63% in the vaccination groups and from 39% to 54% in the placebo groups.<br><br>For the adults aged 60 years or older, the mean age of the 303 treated participants was between 66.3 years (SD 4.4) and 67.1 (SD 4.7) years old in four treatment groups. The proportions of males were between 46% and 57%.
Recruitment	Recruitment notices will be issued to volunteers who meet the enrollment criteria. The informed consent will be explained to the volunteers in detail. Under the condition of voluntary participation, the volunteers and the study doctors sign the informed consent which is in duplicate, and the copy is reserved by the volunteer.<br><br>Subjects who are normal in physical examination (Women undergo a urine pregnancy test to rule out pregnancy.) and screened qualified as per other inclusion/exclusion criteria (Screening No. consists of S and screening order, such as “S0001”.) will be enrolled and given Research Number based on enrollment order.
Ethics oversight	The complete study protocol for adults aged 18-59 years old was approved by the ethics committees of Jiangsu Provincial Centre for Disease Control and Prevention (JSJK2020-A021-02), and the complete study protocol for adults aged 60 years and older was approved by the ethics committees of Hebei Provincial Centre for Disease Control and Prevention (IRB2020-006).

Note that full information on the approval of the study protocol must also be provided in the manuscript.

Clinical data

Policy information about clinical studies

All manuscripts should comply with the ICMJEguidelines for publication of clinical research and a completedCONSORT checklist must be included with all submissions.

Clinical trial registration	NCT04352608 and NCT04383574.
Study protocol	Study protocols are provided in supplementary files.
Data collection	The initial trial involving 600 healthy adults aged 18-59 years old in a single-centre, double-blind, randomised, placebo-controlled, phase 2 clinical trial conducted from May 3, 2020 in Suining county, Jiangsu province, China. Last participant's visit was on 24th July 2021.<br><br>The other trial, involving 350 healthy adults aged 60 years and older, was a single-centre, double-blind, randomised, placebo-controlled, phase 2 clinical trial conducted from June 12, 2020 in Renqiu county, Hebei province, China. Last participant's visit was on 24th September 2021.
Outcomes	Phase 2 trial among adults aged 18-59 years old<br>Primary endpoint<br>- Positive conversion rate of serum neutralizing antibody on Day 14 (Cohort 1)/Day 28 (Cohort 2) after two doses of test vaccine;<br>- Incidence of adverse reaction on Day 0-28 (Day 0-14 for the first dose for Cohort 1) after each dose;<br>Secondary endpoint<br>- Positive rate, GMT and GMI of serum neutralizing antibody on Day 28 after two doses of test vaccine;<br>- Positive conversion rate, positive rate, GMT and GMI of serum neutralizing antibody on Day 28 after three doses of test vaccine (only for Cohort 1a-14d-2m and Cohort 2a-28d-2m);<br>- Incidence of adverse reactions 0-7 days after each dose of vaccination;<br>- Incidence of serious adverse event from the inoculation to 6 months after full course vaccination;<br>Exploratory Endpoints<br>- The seropositive rate and GMT of neutralizing antibody at 6 months after the second dose (only for Cohort 1b-14d-8m and Cohort 2b-28d-8m);<br>- The seropositive rate and GMT of neutralizing antibody at 12 months after the third dose (only for Cohort 1a-14d-2m and Cohort 2a-28d-2m);<br>- The seropositive rate and GMT of neutralizing antibody at 14 days (only for<br>Cohort 1b-14d-8m) or 28 days (only for Cohort 2b-28d-8m) after the booster dose;<br>- The seropositive rate and GMT of neutralizing antibody at 6 months after the booster (only for Cohort 1b-14d-8m and Cohort 2b-28d-8m).<br><br>Phase 2 trial among adults aged 60 years and older<br>Primary Endpoint<br>- Incidence of adverse reactions within 28 days after each dose of vaccination;<br>- The seroconversion rate of neutralizing antibodies 28 days after the second dose vaccination.<br>Secondary Endpoint<br>- Incidence of adverse reactions within 7 days after each dose vaccination;<br>- Incidence of SAEs from the beginning of the vaccination to 12 months after booster immunization;<br>- The seropositive rate, GMT, and GMI of neutralizing antibodies 28 days after the second dose vaccination;

Exploratory Endpoint
- The seropositive rate and GMT 6 months after the second dose vaccination;
- The seropositive rate, GMT, and GMI 28 days after the booster vaccination;
- The seropositive rate and GMT 6 months after the booster vaccination;
- The seropositive rate and GMT 12 months after the booster vaccination.

Blood samples were collected at pre-specified visits and subject to neutralizing antibody test. Neutralizing antibodies against infectious SARS-CoV-2 (virus strain SARS-CoV-2/human/CHN/CN1/2020, GenBank accession number MT407649.1, https://www.ncbi.nlm.nih.gov/nuccore/MT407649.1) were quantified using a microcytopathogenic effect assay. Serious adverse events were recorded for 6 months after the third dose for participants in every cohort. Serious adverse events were coded by the Medical Dictionary for Regulatory Activities (MedDRA) System Organ Class. Existence of causal associations between adverse events and vaccination were determined by the investigators.
